# Synthesis of Biologically Active Molecules through Multicomponent Reactions

**DOI:** 10.3390/molecules25030505

**Published:** 2020-01-24

**Authors:** Daniel Insuasty, Juan Castillo, Diana Becerra, Hugo Rojas, Rodrigo Abonia

**Affiliations:** 1 Grupo de Investigación en Química y Biología, Departamento de Química y Biología, Universidad del Norte, Km 5 vía Puerto Colombia 1569, Barranquilla Atlántico 081007, Colombia; insuastyd@uninorte.edu.co; 2 Grupo de Catálisis, Escuela de Ciencias Químicas, Universidad Pedagógica y Tecnológica de Colombia UPTC, Avenida Central del Norte 39-115, Tunja 150003, Colombia; juan.castillo06@uptc.edu.co (J.C.); diana.becerra08@uptc.edu.co (D.B.); hugo.rojas@uptc.edu.co (H.R.); 3 Bioorganic Compounds Research Group, Department of Chemistry, Universidad de los Andes, Carrera 1 No. 18A-10, Bogotá 111711, Colombia; 4 Research Group of Heterocyclic Compounds, Department of Chemistry, Universidad del Valle, Cali A. A. 25360, Colombia

**Keywords:** multicomponent reactions (MCRs), medicinal chemistry, biological activity, drug discovery

## Abstract

Focusing on the literature progress since 2002, the present review explores the highly significant role that multicomponent reactions (MCRs) have played as a very important tool for expedite synthesis of a vast number of organic molecules, but also, highlights the fact that many of such molecules are biologically active or at least have been submitted to any biological screen. The selected papers covered in this review must meet two mandatory requirements: (1) the reported products should be obtained via a multicomponent reaction; (2) the reported products should be biologically actives or at least tested for any biological property. Given the diversity of synthetic approaches utilized in MCRs, the highly diverse nature of the biological activities evaluated for the synthesized compounds, and considering their huge structural variability, much of the reported data are organized into concise schemes and tables to facilitate comparison, and to underscore the key points of this review.

## 1. Introduction

**Figure 1:**
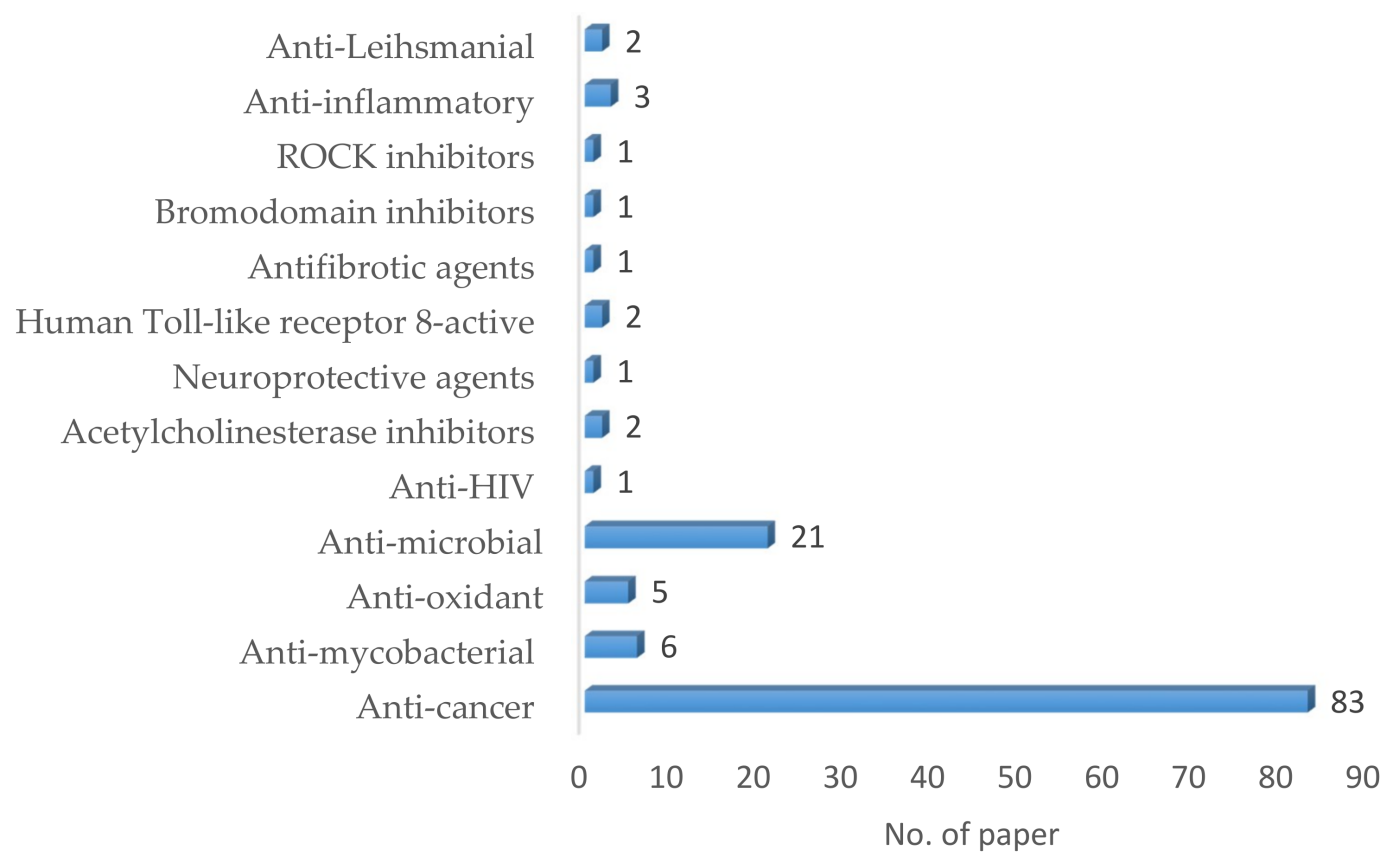
Bibliometric graphic depicting the number of published research papers vs the type of biological activity screened [Data were collected searching for the keywords “multicomponent reactions” and “biologically active molecules” by the period 2002 to date.

Multicomponent reactions (MCRs) are a type of convergent organic reactions in which three or more precursors react in only one step to form a product that incorporates substantial portions of all components (i.e., atom economy) [1]. As a result, there is very little waste or unwanted by-product formation compared to sequential synthesis. The concomitant step economy, high convergence and structural diversity of the resulting products make this sustainable approach a powerful tool for the synthesis of biologically active molecules and optimization processes in the pharmaceutical industry [2]. Even though the MCR concept for the synthesis of diverse organic structures has been well known for over a century, it has only recently started gaining more attention and there is an increasing number of research articles emphasizing on the synthesis of biologically relevant organic molecules via this approach [3]. Such an attempt is presented in this review paper with an emphasis on organic molecules synthesized via MCRs and subjected to screening for biological activity. It was found that most of such screening was focused on anti-leihsmanial, anti-inflammatory, ROCK inhibitors, bromodomain inhibitors, antifibrotic agents, human toll-like receptor 8-active, neuroprotective agents, acetylcholinesterase inhibitors, anti-HIV, antimicrobial, antioxidant, anti-mycobacterial and anticancer activities (Figure 1). It is noteworthy that 64% and 16% of the supporting literature found for this review correspond to heterocyclic structures with anticancer and antimicrobial activities, respectively.

## 2. Some Aspects of the Multicomponent Reactions

Reactions like Strecker (1850) [4], Hantzsch (1890) [5], Biginelli (1893) [6], Mannich (1912) [7], Passerini (1921) [8], Asinger (1956) [9] and Ugi (1959) [10], among others, are good classical examples of MCRs. In a MCR, the product is assembled according to a cascade of elementary two-component reactions. Thus, there is a network of reaction equilibria, which all finally flow into an irreversible step to afford the expected product. The use of MCRs in all areas of the applied chemistry are very popular because they offer a wealth of products, while requiring only a minimum of effort. As opposed to the classical way to synthesize complex molecules by sequential synthesis, MCRs allow the assembly of complex molecules in a one-pot manner. Unlike the usual stepwise formation of individual bonds in the target molecule via a multi-step synthetic approach, the defining attribute of MCRs is the inherent formation of several bonds in one operation without isolating the intermediates (referred to as the bond-forming efficiency, BFE) [1,11,12], nor changing the reaction conditions or adding further reagents.

Recently, chemists have renewed their interest in MCRs. This is driven in part, by the pharmaceutical industry due to the growing need to assemble libraries of small-molecules structurally complexes for evaluation as lead scaffolds in drug discovery and development programs. New libraries of such scaffolds are becoming more and more requested after as pathogens mutate to become resistant to current medications. In addition, anti-aging agents are needed for treatment of Alzheimer’s, Parkinson’s, diabetes and cancer, among other diseases [13]. Thus, MCRs represent an excellent tool for the generation of such libraries, which are indispensable for structure–activity relationship (SAR) studies in drug discovery programs.

## 3. Biologically Active Compounds Obtained from Multicomponent Approaches

As multicomponent reactions represent a powerful tool in the repertoire of sustainable organic synthesis its synergistic utilization with other green chemistry principles would bring organic chemists one-step closer to the ideal synthesis [14]. The rapid and efficient access to a plethora of heterocyclic building blocks through multicomponent reactions have been recognized by the synthetic community as a preferred strategy to design and synthesize biologically active compounds [15,16].

The wide variety of MCR procedures applied to the synthesis of relevant organic molecules clearly shows that MCR-based approaches are exceptionally useful for drug discovery and optimization processes in the pharmaceutical industry due to its high atom-economy, operational simplicity, time/cost efficiency and generation of structural diversity from multifunctional substrates [17,18]. In fact, the growing number of acyclic and heterocyclic building blocks on the market and in clinical evaluation discovered and synthesized by MCR approaches manifests their growing importance in medicinal chemistry and drug discovery programs. The corresponding biological activities displayed by the diverse organic compounds synthesized via MCRs approaches during the period 2002 to date will be discussed as follow.

### 3.1. Anti-Leihsmanial Activity

**Scheme 1:**
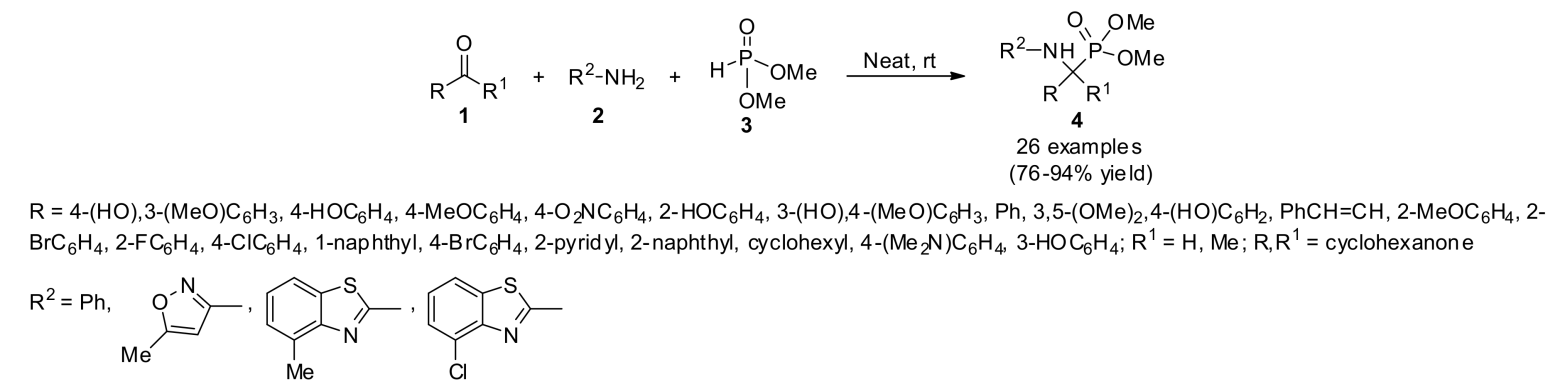
One-pot three-component reaction for the synthesis of diverse *α*-aminophosphonates **4** and their biological activities.

Leishmaniasis, a parasitic disease causes a major public health problem, which is prevalent in some tropical and sub-tropical areas of the world. One of its types, visceral leishmaniasis (VL), also known as kala-azar, is highly endemic in the Indian subcontinent and in East Africa. It is transmitted by the bite of infected female phlebotomine sandflies belonging to the genus *Leishmania* [19]. The existing chemotherapies are not effective enough as these have various drawbacks such as significant toxicity, variable efficacy, lack of oral bioavailability, and high cost involved during the treatment [20]. Thus, for the global health programs there has been a pressing need for the discovery of new lead compounds for the treatment of leishmaniasis [21]. In that direction, a series of structurally diverse *α*-aminophosphonates **4** were synthesized and evaluated for in vitro anti-leishmanial activity and cytotoxicity using the MTT assay (Scheme 1). Compounds **4** were prepared through a three-component reaction involving aldehydes/ketones **1**, amines **2**, and phosphites **3** via a Kabachnik–Fields type reaction under catalyst- and solvent-free reaction conditions at room temperature [22]. Several of the obtained compounds exhibited anti-leishmanial potency against the *L. donovani* promastigote with IC_50_ values in the low micromolar range. The structure–activity relationships were quantitatively evaluated by a statistically reliable CoMFA model with high predictive abilities (r^2^_pred_ = 0.87, r^2^_ncv_ = 0.985) [23].

### 3.2. Anti-Inflammatory Activity

Bacterial infection and inflammation are strongly interrelated with each other. Pain and inflammation often arise due to bacterial infections [24]. Inflammation is nothing but a biological process that arises due to physical, chemical, biological and immunological stimuli to the human body [25], for that, inflammation is the significant indication in numerous pathological conditions such as Alzheimer’s disease, osteoarthritis, rheumatoid arthritis and obesity-related diseases [26,27].

**Scheme 2:**
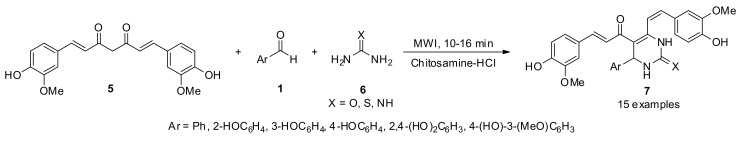
Three-component synthesis of 3,4-dihydropyrimidinones/thiones type **7** under microwave irradiation, for anti-inflammatory activity.

In this sense, a series of curcumin 3,4-dihydropyrimidinones/thiones/imines **7** were synthesized in 90–96% yield through a one-pot multicomponent cyclocondensation reaction between curcumin (**5**), substituted aromatic aldehydes **1** and urea/thiourea/guanidine **6** in the presence of chitosamine hydrochloride as a biodegradable and non-toxic catalyst under solvent-free microwave irradiation (MWI) (Scheme 2). All the synthesized curcumin derivatives **7** were screened for anti-inflammatory (but also for antioxidant) activities. The biological activity data of the synthesized compounds showed that most of them exhibited greater anti-inflammatory activity than curcumin [28].

**Scheme 3:**
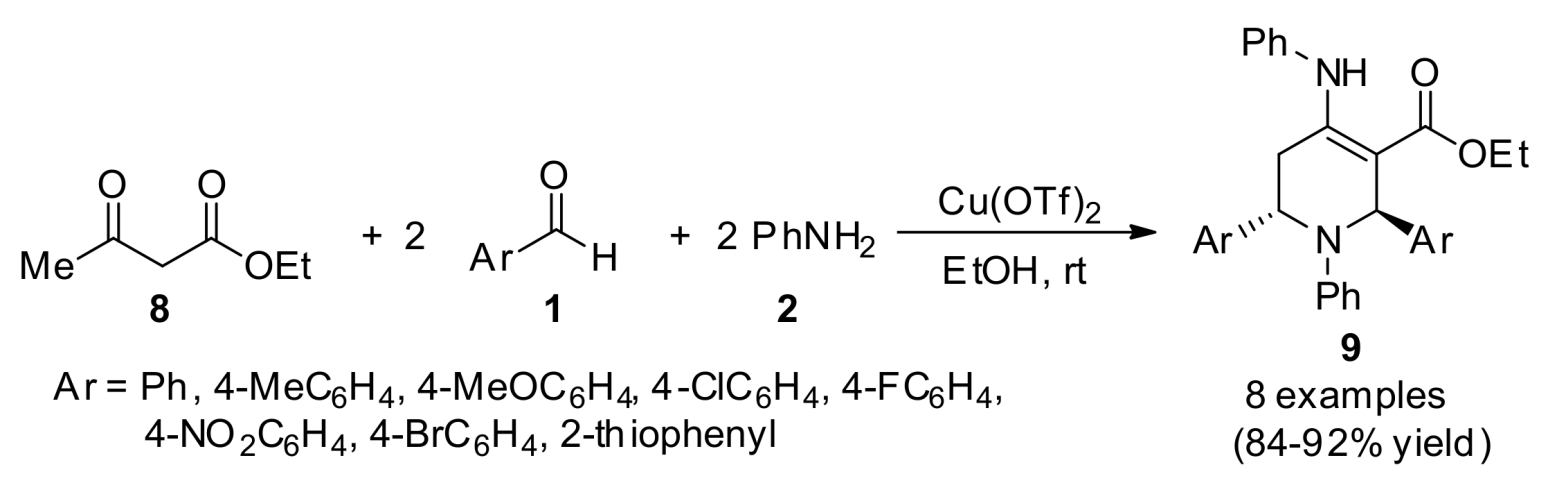
Multicomponent Cu(OTf)_2_ catalyzed synthesis of substituted tetrahydropyridines **9** for anti-inflammatory activity.

Patil et al., reported a one-pot *pseudo*-five-component synthesis of highly functionalized tetrahydropyridines **9** using Cu(OTf)_2_ as catalyst, Scheme 3. In vitro anti-inflammatory activity of the obtained compounds **9** was determined against matrix metalloproteinases (MMPs) as MMP-2 and MMP-9 by using gelatin zymography [29].

**Scheme 4:**
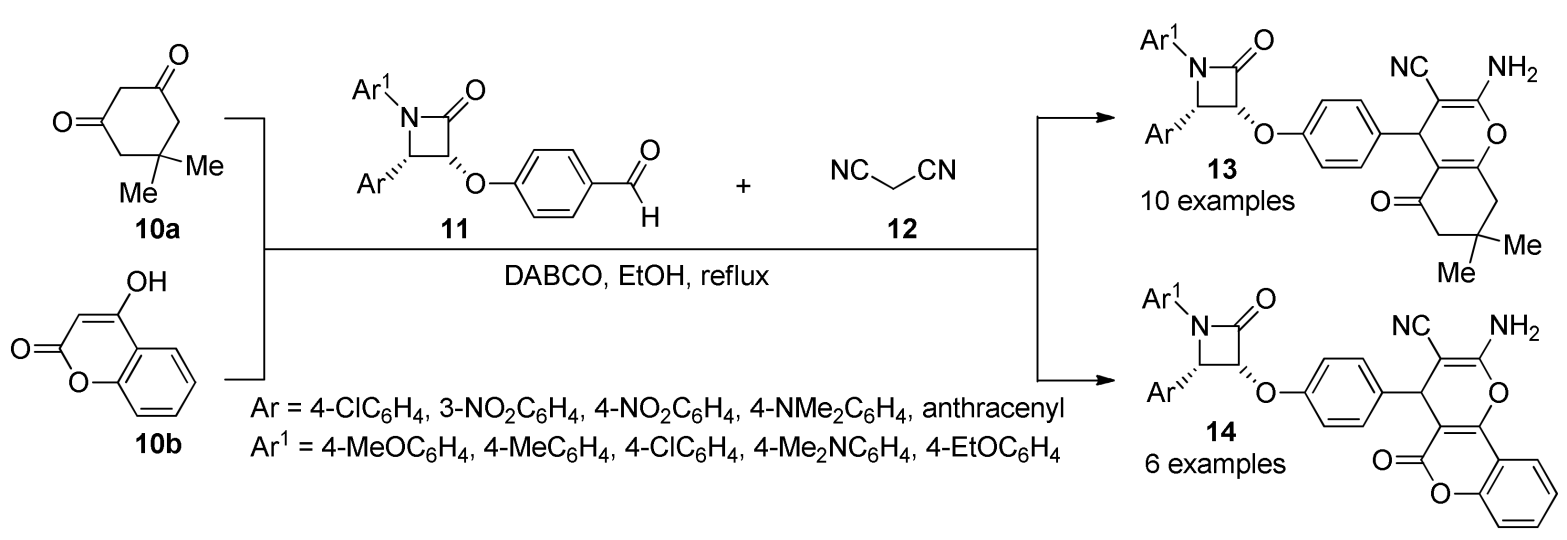
Three-component synthesis of *β*-lactam hybrids **13/14** for anti-inflammatory activity.

A highly diastereoselective synthesis (exclusively the *cis* isomer is formed), of chromeno *β*-lactam hybrids **13/14** was also achieved by an efficient one-pot three-component reaction between either 5,5-dimethylcyclohexane-1,3-dione (**10a**) or 4-hydroxycoumarin (**10b**), diverse benzaldehydes **11** and malononitrile (**12**), in the presence of DABCO under reflux conditions (Scheme 4). 

The synthesized compounds (**13** in 80–95% yield) and (**14** in 82–95% yield) were screened for anti-inflammatory activity (as well as for anticancer activity, please see Section 3.13.13. Compound **13b** (Ar = 4-ClC_6_H_4_, Ar^1^ = 4-MeC_6_H_4_) was the most active of all the chromeno *β*-lactam hybrids **13/14** tested, with a 19.8 anti-inflammatory ratio, although, it resulted less active than the reference drug dexamethasone corticosteroid used for the treatment of rheumatoid and skin inflammation [30].

### 3.3. ROCK Inhibitors

**Scheme 5:**
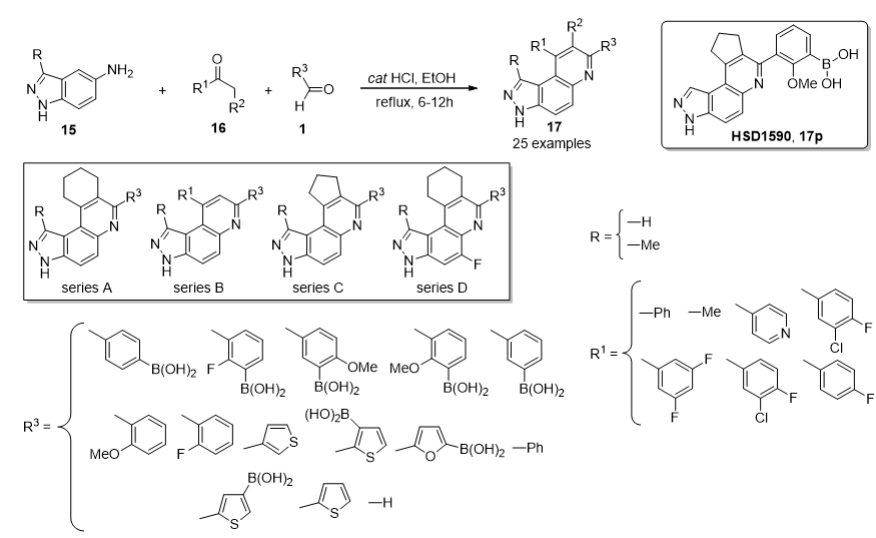
Three-component synthesis of boron containing compounds **17** for ROCK inhibition.

Rho-associated protein kinases (ROCKs) are ubiquitously expressed in most adult tissues, and are involved in modulating the cytoskeleton, protein synthesis and degradation pathways, synaptic function, and autophagy. Among the current limited number of ROCK inhibitors of clinical use, such as fasudil [31] and netarsudil [32], the synthesis and biological evaluation of a series of boronic acid-containing 3*H*-pyrazolo[4,3-*f*]quinolones **17** as potential ROCK inhibitors have recently been reported [33]. The synthetic process involved a three-component Povarov type reaction between indazol-5-amines **15**, methylene active ketones **16** and aldehydes **1** in the presence of catalytic amounts of HCl, affording the expected products **17** in 70–90% yield, as depicted in Scheme 5. After a SAR analysis of the obtained products **17**, the biological trials indicated that compound labeled as **HSD1590** (**17p**), resulted more potent than the reference drug netarsudil at binding to or inhibiting ROCK enzymatic activities. This compound exhibited single digit nanomolar binding to ROCK (Kds < 2 nM) and subnanomolar enzymatic inhibition profile (i.e., ROCK2 IC_50_ was 0.5 nM for **17p** while Netarsudil inhibited ROCK2 with IC_50_ = 11 nM under similar conditions) [33].

### 3.4. Bromodomain Inhibitors

**Scheme 6:**
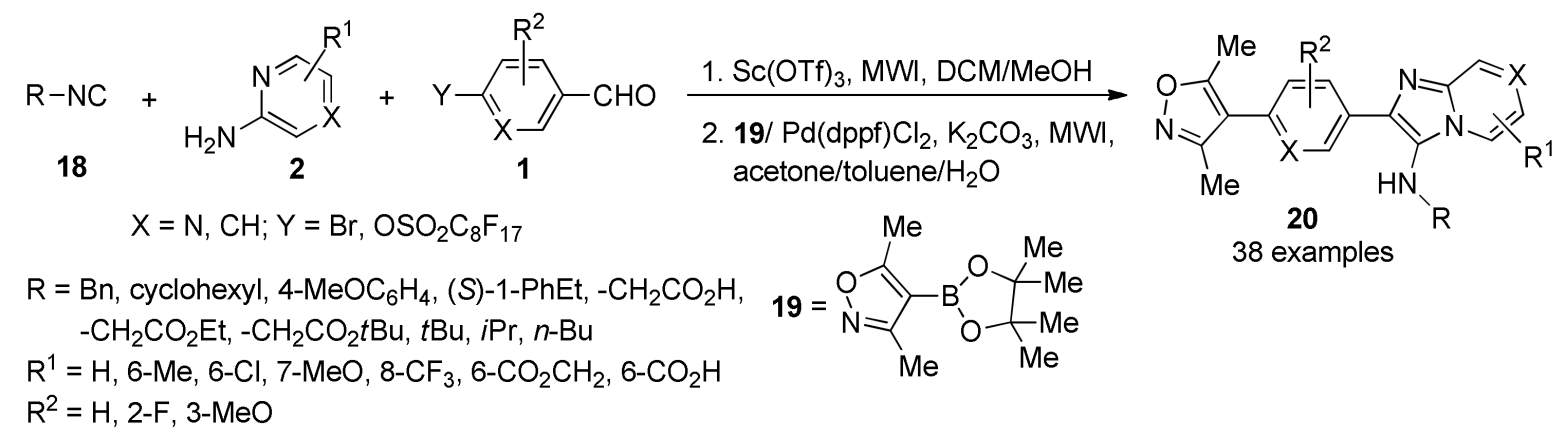
Elaboration of imidazopyridine scaffolds **20** as bromodomain inhibitors mediated by a three-component approach.

The inhibition of the bromodomain and extra-terminal (BET) domain subfamily of human bromodomains from chromatin [34], has contributed new insights into gene regulation and emerged as a promising therapeutic strategy in cancer. Structural analogy of early methyltriazolo BET inhibitors has prompted a need for structurally dissimilar ligands as bromodomain function probes. Using fluorous-tagged multicomponent reactions, a focused chemical library of bromodomain inhibitors **20** with micromolar biochemical IC_50_ values was developed around a 3,5-dimethylisoxazole biasing element. Iterative synthesis and biochemical assessment allowed optimization of novel BET bromodomain inhibitors based on an imidazo[1,2-*a*]pyrazine scaffold. The synthesis of the target molecules **20** (in 11–58% yield), involved a three-component reaction between isocyanides **18**, pyrazines/pyridines **2** and aldehydes **1** in the presence of Sc(OTf)_3_ as catalyst, followed by a Suzuki-type coupling reaction with the boronic acid derivative **19** catalyzed by Pd(dppf)Cl_2_ (Scheme 6). The lead compound **20c** (R = *t*Bu; R^1^ = R^2^ = H) binds BRD4 with a K_d_ of 550 nM and 724 nM cellular potency in BRD4-dependent lines. Additionally, compound **20c** showed potency against TAF1, a bromodomain-containing transcription factor previously unapproached by discovery chemistry [35].

### 3.5. Antifibrotic Agents

**Scheme 7:**
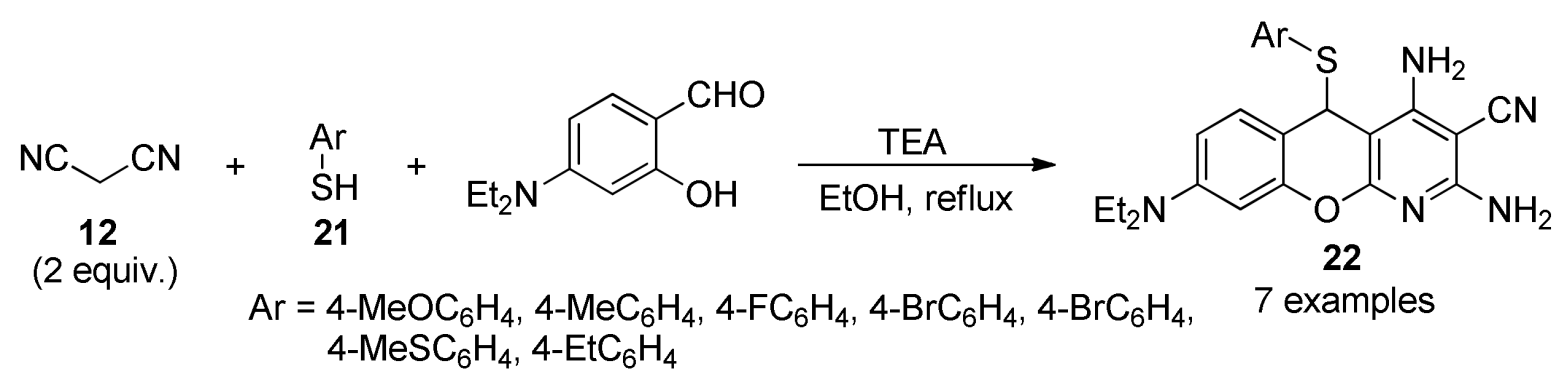
Preparation of 5-arylthio-5*H*-chromenopyridines **22** of anti-fibrotic interest using a multi-component reaction (MCR) approach.

Liver fibrosis is a critical wound healing response to chronic liver injury such as hepatitis C virus (HCV) infection. If persistent, liver fibrosis can lead to cirrhosis and hepatocellular carcinoma (HCC). The development of new therapies for preventing liver fibrosis and its progression to cancer associated with HCV infection remains a critical challenge [36]. Identification of novel anti-fibrotic compounds will provide opportunities for innovative therapeutic intervention of HCV-mediated liver fibrosis. In this sense, it was designed and synthesized a set of 5-arylthio-5*H*-chromenopyridines **22** as a new class of anti-fibrotic agents. Products **22** were synthesized in 16–45% yield through a *pseudo*-four-component reaction involving malonitrile (**12**, 2 mmol), thiophenols **21** (1 mmol) and 4-diethylaminosalicylaldehyde in the presence of triethylamine as catalyst (Scheme 7). Liver fibrosis assays demonstrated that compounds **22a** (Ar = 4-FC_6_H_4_) and **22c** (Ar = 4-BrC_6_H_4_) showed inhibitory activity towards human hepatic stellate cells (LX2) activation at 10 μM. The HCV NS3 and NS5A proteins in HCV subgenome-expressing cells were also significantly reduced in cells treated with **22a** and **22c**, suggesting the possible inhibitory role of the compounds in HCV translation/replication activities [37]. The reactivity of compounds **22** with medicinally-relevant metal compounds such as platinum and gold was also examined. The reactivity of these complexes with metals and during mass spectrometry suggested that C-S bond cleavage is relatively facile.

### 3.6. Human Toll-Like Receptor 8-Active Compounds

**Scheme 8:**
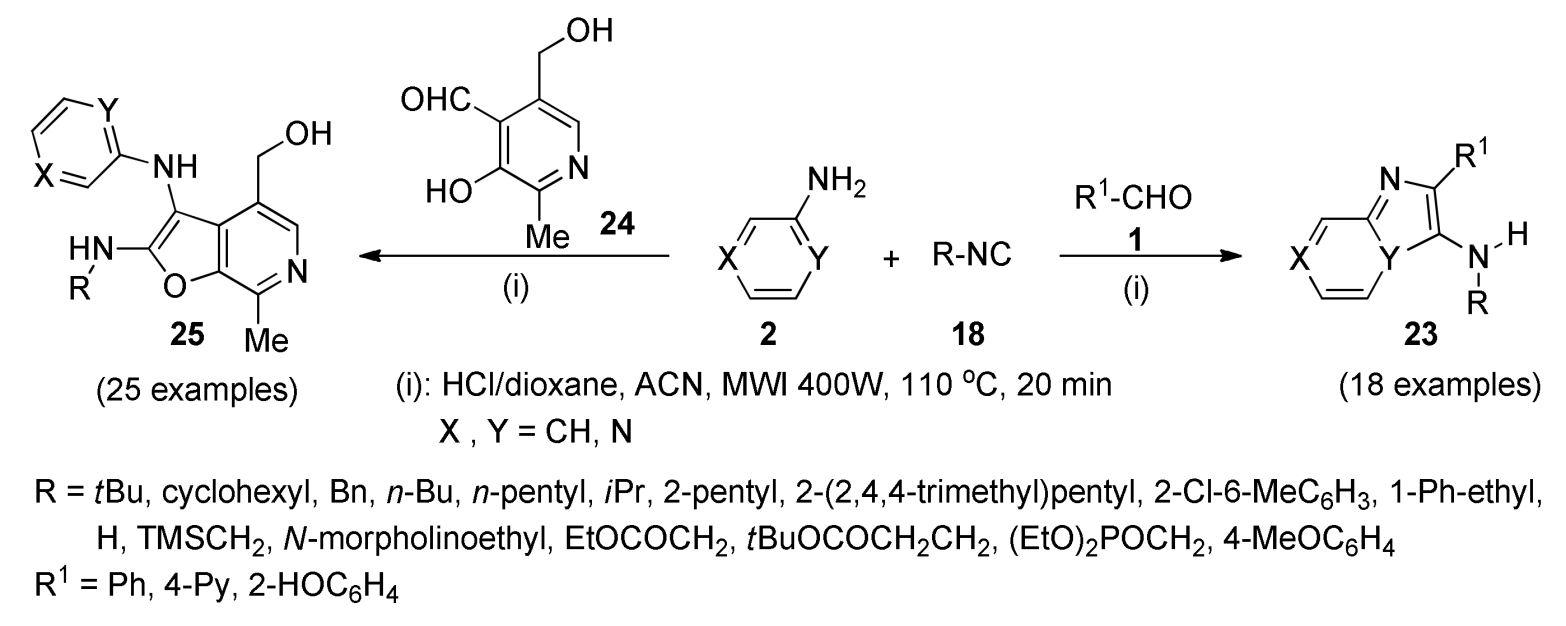
Two step one-pot three-component synthetic processes for TLR7/8 agonistic ligands **23** and **25**.

The innate immune system utilizes germline-encoded pattern recognition receptors (PRRs) to discern pathogen-associated molecular patterns (PAMPs) that are distinct to the pathogen [38]. The transmembrane PRRs include the toll-like receptors (TLRs) [39], which are expressed either on the plasma membrane or in the endolysosomal compartments [38]. At least ten functional TLRs are encoded in the human genome, each with an extracellular domain having leucine-rich repeats and a cytosolic domain called the toll/IL-1 receptor domain [40]. The discovery of TLRs has not only served to greatly accelerate the understanding of the interplay between the innate and adaptive immune systems, but is also catalyzing novel approaches to vaccine design and development. The ligands for these receptors are highly conserved microbial molecules [40,41]. For instance, it was proposed that imidazo[1,2-*a*]pyridine/pyrazines **23** (Scheme 8), could work as TLR7/8 ligands. Compounds **23**, were obtained (in 13–86% yield) via a Groebke-Blackburn-Bienaymé type multicomponent reaction [42], along with the unplanned furo[2,3-*c*]pyridine/pyrazines **25** (in 10–84% yield), when pyridoxal (**24**) was used as aldehyde (Scheme 8) [43]. Both libraries of structures **23** and **25** were subjected to screening for TLR7/8 agonistic activities, as potential vaccine adjuvants.

The biological assays showed that most of the obtained compounds **23** were inactive in NF-κB reporter gene assays specific for human TLR-3, -7, -8, and -9; however, most compounds **25** were found to specifically activate NF-κB signaling in TLR8-transfected HEK293 cells [43].

### 3.7. Neuroprotective Agents

**Scheme 9:**
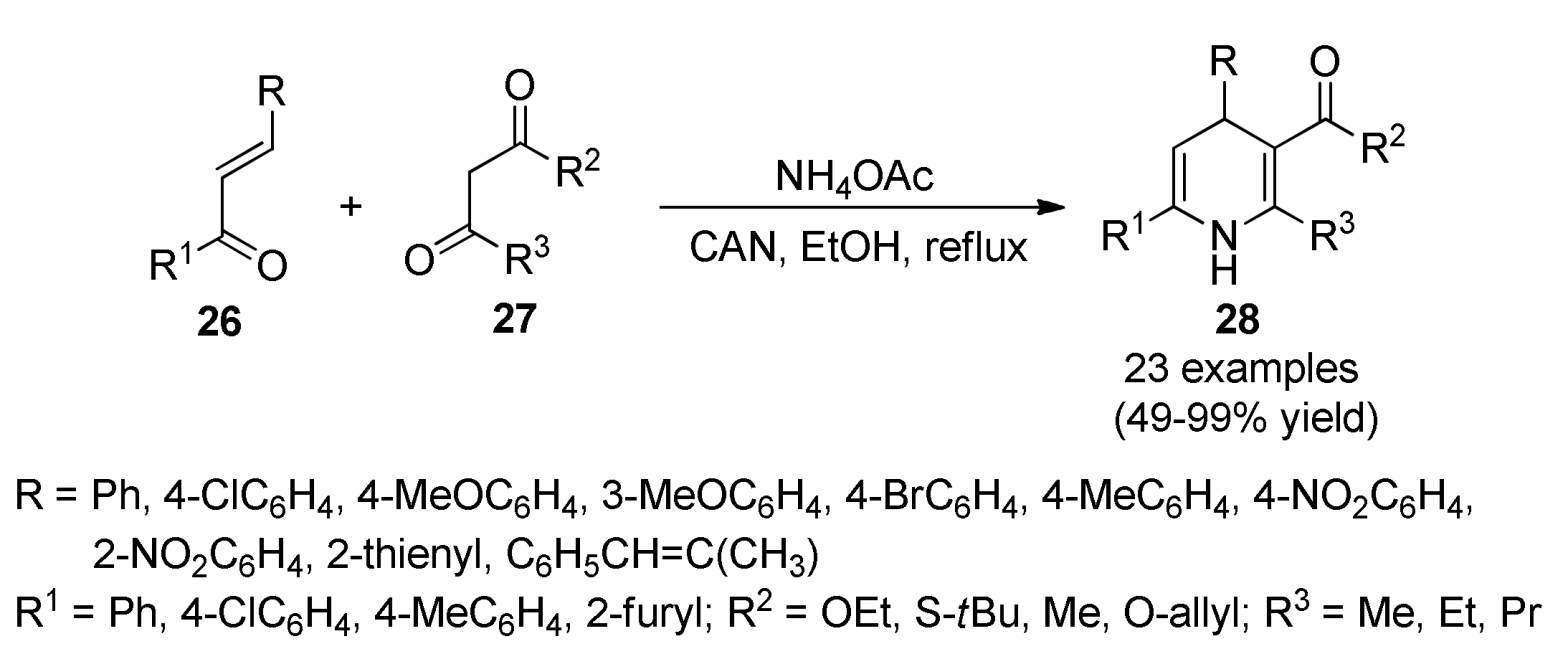
Three-component synthesis of 5-unsubstituted dihydropyridines **28** with improved Ca_v_1.3 selectivity as potential neuroprotective agents against ischemic injury.

Neurodegenerative disorders constitute a significant public health problem worldwide, and among these, cerebrovascular accidents represent one of the leading causes of death, neurological disability, and cognitive impairment [44]. During cerebral ischemia, oxygen and glucose deprivation induces a metabolic cascade that leads to neuronal death. One of the most significant consequences of these changes is the dysregulation of Ca^2+^ homeostasis, leading to brain damage. In this context, a set of C_5_-unsubstituted-C_6_-aryl-1,4-dihydropyridines **28** were prepared by a CAN-catalyzed three-component reaction from chalcones **26**, *β*-dicarbonyl compounds **27**, and ammonium acetate in refluxing EtOH (Scheme 9). Compounds **28** were able to block Ca^2+^ entry after a depolarizing stimulus and showed an improved Ca_v_1.3/Ca_v_1.2 selectivity in comparison with nifedipine. Furthermore, they were able to protect neuroblastoma cells against Ca^2+^ overload and oxidative stress models. It is highlighted that the selectivity ratio of **28** makes them highly interesting for the treatment of neurological disorders where Ca^2+^ dyshomeostasis and high levels of oxidative stress was demonstrated. Furthermore, their low potency toward the cardiovascular channel subtype makes them safer by reducing their probable side effects, in comparison to classical 1,4-dihydropyridines. Some of the obtained compounds **28** afforded good protective profile in a postincubation model that simulates the real clinical situation of ictus patients, offering a therapeutic window of opportunity of great interest for patient recovery after a brain ischemic episode. Good activities were also found in acute ischemia/reperfusion models of oxygen and glucose deprivation [45].

### 3.8. Acetylcholinesterase Inhibitors

**Scheme 10:**
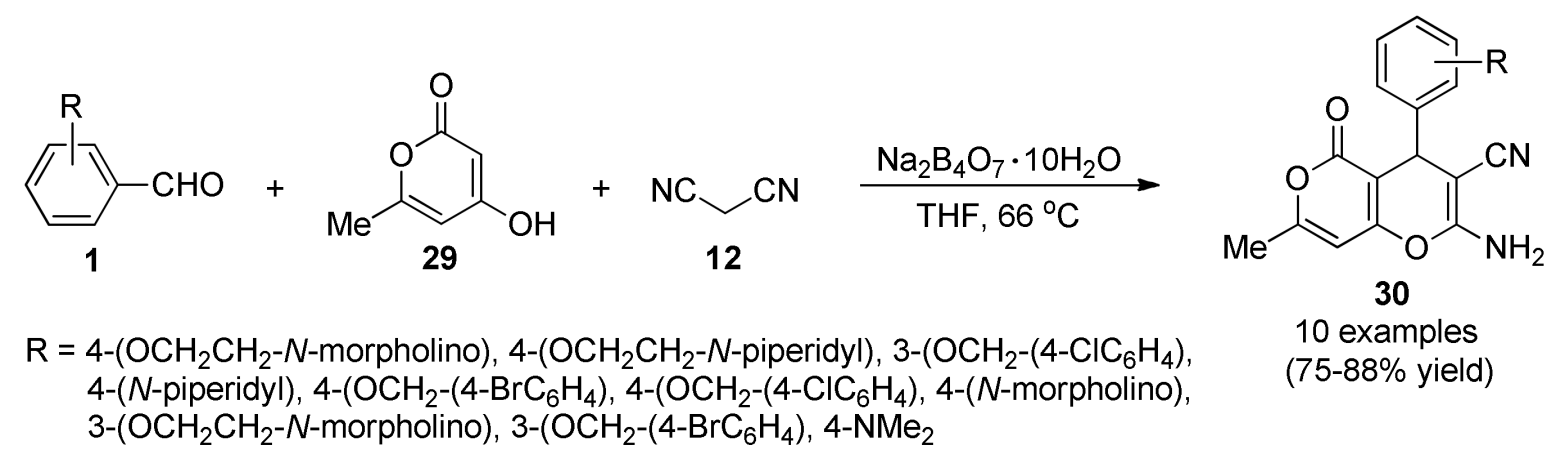
Three-component derivatization of compounds **30** for evaluation of their activity against AChE.

It is suggested that compounds that can inhibit cholinesterase enzyme may be considered as anti-Alzheimer, anti-Parkinson, and anti-autism drugs [46]. Due to the fact donepezil and other FDA approved drugs used for treatment of above diseases present some side effects [47], many efforts trying to find, develop, and explore more potent and permissive anti-Alzheimer drugs without any harmful side effects have been made so far. Among them, a borax-catalyzed protocol for the synthesis of a set of 4-aryl-substituted-4*H*-pyran derivatives fused to *α*-pyrone ring **30** in a one-pot procedure, as potential acetylcholinesterase inhibitors (AChEIs), was described. In this approach, products **30** were obtained in good to excellent yields, from a three-component reaction between aryl aldehydes **1**, 4-hydroxy-6-methyl-2*H*-pyran-2-one (**29**) and malononitrile (**12**), in the presence of borax as catalyst and THF as solvent (Scheme 10). Subsequently, compounds **30** were evaluated in silico against acetylcholinesterase enzyme (AChE) and their Absorption, Distribution, Metabolism, Excretion and Toxicity (ADMETox) properties were also studied, to make the results more reliable and introduce them as remarkable potential candidates for inhibition of AChE, in the treatment of Alzheimer’s, Parkinson’s and autism diseases. Among the evaluated products, compound **30f** (R = *p*-OCH_2_C_6_H_4_Br) showed the best activity against AChE [48].

### 3.9. Anti-HIV Activity

**Scheme 11:**
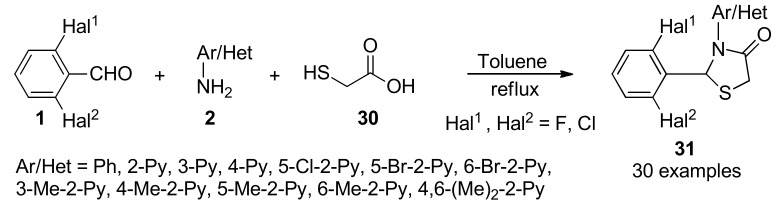
Three-component synthesis of 2,3-diaryl-1,3-thiazolidin-4-ones **31** for structure-activity relationships, molecular modeling and studies as potent anti-HIV agents.

Reverse transcriptase (RT) is a key enzyme which plays an essential and multifunctional role in the replication of the human immunodeficiency virus (HIV) and thus represents an attractive target for the development of new drugs useful in AIDS therapy [49,50]. In view of the increasing incidence of resistance to current drug regimens and the frequency of adverse events, the development of novel, selective, potent, safe, inexpensive antiviral agents, that are also effective against mutant HIV strains, remains a high priority for medical research. In that direction, the design, synthesis, and the structure-activity relationship studies of a series of 2,3-diaryl-1,3-thiazolidin-4-ones **31** was performed. The synthesis of products **31** (in 8–87% yield), involved a three-component procedure reacting a suiTable 2,6-dihalo-substituted benzaldehydes **1** with an equimolar amount of a (hetero)aromatic amines **2** in the presence of an excess of mercaptoacetic acid **30** in refluxing toluene (Scheme 11). Some derivatives **31** proved to be highly effective in inhibiting HIV-1 replication at nanomolar concentrations with minimal cytotoxicity, thereby acting as nonnucleoside HIV-1 RT inhibitors (NNRTIs). Computational studies were used to delineate the ligand-RT interactions and to probe the binding of the ligands **31** to HIV-1 RT [51].

### 3.10. Antimicrobial Activity

**Scheme 12:**
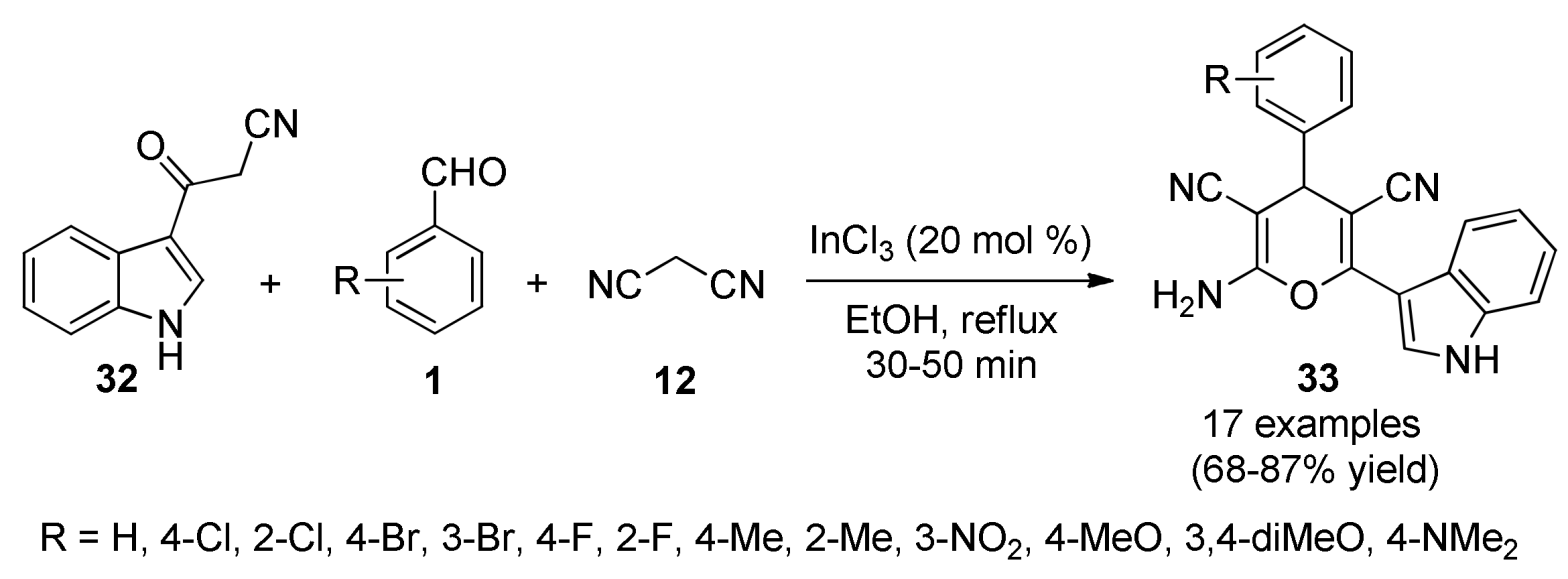
InCl_3_-catalyzed three-component synthesis of 3-pyranyl indole derivatives **33** for assays against Gram-positive and Gram-negative bacteria.

An alarming increment in pathogenic resistance to existing drugs is a serious problem with antimicrobial therapy, indicating the necessity of continuing with the research for new classes and more effective of antimicrobials [52], possibly acting through mechanisms different from those of existing drugs [53]. In this context, it is very essential to successfully develop novel and efficient antimicrobial agents with clinically unexploited mode of action. As a contribution to this topic, Lakshmi et al., reported an InCl_3_-catalyzed three-component reaction for the synthesis of 3-pyranyl indole derivatives **33** as antimicrobial agents. The process was mediated by a tandem Knoevenagel–Michael reaction of 3-cyanoacetyl indole (**32**) diverse aromatic aldehydes **1** and malononitrile (**12**) catalyzed by InCl_3_ in refluxing ethanol (Scheme 12). The antibacterial activity was screened by paper disc diffusion method against two Gram-positive bacteria (*Staphylococcus aureus*, *Bacillus cereus*), and two Gram-negative bacteria (*Escherichia coli*, *Klebsiella pneumoniae*) by using ciprofloxacin as reference compound. As shown in Scheme 12, compound **33e** (R = 2-Cl) was found to exhibit the more potent in vitro antibacterial activity, with MIC values of 12.4, 16.4, 16.5, and 16.1 μM against *S. aureus*, *B. cereus*, *E. coli*, and *K. pneumoniae*, respectively. In addition, compounds **33a** (R = H), **33d** (R = 4-Cl), **33h** (R = 4-F), and **33m** (R = 4-OMe) exhibited significant antibacterial activity when compared to the standard drug ciprofloxacin [54].

**Scheme 13:**
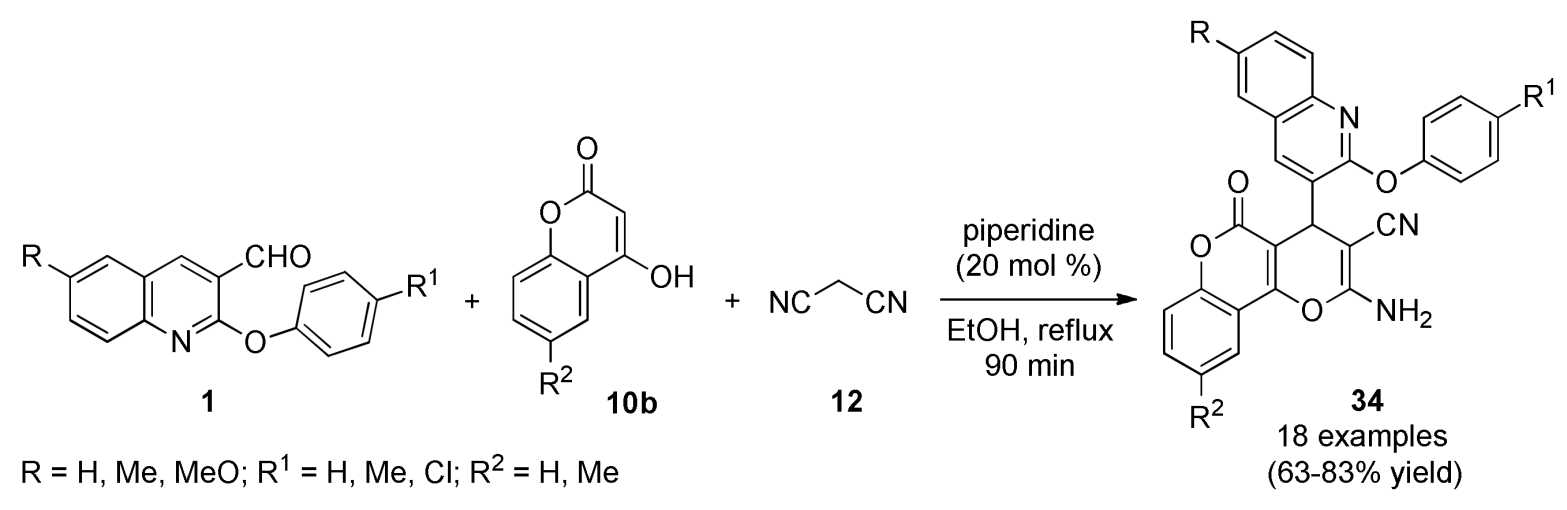
Piperidine-catalyzed three-component synthesis of pyrano[3,2-*c*]chromene derivatives **34** as potential Gram-positive and Gram-negative bactericidal agents.

A new class of pyrano[3,2-*c*]chromene derivatives **34** incorporating a validated molecular target was synthesized through a one-pot multicomponent cyclocondensation reaction between *β*-aryloxyquinoline-3-carbaldehydes **1**, 4-hydroxycoumarins **10b** and malononitrile (**12**) in ethanol containing a catalytic amount of piperidine, Scheme 13. Antibacterial activity was screened against three Gram-positive bacteria (*Bacillus subtilis* MTCC 441, *Clostridium tetani*, *Streptococcus pneumoniae*) and three Gram-negative bacteria (*Escherichia coli*, *Salmonella typhi*, *Vibrio cholerae*) by using ampicillin as a standard antibacterial drug. Remarkably, compounds **34f** (R = Me, R^1^ = Cl, R^2^ = H), **34l** (R = H, R^1^ = Cl, R^2^ = Me) and **34q** (R = MeO, R^1^ = Me, R^2^ = Me) exhibited excellent in vitro antibacterial activity. The majority of compounds **34** were found to possess higher potency as compared to standard bactericidal ampicillin against Gram-positive bacteria *B. subtilis* [55].

**Scheme 14:**
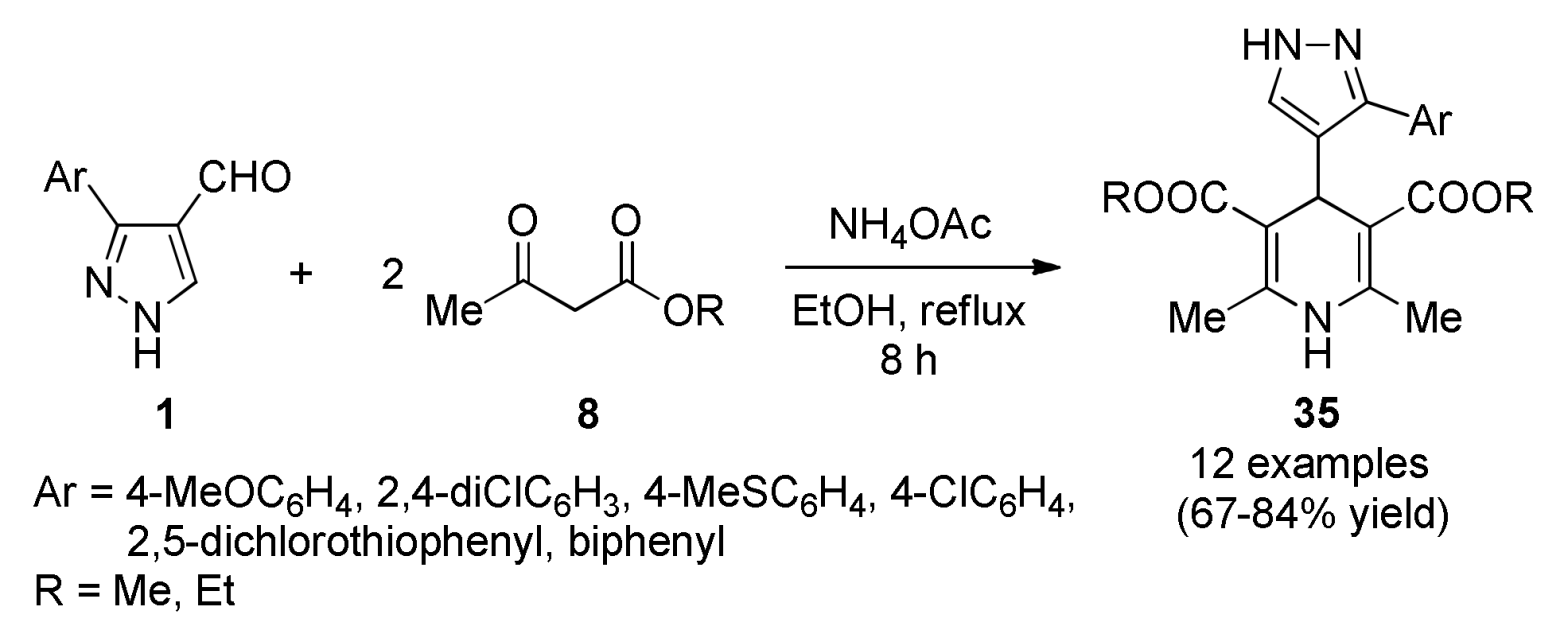
Synthesis of 1,4-dihydropyridine derivatives **35** through a Hantzsch multicomponent reaction for antibacterial assays.

Vijesh et al., reported the synthesis of 1,4-dihydropyridine derivatives (1,4-DHPs) **35** containing substituted pyrazole moiety as potent antimicrobial, as well as, antioxidant agents [56]. The synthetic process involved a *pseudo*-four-component Hantzsch reaction between 3-aryl-1*H*-pyrazole-4-carbaldehydes **1**, 1,3-dicarbonyl compounds **8** (ethyl acetoacetate and methyl acetoacetate) and ammonium acetate in ethanol under reflux conditions (Scheme 14). Antibacterial activity was screened against one Gram-positive bacteria (*Staphylococcus aureus*) and two Gram-negative bacteria (*Escherichia coli*, *Pseudomonas aeruginosa*) by using streptomycin as standard drug. The results indicated that among the tested compounds, **35c** (R = Et, Ar = 4-MeSC_6_H_4_) and **35f** (R = Et, Ar = 4-ClC_6_H_4_) showed excellent activity against all the tested microbial strains *E. coli*, *S. aureus* and *P. aeruginosa* at concentrations of 1, 0.5 and 0.25 mg/mL compared to the standard drug streptomycin.

**Scheme 15:**
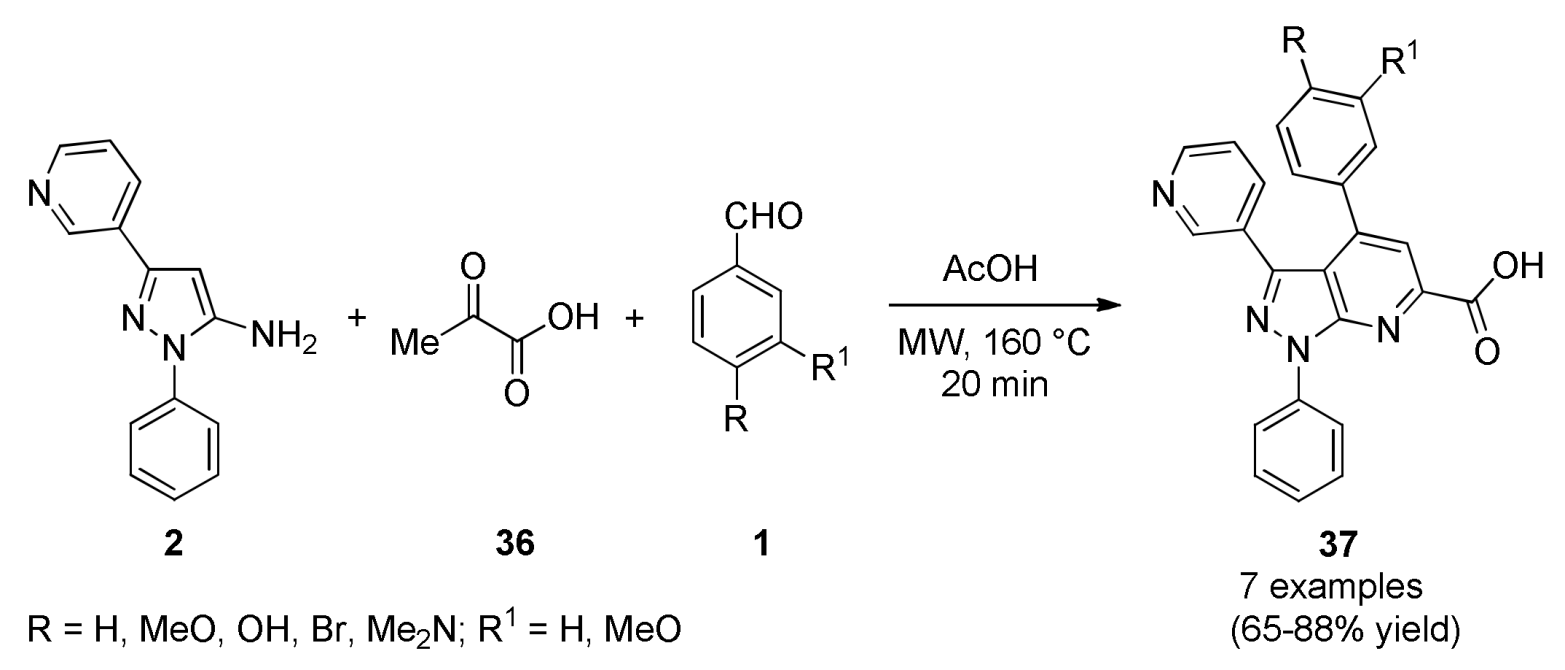
Microwave-assisted three-component synthesis of pyrazolo[3,4-*b*]pyridine derivatives **37** of antibacterial interest.

El-borai et al., reported an efficient protocol for the microwave-assisted synthesis of pyrazolo[3,4-*b*]pyridine derivatives **37** in good to excellent yields [57]. In this approach, products **37** were obtained from a three-component reaction between 5-amino-1-phenyl-3-(pyridin-3-yl)-1*H*-pyrazole type **2**, pyruvic acid (**36**) and diverse aromatic aldehydes **1** using acetic acid as solvent under MWI at 160 °C for 20 min (Scheme 15). Subsequently, products **37** were screened for antibacterial, as well as, antifungal and antitumor activity. In particular, antibacterial activity was screened against one Gram-positive bacteria (*Bacillus cereus*) and three Gram-negative bacteria (*Escherichia coli*, *Enterobacter cloaca* and *Serratia marcescens*). Among the tested compounds, only **37a** (R = MeO, R^1^ = H), **37d** (R = OH, R^1^ = H), **37e** (R = Br, R^1^ = H) and **37f** (R = R^1^ = MeO) exhibited antibacterial activity of high order against all strains of the bacteria yeast tested.

**Scheme 16:**
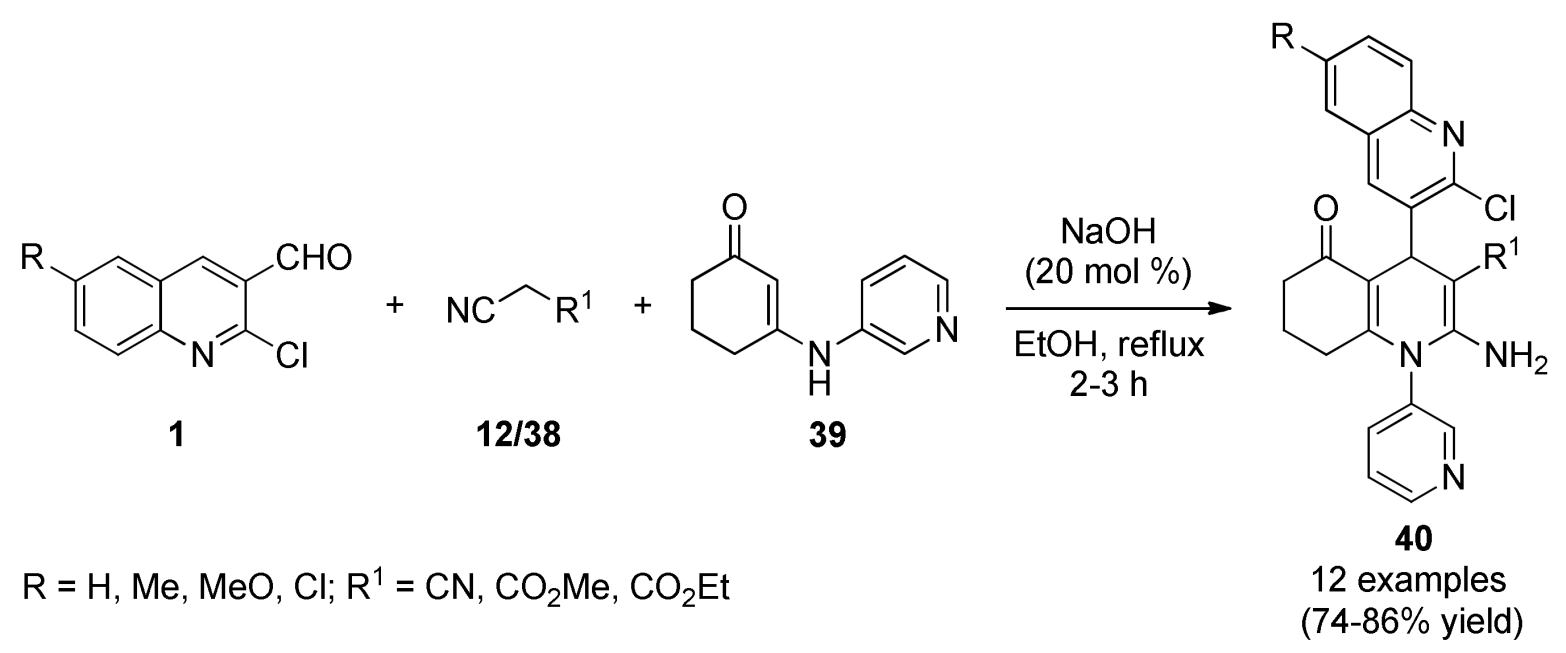
Three-component synthesis of quinoline-pyridine hybrids **40** as potential antibacterial agents.

Shah et al., reported a small library of quinoline-pyridine hybrids **40** through a three-component reaction between a series of 2-chloro-3-formylquinolines **1**, active methylene compounds **12/38** and 3-(pyridine-3-ylamino)cyclohex-2-enone (**39**) in the presence of catalytic amount of sodium hydroxide in ethanol under refluxing conditions (Scheme 16) [58]. This protocol afforded a time-efficient synthesis of the structurally diverse quinoline-pyridine hybrids **40** in good yields for antimicrobial, as well as, for antifungal and antitubercular screening. These products were screened for their antibacterial activity against three Gram-positive bacteria (*Bacillus subtilis*, *Clostridium tetani*, *Streptococcus pneumoniae*), and three Gram-negative bacteria (*Escherichia coli*, *Salmonella typhi*, *Vibrio cholerae*). Among the products tested only **40b** (R = Me, R^1^ = CN) and **40i** (R = H, R^1^ = CO_2_Me) showed better inhibitory effects for *E. coli*, and **40g** (R = MeO, R^1^ = CO_2_Et) showed better results for *S. typhi* compared to standard drugs such as ampicillin, chloramphenicol, ciprofloxacin and norfloxacin.

**Scheme 17:**
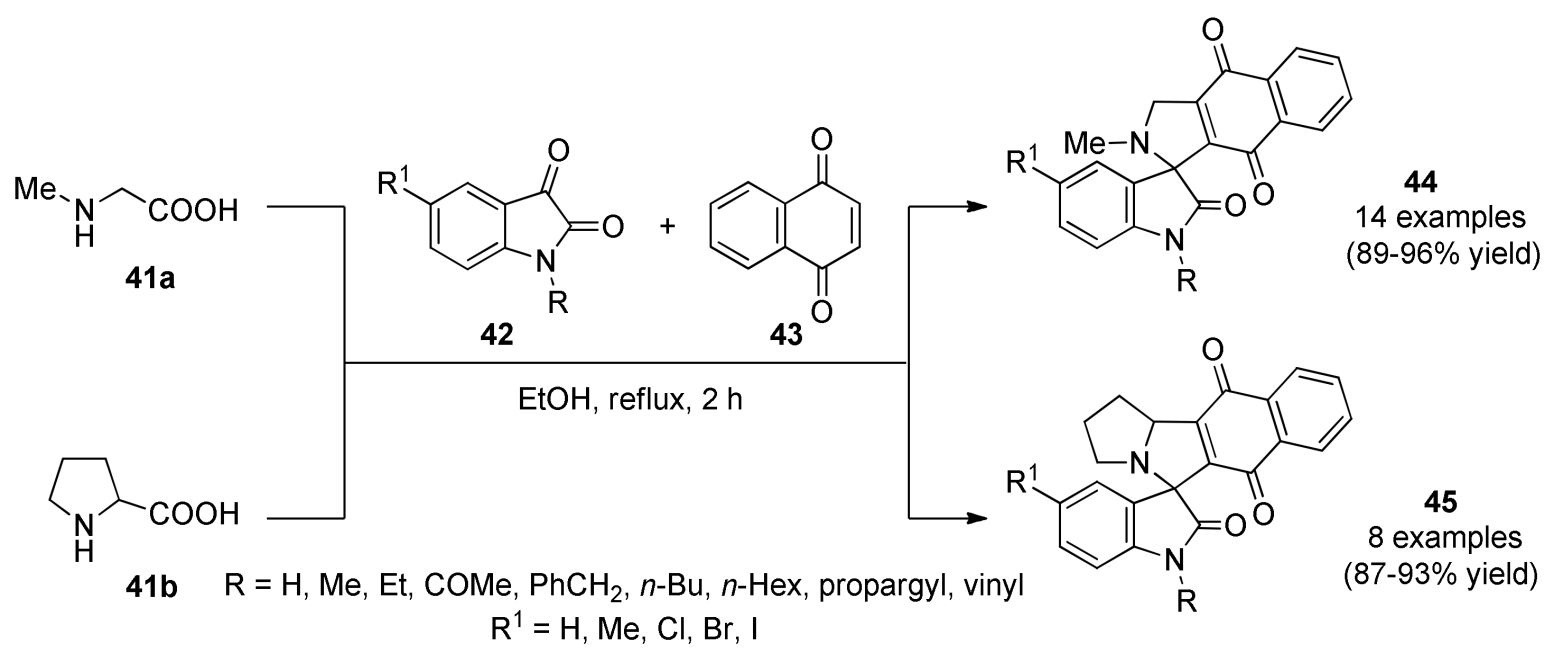
Three-component synthesis of diverse spirooxindole derivatives **44** and **45** for antibacterial studies.

Bhaskar et al., reported the synthesis of a series of spirooxindole derivatives **44** and **45** through a three-component 1,3-dipolar cycloaddition of an azomethine ylide generated in situ from sarcosine (**41a**) or L-proline (**41b**) and isatin **42** with the dipolarophile 1,4-naphthoquinone **43** followed by spontaneous air oxidation in atmospheric reflux conditions (Scheme 17) [59]. Subsequently, products **44** and **45** were screened for antibacterial (and for antifungal), activity against four Gram-positive bacteria (*Staphylococcus aureus*, methicillin-resistant *Staphylococcus aureus*, *Enterobacter aerogenes*, *Micrococcus luteus*), and four Gram-negative bacteria (*Proteus vulgaris*, *Klebsiella pneumoniae*, *Salmonella typhimurium*, and *Salmonella paratyphi-B*). Remarkably, compound **44n** (R = COMe, R^1^ = Me) was found to be more than 1.6 times active against methicillin-resistant *S. aureus* bacteria than streptomycin and ciprofloxacin. Also more than 6.4 times active against *M. luteus* and *S. typhimurium* bacteria than ciprofloxacin.

**Scheme 18:**
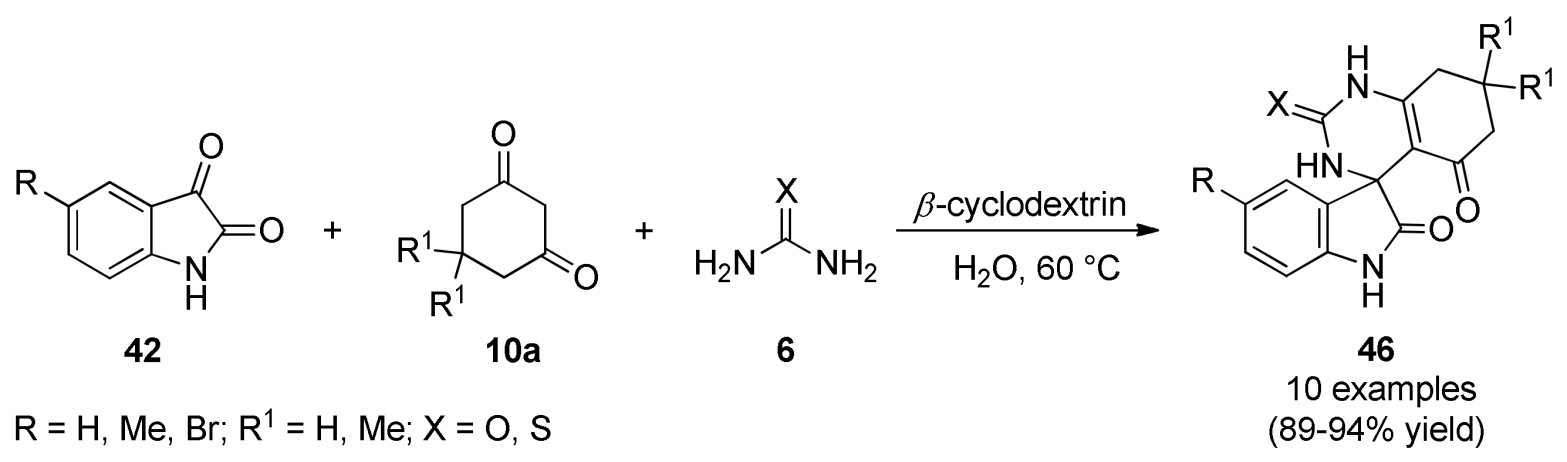
*β*-Cyclodextrin-catalyzed three-component synthesis of spiro-oxindole derivatives **46** for their evaluation as antibacterial agents.

In the course of a synthetic study toward other spiro-oxindole, Singh et al., reported an eco-friendly strategy for the synthesis of spiro-oxindole derivatives **46** in good yields and excellent stereoselectivities by a *β*-cyclodextrin-catalyzed one-pot multicomponent reaction from isatins **42**, cyclic 1,3-diketones **10a** and (thio)urea **6** in water under mild reaction conditions (Scheme 18) [60]. In this approach *β*-cyclodextrin not only formed an inclusion complex with isatin, but also was involved in intermolecular hydrogen bonding with the (thio)urea to promote the reaction. These products were screened for their antibacterial activity against one Gram-positive bacterium (*Staphylococcus aureus*), and one Gram-negative bacterium (*Escherichia coli*). Notably, compounds **46c** (R = H, R^1^ = Me, X = S), **46d** (R = R^1^ = H, X = S), **46g** (R = Br, R^1^ = Me, X = O), **46i** (R = Br, R^1^ = Me, X = S) and **46j** (R = Br, R^1^ = H, X = S) showed comparable antibacterial activity to the standard drug streptomycin. These results revealed that the presence of bromo-substituent and sulphur moiety in the synthesized compounds induced high potency, whereas the presence of methyl group decreased the effectiveness of the compounds.

**Scheme 19:**
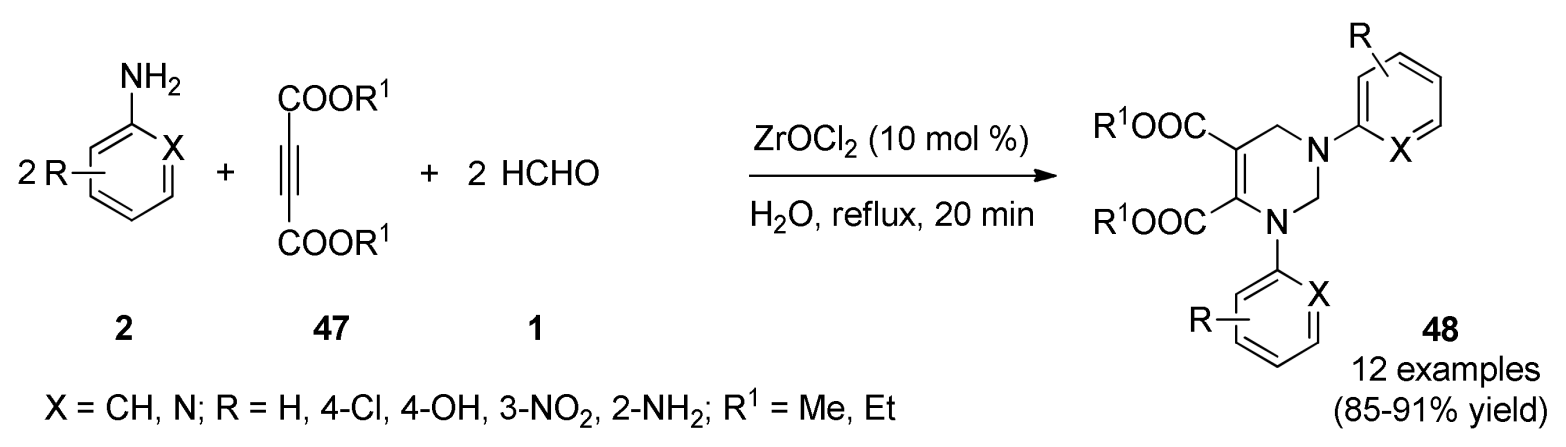
ZrOCl_2_-Catalyzed multicomponent synthesis of 1,3,4,5-tetrasubstituted 1,2,3,6-tetrahydropyrimidines **48** for antibacterial assays.

Contemporaneously, Darandale et al., proposed a green, practical and facile strategy for the synthesis of 1,2,3,6-tetrahydropyrimidine analogues as potent antimicrobial but also as antifungal agents [61]. Thus, the ZrOCl_2_-catalyzed pseudo-five-component reaction of substituted amines type **2**, dialkyl acetylenedicarboxylates **47**, and formaldehyde (**1**) in refluxing water furnished the target compounds **48** in good to excellent yields (Scheme 19). These compounds were screened for their antibacterial activity against two Gram-positive bacteria (*Staphylococcus aureus*, *Bacillus subtilis*), and one Gram-negative bacterium (*Escherichia coli*) by using ciprofloxacin and ampicillin as standard drugs. The results indicated that compounds **48a** (R = H, X = C, R^1^ = Et), **48b** (R = 4-Cl, X = C, R^1^ = Et), and **48e** (R = H, X = N, R^1^ = Et), all having the diethyl but-2-ynedioate functionality, were found to be most active and potent against the tested bacterial strains. They had MIC values (15–60 µM) compatible with standard drugs, except for bacterium *S. aureus* which showed MIC values between 60 and 100 µM. Lastly, to develop potent antibacterial agent, diethyl but-2-ynedioate was better choice than dimethyl but-2-ynedioate.

**Scheme 20:**
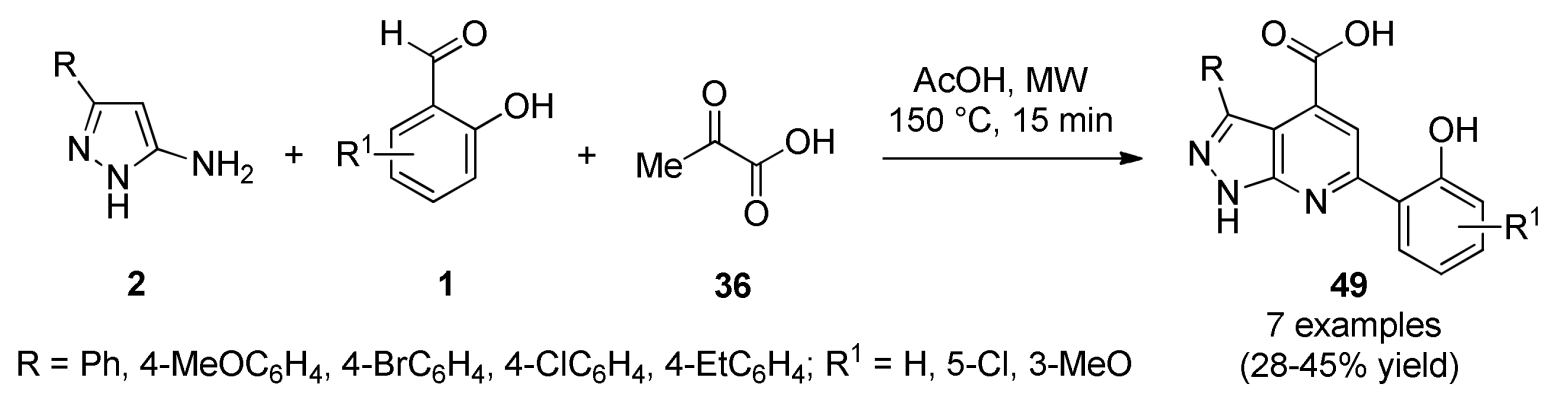
Microwave-assisted three-component synthesis of 3,6-diarylpyrazolo[3,4-*b*]pyridine-4-carboxylic acids **49** of antibacterial interest.

Murlykina, et al., reported the synthesis of 3,6-diarylpyrazolo[3,4-*b*]pyridine-4-carboxylic acids **49** through a MW-assisted three-component reaction between 5-aminopyrazoles type **2**, salicylic aldehydes **1** and pyruvic acid (**36**) in acetic acid acting both as solvent and catalyst (Scheme 20) [62]. These compounds were screened for their antibacterial activity against two Gram-positive bacteria (*Bacillus subtilis*, *Staphylococcus aureus*), and two Gram-negative bacteria (*Escherichia coli*, *Pseudomonas aeruginosa*). It was found that Gram-negative bacteria (*E. coli* and *P. aeruginosa*) showed resistance to all tested compounds in the concentration range of 15–250 µM. Albeit, strains of Gram-positive bacteria (*B. subtilis* and *S. aureus*) were found more sensitive, bacteriostatic activity was fixed only in the highest concentration 250 µM during the first day of the experiment. Thus, the tested compounds displayed lower action in comparison to nitroxoline being the reference substance.

**Scheme 21:**
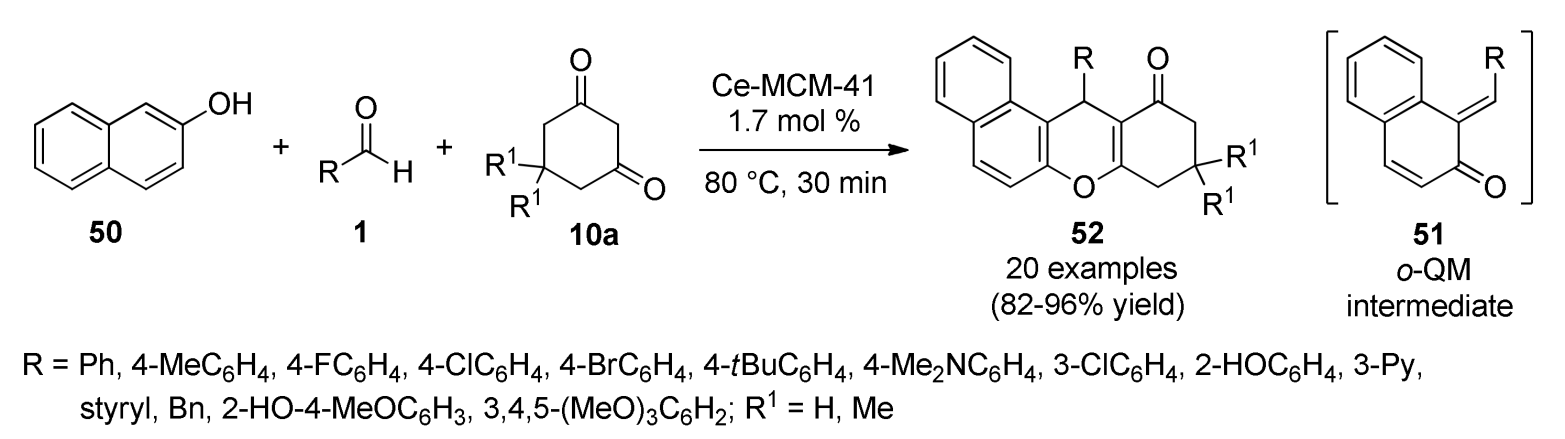
Ce-MCM-41-Catalyzed three-component synthesis of benzo[*a*]xanthenone derivatives **52**, for antibacterial screening, under solvent-free reaction conditions.

Complementarily, Trivedi’s group introduced the *ortho*-quinonemethide **51** (*o*-QM) as powerful intermediate for the generation of complex heterocyclic structures including the benzo[*a*]xanthenone skeleton [63]. The Ce-MCM-41-catalyzed three-component reaction between 2-naphthol (**50**), substituted aldehydes **1** and 1,3-diketones **10a** (5,5-dimethyl-1,3-cyclohexanedione; 1,3-cyclohexanedione; indane-1,3-dione and acetyl acetone) provided a general route to synthesize the target compounds **52** in good yields under solvent-free conditions (Scheme 21). This eco-friendly protocol offered several advantages including a green and cost-effective procedure, shorter reaction times, simpler work-up, recovery and reusability of the solid acid heterogeneous catalyst. Compounds **52** were screened for their antibacterial activity against five Gram-positive bacteria (*Bacillus subtilis*, *Micrococcus luteus*, *Bacillus circulans*, *Streptococcus mutans*, *Lysinibacillus sp*.) and four Gram-negative bacteria (*Escherichia coli*, *Klebsiella pneumoniae*, *Salmonella paratyphi*, *Pseudomonas putida*) using streptomycin as standard drug. It was noticed that the tested compounds **52f** (R^1^ = Me, R = *i*BuC_6_H_4_), **52k** (R^1^ = Me, R = 5-Br-3-Py), **52n** (R^1^ = Me, R = 2-HO-4-MeOC_6_H_3_), **52o** (R^1^ = Me, R = 4-BnO-3-MeOC_6_H_3_) and **52p** (R^1^ = Me, R = 3,4,5-(MeO)_3_C_6_H_2_) showed promising antibacterial activity against either Gram-positive or Gram-negative bacterial strains.

**Scheme 22:**
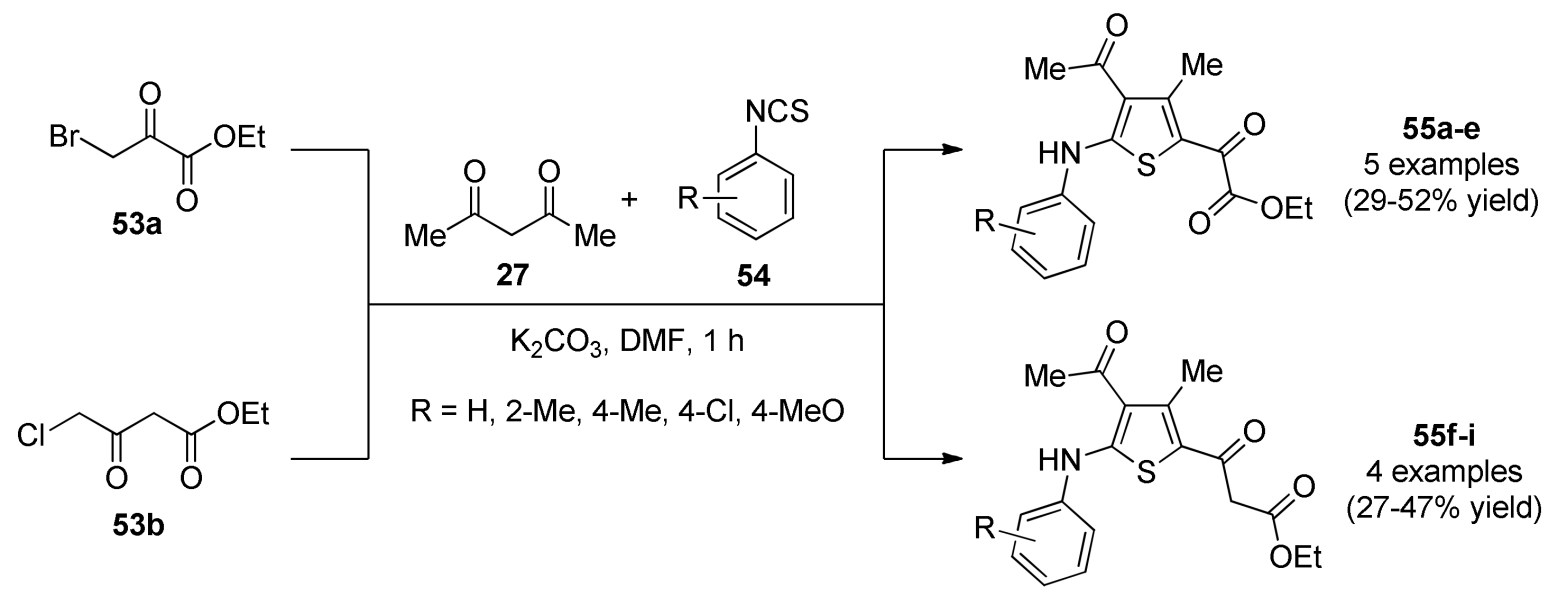
One-pot three-component synthesis of fully substituted thiophene derivatives **55** as potential antibacterial agents.

Sable, et al., reported the synthesis of fully substituted thiophene derivatives **55** through a one-pot three-component reaction of 2-bromo/chloromethyl derivatives **53**, acetyl acetone (**27**) and phenyl isothiocynates **54** under mild reaction conditions (Scheme 22) [64]. Synthesized compounds were tested for antibacterial activity against two Gram-positive bacteria (*Bacillus subtilis*, *Staphylococcus aureus*), and two Gram-negative bacteria (*Pseudomonas aeruginosa, Escherichia coli*) using azithromycin as the reference standard drug. Compounds **55** showed excellent to good activity against Gram-negative bacteria (*P. aeruginosa*, *E. coli*) having MIC values between 0.3 and 8.5 µM, and lower activity against Gram-positive bacteria (*B. subtilis, S. aureus*) having MIC values between 0.1 and 9.5 µM. The structure–activity relationship study (SAR) indicated that a change in the substituent might also affect the antibacterial activity. For example, compounds having R = H/Cl appeared to have more potential against Gram-positive bacteria (*S. aureus*) and Gram-negative bacteria (*P. aeruginosa*, *E. coli*). In addition, compounds having R = CH_3_/OCH_3_ were found to be more active against Gram-positive (*B. subtilis*) and Gram-negative bacteria (*P. aeruginosa*, *E. coli*).

**Scheme 23:**
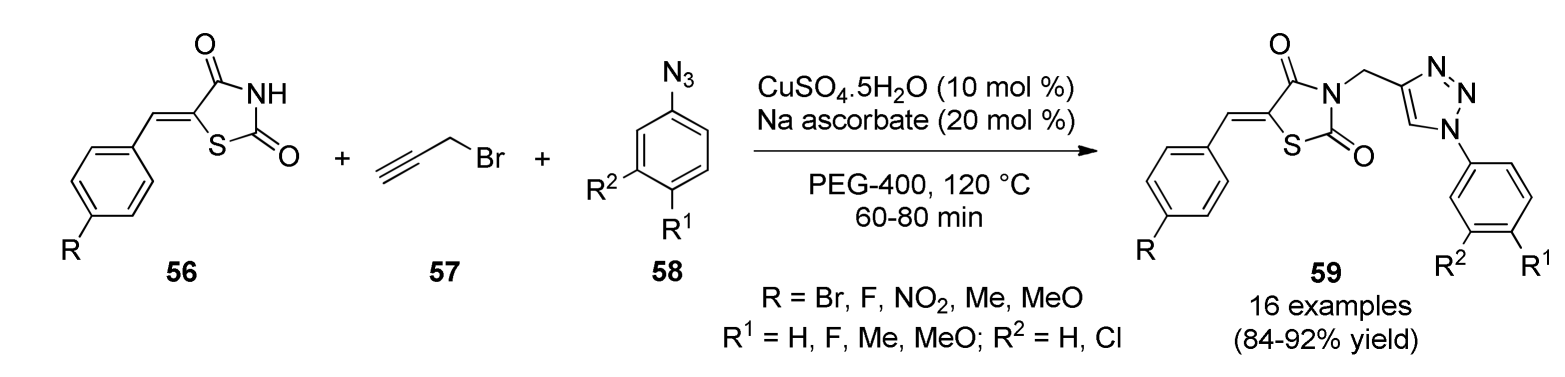
Multicomponent process for the synthesis of thiazolidinedione–triazole hybrids **59** of antibacterial interest.

In 2015, Sindhu et al., reported the three-component synthesis of thiazolidinedione–triazole hybrids **59** in high yields after short reaction times [65]. This copper(I)-catalyzed 1,3-dipolar cycloaddition reaction of 5-(arylidene)thiazolidine-2,4-diones **56**, propargyl bromide (**57**) and substituted aryl azides **58** was performed in PEG-400 as green media in the presence of CuSO_4_.5H_2_O and sodium ascorbate as catalytic system (Scheme 23). The obtained compounds were screened for their antibacterial activity against two Gram-positive bacteria (*Staphylococcus aureus*, *Bacillus subtilis*) and two Gram-negative bacteria (*Escherichia coli*, *Pseudomonas aeruginosa*) using ciprofloxacin as standard drug. The minimum inhibitory concentration (MIC) of compounds ranged between 32 and 256 µM against Gram-positive bacteria. Notably, compounds **59f** (R = Br, R^1^ = Me, R^2^ = H), **59g** (R = Br, R^1^ = H, R^2^ = Cl), **59i** (R = NO_2_, R^1^ = F, R^2^ = Cl), and **59k** (R = NO_2_, R^1^ = F, R^2^ = H) were found to be best as they exhibited MIC values of 64 µM against *S. aureus* and *B. subtilis*. Whereas **59h** (R = Br, R^1^ = F, R^2^ = Cl) was found to be best against *S. aureus* and *B. subtilis* with lowest MIC of 32 µM. However, compounds did not exhibited any activity against Gram-negative bacteria.

**Scheme 24:**
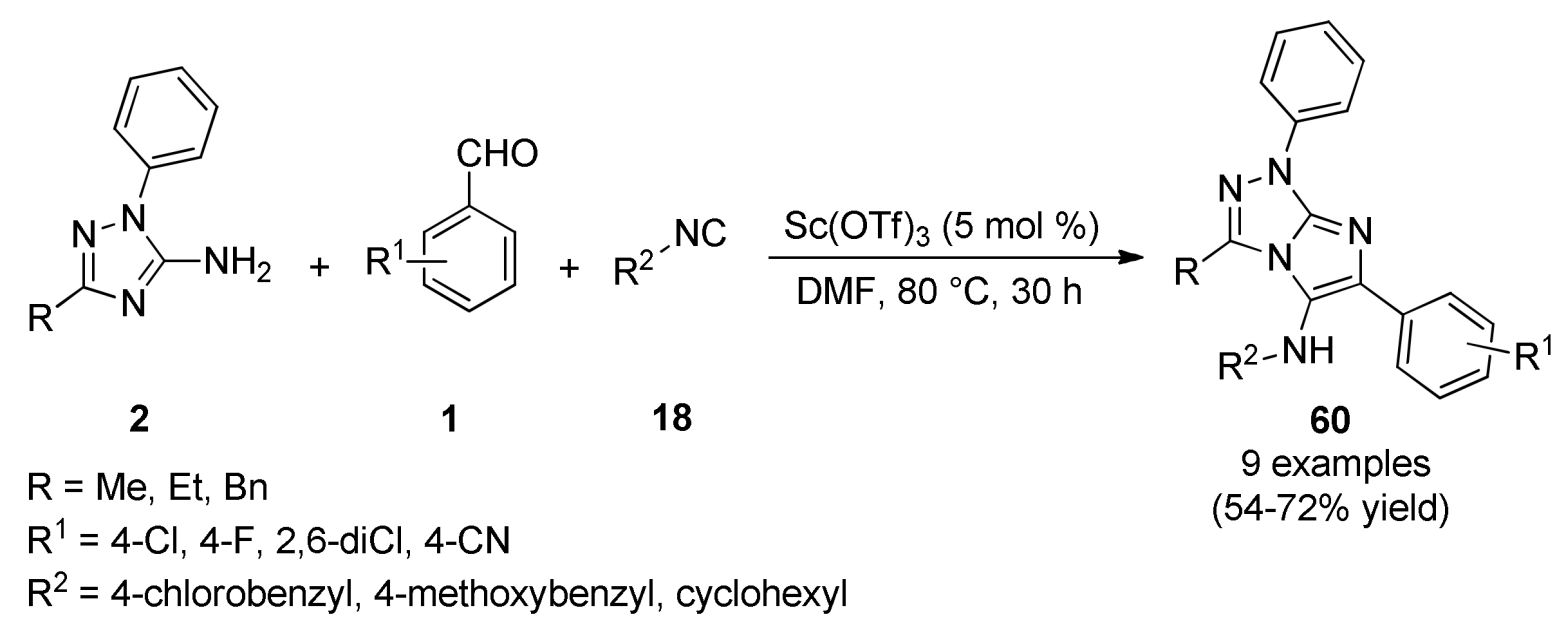
Groebke-Blackburn-Bienaymé three-component reaction for the preparation of imidazo[2,1-*c*][1,2,4]triazole derivatives **60** for antibacterial assays.

During the course of two decades the Groebke-Blackburn-Bienaymé (GBB-3CR) reaction has emerged as a very important multicomponent reaction (MCR), resulting in over a hundred patents and a great number of publications in various fields of interest [66]. For example, Aouali et al., described the synthesis of fully substituted imidazo[2,1-*c*][1,2,4]triazoles **60** by a Groebke-Blackburn-Bienaymé reaction between 5-amino-1,2,4-triazoles **2**, aromatic aldehydes **1** and isocyanides **18** using scandium triflate as a Lewis acid catalyst (Scheme 24). The synthesized derivatives **60** were screened for their antibacterial but also, for antifungal and antioxidant activities [67]. Compounds **60** were screened for their antibacterial activity against two Gram-positive bacteria (*Bacillus cereus*, *Staphylococcus aureus*) and three Gram-negative bacteria (*Escherichia coli*, *Pseudomonas aeruginosa*, *Salmonella enteritidis*). Particularly, compound **60b** (R = Me, R^1^ = 4-Cl, R^2^ = cyclohexyl) exhibited excellent inhibition against the Gram-positive bacteria with inhibition zone diameter ranged from 29 to 20 mm and MIC values of 78 and 312 µM against *B. cereus* and *S. aureus*, respectively. However, these compounds were inactive against Gram-negative bacteria.

**Scheme 25:**
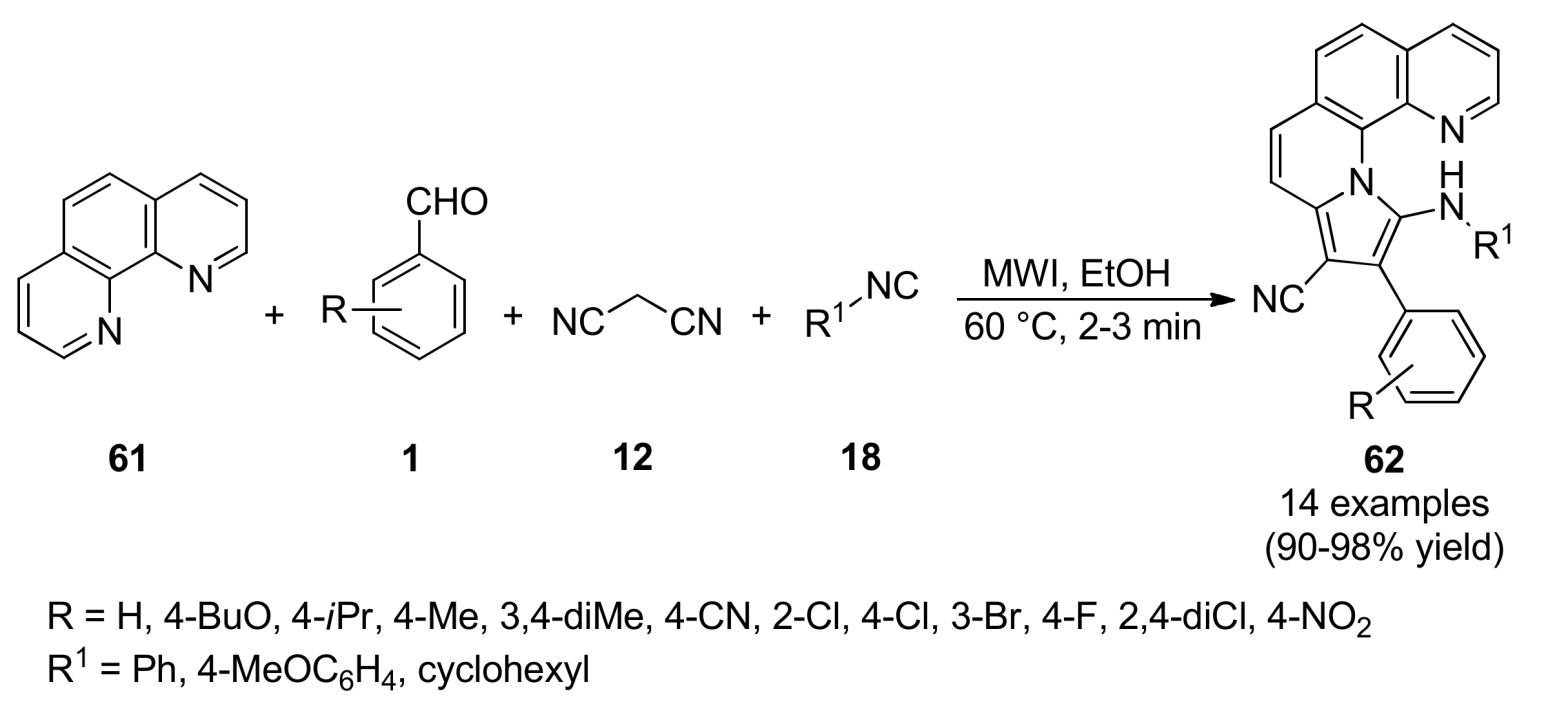
Microwave-assisted multicomponent synthesis of pyrrolo[1,10]phenanthrolines **62** for antibacterial evaluation.

In 2017, an elegant microwave-assisted synthesis of pyrrolo[1,10]phenanthrolines **62** was achieved through a four-component reaction between 1,10-phenanthroline (**61**), aromatic aldehydes **1**, malononitrile (**12**) and isocyanides **18**, leading to the corresponding products **62** in excellent yields at 60 °C (Scheme 25) [68]. The benefits of MWI in terms of reaction times and efficiency were clearly demonstrated by a comparative study with thermal activation. Subsequently, compounds **62** were screened for antibacterial, as well as, for antitumoral and antifungal activity. These compounds were screened for their antibacterial activity against two Gram-positive bacteria (methicillin-resistant *Staphylococcus aureus*, *Bacillus subtilis*) by the agar diffusion method using erythromycin and oxacillin as standard drugs. Remarkably, compounds **62d** (R = 4-Cl, R^1^ = 4-MeOC_6_H_4_) and **62l** (R = 4-F, R^1^ = 4-MeOC_6_H_4_) exhibited the highest antibacterial activity with inhibition zones of 29 mm and 27 mm, respectively, against methicillin-resistant *S. aureus* bacteria.

**Scheme 26:**
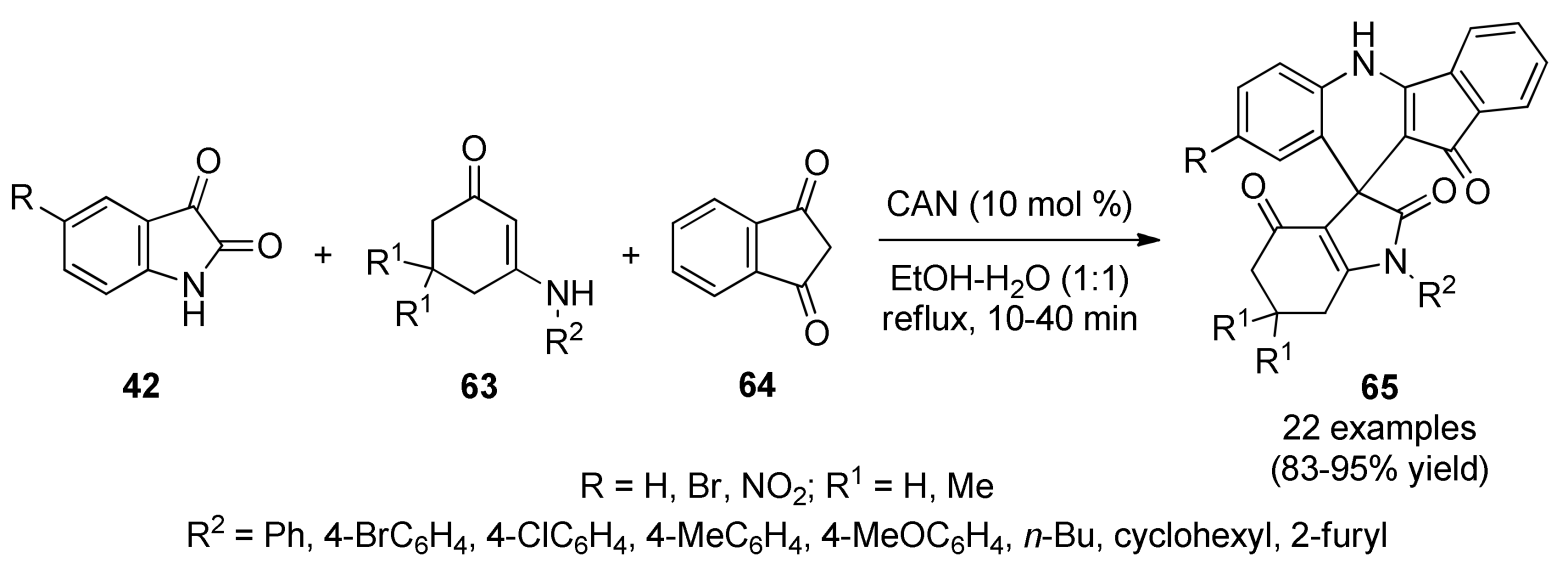
CAN-mediated three-component synthesis of spiro[indolo-3,10′-indeno[1,2-*b*]quinolin]-2,4,11′-triones **65** of antibacterial interest.

Alternatively, Meena et al., described a three-component synthesis of spiro[indolo-3,10′-indeno[1,2-*b*]quinolin]-2,4,11′-triones **65** using isatins type **42**, enaminones **63** and indane-1,3-dione (**64**) [69]. As a result, a collection of spiro[indolo-3,10′-indeno[1,2-*b*]quinolin]-2,4,11′-triones **65** was synthesized in the presence of a catalytic amount of ceric ammonium nitrate (CAN) in ethanol–water (1:1) under refluxing conditions (Scheme 26). This method provided several advantages such as shorter reaction times, high yields and operational simplicity. Compounds **65** were screened for their antibacterial activity against two Gram-positive bacteria (*Staphylococcus aureus*, *Bacillus subtilis*) and two Gram-negative bacteria (*Escherichia coli*, *Pseudomonas aeruginosa*) using ciprofloxacin as standard drug. The minimum inhibitory concentration (MIC) of tested compounds ranged between 8 and 512 µM. Compounds **65c** (R = H, R^1^ = Me, R^2^ = 4-ClC_6_H_4_) and **65n** (R = NO_2_, R^1^ = Me, R^2^ = Ph) exhibited the lowest MIC of 16 µM against *S. aureus*. It is important to note that compound **65c** exhibited lowest MIC of 8 µM and 64 µM against *B. subtilis* and *E. coli*, respectively.

**Scheme 27:**
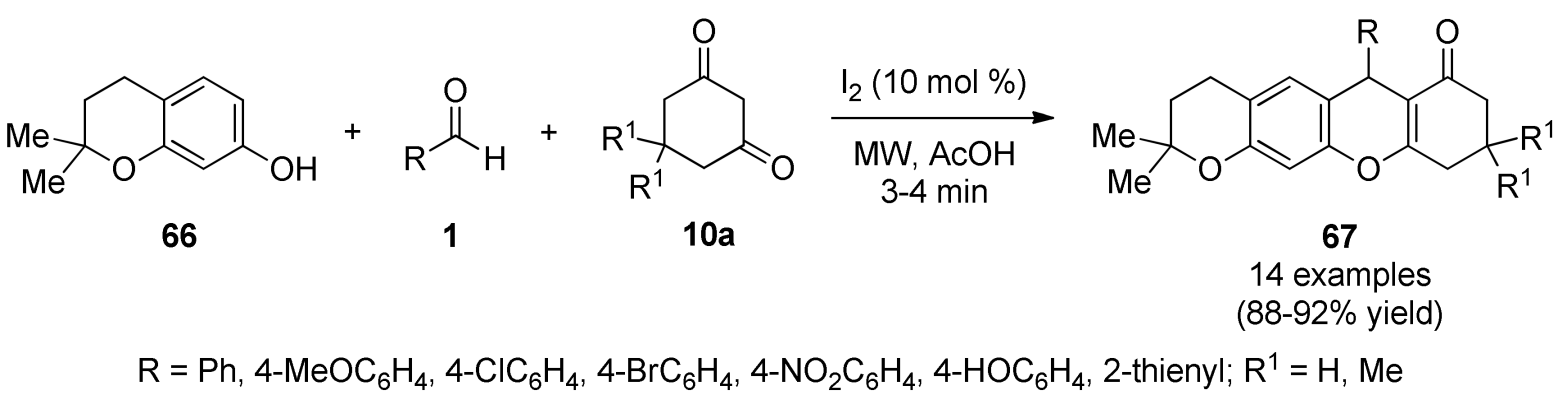
MW-assisted three-component synthesis of pyrano[3,2-*b*]xanthen-7(2*H*)-ones **67** for antibacterial screening.

Very recently, Ashok et al., reported an efficient synthesis of pyrano[3,2-*b*]xanthen-7(2*H*)-ones **67** in good to excellent yields [70]. This I_2_-catalyzed three-component reaction involved 2,2-dimethylchroman-7-ol (**66**), aromatic aldehydes **1** and 1,3-cyclohexanediones **10a** under MWI in acetic acid (Scheme 27). This transformation featured multiple bonds breaking and forming events in a single, atom-economic operation. The synthesized pyrano[3,2-*b*]xanthen-7(2*H*)-ones were screened for antibacterial, as well as, for antifungal and antioxidant activities. Compounds **67** were screened for their antibacterial activity against two Gram-positive bacteria (*Bacillus subtilis*, *Staphylococcus aureus*) and two Gram-negative bacteria (*Escherichia coli*, *Klebsiella pneumoniae*) by the disc diffusion method using ciprofloxacin as standard antibiotic. Among all the synthesized compounds **67j** (R = 4-NO_2_C_6_H_4_, R^1^ = Me) (16.6 mm) and **67n** (R = 2-thienyl, R^1^ = Me) (15.5 mm) showed good zone of inhibition against *S. aureus* compared to standard ciproflaxin (13.6 mm) in both concentrations (20 µM and 40 µM).

**Scheme 28:**
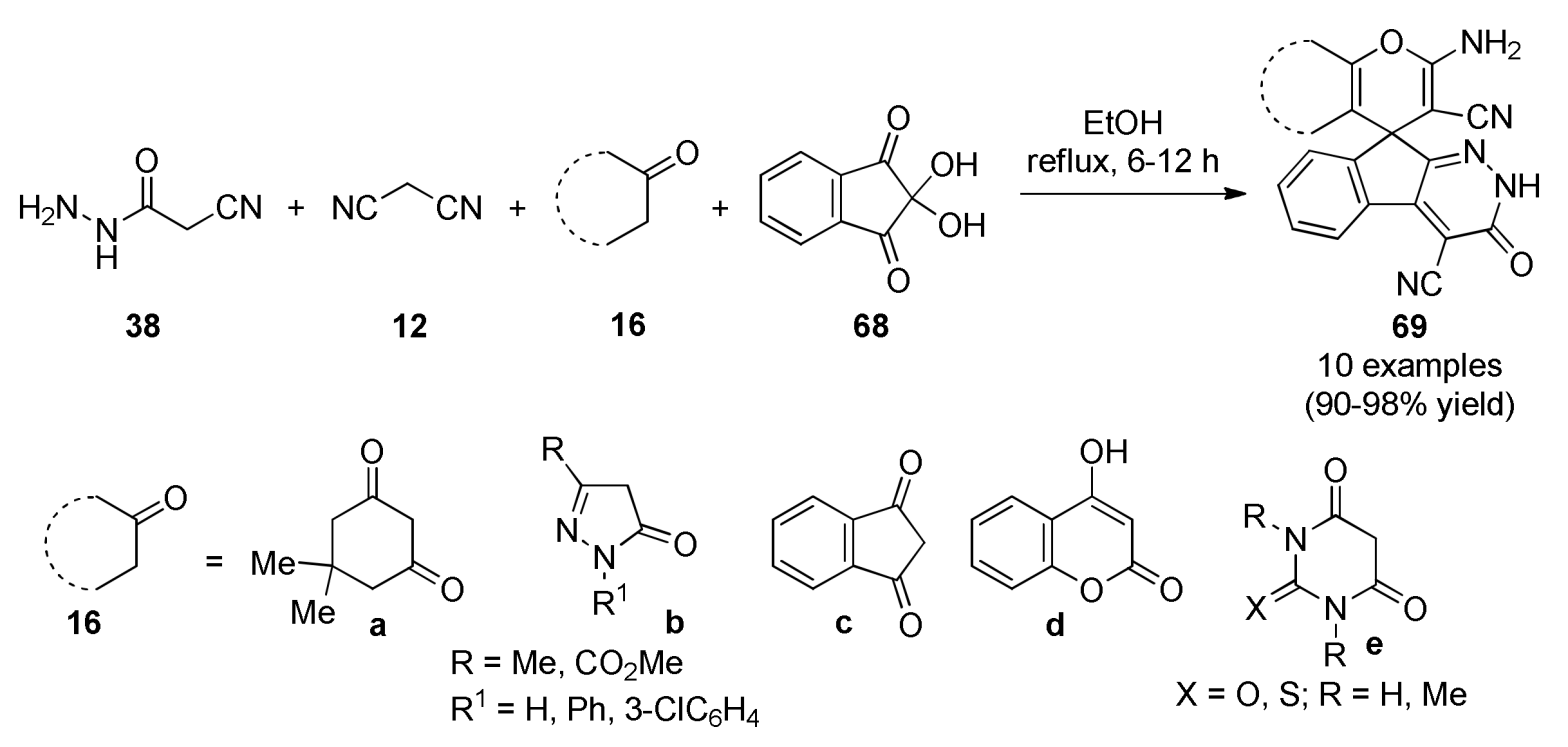
Multicomponent synthesis of spiroindenopyridazine-4*H*-pyran derivatives **69** of antibacterial interest.

More recently, Safari et al., reported a catalyst-free four-component reaction of cyanoacetohydrazide **38**, malononitrile (**12**), diverse cyclic CH-acids type **16** and ninhydrin (**68**) in refluxing ethanol to afford spiro-4*H*-pyran derivatives **69** in excellent yields (Scheme 28). The efficiency of this multicomponent reaction to access complex skeleton is once again remarkable [71]. Compounds **69** were screened for their antibacterial activity against one Gram-positive bacterium (*Staphylococcus aureus*) and one Gram-negative bacterium (*Escherichia coli*) by the disc diffusion method using tetracycline as standard antibiotic. Disc diffusion data showed inhibition zones of 4–15 mm for the synthesized compounds against *S. aureus*, except for **69a** (from **16a**), **69b** (from **16b** with R = CO_2_Me, R^1^ = Ph) and **69c** (from **16c**), compared to standard tetracycline (30 mm). However, *E. coli* was resistant against all compounds tested.

**Scheme 29:**
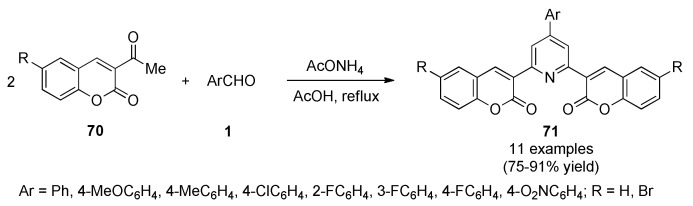
Multicomponent synthesis of 2,6-*bis*(1-coumarin-2-yl)-4-(4-substituted phenyl)pyridine derivatives **71** for antibacterial evaluation.

A one-pot three-component synthesis of 2,6-*bis*(1-coumarin-2-yl)-4-(4-substituted phenyl) pyridine derivatives **71** by a Chichibabin reaction was designed. These compounds were synthesized in 75–91% yield by the reaction of 3-acetyl coumarins **70** with substituted aromatic aldehydes **1** and ammonium acetate under acidic conditions (Scheme 29). The synthesized compounds **71** were evaluated for antimicrobial activity, as well as for DPPH free radical scavenging activity and ferrous ion-chelating ability. The mode of action of the active compounds was established by docking with the receptor GlcN6P synthase. The antimicrobial results revealed that compounds containing halogen or electron-withdrawing substituents either on the coumarin or phenyl rings exhibited potent antimicrobial and antioxidant activities. In the antibacterial studies, compounds bearing a bromo group in addition to a chloro group exhibited greater activity than those bearing only chloro groups. This observation suggested that di-substitution in the target compounds **71** by halogens enhanced the antimicrobial and antioxidant potential [72].

**Scheme 30:**
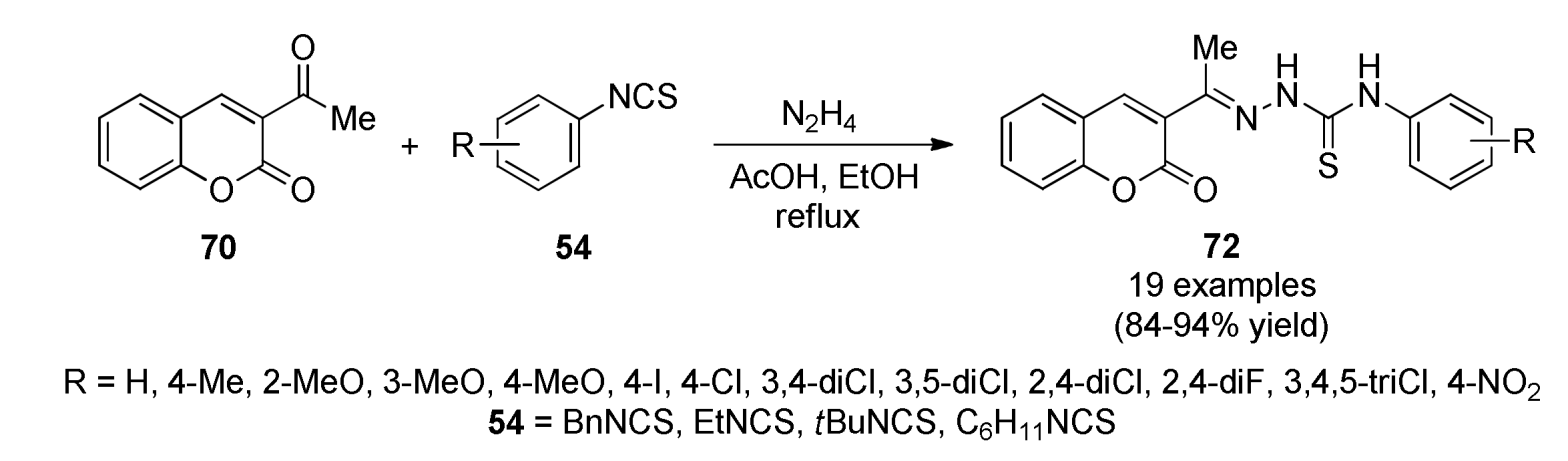
One-pot and solvent-free three-component synthesis of coumarin-thiosemicarbazone hybrids **72** of antibacterial interest.

A convenient, one-pot, three-component protocol for the preparation of 2-(1-(2-oxo-2*H*-chromen-3-yl)ethylidene)hydrazinecarbothioamide derivatives **72** was achieved. Firstly, the synthesis of 3-acetyl-2*H*-chromen-2-one type **70** was carried out using starch sulfuric acid and cellulose sulfuric acid as biodegradable catalysts. Subsequently, the reaction of **70** with isothiocynates type **54** and hydrazine hydrate in the presence of catalytic amount of glacial acetic acid in refluxing ethanol afforded the target products **72** in 84–94% yield (Scheme 30). All synthesized compounds **72** were screened for antimicrobial activity. All compounds were found to show good to excellent activity against *Escherichia coli* MTCC 443 [73].

**Scheme 31:**
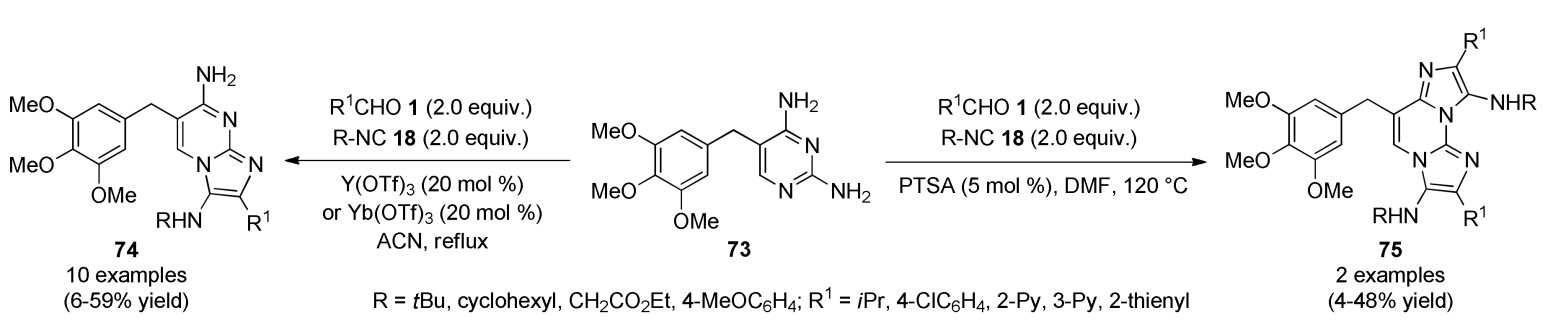
Three-component synthesis of mono-(**74**) and double-(**75**) Trimethoprim-based GBBR adducts for antibacterial evaluation.

A library of trimethoprim-based antibiotic compounds **74** and **75** was prepared through a selective multicomponent reaction upon the known drug trimethoprim **73**. A Groebke-Blackburn-Bienaymé reaction of **73** with aldehydes **1** and isocyanides **18** afforded the desired adducts **74** and **75** in 6–59% and 4–48% yields, respectively, in one-step (Scheme 31). The analogs **74** and **75** displayed meaningful structural features of the initial drug together with relevant modifications at several points, keeping antibiotic potency and showing satisfactory antimicrobial profile (good activity levels and reduced growth rates), especially against methicillin-resistant *Staphylococcus aureus* [74].

### 3.11. Antioxidant Activity

Compounds that exhibit antioxidant activity due to their chemical structure and redox properties have an important role in the uptake of singlet and triplet oxygen species, responsible for cardiovascular diseases, inflammatory bowel syndrome, cancer, aging, atherosclerosis and Alzheimer’s disease [75]. Different areas converge on the need to synthesize antioxidant compounds as protection against these diseases. Thereby, Lakshmi et al., reported the three-component synthesis of the 3-pyranyl indoles **33** described previously in Scheme 12 [54]. The antioxidant activity of such compounds was determined using methods for antioxidant activity estimation such as DPPH and ABTS. Thus, compounds **33m** (R = 4-MeO), **33n** (R = 3,4-diMeO), **33o** (R = 2-Br-4,5-diMeO) and **33p** (R = 4-NMe_2_) showed the most relevant radical scavenging activity in both methods due to the presence of electron-donating groups such as –OMe and –NMe_2_ with an IC_50_ < 50 μM in DPPH method. IC_50_ values of 858.6 μM (**33m**), 867.2 μM (**33n**), 880.3 μM (**33o**) and 900.0 μM (**33p**) were observed using the ABTS method. Ascorbic acid was used as standard, giving an IC_50_ < 50 μM in the DPPH method, and 650.0 μM in the ABTS method.

Lal et al., reported a time-efficient Biginelli reaction for the synthesis of curcumin 3,4-dihydropyrimidinones/thiones/imines **7** as described previously in Scheme 2 [28]. These compounds were screened for their antioxidant activity using ferric reducing antioxidant power (FRAP), cupric reducing antioxidant capacity (CUPRAC) and DPPH radical scavenging activity (DRSA) assays. Notably, compound **7e** derived from 2,4-dihydroxybenzaldehyde and urea showed maximum DRSA antioxidant activity (IC_50_ = 3.86 μM) compared to ascorbic acid (IC_50_ = 3.76 μM), while the antioxidant activity decreased in compounds derived from 2-hydroxybenzaldehyde, 3-hydroxybenzaldehyde, and 4-hydroxybenzaldehyde, confirming the importance of the location of hydroxyl groups in the structure in order to potentiate the antioxidant activity.

**Scheme 32:**
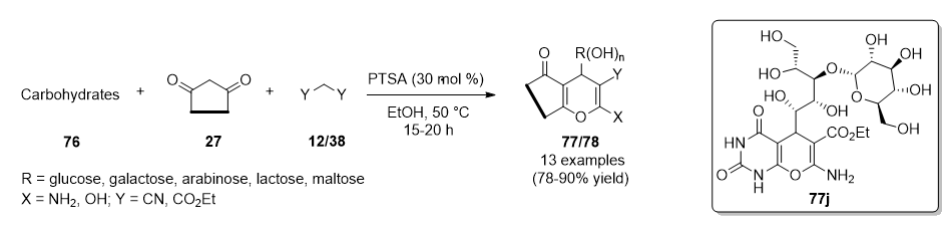
PTSA-catalyzed three-component synthesis of pyrano[2,3-*d*]pyrimidines **77** and chromene-3-carbonitriles **78** for antioxidant assays.

A combinatorial approach carried out by Dangolani, et al., allowed obtaining a wide range of heterocyclic compounds [76]. In order to obtain the pyrano[2,3-*d*]pyrimidines **77**, diverse carbohydrates **76** such as glucose, galactose, arabinose, maltose and lactose reacted with barbituric acid (**27a**) and malononitrile/diethylmalonate (**12**)**/**(**38**) in ethanol and PTSA as catalyst, in a three-component fashion. Additionally, barbituric acid (**27a**) was replaced by cyclohexane-1,3-dione (**27b**) to give two chromene-3-carbonitriles **78** in good yields (Scheme 32).

**Scheme 33:**
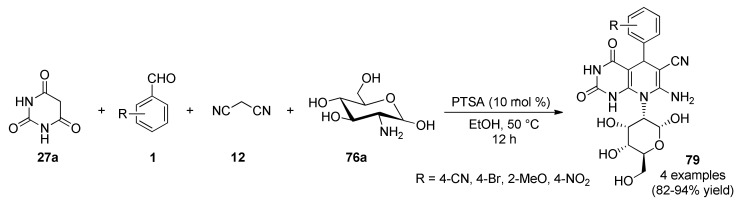
Four-component synthesis of pyrido[2,3-*d*]pyrimidines **79** for antioxidant assays.

Additionally, the same authors reported a four-component reaction of barbituric acid (**27a**), aromatic aldehyde **1**, malononitrile (**12**) and D-glucosamine (**76a**) to furnish the polyhydroxylated pyrido[2,3-*d*]pyrimidines **79** under the same synthetic conditions mentioned above including PTSA as Brønsted acid catalyst and ethanol as solvent (Scheme 33) [76]. 

The above three families of synthesized compounds were evaluated using 2,2′-azino-*bis*(3-ethylbenzothiazoline-6-sulfonic acid) antioxidant measuring system. The finding indicated that the maximum antioxidant activity occurred in the pyrano[2,3-*d*]pyrimidine **77j** which is derived from barbituric acid, ethylcyanoacetate and maltose, Scheme 32, with a Trolox equivalent antioxidant capacity (TEAC) of 5.23 μM.

Within the heterocyclic compounds, the xanthene moiety is a key target due to the large number of biologically active molecules that contain it. Accordingly, Ashok et al., developed a microwave-assisted three-component reaction for the synthesis of pyrano[3,2-*b*]xanthen-7(2*H*)-ones **67** as previously was described in Scheme 27 [70]. The measurement of the antioxidant activity of the synthesized compounds was performed by DPPH free radical scavenging assay using ascorbate as a standard antioxidant. Interestingly, compounds **67g** (R = 4-BrC_6_H_4_, R^1^ = H) (81.86%), **67i** (R = 4-NO_2_C_6_H_4_, R^1^ = H) (83.25%) and **67k** (R = 4-HOC_6_H_4_, R^1^ = H) (82.22%) showed a slightly lower DPPH antioxidant activity than standard ascorbate (86.50%).

**Scheme 34:**
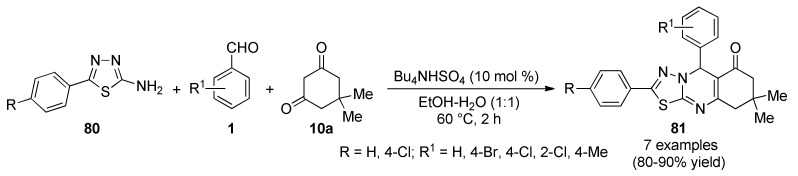
Three-component synthesis of thiadiazolo[2,3-*b*]quinazolin-6-(7*H*)-ones **81** for antioxidant evaluation.

The multicomponent synthesis of thiadiazolo[2,3-*b*]quinazolin-6-(7*H*)-ones **81** could be accomplished from 2-amino-5-phenyl-1,3,4-thiadiazoles **80**, aromatic aldehydes **1** and dimedone (**10a**) in the presence of catalytic amounts of tetrabutylammonium hydrogen sulfate (Bu_4_NHSO_4_) in ethanol–water (1:1) under refluxing conditions (Scheme 34). Besides, this study showed that thiadiazolo[2,3-*b*]quinazolin-6-(7*H*)-ones **81** could be formed with high atom-economy involving the formation of one C-C and two C-N bonds in only one step [77]. The antioxidant activity of the synthesized compounds was screened by DPPH and OH radical scavenging assays using ascorbic acid as reference compound. Compounds **81a** (R = H, R^1^ = 4-Br), **81e** (R = 4-Cl, R^1^ = H) and **81f** (R = 4-Cl, R^1^ = 4-Br) showed remarkable DDPH radical scavenging activity with values of 88.4, 87.6 and 85.4%, respectively, as compared with ascorbic acid (91.4%). In addition, compounds **81a**, **81b** (R = H, R^1^ = 4-Cl) and **81f** showed good OH radical scavenging activity with values between 79.3 and 87.0%, as compared with ascorbic acid (89.5%).

**Scheme 35:**
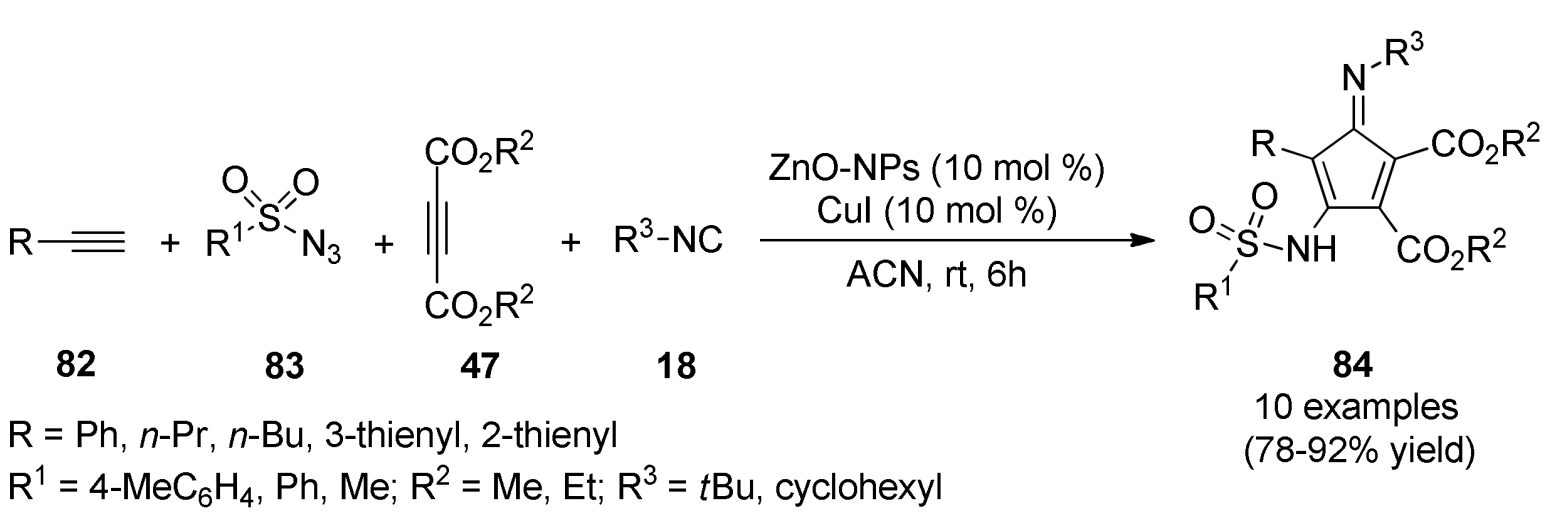
Four-component synthesis of 1,3-cyclopentadiene derivatives **84** for their DPPH scavenging power evaluation.

Very recently, a catalytic four-component synthesis of 1,3-cyclopentadiene derivatives was described by Ezzatzadeh et al. [78]. This unique and mild access to fully substituted 1,3-cyclopentadienes **84** involved terminal alkynes **82**, sulfonyl azides **83**, activated acetylenic compounds **47** and isocyanides **18** in the presence of zinc oxide nanoparticles (ZnO-NPs) and copper iodide as catalytic system in acetonitrile at room temperature (Scheme 35). This procedure offered several advantages such as being eco-friendly, ZnO-NPs could be re-used; the work-up was easy, non-toxic, and had a cleaner reaction profile. Antioxidant activity was investigated for the synthesized compounds **84a** (R = Ph, R^1^ = 4-MeC_6_H_4_, R^2^ = Me, R^3^ = *t*Bu), **84b** (R = Pr, R^1^ = Ph, R^2^ = Me, R^3^ = *t*Bu), **84c** (R = Bu, R^1^ = Me, R^2^ = Me, R^3^ = *t*Bu), and **84d** (R = Pr, R^1^ = Me, R^2^ = Et, R^3^ = *t*Bu) using the DPPH radical trapping and comparing results with synthetic antioxidants (TBHQ and BHT). The DPPH scavenging power was achieved in the following order: TBHQ > BHT > **84a** > **84c** > **84d** > **84b**, respectively. The free radical scavenging power was enhanced from 200 to 1000 ppm concentrations. For example, concentration 1000 ppm of **84a** had 30.2% inhibition, whereas 200 ppm of **84a** showed 9.6% free radical inhibition.

### 3.12. Anti-Mycobacterial Activity

**Scheme 36:**
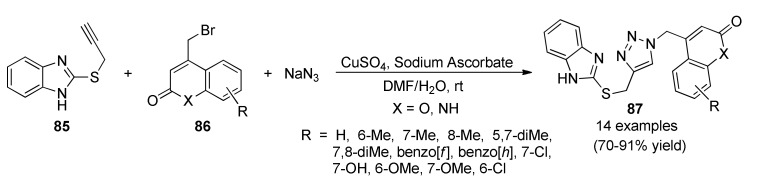
Three-component one-pot synthesis of 1,4-substituted triazoles **87** for evaluation against *M. tuberculosis* (H37Rv).

Emergence of resistance against new tuberculosis (TB) drugs is an alarming issue demanding new drug profiles. For that, designing drugs for the treatment of tuberculosis has been a challenging area in medicinal chemistry in view of the multi-drug resistance [79] and high mortality rate [80] associated with this disease. Among the approved anti-tubercular drugs, benzimidazole derivatives appear as privileged and promising structures in chemotherapy of tuberculosis [81,82,83]. As a contribution to this topic, Anand et al., reported a three-component reaction for the synthesis of 2-mercaptobenzimidazole linked coumarinyl triazoles **87** as anti-tubercular agents. The process is mediated by a Click type reaction between 2-propargylthiobenzimidazole (**85**), 4-bromomethyl coumarins/1-aza-coumarins **86** and sodium azide, in the presence of CuSO_4_/sodium ascorbate as catalytic system (Scheme 36). The anti-tubercular assays of the obtained compounds **87** against *M. tuberculosis* (H37Rv) coupled with in silico molecular docking studies indicated that dimethyl substituted products **87c** (X = O, R = 5,7-diMe) and **87d** (X = O, R = 7,8-diMe) showed promising activity with higher C-score values [84].

**Scheme 37:**
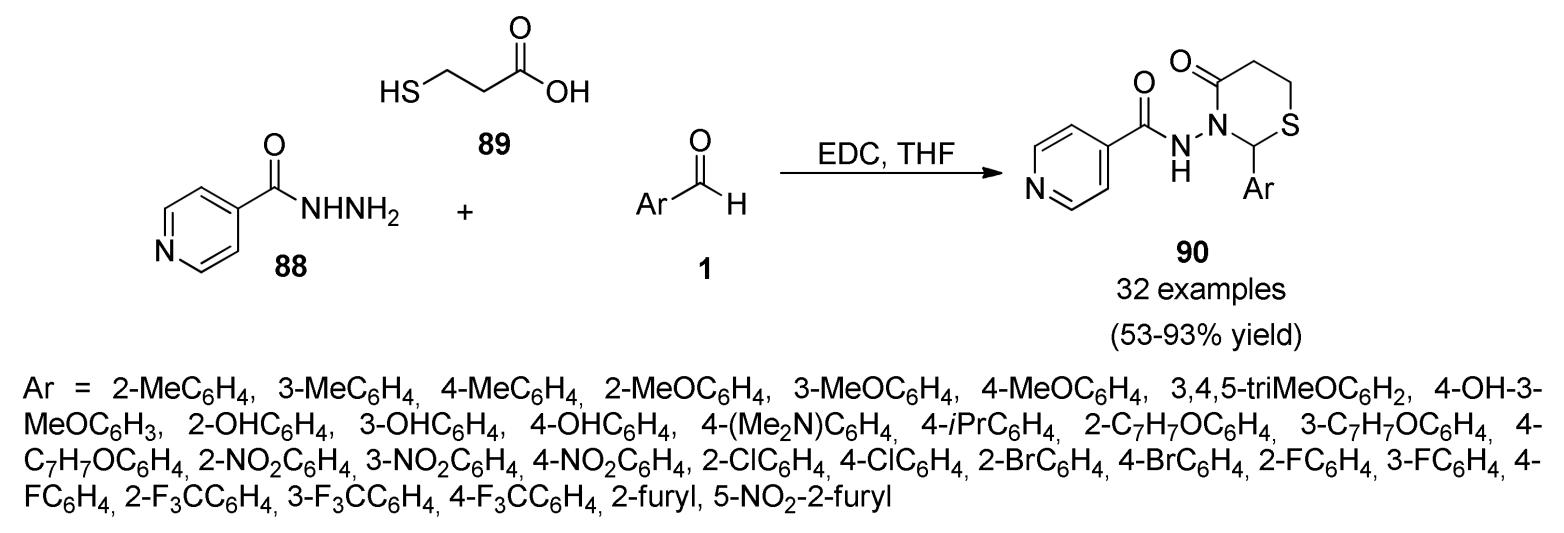
Three-component synthetic protocol of compounds **90** as potential antitubercular agents.

Isoniazid analogues **90** were prepared by three-component condensation reactions of isoniazid (INH) **88**, 3-mercaptopropionic acid **89** and various aldehydes **1** in THF as solvent and EDC (1-ethyl-3-(3-dimethylaminopropyl) carbodiimide) as activating agent (Scheme 37). 

The products were evaluated against *Mycobacterium tuberculosis* H37Rv (MTB) and cytotoxicity. All the obtained compounds showed in vitro activity against MTB with MIC ranging from 0.12 to 41.2 μM. Thirteen compounds inhibited MTB with less than 1 μM. Six compounds were more potent compared than the standard first line drug INH (MIC of 0.36 μM) with MIC less than 0.36 μM and the compound *N*-(2-(4-(benzyloxy)phenyl)-4-oxo-1,3-thiazinan-3-yl)sonicotinamide inhibited MTB with MIC of 0.12 μM and was three times more potent than INH [85].

**Scheme 38:**
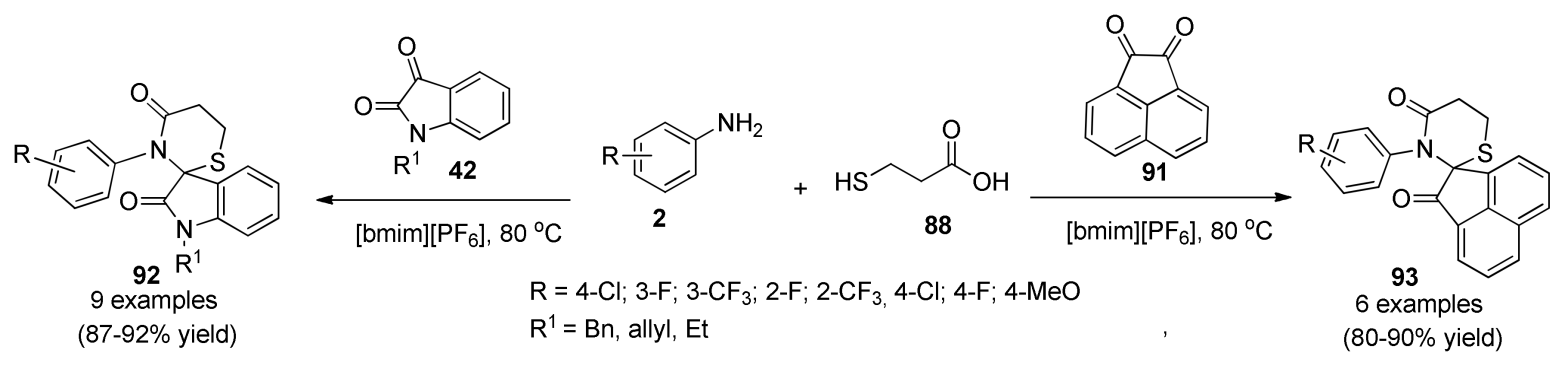
[bmim][PF_6_]-Mediated three-component synthesis of spiro[indole-3,2′[1,3]-thiazine]-2,4-diones **93** of antitubercular interest.

An efficient and green method was reported for the synthesis of fluorinated spiro-thiazine derivatives **92** and **93** via a three-component reaction, using 1-butyl-3-methylimidazolium hexafluorophosphate [bmim][PF_6_] as solvent and catalyst (Scheme 38). The synthesized compounds were subjected to antimycobacterial efficacy against *Mycobacterium tuberculosis* H37Rv strain and DNA cleavage activity. All compounds exhibited very poor antitubercular activities but DNA cleavage studies revealed that the tested compounds exhibited promising cleavage activity [86].

**Scheme 39:**
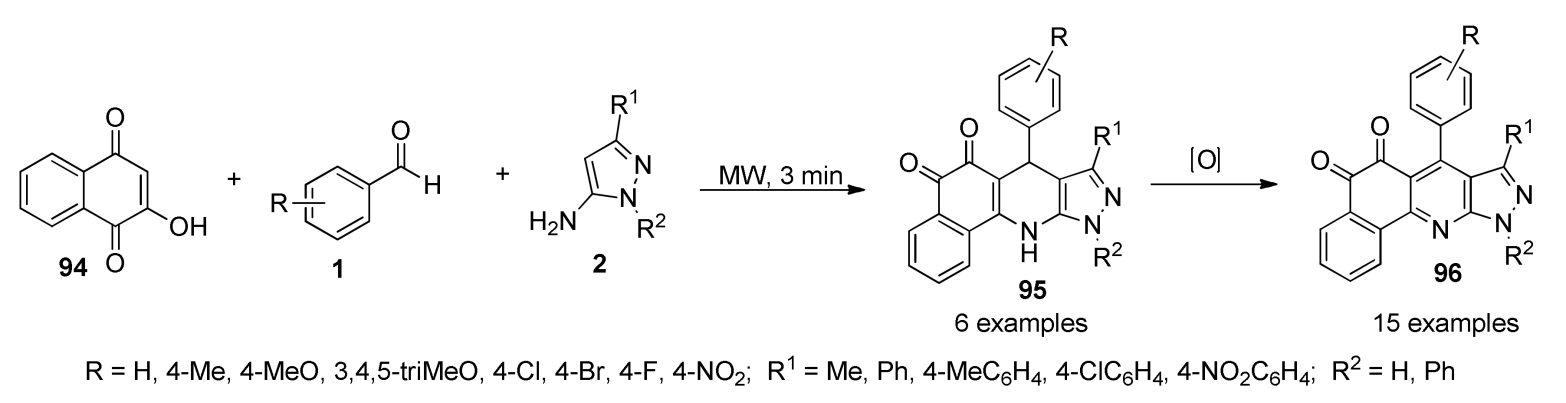
Microwave-assisted three-component synthesis of benzopyrazolo[3,4-*b*]quinolindiones **95/96** for antimycobacterial activity evaluation.

Quiroga et al., reported the synthesis and antimycobacterial activity of 4-arylbenzopyrazolo[3,4-*b*]quinolindiones **95** (in 70–80% yield) and **96** (in 70–85% yield). These compounds were obtained via a three-component reaction between 2-hydroxynaphthoquinone (**94**), benzaldehydes **1** and aminopyrazoles type **2**, assisted by MWI (Scheme 39). Compounds **95** and **96** were evaluated against fifteen *Mycobacterium spp* strains, and six of them showed antimycobacterial activity. The highest inhibitory activity with MIC ≤ 2 μM for three of these compounds **96a** (R = H, R^1^ = Me, R^2^ = Ph), **96b** (R = 4-Me, R^1^ = Me, R^2^ = Ph) and **96g** (R = 4-F, R^1^ = Me, R^2^ = Ph) was related with their highest lipophilicity and lesser polarity within these series [87].

**Scheme 40:**
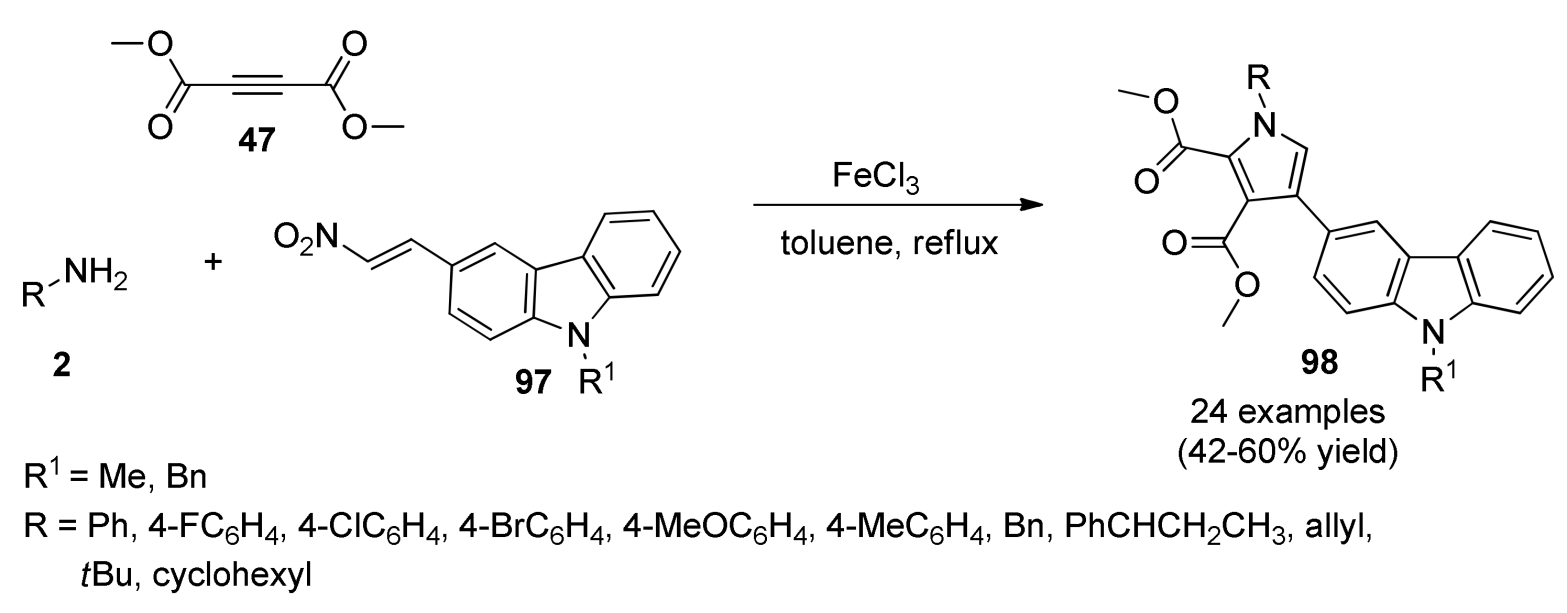
Three-component synthesis of carbazole-tethered substituted pyrrole analogs **98** for anti-mycobacterial activity against *Mycobacterium tuberculosis* H37Rv.

Carbazole-tethered pyrrole derivatives **98** were synthesized via a one-pot three-component condensation of 2-nitrovinylcarbazoles **97** with aryl or alkyl amines **2** and dimethylacetylene dicarboxylate **47** (DMAD) in the presence of FeCl_3_ as an effective catalyst (Scheme 40). Twenty-four compounds were screened for in vitro anti-mycobacterial activity against *Mycobacterium tuberculosis* H37Rv. The compound dimethyl 1-(4-fluorophenyl)-4-(9-methyl-9*H*-carbazol-3-yl)-1*H*-pyrrole-2,3-dicarboxylate was the most active with MIC = 3.13 μM showing low cytotoxicity [88].

**Scheme 41:**
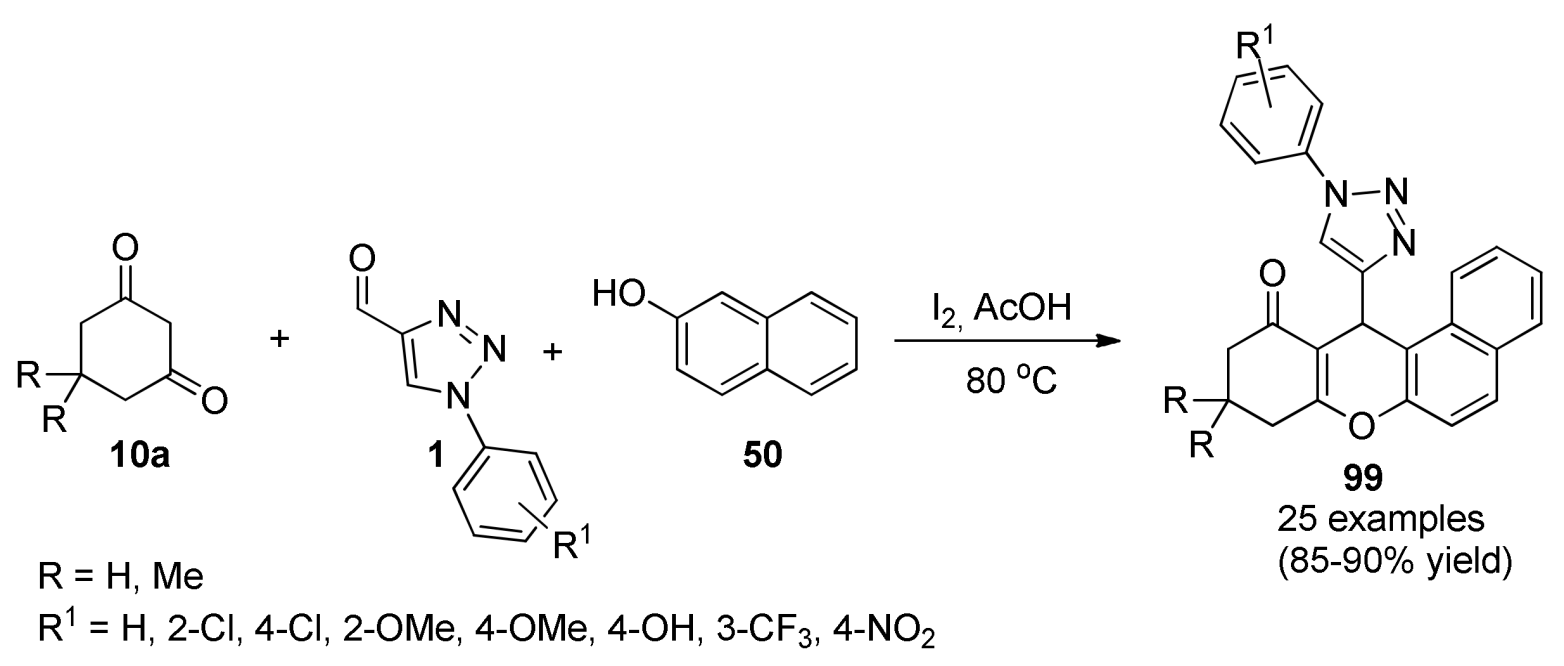
Iodine-mediated three-component synthesis of 1,2,3-triazolyl-xanthenones **99** for antitubercular activity against *M. tuberculosis* H37Rv (ATCC27294) strain.

Ashok et al., reported the antitubercular and antimicrobial activity the 1,2,3-triazolyl xanthenones **99**. The synthetic process involved a one-pot three-component thermal cyclization reaction of cyclic-1,3-diones type **10a**, 1-aryl-1*H*-[1,2,3]triazole-4-carbaldehydes **1** and *β*-naphthol (**50**) in the presence of catalytic amount of iodine (Scheme 41). Xanthenone derivatives **99** were screened for in vitro antitubercular activity against *M. tuberculosis* H37Rv (ATCC27294) strain, as well as, for antibacterial activity against Gram-positive, Gram-negative strains and antifungal activity against a pathogenic strain of fungi. The antimycobacterial results indicated that compounds **99c** (R = Me, R^1^ = 4-Cl), **99e** (R = Me, R^1^ = 4-MeO) and **99k** (R = H, R^1^ = 4-Cl) displayed good antitubercular activity, especially, compounds with substituent at the 4-position of the Ar ring (i.e., 4-ClC_6_H_4_ and 4-MeOC_6_H_4_). Moreover, derived of the 1,3-cyclohexadione type **10a** containing methyl groups (i.e., R = Me) did not display activity. The molecular docking studies of compounds active against pantothenate synthetase revealed the favorable interactions with amino acid residues of such enzyme [89].

### 3.13. Anticancer Activity

#### 3.13.1. Quinoline Derivatives

**Scheme 42:**
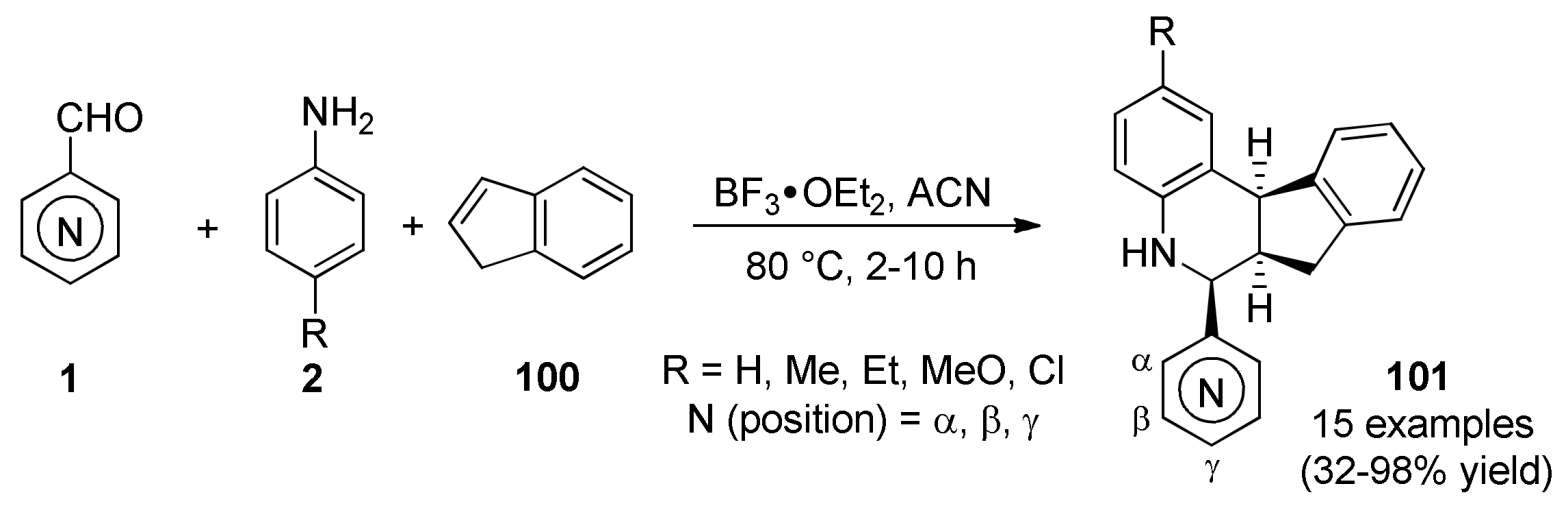
BF_3_∙OEt_2_–Catalyzed three-component synthesis of indeno[2,1-*c*]quinolines **101** of anticancer interest.

Quinoline is one of the most important *N*-based heterocyclic compounds. Furthermore, quinoline derivatives are known to have a broad range of applications in medicinal, bioorganic, and industrial chemistry as well as in the field of synthetic organic chemistry [90,91]. Due to its importance, the synthesis of the quinoline scaffold has been studied for well over a century through the classical methods including the well-known Skraup, Doebner–Miller, Friedländer and Combes reactions, among others [92,93]. In 2006 Kouznetzov et al., reported the three-component cyclization of pyridinecarbaldehydes **1**, anilines **2** and indene (**100**) in the presence of BF_3_∙OEt_2_ as catalyst [94]. The process involved an imino Diels-Alder cycloaddition affording the corresponding indeno[2,1-*c*]quinolines **101** in 32−98% yield (Scheme 42). Nearly all of the obtained products **101** were active against breast (MCF-7), lung (H-460) and CNS (SF-268) human cancer cell lines. Regarding the results, the activity of products **101** seems not to be related to the electronic properties of the R substituent. In contrast, the position of N-atom in the pyridine ring appears to exert some influence on the activity. Thus, compounds possessing α-pyridine were less active than the *β*- and *γ*- ones. According to the reviewed literature, this report correspond to the first example in which is combined both a synthetic approach mediated by a multicomponent reaction, as well as, the biological evaluation of the obtained products.

**Scheme 43:**
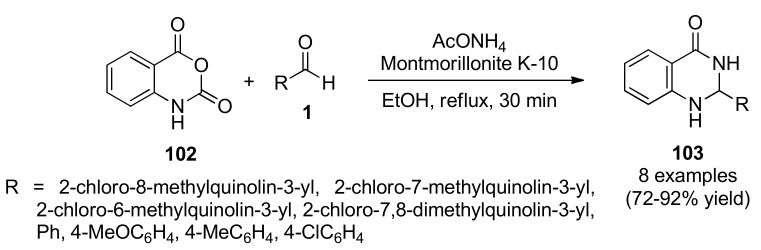
Three-component synthesis of 2-aryl-2,3-dihydroquinazolin-4(1*H*)-ones **103** for anticancer activity analysis.

Various heterogeneous catalysts have also been encountered for the synthesis of quinoline derivatives. In 2011, Roopan et al., found that Montmorillonite K10 catalyzed a three-component cyclocondensation reaction of isatoic anhydride (**102**), (hetero)aromatic aldehydes **1** and AcONH_4_ under mild reaction conditions to afford the corresponding 2-aryl-2,3-dihydroquinazolin-4(1*H*)-ones **103** in 72−92% yield (Scheme 43) [95]. This methodology offered very attractive features such as reduced reaction times, higher yields and economic viability of the catalyst. Furthermore, the catalyst could be recovered and reused without change in the yield and catalytic activity. The 2-aryl-2,3-dihydroquinazolin-4(1*H*)-ones **103** were screened for their antitumor activity against an Ehrlich Ascites Carcinoma (EAC) tumor cell line. The result suggested that compounds **103a** (R = 2-chloro-8-methylquinolin-3-yl), **103b** (R = 2-chloro-7-methylquinolin-3-yl), **103c** (R = 2-chloro-6-methyl-quinolin-3-yl) and **103d** (R = 2-chloro-7,8-dimethylquinolin-3-yl) had the lowest IC_50_ values compared to the standard 5-fluorouracil (132.12 μM) and hence higher cytotoxicity effects on EAC tumor cell line than the standard drug.

**Scheme 44:**
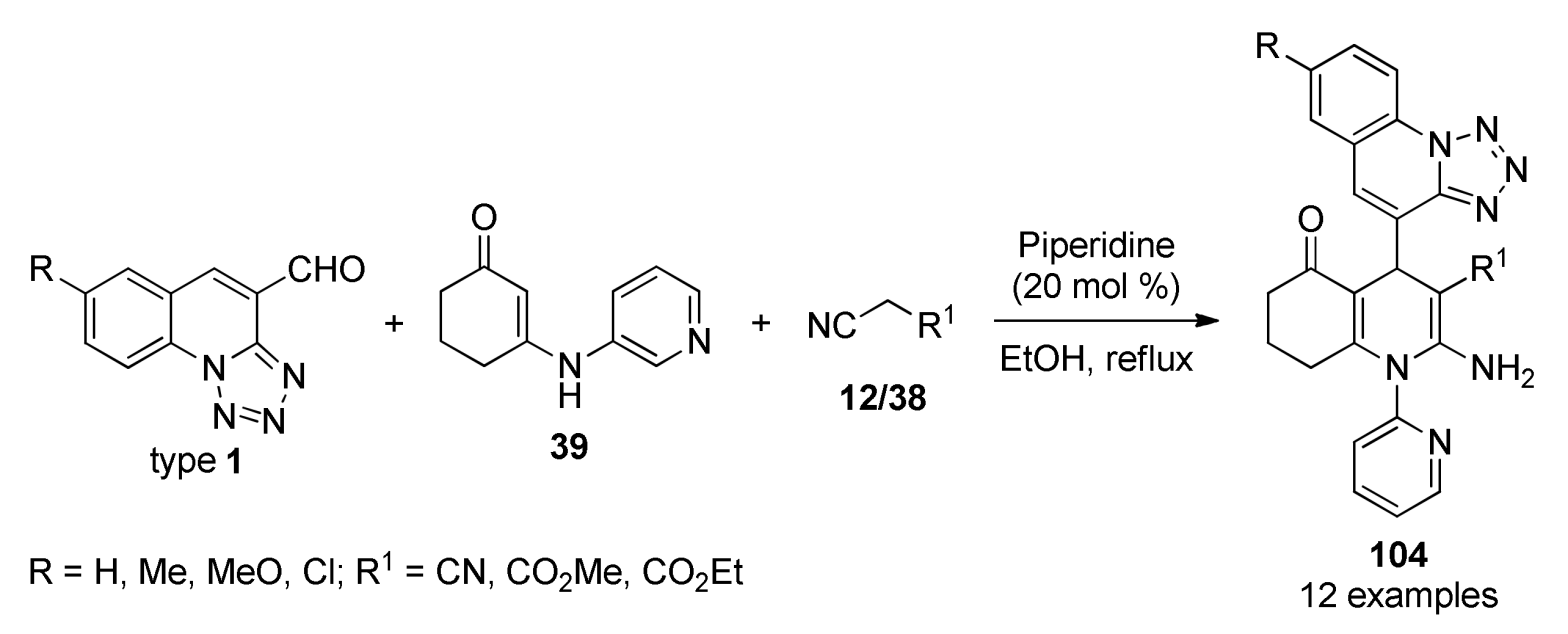
Piperidine-catalyzed three-component synthesis of biquinoline-pyridine hybrids **104** for in vitro anticancer activity against A549 and Hep G2 cancer cell lines.

The synthesis of quinoline derivatives has also been carried out with homogeneous catalysts such as, piperidine (20 mol%) under mild reaction conditions. In 2014, Sangani et al., applied this chemistry to the synthesis of biquinoline-pyridine hybrids **104** [96]. The piperidine-catalyzed three-component reaction of tetrazolo[1,5-*a*]quinoline-4-carbaldehydes type **1**, *β*-pyridinyl enaminone **39** and malononitrile/methylcyanoacetate/ethylcyanoacetate (**12**)**/38** led to the corresponding biquinoline–pyridine hybrids **104** in ethanol under reflux conditions (Scheme 44). All compounds **104** were tested for in vitro anticancer activities against two cancer cell lines A549 (adenocarcinomic human alveolar basal epithelial) and Hep G2 (liver cancer). Enzyme inhibitory activities of all compounds were carried out against EGFR and HER-2 kinase. Remarkably, compound **104i** (R = H, R^1^ = CO_2_Et) displayed the most potent inhibitory activity with IC_50_ values of 0.09 µM and 0.2 µM against EGFR and HER-2 kinase, respectively.

**Scheme 45:**
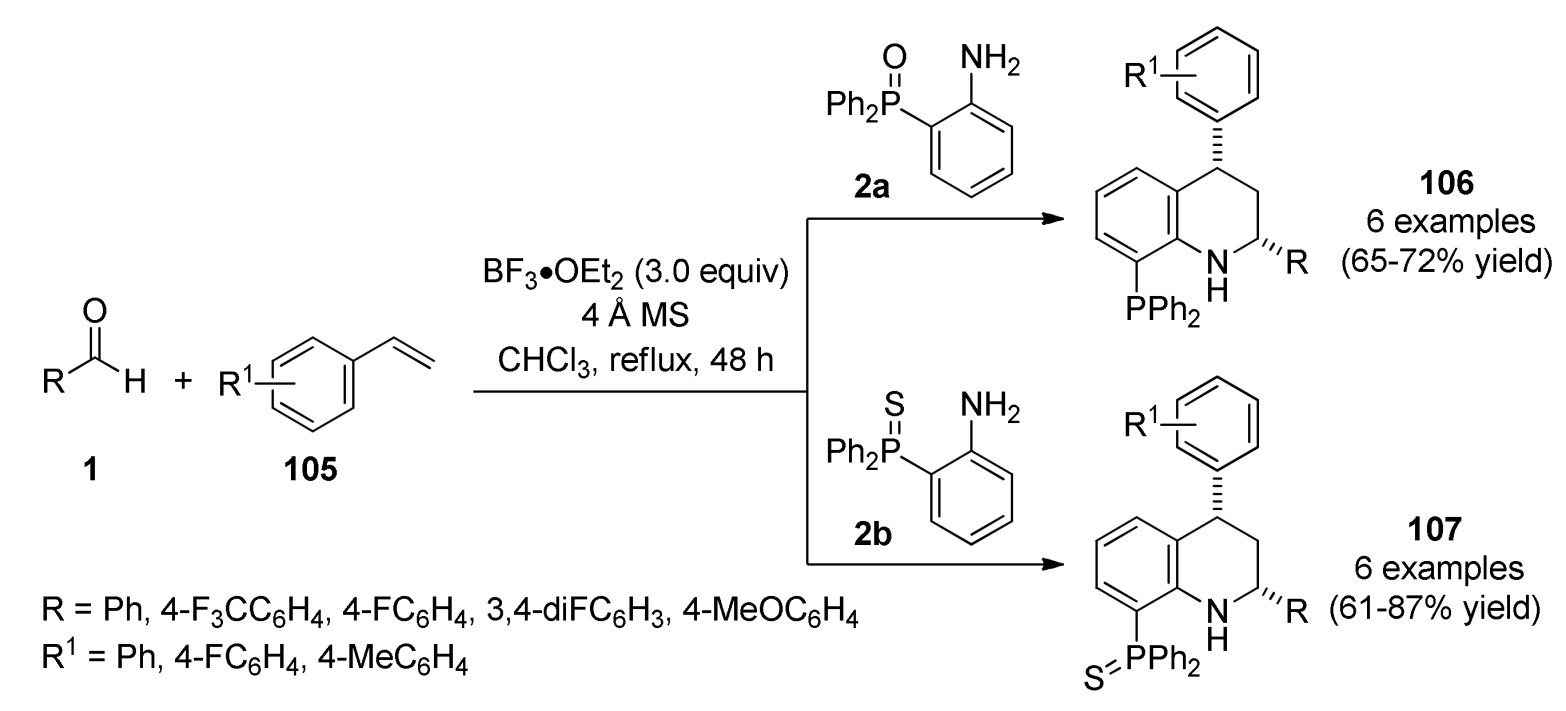
Povarov three-component reaction for the synthesis of 1,2,3,4-tetrahydroquinolinylphosphanes **106** and 1,2,3,4-tetrahydroquinolinylphosphine sulfides **107** for antitumor screening.

Alternatively, the use of an asymmetric Povarov reaction allowed Alonso et al., to synthesize 1,2,3,4-tetrahydroquinolinylphosphanes **106** through a regio- and stereoselective aza-Diels-Alder reaction from aldehydes **1**, styrenes **105** and phosphine oxide aniline **2a** in the presence of BF_3_∙OEt_2_ as Lewis acid and 4 Å molecular sieves in chloroform under reflux conditions (Scheme 45). The amount of BF_3_∙OEt_2_ (3.0 equiv) and reaction times (48 h) are relatively high without any specific explanation as to why by the authors [97]. Furthermore, the multicomponent reaction also proceeded with an aniline containing the phosphine sulfide group type **2b** to form 1,2,3,4-tetrahydro-quinolinylphosphine sulfides **107** in good yields using homogeneous acidic catalysis. The synthesized compounds were screened against three human cancer cell lines such as lung adenocarcinoma (A549), ovarian carcinoma (SKOV03) and embryonic kidney (HEK293). Notably, compound **106c** (R = 4-FC_6_H_4_, R^1^ = 4-F) with an IC_50_ value of 0.08 µM showed excellent activity against the A549 cell line, while 1,2,3,4-tetrahydroquinolinylphosphine sulfide **107f** (R = 4-FC_6_H_4_, R^1^ = 4-F) with an IC_50_ value of 0.03 µM was the most active against the A549 cell line.

**Scheme 46:**
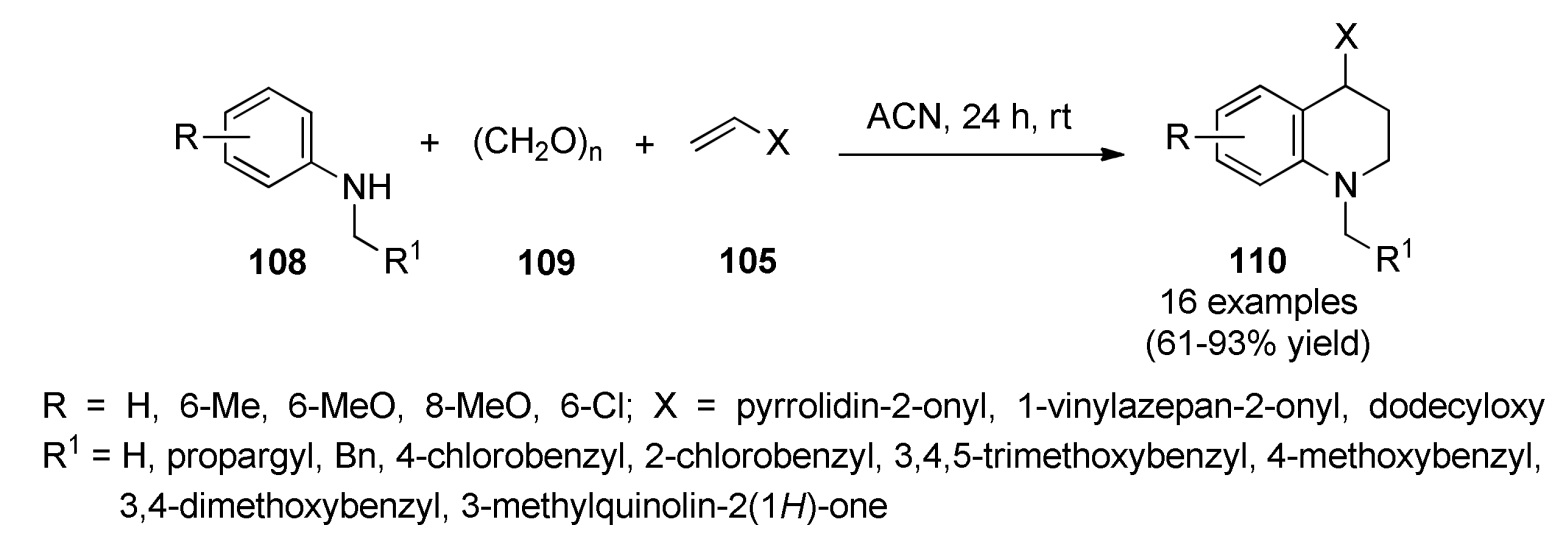
Three-component Domino-Mannich/Friedel-Crafts alkylation reaction for the synthesis of 1,2,3,4-tetrahydroquinolines **110** of anticancer interest.

In 2018, Castillo et al., described a catalyst-free method to construct diversely substituted 1,2,3,4-tetrahydroquinolines **110** through a Domino Mannich/Friedel-Crafts alkylation reaction of *N*-arylamines **108**, paraformaldehyde (**109**) and electron-rich olefins **105** in ACN at room temperature [98]. This work showed that the choice of solvent was crucial and could greatly influence the reaction course: for example, *γ*-aminoethers were observed when methanol, ethanol, *n*-propanol or *n*-butanol were used as solvent [99]. These conditions enabled the construction of a broad library of 1,2,3,4-tetrahydroquinolines **110** in good to excellent yields via the formation of *N*-aryl-*N*-alkylmethyleneiminium ions as the key intermediates (Scheme 46). Interestingly, nine of the synthesized compounds were evaluated by the U.S. National Cancer Institute (NCI), where compound **110f** (R = 6-MeO, R^1^ = 4-ClC_6_H_4_, X = pyrrolidin-2-onyl) presented a remarkable activity against 57 cancer cell lines, with the most important GI_50_ values ranging from 1.46 to 8.28 µM.

**Scheme 47:**
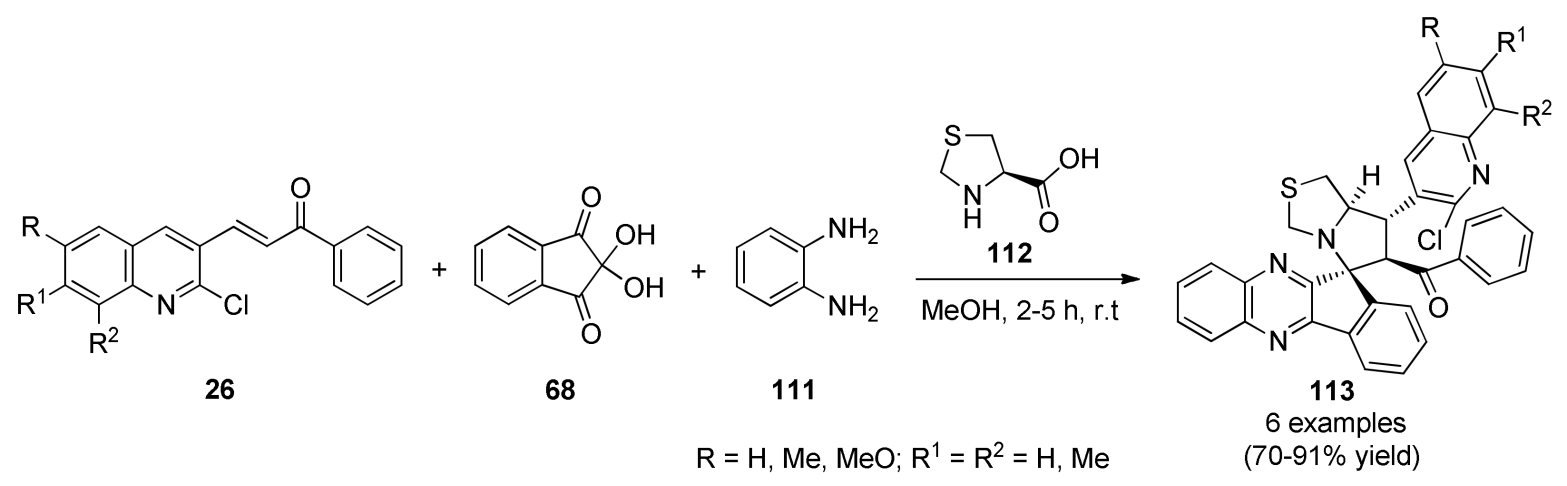
Regioselective multicomponent synthesis of spiro-indeno[1,2-*b*]quinoxalin-pyrrolothiazoles **113** for antitumor screening.

An elegant synthesis of spiro-indeno[1,2-*b*]quinoxaline pyrrolothiazoles **113** in a highly regio- and stereoselective manner was reported in 2018 by Mani et al. [100]. The use of chalcones type **26** containing quinoline moiety in combination with ninhydrin (**68**), *o*-phenylenediamine (**111**) and thiazolidine-2-carboxylic acid (**112**) led to pyrrolothiazoles **113** in good yields and short reaction times (Scheme 47). This four-component reaction proceeded through cyclocondensation of ninhydrin (**68**) and *o*-phenylenediamine (**111**) to form an indeno[1,2-*b*]quinoxalin-11-one, which further condensed with thiazolidine-2-carboxylic acid (**112**) to produce an azomethine ylide derivative. This 1,3-dipole subsequently underwent cycloaddition reaction with chalcones type **26** to obtain a small library of pyrrolothiazole derivatives **113**. The latter compounds showed interesting in vitro antitumor activity against two human cancer cell lines such as breast cancer (MCF-7) and lung adenocarcinoma (A549). Noteworthily, compound **113e** (R = Me, R^1^ = H, R^2^ = Me) showed excellent activity with IC_50_ values of 11.0 µM and 15.0 µM against MCF-7 and A-549 cell lines, respectively.

**Scheme 48:**
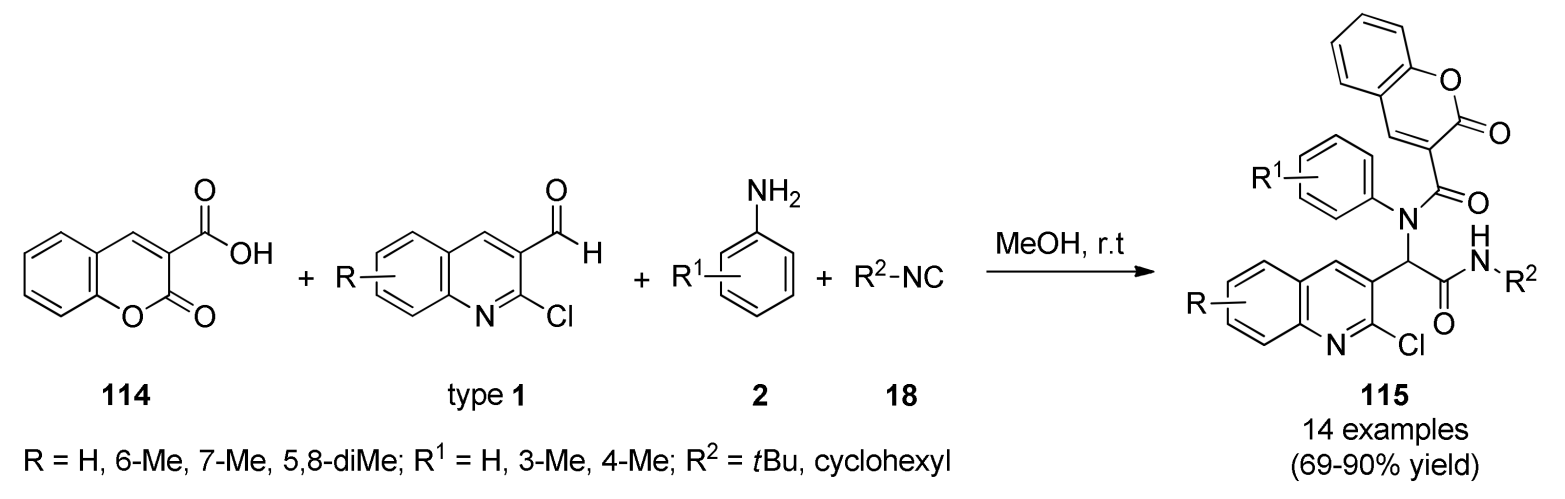
Ugi multicomponent reaction for the synthesis of quinoline-coumarin derivatives **115** to be investigated against A2780 human *Ovarian* cancer cell line.

Very recently, an Ugi four-component synthesis of quinoline-coumarin hybrids **115** was described by Taheri et al. [101]. This efficient and simple access to quinoline-coumarin derivatives involved coumarin-3-carboxylic acid (**114**), diverse 2-chloroquinoline-3-carbaldehydes **1**, aniline derivatives **2** and aliphatic isocyanides **18** in methanol at room temperature (Scheme 48). 

Cytotoxic effects of fourteen products were investigated in A2780 human ovarian cancer cells. Interestingly, compound **115k** (R = 5,8-diMe, R^1^ = H, R^2^ = cyclohexyl) showed excellent anticancer activity with IC_50_ values of 0.042 µM. Furthermore, the treatment of A2780 cells with compound **115k** significantly (*p*-value ≤ 0.05) induced apoptosis by down-regulation of Bcl-2 and survivin both in mRNA and protein level via a single dose (0.042 µM), as well as, activation of caspase 9 and 3, loss of mitochondrial membrane potential (MMP), and high reactive oxygen species (ROS).

#### 3.13.2. Pyrazole Derivatives

**Scheme 49:**
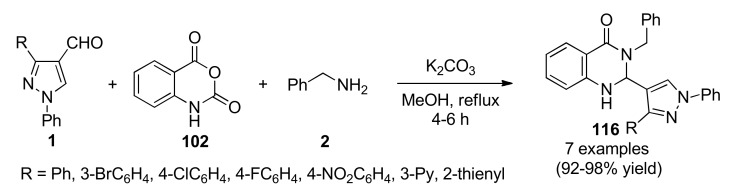
Three-component synthesis of pyrazol-4-yl-2,3-dihydroquinazolin-4(1*H*)-ones **116** for in vitro screening by the US National Cancer Institute (NCI) against 60 human tumor cell lines.

Pyrazole is a heteroaromatic compound of 5-membered containing two adjacent nitrogen atoms. The wide range of biological and synthetic applications displayed by pyrazoles, as well as, by their fused heterocyclic systems has been well documented in several comprehensive reviews [102,103,104,105,106]. In particular, formylpyrazoles occupy a noticeable place in the field of organic and medicinal chemistry despite somewhat being less popular than the amino derivatives, since such heteroaryl aldehydes are key intermediates for obtaining a wide range of biologically active compounds. In 2017, Kamble et al., employed a series of 3-(substituted)-1-phenyl-1*H*-pyrazole-4-carbaldehydes **1** in combination with isatoic anhydride (**102**) and benzylamine (**2**) in a K_2_CO_3_-mediated process conducted under reflux conditions in methanol (Scheme 49). This three-component reaction resulted in the synthesis of pyrazol-4-yl-2,3-dihydroquinazolin-4(1*H*)-ones **116** in high yields and short reaction times [107]. Compounds **116a** (R = Ph), **116d** (R = 4-FC_6_H_4_), **116f** (R = 3-Py) and **116g** (R = 2-thienyl) were in vitro screened by the NCI against 60 human tumor cell lines at a single dose of 10 µM. Results for each tested compound were reported as a mean graph of the percentage growth inhibition (%GI) of the treated cells, and deliberated by comparing with untreated control cells as percentage of growth inhibition (%GI) over all the tested cell lines. Remarkably, compound **116d** exhibited the highest activity being breast cancer MCF7 (%GI 66.73), leukemia MOLT-4 (%GI 60.28), prostate cancer PC-3 (%GI 59.04), renal cancer UO-31 (%GI 57.83), non-small cell lung cancer EKVX (%GI 54.20) and leukemia HL-60 (TB) (%GI 54.03) the most sensitive strains. In addition, compound **116a** exhibited good activity being renal cancer UO-31 (%GI 51.80), breast cancer T47D (%GI 50.85), prostate cancer PC-3 (%GI 45.38) and ovarian cancer IGROV1 (%GI 44.57) the most sensitive strains.

**Scheme 50:**
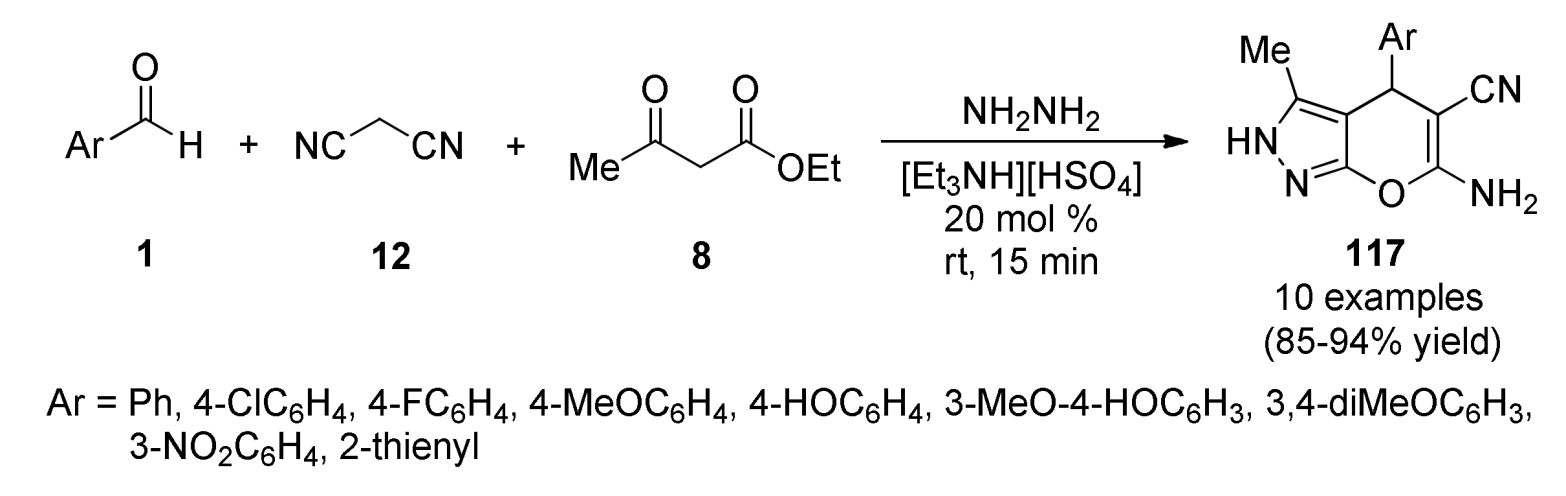
Ionic liquid-catalyzed four-component synthesis of dihydropyrano[2,3-*c*]pyrazoles **117** for anticancer evaluation.

Nikalje’s group developed an efficient and green synthetic protocol to prepare 6-amino-4-substituted-3-methyl-2,4-dihydropyrano[2,3-*c*]pyrazole-5-carbonitriles **117** by four-component condensation reactions of various aromatic aldehydes **1**, malononitrile (**12**), hydrazine hydrate and ethyl acetoacetate (**8**) using the ionic liquid triethylammonium hydrogen sulphate [Et_3_NH][HSO_4_] as a green reaction medium and also as a catalyst (Scheme 50). The fused heterocyclic products were isolated in high yields after short reaction times [108]. It is noteworthy that the catalyst was reused up to 4 times without much loss of catalytic activity. The in vitro anticancer activity of the synthesized compounds was carried out by the sulforhodamine B (SRB) assay against four human cancer cell lines including melanoma (SK-MEL-2), breast (MDA-MB-231), leukemia (K-562) and cervix (HeLa). The results indicated that compounds **117b** (Ar = 4-ClC_6_H_4_), **117d** (Ar = 4-MeOC_6_H_4_), **117g** (Ar = 3,4-diMeOC_6_H_3_) and **117h** (Ar = 3-NO_2_C_6_H_4_) exhibited significant cancer cell growth inhibition against the SK-MEL-2, MDA-MB-231 and K-562 cancer cell lines. Notably, compound **117b** containing one chlorine atom in the *para* position of the phenyl ring exhibited excellent in vitro anticancer activity against the SK-MEL-2, MDA-MB-231 and K-562 cell lines with GI_50_ concentrations of 0.1 µM, 0.74 µM and 11.20 µM, respectively.

#### 3.13.3. Curcumin Derivatives

Curcumin (diferuloylmethane or 1,7-bis(4-hydroxy-3-methoxyphenyl)-1,6-heptadiene-3,5-dione) is an edible natural pigment extracted from *Curcuma longa* [109]. The therapeutic activity of curcumin has been widely investigated over the last few decades and reports suggest the role of curcumin in innumerable biological activities, particularly its prominent in vitro and in vivo anticancer activity [109,110]. However, multiple structural-pharmacokinetic challenges such as low solubility and poor bioavailability during oral administration greatly limit its clinical application. Albeit, several strategies have been developed to overcome these disadvantages, one of the most common is the synthesis of novel curcumin derivatives that have better therapeutic properties and bioavailability [111,112]. From the viewpoint of the organic chemistry, curcumin exists in solution as a tautomeric keto form in acidic and neutral solutions while enol form in the alkaline medium. Furthermore, it has both an electrophilic Michael acceptor and an active methylene Michael donor units. In that direction, a series of structurally diverse 4-dihydropyrimidin-2(*H*)-one/thione derivatives of curcumin **7**, as shown in Scheme 2, were synthesized through a three-component reaction involving curcumin (**5**), substituted aromatic aldehydes **1**, and urea/thiourea **6** via a Biginelli type reaction in ethanol and concentrated sulphuric acid under reflux conditions [113]. Compounds **7b** (Ar = 4-MeOC_6_H_4_, X = O), **7d** (Ar = 4-ClC_6_H_4_, X = O) and **7g** (Ar = 3-MeO-4-OH C_6_H_3_, X = O) were in vitro screened by the NCI against 60 human tumor cell lines at a single dose of 10 µM. Remarkably, compound **7d** showed the highest activity, being leukemia CCRF-CEM (%GI 78.55), renal cancer UO-31 (%GI 75.92), prostate cancer PC-3 (%GI 75.87), central nervous system (*CNS*) SNB-75 (%GI 75.49), breast cancer MDA-MB-231 (%GI 73.63) and leukemia MOLT-4 (%GI 73.57) the most sensitive strains. In addition, compound **7g** exhibited good activity being prostate cancer PC-3 (%GI 76.71), colon cancer HCT-116 (%GI 73.27), leukemia MOLT-4 (%GI 70.26), breast cancer MDA-MB-231 (%GI 67.70) and central nervous system (CNS) SNB-75 (%GI 65.81) the most sensitive strains.

**Scheme 51:**
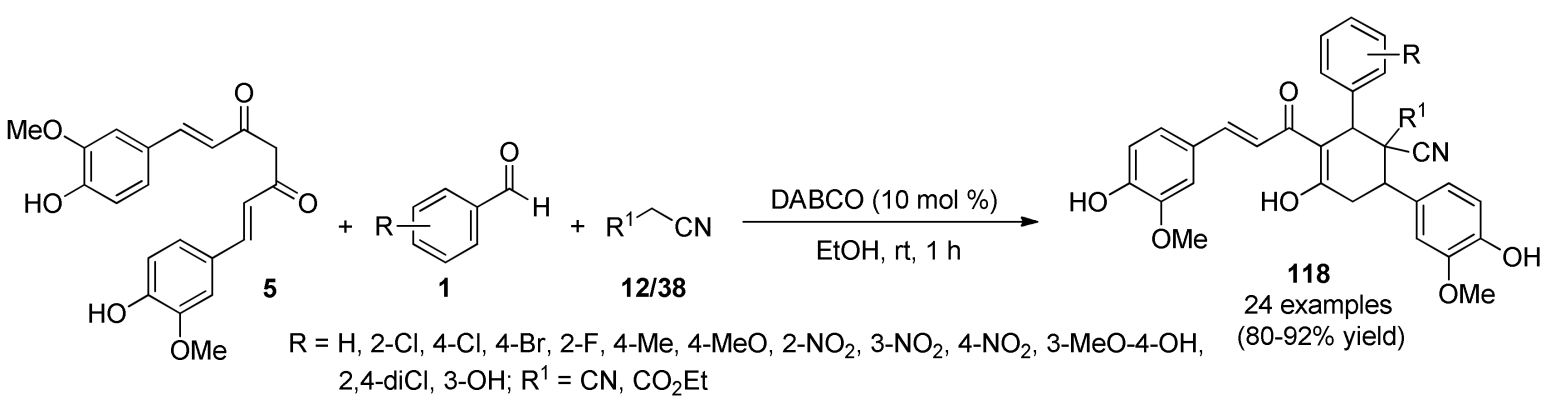
DABCO-catalyzed three-component synthesis of functionalized curcumin derivatives **118** of anticancer interest.

Bhuvaneswari et al., reported that 1,4-diazabicyclo[2.2.2]octane (DABCO) catalyzed a three-component reaction of curcumin (**5**), substituted aromatic aldehydes **1** and malononitrile/ethyl 2-cyanoacetate (**12**)**/**(**38**) in ethanol at room temperature to afford functionalized curcumin derivatives **118** in 80−92% yield, Scheme 51. The construction of the cyclohexene ring proceeded through a Knoevenagel/Michael/cyclization sequence catalyzed by DABCO (10 mol%) [114]. Compounds **118j** (R = 4-Me, R^1^ = CO_2_Et) and **118k** (R = 2-NO_2_, R^1^ = CO_2_Et) were screened for their in vitro antitumor activity against human breast cancer cells (MCF-7) using the MTT assay. Compound **118j** showed excellent activity with IC_50_ value of 10.0 µM against human breast cancer cells (MCF-7). In addition, molecular docking studies allowed rationalizing the anti-apoptotic Bcl-2 binding of all synthesized compounds and revealed that the docking of compounds **118j** and **118k** with Bcl-2 was more potent compared to curcumin **5**.

#### 3.13.4. Pyrrole Derivatives

**Scheme 52:**
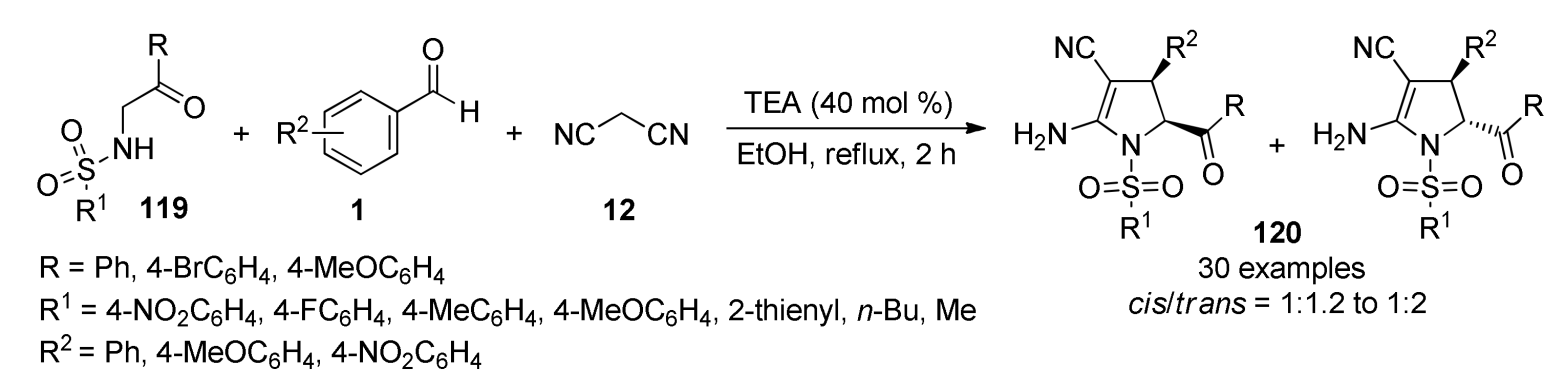
Three-component synthesis of highly functionalized 2-pyrrolines **120** for anticancer evaluation.

Pyrrole is one of the most relevant *N*-heterocyclic unit because of its presence in diverse natural and synthetic compounds with a broad range of applications in medicinal chemistry, drug discovery and materials science [115,116,117]. Although, several synthetic approaches for this scaffold have been developed, there remains a great need to find simpler and atom-economical approaches for the construction of functionalized pyrrole-fused derivatives, which have been considered of interest in view of their pharmacological importance [118,119,120]. In this sense, Magedov et al., employed a series of *N*-(aryl- and alkylsulfonamido)acetophenones **119** in combination with diverse aromatic aldehydes **1** and malonitrile (**12**) in a TEA-catalyzed three-component procedure in ethanol under reflux conditions to afford highly functionalized 2-pyrrolines **120** in good yields, high regioselectivity and short reaction times (Scheme 52) [121]. However, the poor diastereoselectivity generated a mixture of *cis*- and *trans*-2-pyrrolines in 1:1.2 to 1:2 ratio. Rationalization of the low diastereoselectivity was not discussed. The synthesized compounds **120** were screened against two human cancer cell lines such as cervical adenocarcinoma (HeLa) and breast cancer (MCF-7). Because of the labor-intensive separation of the stereoisomeric pyrroline mixtures, the tests were performed with these mixtures. Remarkably, compound **120m** (R = Ph, R^1^ = 4-MeOC_6_H_5_, R^2^ = Ph) showed the most potent inhibitory activity with GI_50_ values of 36.0 µM and 50.0 µM against the HeLa and MCF-7 cell lines, respectively.

**Scheme 53:**
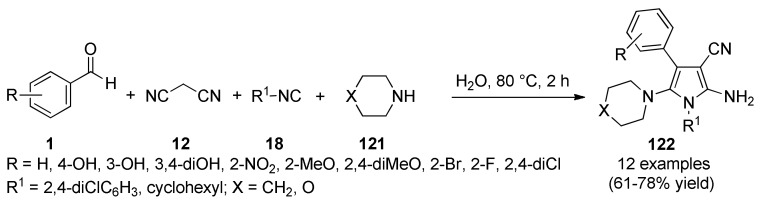
Multicomponent synthesis of fully substituted pyrrole-3-carbonitriles **122** in aqueous medium to be screened for their antitumoral activity against breast (MCF-7) and colon (HT-29) human cancer cell lines.

As originally described by Pagadala [122], highly functionalized pyrroles **122** are also available by means of a catalyst-free four-component reaction in aqueous medium. This green transformation involved an aromatic aldehyde **1**, malononitrile (**12**), an isocyanide **18** and a cyclic secondary amine **121**, allowing an efficient access to polysubstituted pyrroles **122** in good yields (Scheme 53). It is noteworthy that aromatic aldehydes **1** were successfully employed while aliphatic aldehydes such as acetaldehyde and *n*-butyraldehyde failed to provide the corresponding polysubstituted pyrrole. Easy accessibility of starting materials, short reaction times and catalyst-free reaction, as well as, the use of water as solvent are claimed as the key advantages of this procedure. The synthesized compounds **122** were screened for their antitumoral activity against breast (MCF-7) and colon (HT-29) human cancer cell lines using doxorubicin and cisplatin as reference standards. Notably, compound **122g** (R = 2,4-diMeOC_6_H_3_, R^1^ = 3,4-diClC_6_H_3_, X = O) with an IC_50_ value of 1.24 µM showed the highest activity against the MCF-7 cell line, while compound **122h** (R = 2-BrC_6_H_4_, R^1^ = 3,4-diClC_6_H_3_, X = O) with an IC_50_ value of 1.47 µM was the most active against the HT-29 cell line.

#### 3.13.5. Chromone and Chromene Derivatives

**Scheme 54:**
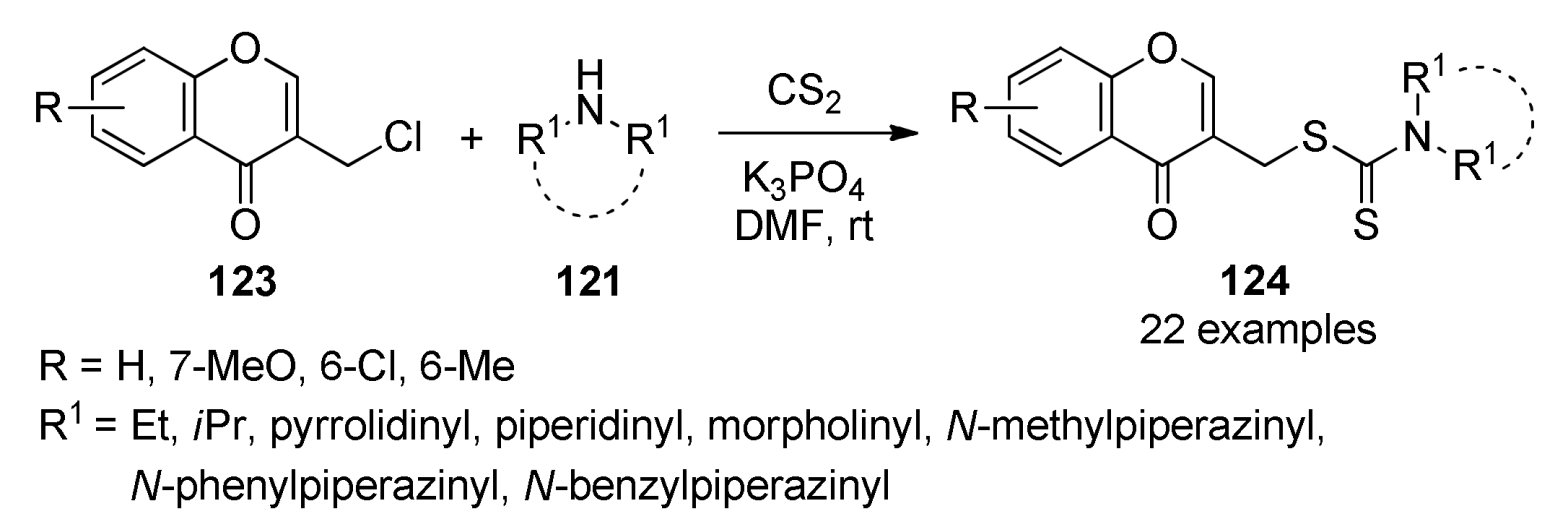
Three-component synthesis of dithiocarbamate substituted chromones **124** to be screened for their in vitro antiproliferative activity by mean of the MTT method.

The oxygen-containing heterocycles are an important class of compounds in organic chemistry. The fusion of an aromatic ring to an oxygen-containing heterocycle will alter the electron density; thereby, the physical, chemical and biological properties will change. In particular, chromone is an oxygen-containing heterocyclic system with a benzoannelated *γ*-pyrone ring being chromone (4*H*-chromen-4-one, 4*H*-1-benzopyran-4-one) the parent compound [123,124]. Furthermore, bicyclic oxygen-containing heterocycles resulting from fusion of benzene ring with 5,6-positions of either 2*H*- or 4*H*-pyran ring system are designated as 2*H*-chromene and 4*H*-chromene, respectively. Hence, the structural importance of the chromone and chromene moiety has elicited a great deal of interest in the field of organic synthesis, medicinal chemistry and drug discovery to develop novel and improved synthesis of these molecular skeletons [125,126,127]. In this sense, Huang et al., described a catalyst-free access to dithiocarbamate substituted chromones **124** by means of a three-component reaction (Scheme 54) [128]. A mixture of 3-chloromethyl chromone derivatives **123**, cyclic secondary amines **121** and an excess of carbon disulfide was stirred at room temperature in DMF affording compounds **124** in good yields. The synthesized compounds were screened for their in vitro antiproliferative activity against six cancer cell lines, including HCCLM-7 (hepatocellular carcinoma cell), He-La (cervical carcinoma cell), MDA-MB-435S (mammary adenocarcinoma cell), SW-480 (colon carcinoma cell), Hep-2 (laryngocarcinoma cell) and MCF-7 (mammary adenocarcinoma cell) by using the MTT method. It should be noted that compound **124u** (R = 6-Cl, R^1^ = piperidinyl) was identified as the most promising candidate due to their high potency against all cancer cell lines with IC_50_ values ranging from 0.24 to 0.85 µM. Further flow-activated cell sorting analysis revealed that compound **124u** arrest the cell cycle of MDA-MB-435S and SW-480 both in G_2_/M phase with dose-dependent effect and might display apoptosis-inducing effect on these tumor cell lines.

**Scheme 55:**
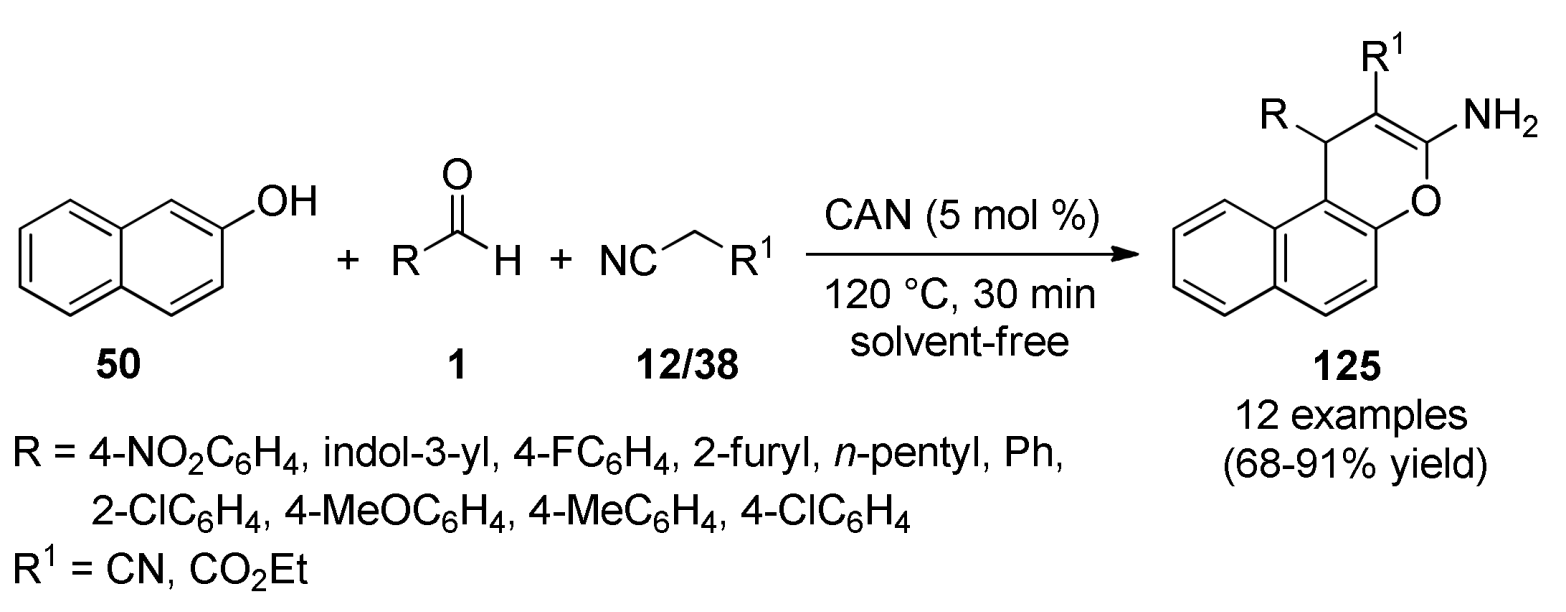
CAN-Catalyzed three-component synthesis of 3-amino-1-substituted-1*H*-benzo[*f*]chromenes **125** under solvent-free conditions for evaluation of their antiproliferative activity.

In 2010, Kumar et al., reported an intriguing three-component synthesis of benzochromenes **125** in good yields by mixing 2-naphthol (**50**), an aldehyde **1** and malononitrile/ethyl cyanoacetate (**12**)**/**(**38**) in the presence of a catalytic amount of ceric ammonium nitrate (CAN) under solvent-free conditions (Scheme 55) [129]. Great functional diversity was achieved with this thoughtful strategy that accommodated aromatic, heteroaromatic, and aliphatic aldehydes. The advantages of this method include the use of a recyclable catalyst, short reaction times, simple work-up procedure and easy isolation. From a mechanistic standpoint, a sequence initiated by the formation of an *ortho*-quinonemethide through the nucleophilic addition of 2-naphthol to the aldehyde catalyzed by CAN. Then, Michael addition of malononitrile or ethyl cyanoacetate onto the *ortho*-quinonemethide, followed by intramolecular 6-*exo*-*dig* cyclization/imine-enamine tautomerization sequence generated the benzochromene ring. The synthesized compounds **125** were screened for their antiproliferative activity in prostat*e* cancer (DU-145), breast cancer (MCF-7), cervical carcinoma (C-33A) and lung carcinoma (A-549) human cell lines. It is noteworthy that compound **125b** (R = indol-3-yl, R^1^ = CN) exhibited the highest activity against all cancer cell lines with IC_50_ values ranging from 5.4 to 12.2 µM.

**Scheme 56:**
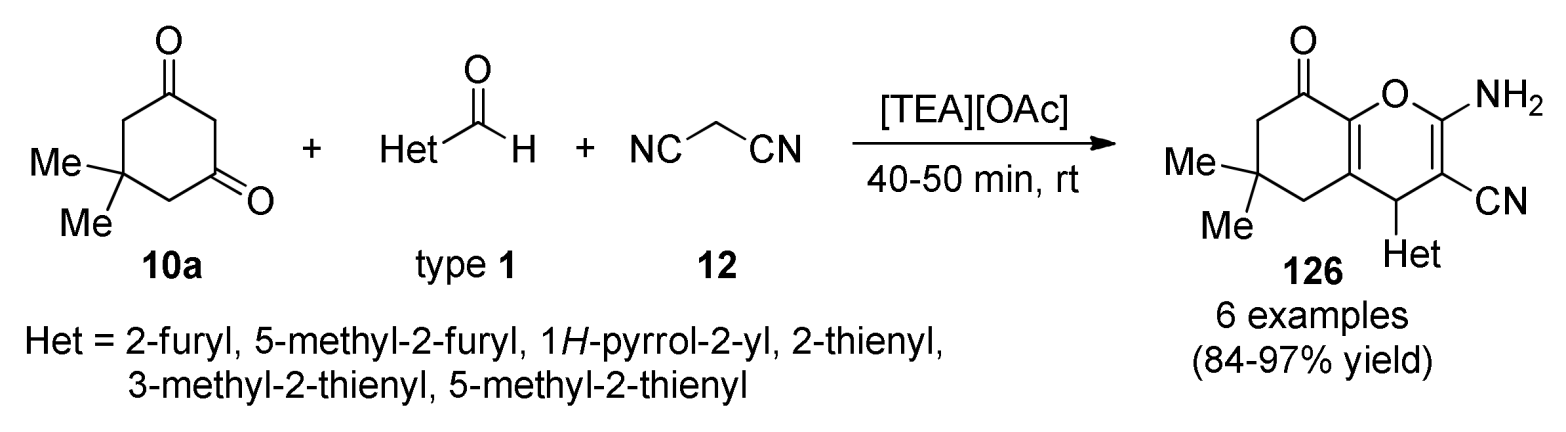
TEAA-promoted three-component synthesis of 5,6,7,8-tetrahydro-4*H*-chromene-3-carbonitriles **126** of antitumor interest.

Shortly after, the Paliwal´s group reported the three-component reaction between dimedone (**10a**) an heteroaryl aldehyde e **1** and malononitrile (**12**) at room temperature in the ionic liquid (IL) [TEA][OAc] (TEAA), gave rise to a series of 5,6,7,8-tetrahydro-4*H*-chromene-3-carbonitriles **126** in good yields (Scheme 56) [130]. According to the authors, [TEA][OAc] acted as a green catalyst as well as a reusable solvent. Use of IL media for this transformation resulted in improved yields within shorter reaction times. A complementary study on these latter conditions suggested that IL could be reused for six times without apparent loss of catalytic activity. The synthesized compounds **126** were screened for their in vitro antitumor activity in breast cancer (MDA-MB-435), prostate cancer (PC-3) and ovarian cancer (Ovkar-3) human cell lines. However, the synthesized compounds did not give the satisfactory results between 10^−7^ and 10^−4^ molar concentrations in comparison with doxorubicin.

**Scheme 57:**
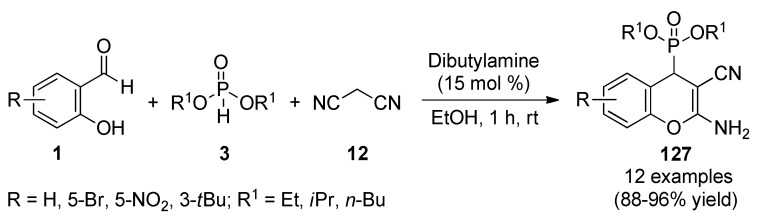
Three-component synthesis of 2-amino-3-cyano-4*H*-chromen-4-ylphosphonates **127** to be screened against two cancer cell lines using MTT assay.

An alternative access to chromenyl phosphonates from dialkylphosphites was reported in 2014 [131]. Thus, an ethanolic solution of a substituted salicylaldehyde type **1**, a dialkylphosphite type **3** and malononitrile (**12**) was stirred at room temperature in the presence of a catalytic amount of dibutylamine, afforded the 2-amino-3-cyano-4*H*-chromen-4-ylphosphonates **127** in very good yields, (Scheme 57). 

The ready availability of this non-toxic catalyst, high yields, and simple work-up make this approach an eco-friendly alternative to the currently existing protocols. The synthesized compounds **127** were screened against two cancer cell lines, including adenocarcinomic alveolar basal epithelial (A-549) and epidermoid cancer (KB) using MTT assay. Albeit all synthesized compounds showed moderate activity at 20 and 40 µM concentrations, compounds **127a** (R = H, R^1^ = Et) and **127d** (R = 6-Br, R^1^ = Et) showed remarkable activity against the two tested cell lines.

**Scheme 58:**
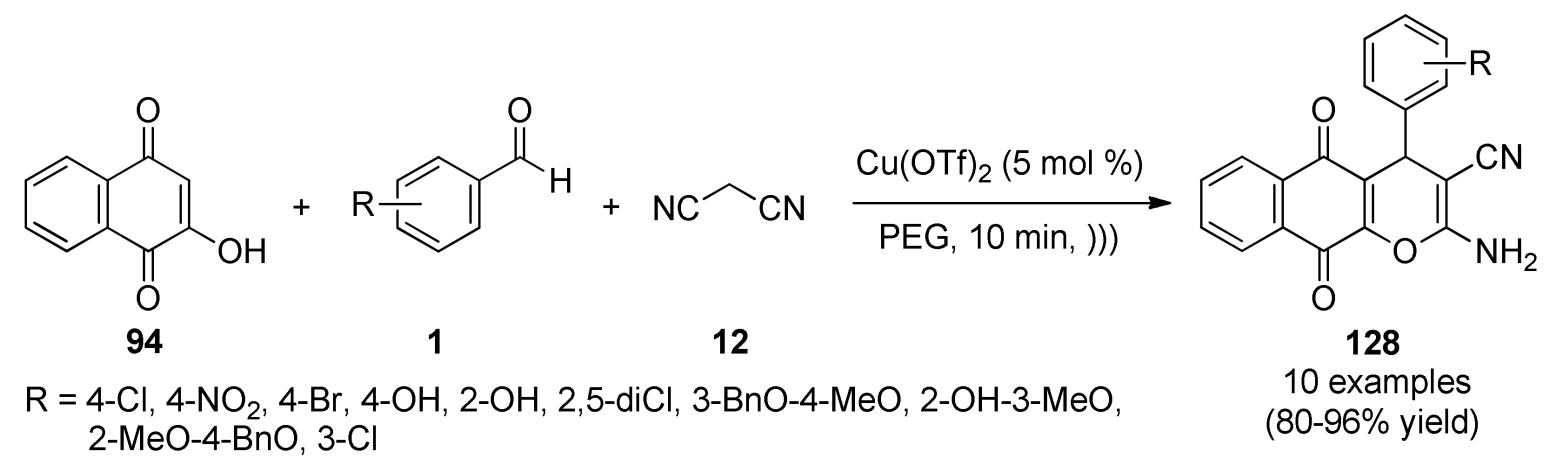
Cu(OTf)_2_-Catalyzed three-component synthesis of benzo[*g*]chromenes **128** under ultrasound irradiation for testing of their in vitro anticancer activity against cervical cancer cell line (HeLa).

Perumal et al., reported an ultrasound-assisted three-component reaction of 2-hydroxy-1,4-naphthoquinone **94**, an aromatic aldehyde **1** and malononitrile **12** in the presence of Cu(OTf)_2_ catalyst and eco-friendly polyethylene glycol solvent, thus giving access to highly functionalized benzo[*g*]chromene derivatives **128** in good yields and short reaction times (Scheme 58) [132]. The synthesized compounds **128** were tested for their in vitro anticancer activity against cervical cancer cell line (HeLa). Most of the compounds showed higher antitumor activity that doxorubicin. Particularly, compounds **128c** (R = 4-Br), **128g** (R = 3-benzyloxy-4-MeO), **128h** (R = 2-hydroxy-3-MeO) and **128j** (R = 3-Cl) displayed the highest activity with IC_50_ values ranging from 1.2 to 4.1 µM.

#### 3.13.6. Pyridone Derivatives

**Scheme 59:**
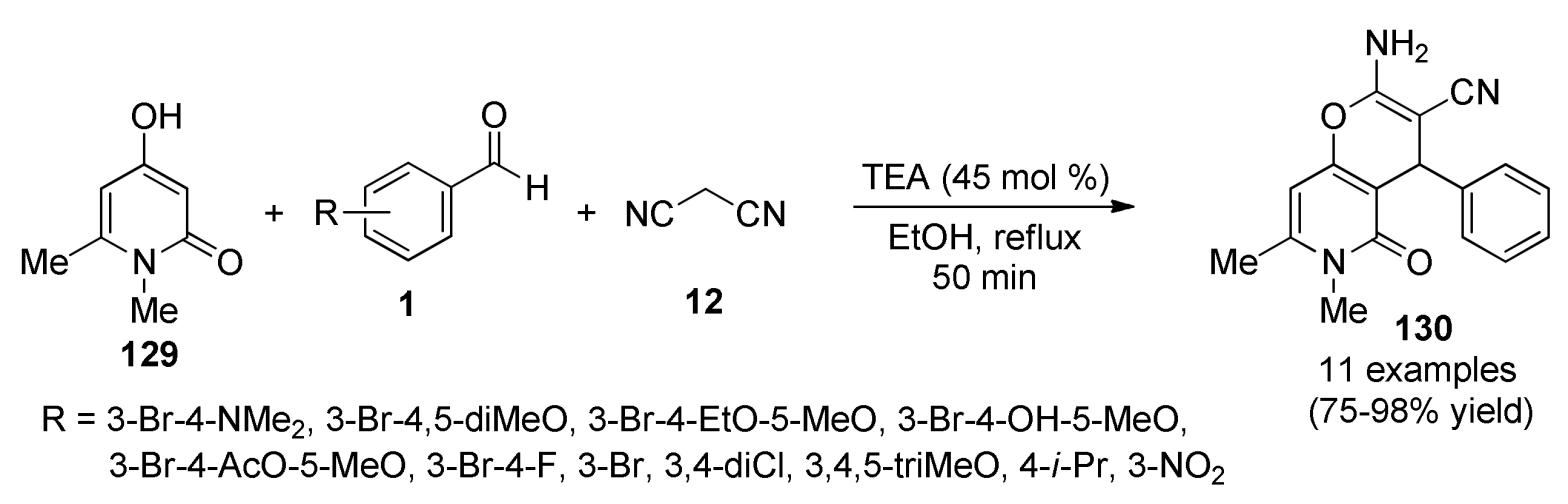
Three-component synthesis of pyrano[3,2-*c*]pyridone derivatives **130** for antiproliferative assays.

2-Pyridone, the tautomer of 2-hydroxypyridine, is one of the privileged heteroaromatic rings in natural products, bioactive molecules and pharmaceutical agents [133,134]. Furthermore, pyridone fused with a benzene or heterocyclic ring gives rise to diverse heterocyclic systems with innumerable pharmacological properties. Thus, the selective synthesis of functionalized 2-pyridone derivatives and fused-pyridone heterocycles has been one of the important longstanding subjects in organic synthetic chemistry [135,136,137]. In that direction, a series of highly functionalized pyrano[3,2-*c*]pyridones **130** were synthesized through a TEA-catalyzed three-component reaction of 4-hydroxy-1,6-dimethylpyridin-2(1*H*)-one (**129**), an aromatic aldehyde **1** and malononitrile (**12**) in ethanol under reflux conditions (Scheme 59) [138]. Annexin-V staining and DNA laddering assays.

The biheterocyclic products **129** were isolated in high yields after short reaction times. The obtained compounds **129** were screened for their in vitro antitumor activity against cervical cancer cell line (HeLa). Notably, compounds exhibited submicromolar or low micromolar potencies for the inhibition of proliferation of HeLa cells with GI_50_ values ranging from 0.33 to 43.3 µM. Furthermore, the antiproliferative effect from the potent apoptosis inducing ability of these heterocycles, was confirmed by the flow cytometric 

**Scheme 60:**
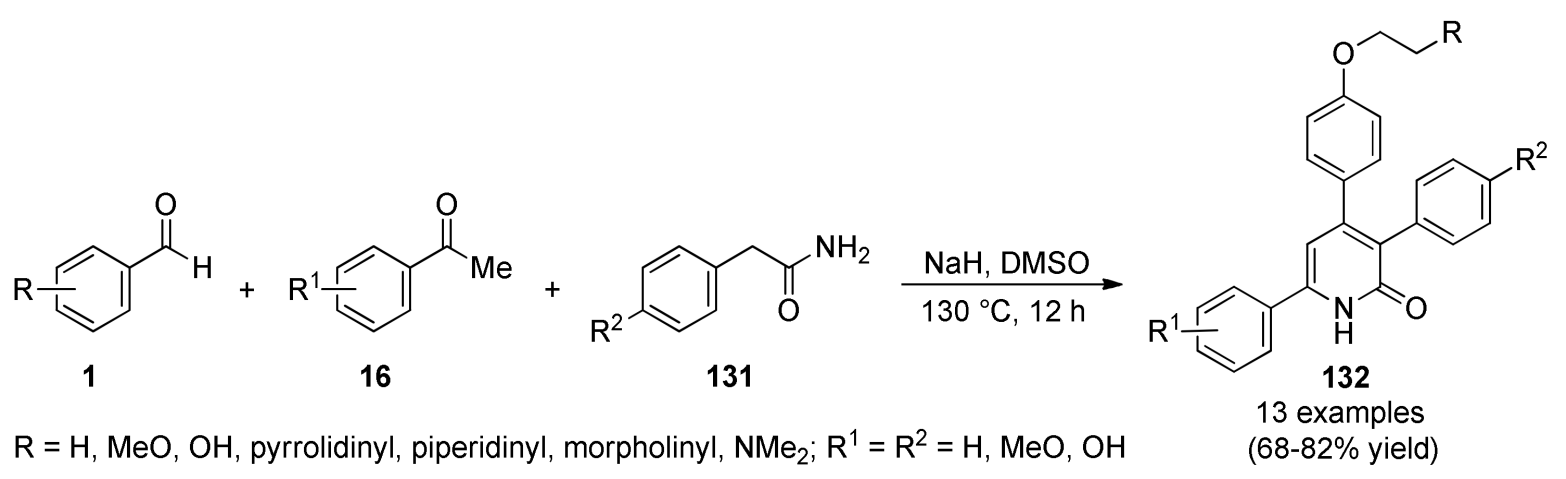
Three-component synthesis of 3,4,6-triaryl-2-pyridones **132** for antiproliferative activity against human *Breast* carcinoma cell line.

Within their research efforts, the Hajela group reported the regioselective synthesis of densely substituted 2-pyridones **132** through a three-component reaction of aromatic aldehydes **1**, substituted acetophenones **16** and phenyl acetamides **131** in the presence of sodium hydride in DMSO to afford the corresponding 3,4,6-triaryl-2-pyridones **132** in good yields as a single regioisomer (Scheme 60) [139]. The obtained compounds **132** were evaluated for their antiproliferative activity against human breast carcinoma cell line MCF-7 and MDA-MB-231. Thus, compound **132h** (R = 3,4-diMeO, R^1^ = 4-MeO, R^2^ = H) displayed the highest anti-breast cancer activity with IC_50_ values of 90.5 µM and 15.1 µM against MCF-7 and MDA-MB-231 cell line, respectively. In addition, cell cycle analysis showed that compound **132h** induced statistically significant arrest of cells in G1 phase and reduction in S-phase cells in a dose-dependent manner.

**Scheme 61:**
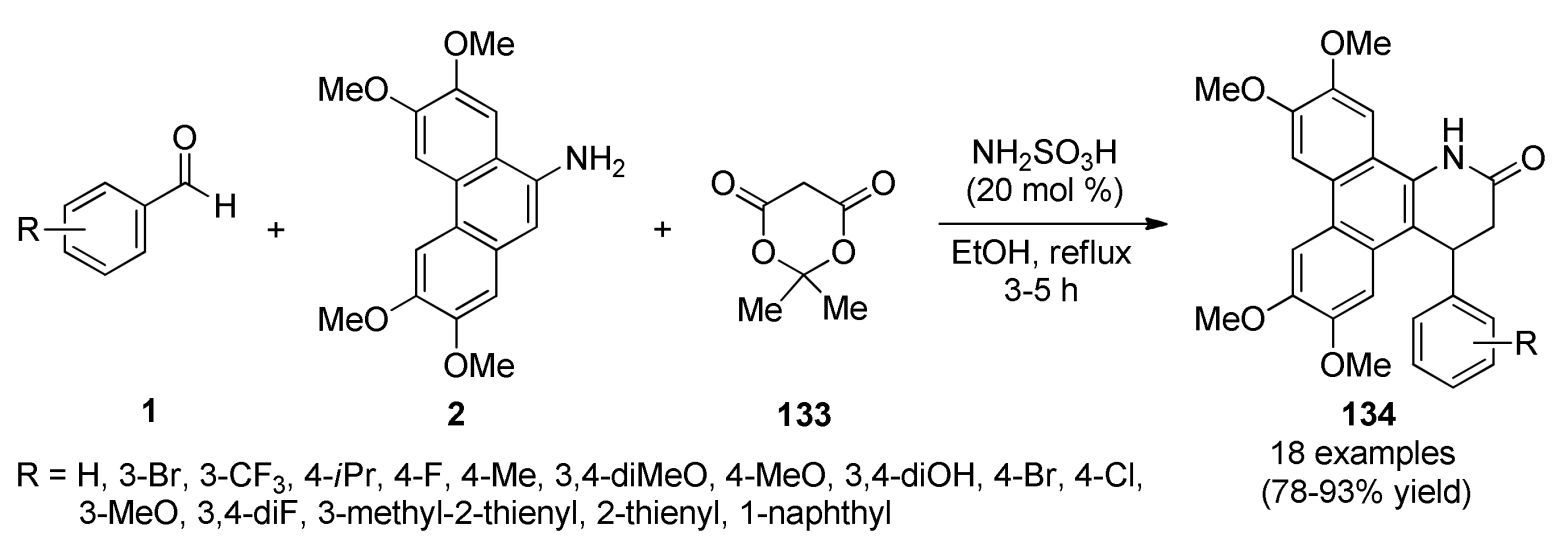
Sulfamic acid-catalyzed three-component synthesis of 3,4-dihydrodibenzo[*f*,*h*]quinolin-2(1*H*)-ones **134** for evaluation of their in vitro cytotoxic potential.

Alternatively, Kumar et al., described a three-component synthesis of quinolinone derivatives **134** using diverse aromatic aldehydes **1**, 2,3,6,7-tetramethoxyphenanthren-9-amine (**2**) and Meldrum’s acid (**133**) (Scheme 61) [140]. As a result, a collection of 3,4-dihydrodibenzo[*f*,*h*]quinolin-2(1*H*)-ones **134** was elaborated in the presence of a catalytic amount of sulfamic acid in refluxing ethanol. The authors demonstrated that the mechanism consisted of the preliminary Knoevenagel condensation of Meldrum’s acid with the aldehyde, affording the arylidene intermediate. Then, Michael addition of amine and subsequent dehydration, cyclization by loss of CO_2_ and acetone gave quinolinone derivatives **134**. The obtained compounds were evaluated for their in vitro cytotoxic potential against human lung (A549), prostate (PC-3 and DU145), breast (MCF-7) and colo*n* (HT-29 and HCT-116) cancer cell lines. Notably, compound **134p** (R = 3,4-OCH_2_O) showed excellent antiproliferative activity against A549 lung cancer cell line with an IC_50_ of 3.17 µM. Flow cytometric analyses revealed that compound **134p** arrested both Sub G1 and G2/M phases of cell cycle in a dose dependent manner. Additionally, compound **134p** also displayed significant inhibition of tubulin polymerization and disruption of microtubule network with an IC_50_ of 5.15 µM.

#### 3.13.7. Thiazole Derivatives

**Scheme 62:**
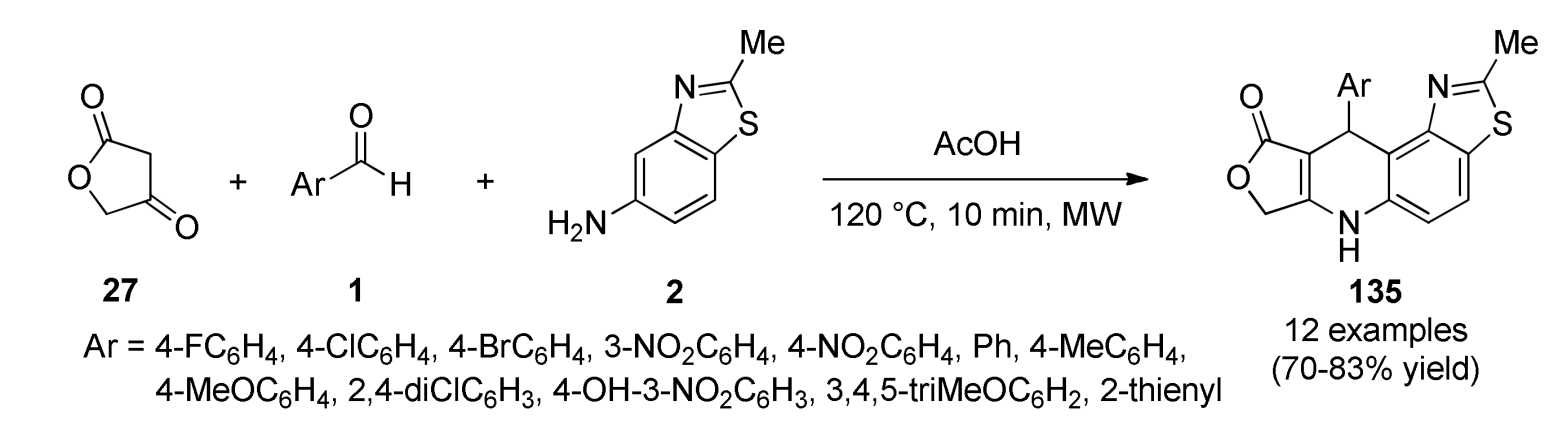
Microwave-assisted three-component synthesis of 4-aza-podophyllotoxin analogs **135** for testing of their in vitro cytotoxicity.

*S*-Heterocycles have maintained their status as an important nucleus with high medicinal value and low toxicity profile, in comparison to previous *N*-heterocycles. Particularly, thiazole has been widely found in diverse pharmacologically active substances and some naturally-occurring compounds [141]. For that reason, thiazole is a versatile building-block for the preparation of thiazole-based compounds with innumerable biological properties [142]. Thus, thiazole derivatives possessing anticancer activity will reignite the interest of the scientific community in the usefulness of these *S*-heterocycles in the medicinal chemistry and drug discovery [143,144]. In this context, tetronic acid type **27** was used as the 1,3-dicarbonyl parent in a straightforward construction of the 4-aza-podophyllotoxin skeleton embedded with a thiazole unit [145]. Thus, tetronic acid (**27**) reacted with aromatic aldehydes **1** and 2-methylbenzo[*d*]thiazol-5-amine (**2**) at 120 °C in acetic acid under MWI, leading to a series of novel 4-aza-podophyllotoxin analogs **135** (Scheme 62). The triheterocyclic products **135** were isolated in high yields after short reaction times. Then, some selected compounds were subject to the test of in vitro cytotoxicity to malignant melanin carcinoma cell line M14, mammary carcinoma cell line MCF-7, and colon carcinoma cell line SW1116. Interestingly, the 4-aza-podophyllotoxin analog **135j** (Ar = 4-OH-3-NO_2_C_6_H_3_) displayed the most potent inhibitory activity with IC_50_ values of 33.9, 56.0 and 69.4 µM against M14, MCF-7 and SW1116 human cell lines, respectively.

**Scheme 63:**
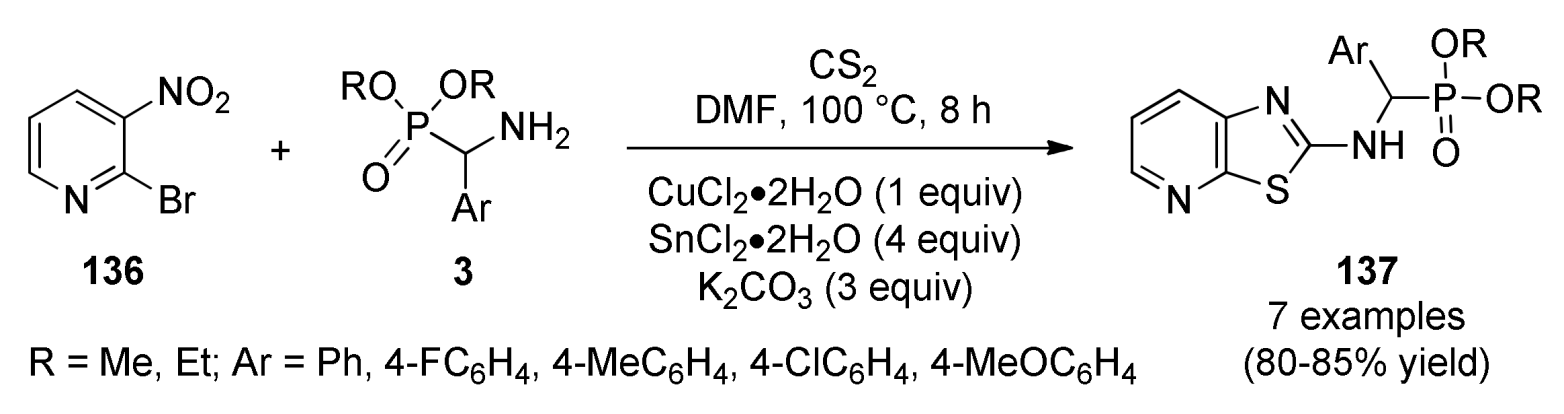
Three-component synthesis of α-aminophosphonate derivatives **137** to be assayed against PC-3, Bcap-37 and H460 human cancer cell lines by the MTT method.

Combining two potentially bioactive moieties to form heterocyclic scaffolds is a known process in drug discovery. In 2012, Gu et al., reported an impressive three-component reaction of 3-nitro-2-bromopyridine **136**, primary 1-aminophosphonates **3** and carbon disulfide in the presence of copper(II) chloride (1 equiv.), tin(II) chloride (4 equiv.) and potassium carbonate (3 equiv.) at 100 °C in DMF, leading to α-aminophosphonates **137** containing thiazole[5,4-*b*]pyridine moiety with yields up to 85% (Scheme 63) [146]. According to the authors, SnCl_2_∙2H_2_O enabled reduction of the nitro group to amine, while CuCl_2_∙2H_2_O promoted C–S bond cross-coupling reaction between dithiocarbamate salts and 3-amino-2-bromopyridine. This unique transformation featured multiple bonds breaking and forming events in a single, atom-economic process. The antitumor activity of the obtained compounds **137** was determined by the MTT assay against three human cancer cell lines such as PC-3, Bcap-37 and H460. Notably, compound **137f** (R = Et, Ar = 4-FC_6_H_4_) showed the most potent inhibitory activity with IC_50_ values of 1.04, 0.81 and 2.23 µM against PC-3, Bcap-37 and H460 cell lines, respectively.

**Scheme 64:**
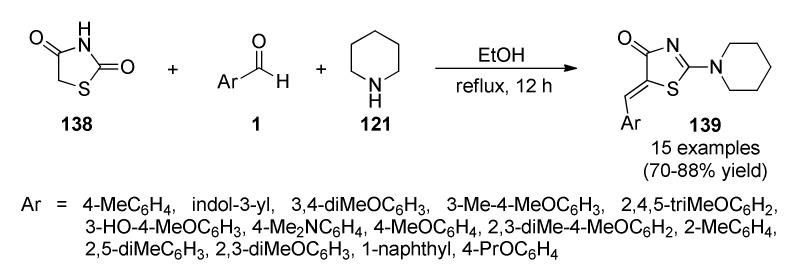
Three-component synthesis of 5-arylidenethiazolidinones **139** for screening of their in vitro antitumor activity against human *Breast* cancer cells.

In order to develop new drugs, an elegant and efficient synthesis of 5-arylidenethiazolidinones **139** was discovered in 2016. This three-component reaction involved thiazolidine-2,4-dione **138**, substituted aromatic aldehydes **1** and an excess of piperidine (**121**) in refluxing ethanol (Scheme 64) [147]. A reasonable mechanism was suggested to rationalize the formation of these 5-arylidenethiazolidinones **139**. Initially, piperidine-promoted Knoevenagel condensation between thiazolidine-2,4-dione **138** and aromatic aldehyde **1** afforded a Knoevenagel adduct. Then, nucleophilic addition of piperidine **121** onto one of the carbonyl functionalities followed by dehydration would give the observed 5-arylidenethiazolidinone **139**. These compounds were screened for their in vitro antitumor activity against human breast cancer cells such as MCF-7 and MDA-MB-453. The result suggested that compound **139i** (Ar = 4-PrOC_6_H_4_) displayed the highest inhibitory activity against MCF-7 (%GI 66.4) and MDA-MB-453 (%GI 60.0) cell lines. Furthermore, cell cycle analysis showed that compound **139i** arrest the progression of MCF-7 cells at G0/G1 phase.

**Scheme 65:**
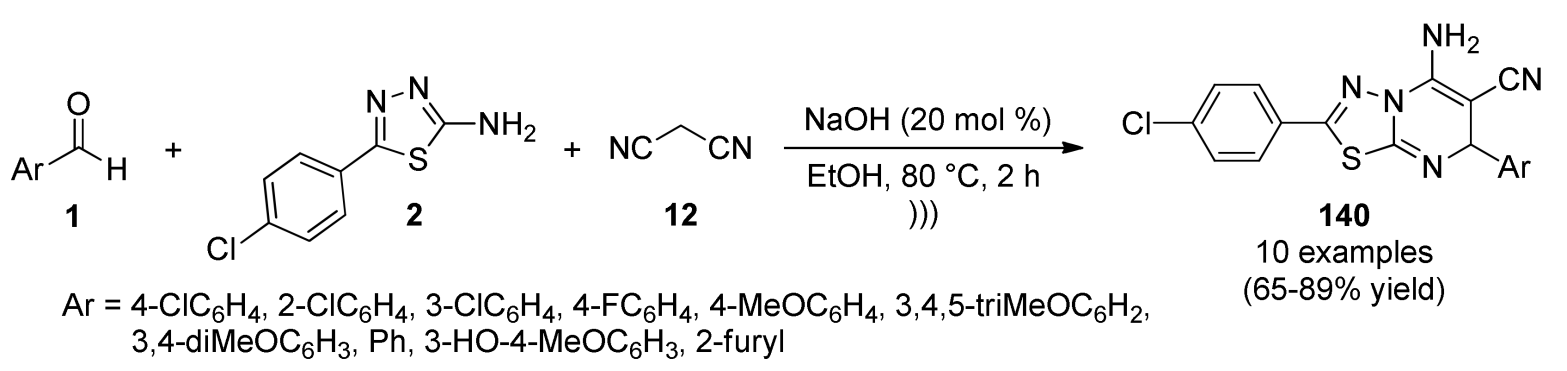
Ultrasound-assisted three-component synthesis of thiadiazolo[3,2-*a*]pyrimidine-6-carbonitriles **140** to be evaluated for their in vitro anticancer activity against MCF-7, K562, HeLa and PC-3 cancer cell lines using fluorouracil as positive control.

Alternatively, an eco-friendly access to thiadiazolo[3,2-*a*]pyrimidine-6-carbonitriles from a 1,3,4-thiadiazol-2-amine was reported in 2016 [148]. An ethanolic solution of 5-(4-chlorophenyl)-1,3,4-thiadiazol-2-amine **2**, an aromatic aldehyde **1** and malononitrile (**12**) was sonicated at 80 °C in the presence of catalytic amounts of NaOH, affording the thiadiazolo[3,2-*a*]pyrimidine-6-carbonitriles **140** in good yields (Scheme 65). A comparative study shed light on the benefits of ultrasound irradiation in terms of yields and reaction times in comparison to the same transformation under conventional heating. The obtained compounds were evaluated for their in vitro anticancer activity against MCF-7, K562, HeLa and PC-3 cancer cell lines using fluorouracil as positive control. From the anticancer activity results, compound **140i** (Ar = 3-HO-4-MeOC_6_H_3_) showed the highest GI_50_ values of 32.7, 34.3, 55.3, and 28.9 µM for the MCF-7, HeLa, K562 and PC-3 cancer cell lines, respectively. A docking study of the synthesized compounds showed good binding mode in the active site of thymidylate synthase enzyme.

**Scheme 66:**
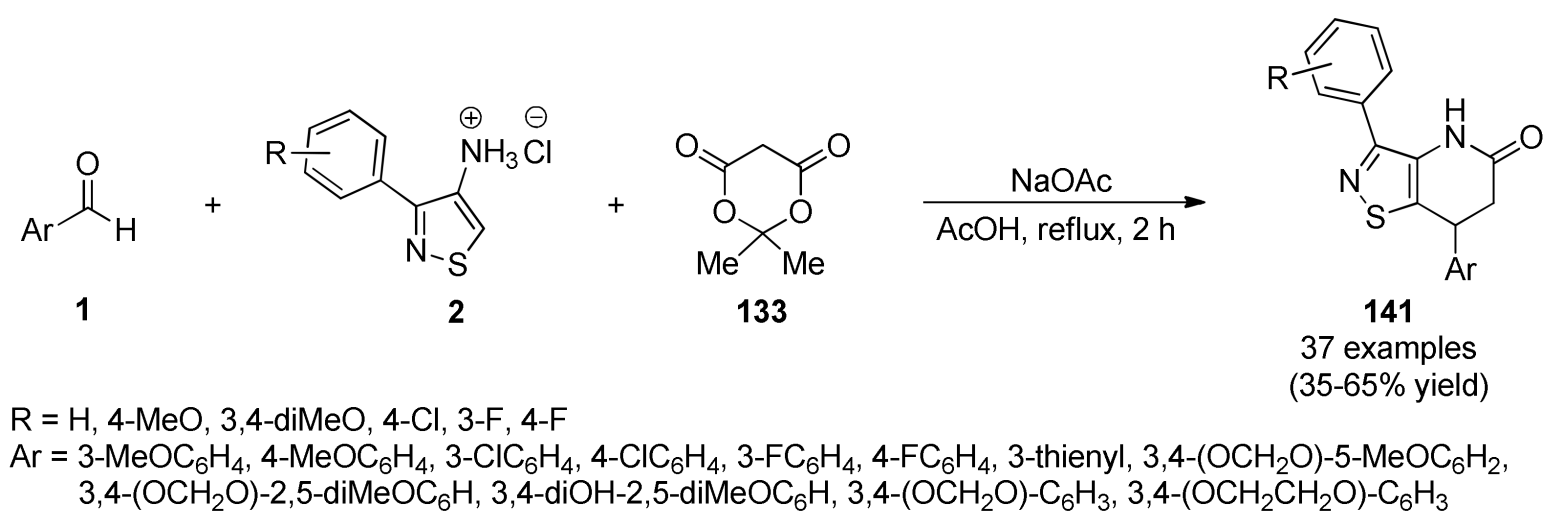
Three-component synthesis of isothiazolo[4,5-*b*]pyridin-5-one derivatives **141** for screening of their in vitro antitumor activity against a panel of human cancer cell lines.

In 2017, Semenov’s group developed similar reactions using Meldrum’s acid (**133**) as the 1,3-dicarbonyl partner [149]. Since 3-arylisothiazol-4-aminium chloride **2**∙HCl has been rarely exploited as a nucleophile in multicomponent processes, this substrate was reacted by refluxing glacial acetic acid with an aromatic aldehyde **1** and Meldrum’s acid (**133**) in the presence of sodium acetate, affording bicyclic derivatives **141** with modest yields (Scheme 66). Selected products were screened for their in vitro antitumor activity using a panel of human cancer cell lines, including CAOV-3 and TOV-112D from ovarian tumors, MDA-MB231, MDA-361/DYT2 and MDA-MB-468 from breast adenocarcinoma, MDA-MB-435 from melanoma, and NCI-H1975 from non-small cell lung cancer. Notably, compound **141b** (R = 4-MeO, Ar = 3-thienyl) exhibited the highest activity against all cancer cell lines with IC_50_ values ranging from 80.72 to 309.17 nM. Furthermore, compound **141b** blocked cell cycle in mitosis and disintegrated interphase microtubule network, suggesting its tubulin-targeting microtubule destabilizing effect.

**Scheme 67:**
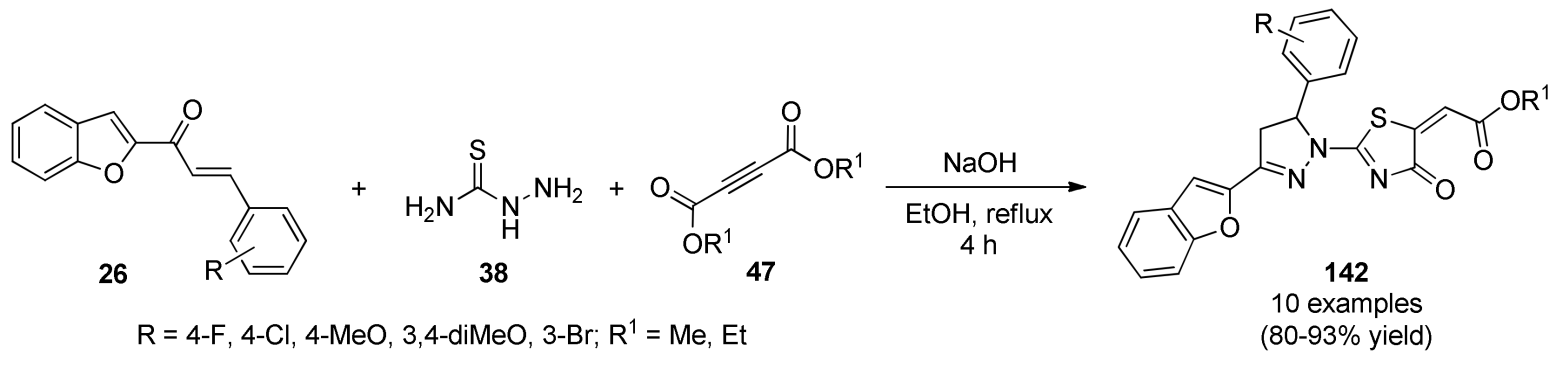
Three-component synthesis of pyrazolo-oxothiazolidine derivatives **142** for evaluation of their antiproliferative activity against A549 *Lung* cancer cell line.

In 2018, a time-efficient three-component synthesis of a series of pyrazolo-oxothiazolidine derivatives **142** was achieved through the reaction between 1-(benzofuran-2-yl)-3-(substituted-arylprop-2-en-1-ones type **26**, thiosemicarbazide **38** and dialkyl acetylenedicarboxylates **47** in the presence of sodium hydroxide in refluxing ethanol (Scheme 67) [150]. Synthesized compounds were evaluated for their antiproliferative activity against the A549 lung cancer cell line. Particularly, compounds **142a** (R = 4-F, R^1^ = Et), **142f** (R = 4-F, R^1^ = Me) and **142h** (R = 3,4-diMeO, R^1^ = Me) showed much better activity than the standard drug sorafenib (IC_50_ = 3.77 µM), with IC_50_ values of 0.93 µM, 0.80 µM and 0.96 µM, respectively. Molecular docking studies indicated that compound **142f** had the greatest affinity for catalytic site of receptors EGFR and VEGFR2.

**Scheme 68:**
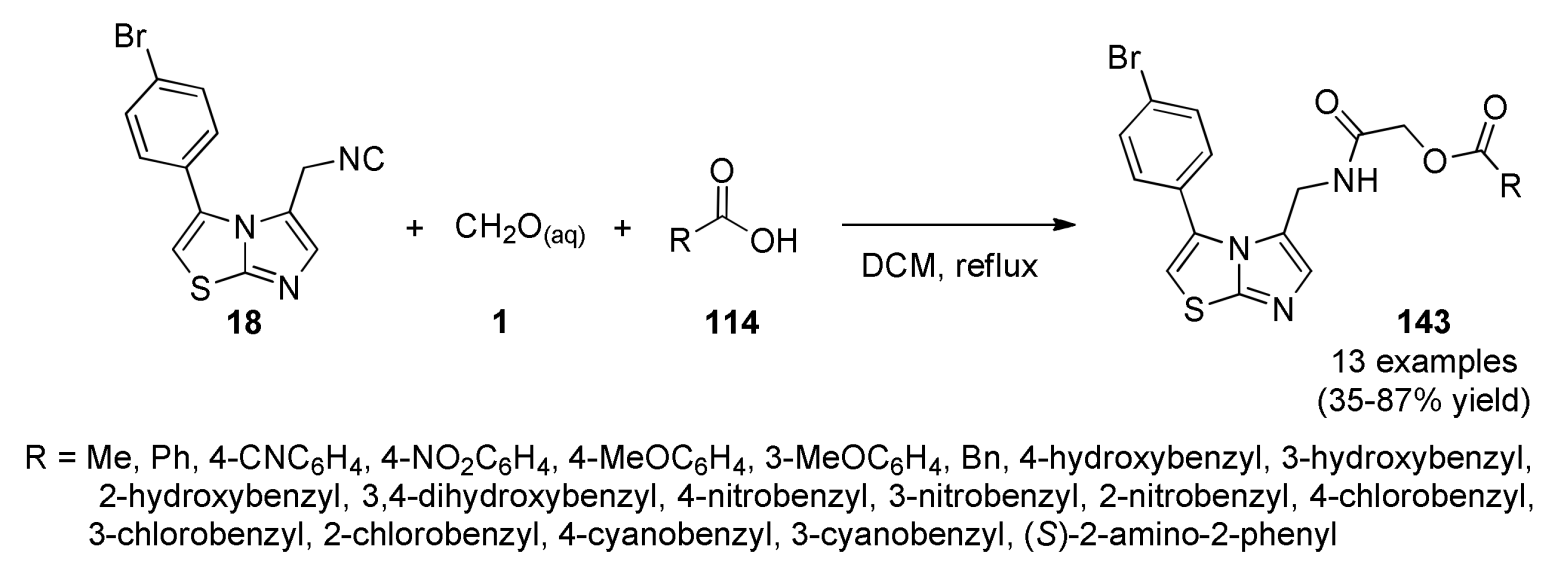
Passerini three-component reaction for the synthesis of imidazothiazoles **143** for targeting IDO1 active site.

Indoleamine 2,3-dioxygenase 1 (IDO1) has emerged as an attractive target for cancer immunotherapy. In this context, the Passerini reaction was employed to assemble a small library of imidazothiazoles **143** that target IDO1 [151]. The reaction of isocyanides **18** with aqueous formaldehyde (**1**) and functionalized phenylacetic acids **114** led to imidazothiazoles **143** containing a α-acyloxyamide moiety in the side chain, in moderate to good yields (Scheme 68). Notably, compound **143d** (R = 4-hydroxybenzyl) showed an IC_50_ value of 0.20 µM in the IDO1-based assay, a full biocompatibility at 10 µM, together with a modest inhibitory activity in A375 cells. Furthermore, molecular docking studies showed that **143d** displayed a unique binding mode in the indoleamine 2,3-dioxygenase 1 (IDO1) active site, with the side-chain protruding in an additional pocket C, where a crucial hydrogen bond is formed with Lys238.

#### 3.13.8. Indole-Based Anticancer Heterocyclic Systems

Interest in synthesizing new indole derivatives continue due to their biological properties displayed [152]. In particular, substituted indole derivatives play a key role in the synthesis of biologically active compounds especially with anticancer, antitumor, and anti-inflammatory activities [153,154,155]. They are also reported as potent inducers of apoptosis through a cell-based HTS caspase assay [156].

##### Indole-Substituted Heterocyclic Derivatives

**Scheme 69:**
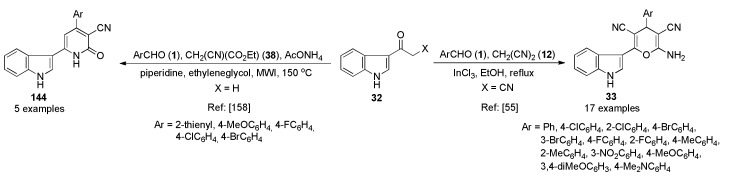
One-pot three-component methods for the synthesis of 3-pyranylindoles **33** and 4-aryl-6-indolyl-nicotinonitrile-2-ones **144**, mediated by 3-acetylindole type **32** as common precursor, for evaluation of their anticancer activity.

A series of 4-aryl-6-indolyl-nicotinonitrile-2-ones **144** were synthesized in 77–87% yield, through a one-pot four-component reaction of 3-acetylindole type **32** (X = H), aromatic aldehydes **1**, ethyl cyanoacetate (**38**) and ammonium acetate in the presence of piperidine as a catalyst under microwave irradiation (Scheme 69). Products **144** were evaluated for antiproliferative activity against human ovarian adenocarcinoma (SK-OV-3), breast adenocarcinoma (MCF-7), and cervix adenocarcinoma (HeLa) cells to establish a structure–activity relationship [157]. In the same direction, Lakshmi et al., reported a one-pot three-component method for the synthesis of 3-pyranylindoles **33** in 68–87% yield. The procedure was accomplished by a tandem Knoevenagel–Michael reaction of 3-cyanoacetyl indole type **32** (X = CN), various aromatic aldehydes **1** and malononitrile (**12**) catalyzed by InCl_3_ in ethanol under refluxing conditions (Scheme 69). The synthesized compounds **33** were evaluated for anticancer activity, in addition to anti-microbial and antioxidant activity, some of them showing good anticancer activity against MCF-7 breast cancer cell lines in comparison with the standard drug used [54].

**Scheme 70:**
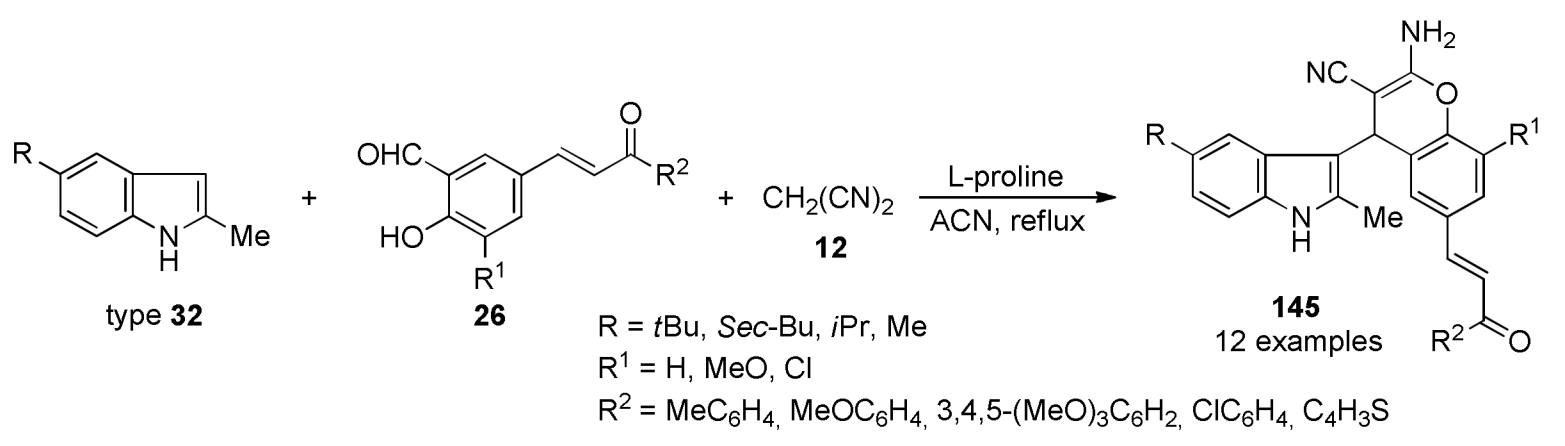
l-Proline-catalyzed three-component synthesis of indole-chalcone based benzopyran hybrids **145** for testing of their antiligase and antiproliferative activities in cancer cells.

Based on previous molecular hybridization techniques [158], Gupta et al., designed a series of indole-chalcone based benzopyran hybrid compounds **145** in order to identify molecules that can inhibit DNA ligation and cell proliferation, and may serve as drug-like molecules. Products **145** were obtained in 70–78% yield, via a three-component reaction between substituted 2-methylindoles **32**, chalcones **26** and malononitrile (**12**) in the presence of L-proline as catalyst and acetonitrile as solvent, Scheme 70. The synthetic molecules **145** were tested for their antiligase and antiproliferative activities in cancer cells. A detailed study of the most active compound **145a** (R = H, R^1^ = *t*Bu, R^2^ = C_4_H_3_S) was carried out in order to verify the mode of action and cytotoxicity in in vitro and 3D tumor models [159].

##### Carbazole (Benzo[*b*]Indole)-Substituted Heterocyclic Derivatives

Carbazole is a benzo[*b*]indole in which the benzene ring is fused with the 2,3-position of the indole ring. This motif is a privileged pharmacophore scaffold found in many biologically active compounds of diverse origins, from natural products to synthetic sources. Widespread interests of chemists have been attracted to these structures due to their biological activities and potential applications as pharmacological agents [160].

**Scheme 71:**
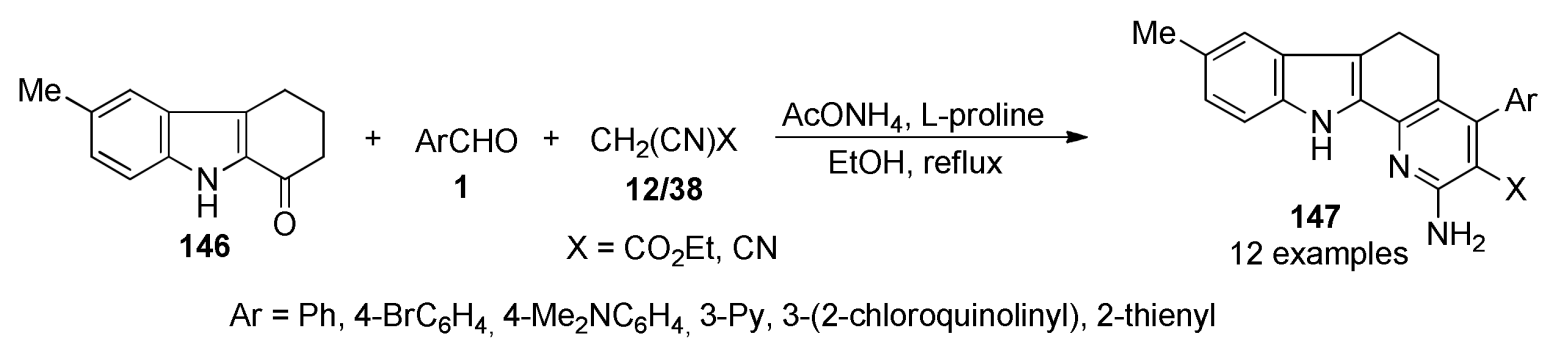
L-Proline catalyzed multicomponent synthesis of pyrido[2,3-*a*]carbazoles **147** for evaluation of their in vitro cytotoxicity against five cancer cell lines.

Thus, Indumathi et al., reported the synthesis of pyrido[2,3-*a*]carbazoles **147**, in 81–85% yield, by a four-component reaction of 6-methyl-2,3,4,9-tetrahydro-1*H*-carbazol-1-one (**146**), aromatic aldehydes **1**, malononitrile/ethylcyanoacetate (**12**)/(**38**), ammonium acetate and L-proline as catalyst in ethanol (Scheme 71). The obtained compounds **147** were evaluated for their in vitro cytotoxicity against five cancer cell lines, as well as, antimicrobial and antioxidant assays. Additionally, their primary structure-activity relationships were established [161].

**Scheme 72:**
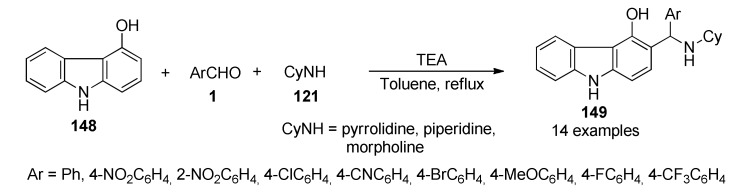
Three-component synthesis of a library of carbazole Mannich bases **149** to be tested for their in vitro antiproliferative activity against three cancer cell lines.

A library of carbazole Mannich bases **149** was synthesized in 85–94% yield from a three-component reaction of 4-hydroxycarbazole (**148**), aromatic aldehydes **1** and cyclic amines type **121** in toluene under reflux in the presence of TEA as catalyst (Scheme 72). Further, the synthesized Mannich bases **149** were tested for their in vitro antiproliferative activity against three cancer cell lines (i.e., Hela, MDA-MB-231, and HepG2). The results indicated that compounds **149** showed selective cytotoxicity against Hela cells. Additionally, in silico molecular docking study of carbazole Mannich bases **149** against colchicine binding site of the tubulin polymer was investigated [162].

**Scheme 73:**
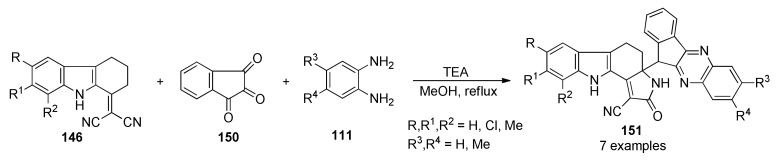
Three-component synthesis of highly poly functionalized pyrrolo[3,2-*a*]carbazoles **151**, via a TEA-catalyzed ring contraction process, for their study against *Colorectal* cancer cells.

Synthesis of highly functionalized pyrrolo[3,2-*a*]carbazoles **151** via ring contraction through an aerobic metal free rearrangement and intramolecular Michael addition reaction using one-pot three-component reaction was reported. Thus, a mixture of (tetrahydro-carbazol-1’-ylidene)-propanedinitriles type **146**, ninhydrin (**150**) and *o*-phenylenediamines **111** were subjected to reflux in methanol as the solvent and catalytic amount of TEA to afford the target products **151** in 67–90% yield (Scheme 73). The obtained compounds were studied for colorectal cancer activity, as well as, free radical scavenging in vitro via MTT and DPPH assays, respectively. Further, the structure-activity relationships were also carried out [163].

#### 3.13.9. Pyrimidine-Based Anticancer Heterocyclic Systems

The great interest in pyrimidine and dihydropyrimidine (DHPM) derivatives lies in the fact that these classes of compounds has, in principle, pronounced biological activity [164,165]. Consequently, diverse methodologies have been developed to improve the synthesis of this attractive family of compounds [166].

##### Biginelli-Mediated Synthesis of Dihydropyrimidines

The Biginelli reaction, is an MCR that involves the cyclocondensation of acetoacetic esters, aromatic aldehydes and (thio)urea [167]. The products of this three-component synthesis are identified as 3,4-dihydropyrimidin-2(1*H*)-ones (DHPMs). Thus, a library of Biginelli adducts **152** was synthesized in 31–92% yield, as described in Table 1, and evaluated as potential inhibitors of in vitro cancer cells proliferation, but also, as scavengers of reactive nitrogen and oxygen species (RNS and ROS, respectively). The capacity of all compounds **152** to inhibit cancer cells growth was dependent on the histological origin of cells, except for **152p** (X = S, R = MeSC_6_H_4_), which was highly active against all cell lines. Compounds **152k** (X = O, R = 4-HO-3-MeOC_6_H_3_), and **152x** (X = S, R = C_6_H_11_), were as potent as the reference drug doxorubicin against adriamycin-resistant ovarian and prostate cancer cells, respectively [168].

Yadlapalli et al., reported the synthesis of a series of dihydropyrimidine derivatives **153** in 46–93% yield via a Biginelli reaction, as described in Table 1. The obtained compounds were evaluated for their in vitro anticancer activity against MCF-7 human breast cancer (HBC) cell line using sulforhodamine B (SRB) assay, but also, for their antitubercular activity against *Mycobacterium tuberculosis* (MTB) H37Rv using the Microplate Alamar Blue Assay (MABA). Interestingly, compounds **153p** (X = S, R = OEt, R^1^ = Cl) and **153t** (X = S, R = 4-MeC_6_H_4_NH, R^1^ = Cl) exhibited 70.6% and 63.7% of HBC cell growth inhibition, respectively, at 10 μM concentration. Compound **153p** was also found to be the most potent in the series against MTB H37Rv with MIC value of 0.125 μM [169].

The synthesis, characterization, and application of a reusable ion-tagged iron catalyst was described. The catalyst was employed in the Biginelli reaction with impressive performance. High yields (42–99%) of DHPMs **154** were achieved when the reaction was carried out in imidazolium-based ionic liquids (ILs) (BMI·PF_6_, BMI·NTf_2_, and BMI·BF_4_) (Table 1), thus showing that the IL effects play a role in the reaction. Further, the cytotoxicity of the obtained compounds **154** was evaluated against MCF-7 cancer cell linages with encouraging results of some derivatives, which were virtually non-toxic against healthy cell linages (fibroblasts) [170].

On the other hand, two coordination polymers (CPs) were synthesized, characterized and successfully applied as robust heterogeneous catalysts for the Biginelli multicomponent reaction to obtain 3,4-dihydropyrimidin-2(1*H*)-one/thione (DHPMs) derivatives **155** in 80–99% yield (Table 1). The reaction was initially developed using both CPs and the Zn-based material showed much better catalytic activity. After the reaction optimization under batch conditions, a continuous flow protocol was developed and applied with impressive results. The mechanism of the transformation was also investigated by electrospray (tandem) mass spectrometry (ESI-MS(/MS)) analyses. Nine of the obtained DHPMs **155** had their antitumoral activities evaluated against MCF-7 (human breast cancer cells), A549 (human alveolar basal epithelial cells) and Caco-2 (human epithelial colorectal cells) cancer cell linages. Fibroblasts (healthy cells) were not affected by the tested DHPMs showing an excellent selectivity for tumor cells. Three DHPMs returned impressive results, being capable of inhibiting tumor cell proliferation in 72 h [171].

An additional series of DHPMs **156** was synthesized in 32–93% yield through a bismuth(III) catalyzed Biginelli reaction, Table 1, and their in vitro antiproliferative activity was evaluated in different human cell lines. A quantitative structure-activity relationship (QSAR) analysis was performed using Bayesian regularized artificial neural networks to model the relationships between in silico molecular descriptors and the observed antiproliferative activity of molecules across the tested cell lines. Among the compounds prepared, the molecules containing chloro atoms in their structure demonstrated a relevant potency and a selective antiproliferative activity against a *Hepatic* cancer cell line (HepaRG) without exhibiting noticeable cytotoxicity in normal dermal cells (NHDF). In prostatic (LNCaP), colon (Caco-2) and Breast (T47D and MCF-7) cancer cell lines generally compounds **156** did not exhibit relevant cytoxicity. A statistically valid QSAR model was obtained (internal validation Q^2^ = 0.663, RMSE_CV_ = 0.071, 10-fold cross-validation procedure, and external validation R^2^_pred_ = 0.740, RMSE = 0.077), which allowed the analysis of the involved relationships between molecular descriptors and the reliable prediction of the antiproliferative activity for hypothetical related compounds in the studied cell lines. Flow cytometry analysis showed that in HepaRG and MCF-7 cell lines, compound **156p** (R = MeO, Ar = 2,4-diCl_2_C_6_H_3_) did not decrease cell viability but, led to an accumulation of cells in G_0_/G_1_ phase of the cell cycle [172].

#### 3.13.10. Dihydropyridine-Based Anticancer Heterocyclic Systems

Dihydropyridine derivatives (DHPs) are recognized because of their potential antioxidant, antituberculosis, analgesic, antimicrobial, and antitumor activity [173,174]. Particularly, there is much interest in the anticancer activity of these compounds owing to different types of biological targets they might interfere with for this effect to occur (e.g., PDE3, PIM1 Kinase, and Survivin protein) [175].

In this rigard, two series of 2-oxo-1,2-dihydropyridine-3-carbonitriles **157** and their isosteric 2-imino-1,2-dihydropyridine-3-carbonitriles **158** were synthesized in 62–92% and 60–85%, respectively, through one-pot four-component reaction of the appropriate acetophenones **16**, aldehydes **1**, and ammonium acetate with ethyl cyanoacetate (**38**) or malononitrile (**12**), respectively (Table 2). The synthesized compounds were evaluated for their tumor cell growth inhibitory activity against the human HT-29 colon tumor cell line, as well as their PDE3 inhibitory activity. Compound **157p** (Ar^1^ = 3-thienyl, Ar = 2-EtOC_6_H_4_) showed tumor cell growth inhibitory activity with an IC_50_ value of 1.25 µM. Meanwhile, compound **158m** (Ar^1^ = 3-thienyl, Ar = 4-EtOC_6_H_4_) showed inhibitory effect upon PDE3 using cAMP or cGMP as substrate. No correlation was found between PDE3 inhibition and the tumor cell growth inhibitory activity. Docking compound **157p** to other possible molecular targets showed the potential to bind PIM1 Kinase [176].

On the other hand, a series of symmetrical DHPs **159** and **160** were synthesized in 87–95% and 38–55% yield, respectively, through a rapid, four-component MWI-based protocol (Table 2). Compounds **159/160** were evaluated for their tumor cell cytotoxicity against HL-60 tumor cells. A 3D-QSAR study using CoMFA and CoMSIA was carried out to decipher the factors governing MDR reversing ability in cancer. The resulting contour maps derived by the best 3D-QSAR models provided a good insight into the molecular features relevant to the biological activity in this series of analogs. 3D contour maps as a result of 3D-QSAR were utilized to identify some novel features that can be incorporated into the DHP framework to enhance the activity [177].

A sequence of DHP analogues **161** was also synthesized in 79–93% yield by Kumari et al., through a tetracomponent green synthetic method mediated by Montmorillonite-K10 (Table 2). Besides, promoter reusability, easy handling of the chemical reagent, simple reaction process, time minimization, ethanol–water solvent compatibility, and cost reduction reagent were key tools for this fruitful path. In addition, all compounds **161** were evaluated for their cytotoxic activities against three human cancer cell lines and mouse melanoma and figured out the most active compounds. Thus, these examinations recommended that DHPs and their derivatives are motivating moieties for the discovery of new anticancer drugs [178].

#### 3.13.11. Fused Dihydroquinoline-Based Anticancer Heterocyclic Systems

Dihydroquinolines (DHQs) and their fused analogues are heterocyclic scaffolds that are ubiquitous in natural products, therapeutics, fluorophores and dyes [179]. They are structures of great versatility, and their physical and chemical properties can be finely tuned using synthetic chemistry [180].

Thus, Shi et al., reported the synthesis of a series of 4-aza-podophyllotoxin analogs **162** in 70–82% yield, containing the DHQ unit in their structures. Products **162** were obtained via a three-component reaction of tetronic acid type **27**, aldehydes **1** and 2-methylbenzo[*d*]thiazol-5-amine **2** under MWI (Table 3). The method not only provided a valuable tool in design and synthesis of the fused systems **162** but also had the advantages of atom-economy, environmental-friendliness, good yields and operational simplicity. Additionally, the preliminary evaluation on the cytotoxic activity of this type of compounds resulted in the finding of several structures with potent and efficacious cytotoxicity to three carcinoma cell lines M14, MCF7 and SW1116 [145].

Another series of *para*-naphthoquinone **163** embodied 4-aza-podophyllotoxin hybrids, designed via molecular hybridization approach, were synthesized in 7–99% yield using a one-pot three-component condensation of 2-hydroxy-1,4-naphthoquinone (**94**), aldehydes **1** and 3,4-methylene-dioxyaniline (**2**) in the presence of L-proline as catalyst (Table 3). The synthetic derivatives **163** were evaluated for their antitumor activity on human hepatoma cells (HepG2) and Henrietta Lacks strain of cancer cells (Hela). Among the eighteen compounds screened, **163o** (R = 3,4,5-triMeOC_6_H_2_) has pronounced activity. The results demonstrated potential importance of molecular hybridization in the development of **163o** as potential antitumor agent [181].

An alternative route for the synthesis of a series of 4-aza-2,3-dihydropyridophenanthrene derivatives **164** were obtained in 74–90% yield via a three-component reaction of tetronic acid type **27**, substituted aldehydes **1** and 2,3,6,7-tetramethoxy phenanthrene amine **2** (Table 3). These compounds were evaluated for their cytotoxic potential against human lung (A549), prostate (PC-3 and DU145), breast (MDA-MB-231 and 4T1), gastric (HGC-27), colon (Caco-2) and cervical (HeLa) cancer cell lines. Compound **164l** (Ar = 4-*i*PrC_6_H_4_) showed significant anticancer profile against DU145 cell line with an IC_50_ value of 2.6 μM. 

Disruption of F-actin cytoskeleton structure and cell migration inhibition in DU145 cells indicate that the tumor progression and metastasis are affected by compound **164l**. Cell cycle analysis revealed that it arrests the cells in G2/M phase. Acridine orange/ethidium bromide (AO/EB) staining, Hoechst staining and annexin-V binding assays showed that cell proliferation is inhibited through induction of apoptosis [182].

Additionally, an expeditious microwave-assisted one-pot three-component synthesis of new cytotoxic phenanthrene fused-tetrahydrodibenzo-acridinones **165** was successfully accomplished in 89–95% yield. This protocol offers wide substrate scope, catalyst-free synthesis, atom-economy, simple recrystallization, high yields, and ethanol was used as green solvent (Table 3). The obtained compounds **165** were tested for their in vitro cytotoxicity against cervical (HeLa), prostate (PC-3), fibrosarcoma (HT-1080), ovarian (SKOV-3) cancer cells, and were safer to normal (Hek-293T) kidney cell line. All the compounds displayed significant cytotoxicity profile, among them **165m** (R = H, Ar = 3-CF_3_C_6_H_4_) being the most potent compound with an IC_50_ 0.24 μM against SKOV-3 ovarian cancer cells. Flow cytometry analysis revealed that cells were blocked at the G2/M phase of the cell cycle. The effect of **165m** on F-actin polymerisation was also studied. Hoechst staining showed the decreased number of viable cells and indicated apoptosis progression. Compound **165m** caused the collapse of mitochondrial membrane potential as observed via JC-1 staining and also enhanced the generation of reactive oxygen species. The increase of caspase-3 activation by 3.7 folds supported the strong apoptosis induction. In addition, an in vitro 3D-spheroid progression assay was performed with **165m** that significantly suppressed the tumor cells [183].

#### 3.13.12. Purine-Like Pyrrolo[2,3-*d*]Pyrimidine Systems of Anticancer Interest

Several purine-derivatives have been considered as important and effective drugs used in cancer chemotherapy, for immunosuppression in kidney or heart transplantation and autoimmune diseases [184,185]. Various pharmacological effects of such compounds including antiviral, antibacterial, antitumor, and antifungal activity were developed for treatment of patients suffering different illness [186]. For this reason, many thiopurine derivatives and analogs have been synthesized for evaluation of their biological activities and reduced toxicity.

In that direction a synthetic approach to access the marine alkaloid rigidins and over forty synthetic analogues based on the 7-deazaadenine (**166**), 7-deazapurine (**167**) and 7-deaza-hypoxanthine skeletons **168** and **169** was developed (Table 4). Analogues based on the 7-deaza-hypoxanthine skeleton **169** exhibited nanomolar potencies against cell lines representing cancers with dismal prognoses, tumor metastases and multidrug resistant cells. Studies aimed at elucidating the modes of action of compounds **169** in cancer cells revealed that they inhibited in vitro tubulin polymerization and disorganized microtubules in live HeLa cells. Experiments evaluating the effects of compounds **169** on the binding of [^3^H]colchicine to tubulin identified the colchicine site on tubulin as the most likely target for these compounds in cancer cells [187].

#### 3.13.13. Coumarin Derivatives

Coumarins are one of the largest classes of naturally occurring compounds and are an essential component in the pharmaceutical, cosmetics and perfumes industry. Moreover, coumarins are considered as a privileged scaffold in the design of compounds with several biological targets. Coumarin derivatives displayed a wide range of pharmacological activities like anti-cancer [188], among others [189,190,191,192,193].

**Scheme 74:**
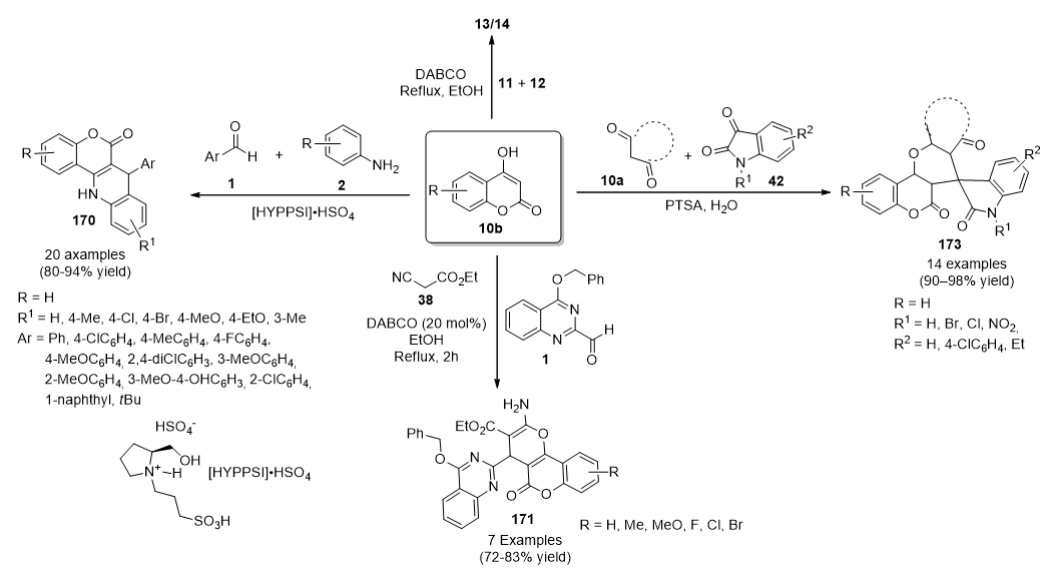
Multicomponent approaches for the synthesis of fused coumarin derivatives **13/14**, **170**, **171** and **173** of potential antitumor activity.

The coumarin nucleus has been fused with different classes of heterocyclic compounds with the aim of increasing the biological activity, especially the anticancer activity. For example, the 7,12-dihydro-6*H*-chromeno[4,3-*b*]quinoline **170** showed good anticancer activity against human cancer cell lines A-549 and MCF-7 with IC_50_ values from 0.05 μM. The derivatives **170** were synthesized via a three-component condensation of 4-hydroxycoumarin (**10b**) aromatic amines **2** and aldehydes **1** in water, catalyzed by sulfonic acid functionalized ionic liquid *L*-2-(hydroxymethyl)-1-(4-sulfobutyl)pyrrolidinium hydrogen sulfate ([HYSBPI]∙HSO_4_) (Scheme 74) [194]. Furthermore, the hibrids **171** and **13/14** containing dihydropyrano[*c*]chromene substituted quinazolines and *β*-lactams showed good anti-cancer activity against breast and colon cancer cell lines (MDA-MB 231, MDA-MB 453 and SW1116). Compounds **13/14** were previously depicted in Scheme 4 [30], while **171** were obtained by a DABCO-catalyzed three-component reaction using various substituted coumarins type **10b**, 4-(benzyloxy)quinazoline-2-carbaldehyde **1** and ethyl cyanoacetate (**38**) (Scheme 74) [195].

In another study, Kumar et al., developed a green, efficient and straightforward three-component procedure for the one-pot regioselective synthesis of spiro-chromeno indoline-triones **173** using cyclic diketones type **10a**, isatin type **42** and 4-hydroxycoumarins type **10b** in the presence of PTSA as catalyst and water as green solvent (Scheme 74). The main advantages of this protocol were short reaction time, good yield, easy work-up, high regioselectivity and reduced pollutant. Compounds **173** were effective as alkaline phosphatase (ALP) inhibitors and prostate cancer medication capabilities [196].

**Scheme 75:**
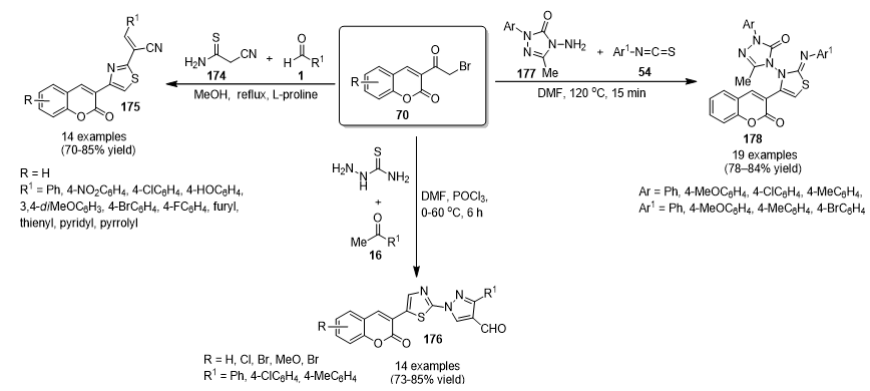
Multicomponent approaches for the synthesis of non-fused coumarin derivatives **175**, **176** and **178** for evaluation of their antitumor activity.

A simple and efficient one-pot three-component method for the synthesis of a series thiazolyl-coumarin hybrids **175** was reported by Kavitha et al. [197]. The hybrids **175** were synthesized by treating equimolar amounts of 3-(2-bromoacetyl)coumarins type **70** with various aryl/heteryl aldehydes **1** and 2-cyanothioacetamide **174** and catalyzed by L-proline in MeOH (Scheme 75). The synthesized compounds were screened for the anti-hepatocarcinoma activity. Remarkably, compounds **175** showed good to excellent anti-hepatocarcinoma activity with the support of molecular docking studies. Similarly, Vaarla et al., reported the synthesis of a series of coumarin substituted thiazolyl-3-arylpyrazole-4-carbaldehydes **176** via an efficient, one-pot multicomponent approach involving 3-(2-bromoacetyl)coumarins type **70**, thiosemicarbazide and substituted acetophenones type **16** via a Vielsmeier-Haack reaction (Scheme 75). 

These coumarin derivatives **176** showed moderate to appreciable cytotoxic activities against MCF-7, DU-145 and Hela cell lines. Compounds **176m** (R = 6,8-diCl, R^1^ = 4-MeC_6_H_4_) and **176n** (R = 6,8-diBr, R^1^ = 4-MeC_6_H_4_) exhibited significant cytotoxic activity with IC_50_ values of 5.75 and 6.25 μM, respectively, against Hela cell line [198]. In 2015, Shaikh et al., described a microwave-expedite green synthesis of coumarin-3-yl-thiazol-3-yl-1,2,4-triazolin-3-ones **178** by a one-pot multicomponent method from 3-(2-bromoacetyl)coumarins type **70**, 1,2,4-triazolone **177** and aryl isothiocyanate type **54** at 120 ^0^C in DMF without catalyst, Scheme 75. The obtained compounds were evaluated for their anticancer activity against MDA-MBA-231(breast cancer), A549 (lung cancer), K562 (leukaemia) and HeLa (human cervical cancer) cell lines by MTT assay. The synthesized compounds **178** showed promising inhibition with IC_50_ values in the range of 0.16-1.12 μM and the docking studies revealed that the compounds displayed polar and hydrophobic interactions with the active site of EGFR-TKD with amino acid residues like Met769, Lys721 and THR830 [199].

**Scheme 76:**
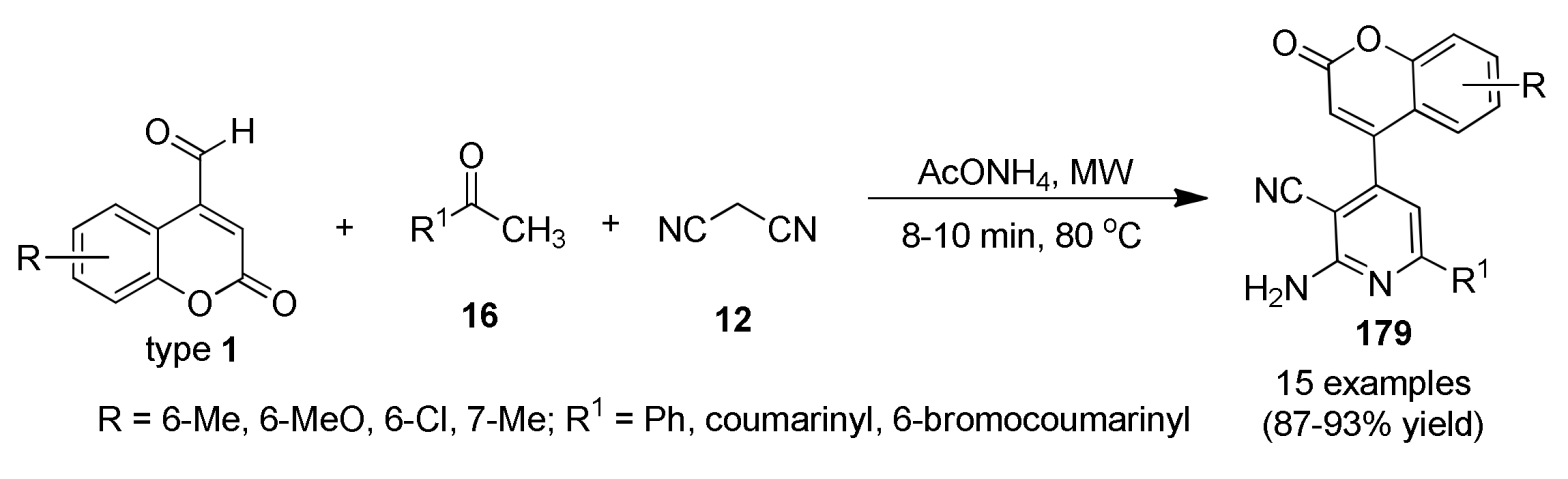
MW-assisted multicomponent synthesis of a series of coumarin-pyridine hybrids **179** for anticancer activity against HT29, HepG2 and KB cell lines.

Chougala et al., designed and synthesized a series of coumarin-pyridine hybrids **179**. These hibrids were prepared via a one-pot four-component condensation reaction using MWI-assisted solvent free conditions from 4-formylcoumarins **1**, ammonium acetate, different ketones **16** and malononitrile (**12**) (Scheme 76). All synthesized compounds presented a remarkable anticancer activity against HT29, HepG2 and KB cell lines in the range of 4.22 to 27.58 μM. Compound **179f** (6-methyl on coumarin and 6-coumarin on pyridine) and **179k** (6-methyl on coumarin and 6-bromocoumarin on pyridine) presented prominent antitumor activity with IC_50_ values of 4.22 and 5.62 μM for HT-29, 5.02 and 6.94 μM for HepG2 and 6.82 and 9.64 μM for KB, respectively [200].

**Scheme 77:**
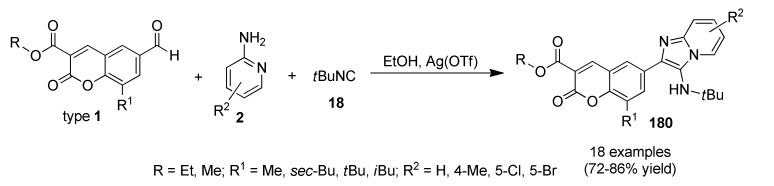
Silver(I)-catalyzed three-component synthesis of a series of coumarin-5-imidazo[1,2-*a*]pyridine hybrids **180** for evaluation of their cancer cell growth inhibition using MTT assay.

In the continuous search of new coumarin derivatives for anticancer activity, Sashidhara et al., synthetized, a series of biologically active coumarin-5-imidazo[1,2-*a*]pyridine hybrids **180** by employing the silver(I)-catalyzed Groebke-Blackburn-Bienayme multicomponent reaction (Scheme 77). Some of the synthetized compounds were evaluated for cancer cell inhibition using MTT assay against breast cancer (MCF-7, MDAMB-231) and cervical cancer (Ishikawa) cell line. Compound **180o** (R = Me_,_ R^1^ = *i*Pr, R^2^ = 4-Me) was the most active against MDA-MB-231 cells with IC_50_ of 14.12 μΜ. In addition, it was suggested that induced apoptosis in MDA-MB-231 cells was associated with mitochondrial depolarization and could induce cell cycle arrest in MDA-MB-231 cells at G_0_/G_1_ phase with a concomitant reduction in S-phase and G_2_/M phase [201].

#### 3.13.14. Spiro Derivatives

**Scheme 78:**
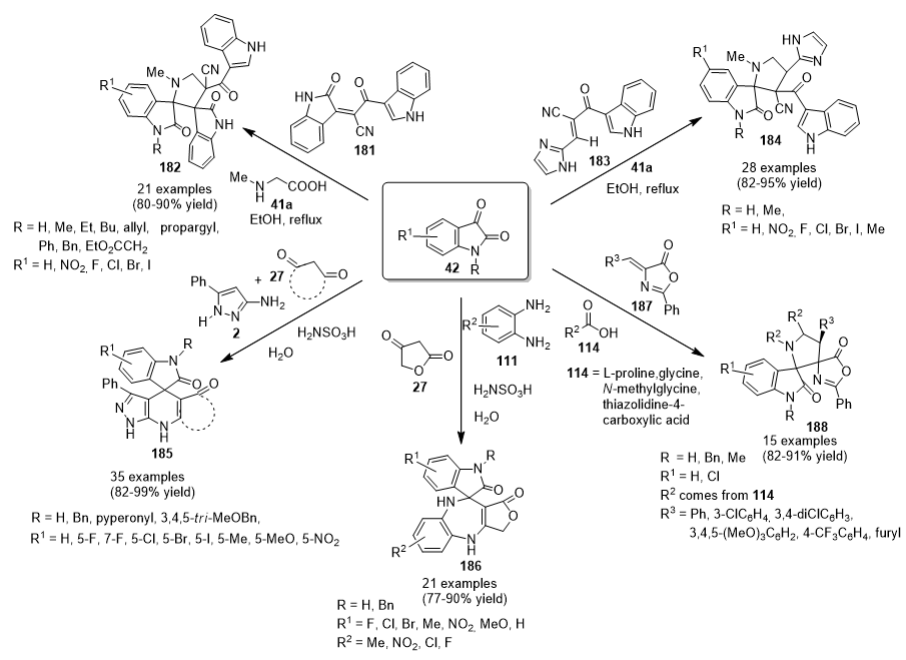
Multicomponent approaches for the synthesis of spirooxindole derivatives **182**, **184**, **185**, **186**, and **188** for evaluation of their antitumor activity.

Spiro compounds are considered privileged structures and often show interesting biological activity. They are found in a number of natural and synthetic compounds exhibiting wide range of activities against a variety of disease areas, such as anticancer [202], among others [203,204,205,206,207]. Multicomponent reaction (MCR) is a powerful tool for get new spiro-derivates with potential biological activity. In this sense, Arun et al., synthesized a series of dispirooxindole-pyrrolidine derivatives **182** and **184** through a one-pot tandem/domino approach via a 1,3-dipolar cycloaddition reaction. This reaction was performed by heating an equimolar mixture of substituted isatins **42**, sarcosine (**41a**), 3-(1*H*-indol-3-yl)-3-oxo-2-(2-oxoindolin-3-ylidene)propanenitrile (**181**) and 3-(1*H*-imidazol-2-yl)-2-(1*H*-indole-3-carbonyl)acrylonitrile (**183**), respectively. The reaction proceeded in ethanol under reflux for 90–120 min, affording the spirooxindole derivatives **182/184** in 80–92% yields (Scheme 78). These compounds were evaluated against A549 human lung adenocarcinoma cancer cell lines, where the spirooxindoles **182/184** exhibited very good anticancer activity with percentage of inhibition in the range of 60.22–67.67%. Compound **182i** (R = CH≡CCH_2_-, R^1^ = H) showed IC_50_ value of 50 μM [208,209]. In a similar study, it was reported a simple, mild and efficient one-pot multicomponent reaction for the synthesis of pyrazolopyridine- and benzodiazepine-based spirooxindoles **185** and **186**, respectively. This one-pot protocol proceeded via a three-component reaction of isatins type **42**, tetronic acid **27**, 5-phenyl-1*H*-pyrazol-3-amine type **2** (for spirooxindoles **185**) and *o*-phenylenediamines type **111** (for spirooxindoles **186**), using sulfamic acid (H_2_NSO_3_H) as a green catalyst and water as solvent (Scheme 78). Compounds **185**/**186** were evaluated for their in vitro cytotoxic activities against a selected human cancer cell lines, such as Breast cancer (MCF-7 and MDA-MB-231), prostate cancer (DU-145), cervical cancer (HeLa) and lung cancer (A549). The spirooxindoles **185**/**186** showed moderate to very good cytotoxicity with IC_50_ values in the range of 0.35–75.20 μM. Compounds **185o** (R = pyperonyl, R^1^ = 5-Cl) and **186h** (R = R^1^ = R^2^ = H) exhibited significant cytotoxicity with IC_50_ 0.35 μM and 1.14 μM values against MDA-MB-231 and DU-145 cell lines, respectively [210,211].

Hui et al., published a direct route to prepare biologically relevant spirooxindole-pyrrolidine, pyrrolizidines and pyrrolothiazoles **188** by a one-pot, multicomponent 1,3-dipolar cycloaddition reaction via azomethine ylides from isatin type **42** and an amino acid derivative **114** (Scheme 78). The advantages of this protocol included, high yields, simple work-up procedure and regio- and diastereo-selectivities. Compounds **188** were evaluated for their antiproliferative activity against various cancer cell lines. Although all compounds showed good cytotoxicity activity against the tested cell lines, studies showed that compound **188j** (R = R^1^ = H, R^3^ = Ph) displayed the highest inhibitory activity against HCT116 (colon cancer) and HepG2 (hepatocellular carcinoma) at a concentration of 10 μM [212].

**Scheme 79:**
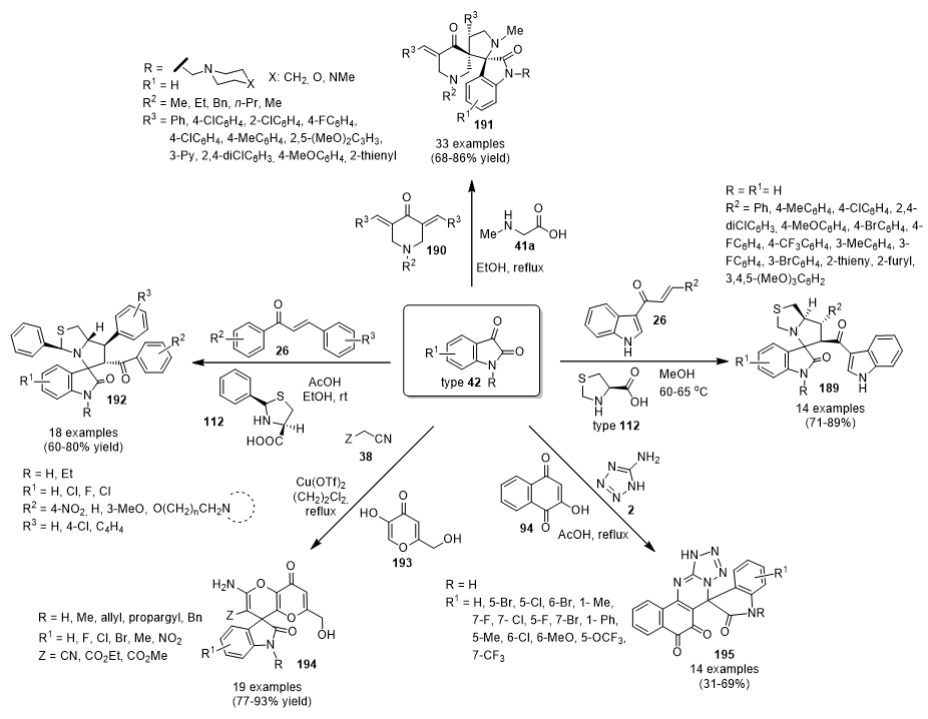
Multicomponent approaches for the synthesis of spirooxindole derivatives **189**, **191**, **192**, **194**, and **195** for evaluation of their tumor cell growth inhibitory activity.

Similarly, a class of diastereoselective spiropyrrolidine-oxindole derivatives **189, 191** and **192** were synthesized from isatins type **42**, *α*,*β*-unsaturated ketones **26** and **190** and amino acids **41a** and **112** in a one-pot multicomponent reaction via 1,3-dipolar cycloaddition (Scheme 79). Most of the spiro-oxindoles **189, 191** and **192** exhibited potent antitumor properties against different cancer cell lines. For example, compounds **192** inhibited the growth of MCF-7, T47D and MDA-MB-231 cells with an IC_50_ less than 20.0 μM. Compound **191** (R^2^ = Me, R^3^ = 4-MeC_6_H_4_) showed bio-potency against the HepG2 (hepatocellular cancer) cell line, comparable to that for doxorubicin hydrochloride (standard reference), and the spiropyrrolidine-oxindole **189k** (R = R^1^ = H, R^2^ = 4-FC_6_H_4_) presented a high cytotoxic activity and selectivity against colon cancer cells HCT-116 (IC_50_ = 7 μM, SI: 3.7), and HepG2 (IC_50_ = 5.5 μM, SI: 4.7) [213,214,215].

Parthasarathy et al., reported a catalytic efficiency of Cu(OTf)_2_ for the synthesis of spiropyrano[3,2-*b*]pyran-4(8*H*)-ones **194** through a three-component reaction between isatins **42**, nitriles **38** and kojic acid (**193**) (Scheme 79). The synthesized compounds were evaluated for their tumor cell growth inhibitory activity against the human lung cancer cell line (A549). Compounds exhibited moderate to good cytotoxicity, out of which, two derivatives **194e** (R = Me, R^1^ = H, Z = CN) and **194c** (R = H, R^1^ = Cl, Z = CN) exhibited good anticancer potency with IC_50_ values of 51.1 and 51.4 μM, respectively [216].

A library of spirooxindole-*O*-naphthoquinone-tetrazolo[1,5-*a*]pyrimidine hybrids **195** was designed, synthesized and evaluated as potent antitumor agents. Hybrids **195** were obtained in 31–69% yield via a one-pot three-component reaction of isatins type **42**, 5-aminotetrazole type **2** and 2-hydroxy-1,4-naphthoquinone **94** in refluxing AcOH (Scheme 79). These hybrids exhibited relatively high cytotoxic activity against cancer cell line HepG2 (IC_50_ = 2.86–36.34 μM), while normal cell line LO2 was less sensitive to these hybrids (IC_50_ = 36.37–248.39 μM). Among all the tested compounds, structure **195e** (R = 1-Me, R^1^ = 7-F) was the most active derivative with a mean IC_50_ value of 2.86 μM [217].

**Scheme 80:**
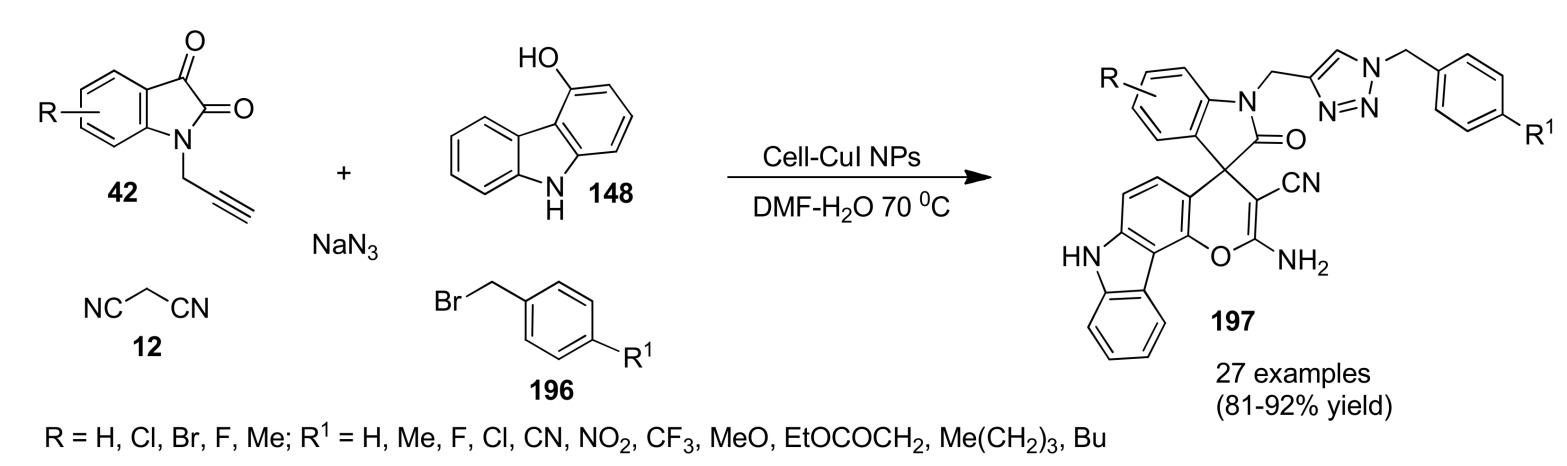
Cell-CuI NPs-catalyzed multicomponent synthesis of spirochromenocarbazoles **197** for evaluation of their antitumor activity.

In other study, a series of spirochromenocarbazoles **197** was synthesized via a click chemistry-based one-pot, five-component reaction between *N*-propargyl-isatins **42**, alkyl/arylalkyl halides **196**, 4-hydroxycarbazole (**148**), malononitrile (**12**) and sodium azide in a mixture DMF/H_2_O at 70 ^0^C using cellulose supported CuI nanoparticles (Cell-CuI NPs) as the heterogeneous catalyst, Scheme 80. The synthesized compounds were screened against a panel of six human cancer cells namely MCF-7 and MDA-MB-231 (breast cancer), HeLa (cervical cancer), PANC-1 (pancreatic cancer), A-549 (lung cancer) and THP-1 (acute monocytic leukemia). Some compounds presented excellent activity against MCF-7, MDA-MB-231 and HeLa cancer cell lines. Particularly, compound **197j** (R = H, R^1^ = Bu) displayed the highest activity with IC_50_ values 4.8, 8.4 and 9.2 µM, respectively [218].

**Scheme 81:**
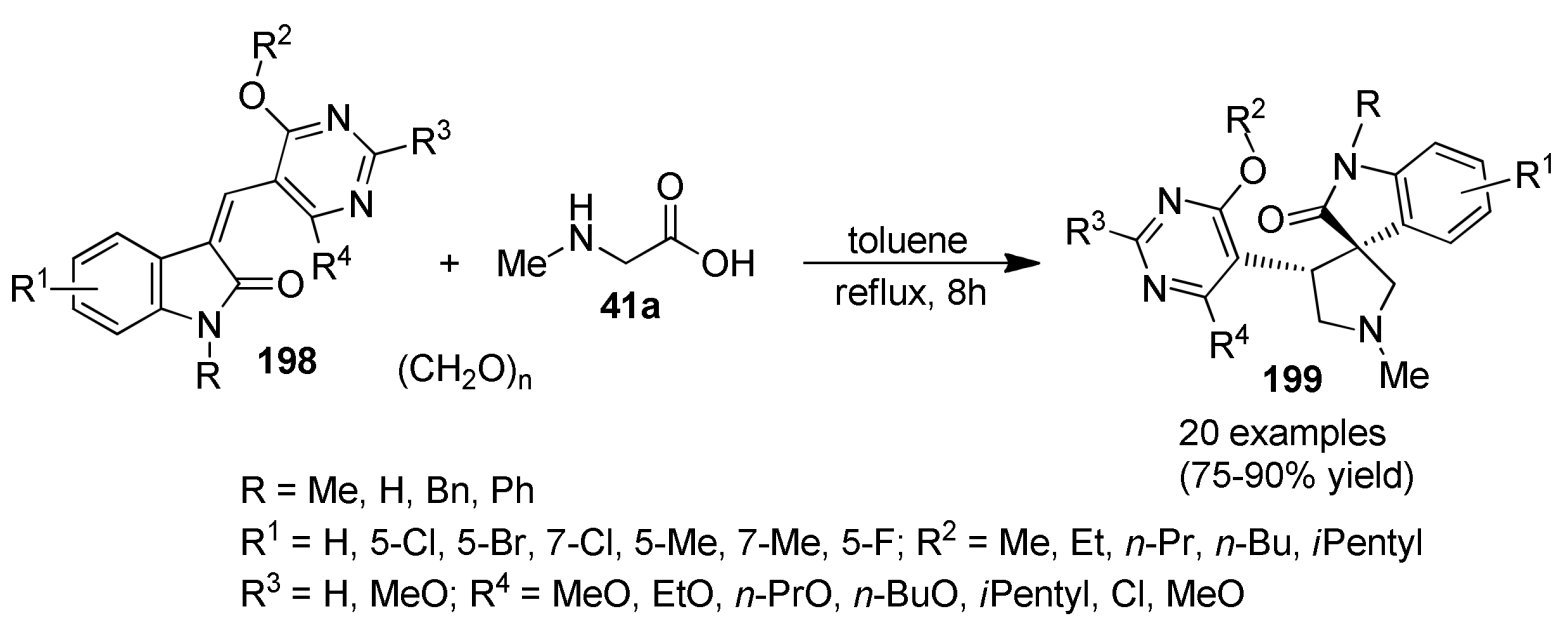
Three-component synthesis of pyrimidine-fused 3-alkenyloxindole **199** for evaluation of their tumor cell growth inhibitory activity.

In 2016, a three-component 1,3-dipolar cycloaddition reaction of pyrimidine-fused 3-alkenyloxindole **198** with azomethine ylides (thermally generated in situ from sarcosine (**41a**) and polyformaldehyde) for the synthesis of a series of pyrimidine-fused spiropyrrolidine oxindoles **199** was investigated by Liu et al. The products were obtained in high yields (up to 90% yield) with good diastereoselectivity (up to > 20:1) (Scheme 81). The anticancer activity was screened by MTT assays against lung cancer cells A549, human prostate cancer cells PC-3, and human leukemia cells K562 by using cisplatin as a positive control. Some spiropyrrolidine oxindoles **199** showed GI_50_ ranging from 8.9 µM to 23.2 µM in vitro inhibitory activity against human leukemia cells K562, and exhibited equipotent or more potent activity than the positive control cisplatin (up to 3.0 times) [219].

**Scheme 82:**
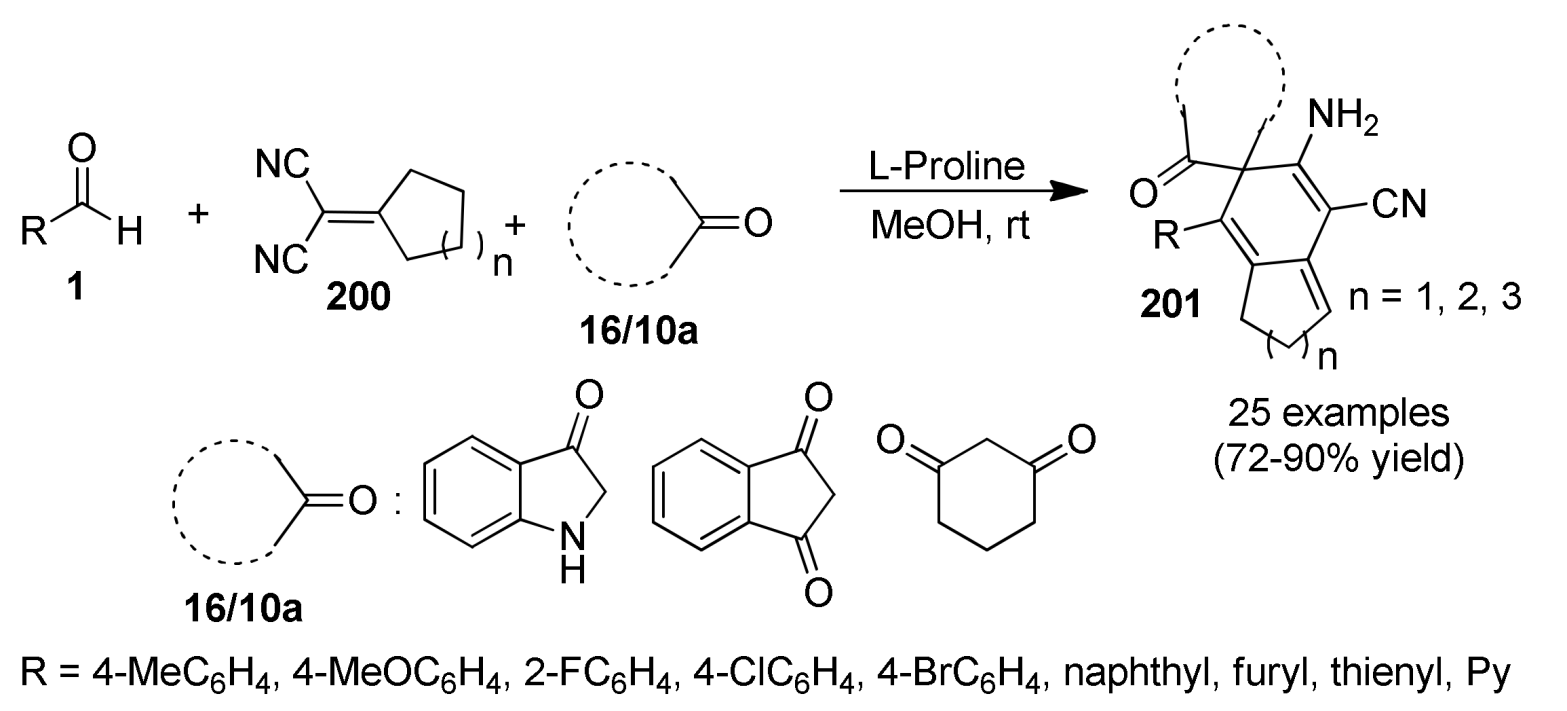
L-Proline-catalyzed three-component synthesis of spirocarbocycle derivaties **201** for evaluation of their anticancer activity.

Sudhapriya et al., reported the synthesis of spirocarbocycle derivaties **201** by a multicomponent-domino reaction that involved cyclic nucleophiles **16/10a**, vinyl malononitriles **200** and different aldehydes **1** using L-proline as catalyst in methanol as solvent (Scheme 82). The reaction proceeded smoothly in good yields with good diastereoselectivities. The synthesized spirocarbocycles **201c** (R = 4-MeC_6_H_4_, n = 3), **201i** (R = 4-ClC_6_H_4_, n = 3) and **201h** (R = thiophenyl, n = 2) showed good anticancer activity against A549 cancer cell line with IC_50_ values of 50, 30 and 20 μM, respectively. The docking scores showed that the spirocarbocyclic molecules **201** had good potential against the human lung cancer cells [220].

#### 3.13.15. Miscellaneous of Diverse Heterocyclic Systems with Potential Antitumor Activity

**Scheme 83:**
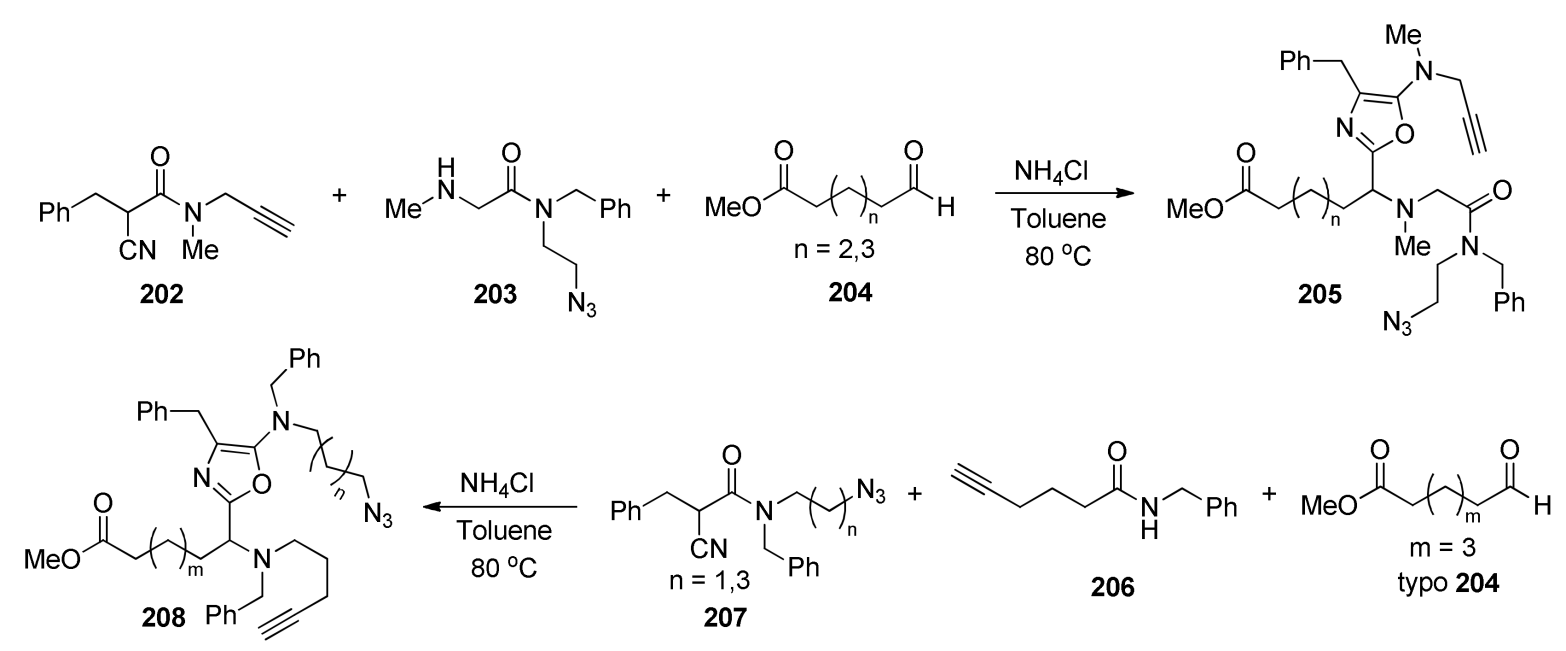
NH_4_Cl-Catalyzed three-component synthesis of highly functionalized oxazoles **205** and **208** for evaluation of their anticancer activity.

Pirali et al., reported the synthesis of highly functionalized structures **205** and **208** in 60–62% and 60–68% yields, respectively, as intermediates of macrocyclic peptide mimetics by exploiting a three-component and an azide–alkyne [3+2] cycloaddition reaction catalyzed by ammonium chloride (Scheme 83). The obtained compounds were screened as HDAC inhibitors allowing to identify some of these compounds with promising biological activity. Moreover, in order to rationalize the biological results, computational studies were also performed [221].

**Scheme 84:**
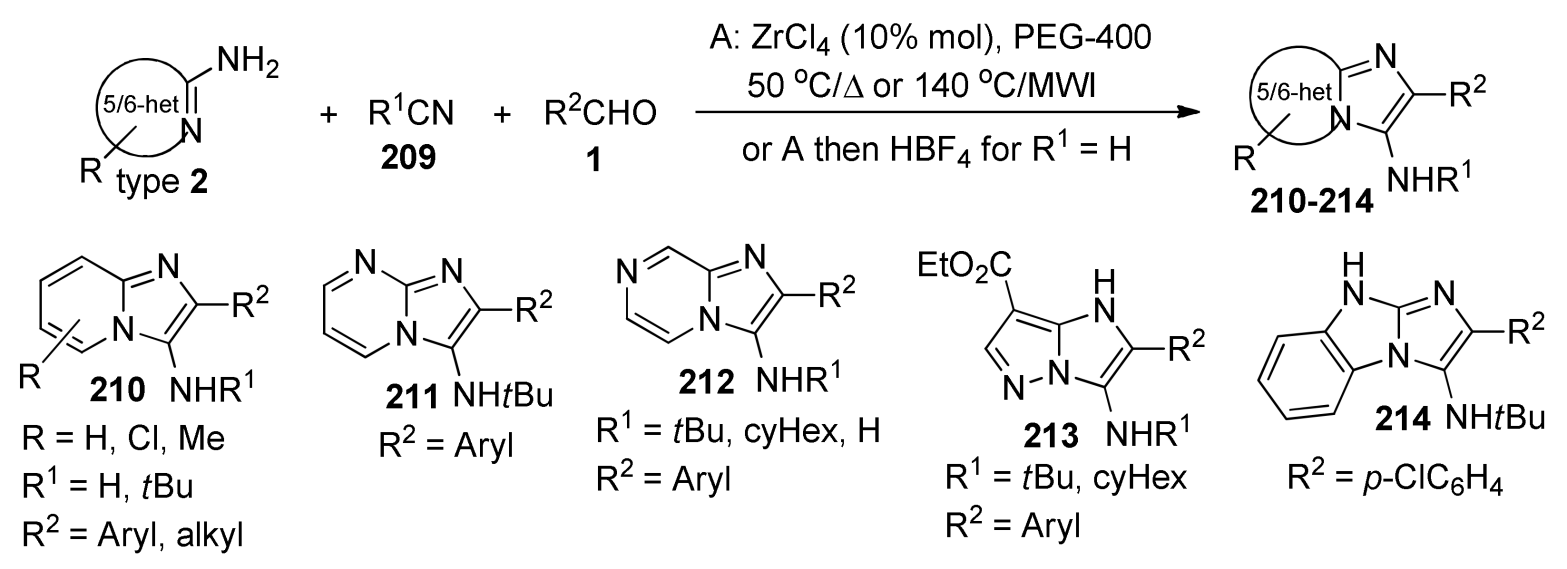
ZrCl_4_-Catalyzed three-component synthesis of bicyclic *N*-fused aminoimidazoles **210**–**214** for evaluation of their anticancer activity.

On the basis of structures of known topoisomerase II catalytic inhibitors and initial molecular docking studies, bicyclic *N*-fused aminoimidazoles **210**–**214** were predicted by Baviskar et al., as potential topoisomerase II inhibitors. These compounds were synthesized in high yields by a three-component reaction, as described in Scheme 84, and evaluated against human topoisomerase IIα (hTopoIIα) in decatenation, relaxation, cleavage complex, and DNA intercalation in vitro assays. Several of the obtained compounds exhibited potent inhibition of catalytic activity of hTopoIIα while not showing DNA intercalation. Molecular docking studies and molecular dynamics (MD) simulation analysis, ATPase-kinetics and ATP-dependent plasmid relaxation assay revealed the catalytic mode of inhibition of the obtained compounds plausibly by blocking the ATP-binding site. Some of the obtained compounds also showed potent anticancer activities in kidney and breast cancer cell lines, low toxicity to normal cells, relatively higher potency compared to etoposide and 5-fluorouracil in kidney cancer cell lines, and potent inhibition in cell migration. These compounds were found to exert apoptotic effect in G1/S phase [222].

**Scheme 85:**
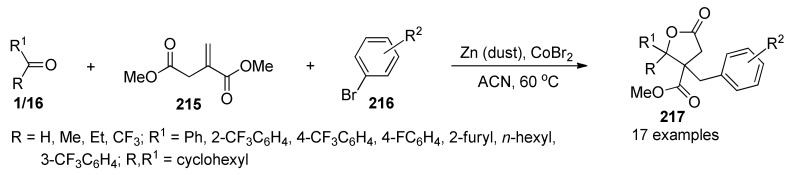
Zn-Mediated three-component synthesis of polysubstituted *γ*-butyrolactone **217** for evaluation of their antiproliferative activity.

A series of 2,3-tri- and tetrasubstituted *γ*-butyrolactone **217** analogous to paraconic acids were synthesized in 14–99% yield, through a one-step and straightforward three-component reaction between carbonyl compounds type **1/16**, dimethyl itaconate (**215**) and aryl bromides **216** (Scheme 85). The in vitro cytotoxic activity of representative compounds was evaluated against a panel of human cancer cell lines (KB, HCT-116, MCF-7, HL60). While most molecules **217** exhibited a low to moderate background activity on both KB and HL60 cancer cell lines, compound **217m** (R = Ph, R^1^ = Me, R^2^ = 4-OMe), showed increased antiproliferative activity against both cell lines with IC_50_ values in the 0.1 to 1.0 μM range. An extended evaluation indicated that this compound also inhibited PC3, SK-OV3, MCF-7R and HL60R cell growth in the same fashion [223].

**Scheme 86:**
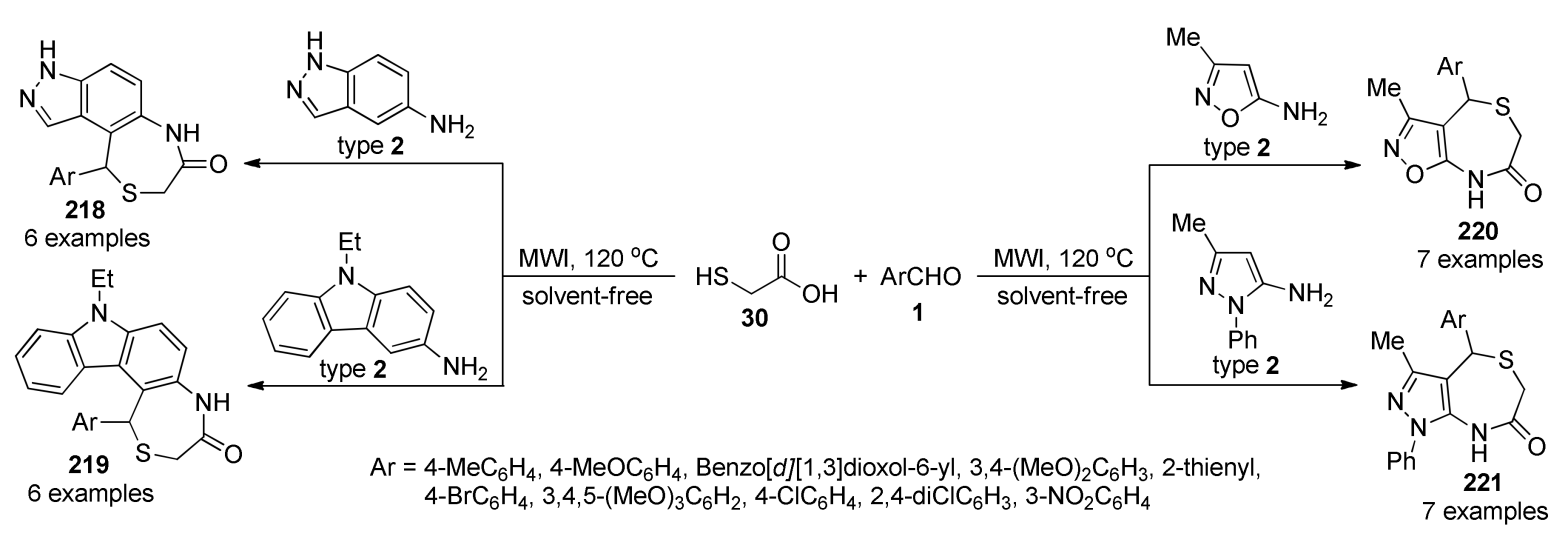
MWI-Assisted three-component synthesis of 1,4-thiazepine-embedded with carbazole, pyrazole or isoxazole derivatives **218**–**221** for evaluation of their in vitro cytotoxic activity.

In a study Shi et al., achieved the design and diversity-oriented synthesis of libraries of 1,4-thiazepine derivatives **218** (88–93% yield), **219** (73–93% yield), **220** (90–94% yield) and **221** (90–93% yield) embedded with carbazole, pyrazole or isoxazole motifs via MWI-assisted three-component reactions under solvent-free condition (Scheme 86). 

This strategy led to a green and facile access to the above compounds with prominent features of high structural diversity, short reaction times, high yields and environmental friendliness. Further, the obtained compounds were subjected to the test of their in vitro cytotoxic, as well as, antioxidant activities, resulting in the finding that these compounds not only displayed significant antioxidant activity, but also exhibited remarkably selective cytotoxicity to carcinoma cell line HCT-116 [224].

**Scheme 87:**
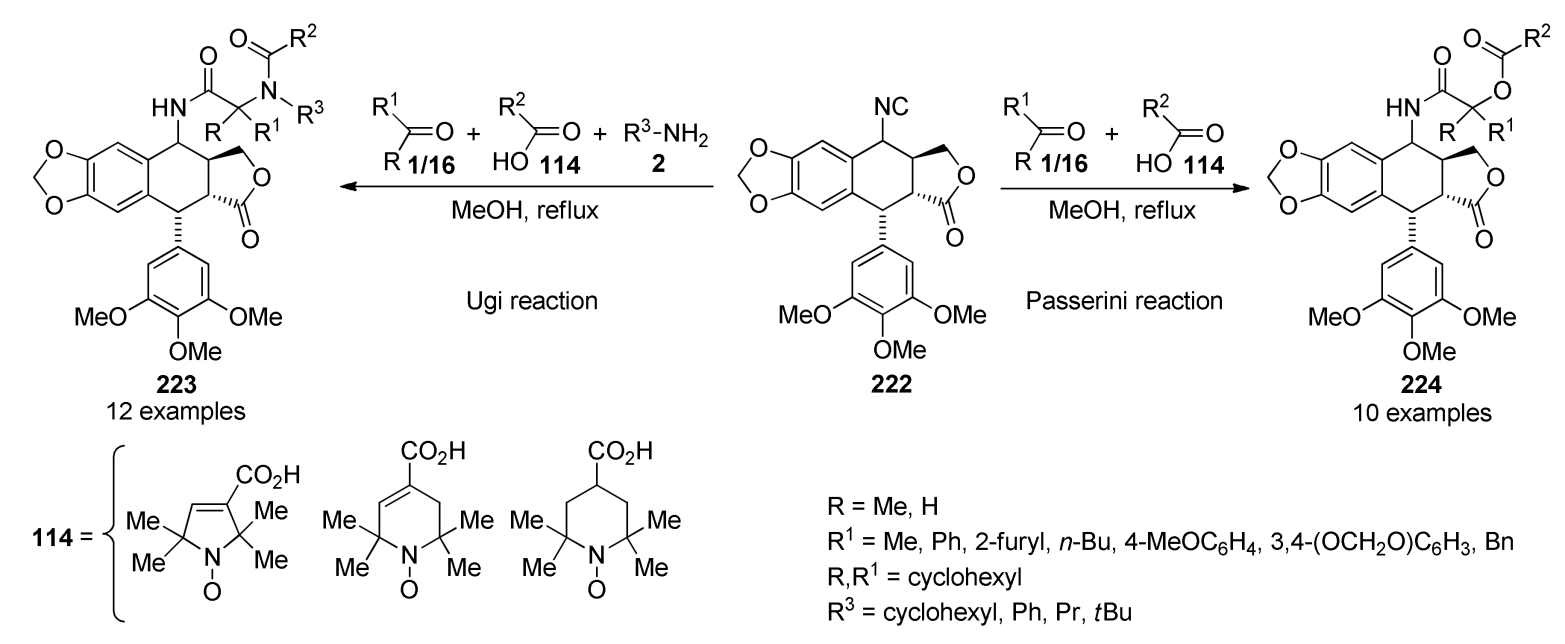
Ugi- and Passerini-mediated multicomponent synthesis of spin-labeled podophyllotoxin analogs **223** and **224** for evaluation of their in vitro cytotoxicity against four human cancer cell lines.

Due to its antitumor interest, two series of third generation of spin-labeled podophyllotoxin analogs **223** and **224** were synthesized via isocyanide-mediated four- and three-component reactions. Both processes were inspired by Ugi (Ugi-4CR) and Passerini (P-3CR) multicomponent reactions, respectively, through the isocyanopodophyllotoxin (**222**) as the key starting material (Scheme 87). Subsequently, the obtained products **223** (39–84% yield) and **224** (36–85% yield) were subjected to evaluation of their cytotoxicity against four human cancer cell lines (A-549, DU-145, KB and KBvin). Most of the evaluated compounds exhibited potent cytotoxic activity against all four cell lines, mainly against the drug resistant KBvin cancer cell line. Compounds **224e** (R = H, R^1^ = 4-MeOC_6_H_4_, R^2^ = 2,2,5,5-tetramethyl-2,5-dihydro-1*H*-pyrrol-3-yl-*N*-oxide) (IC_50_ = 0.60-0.75 μM) and **224h** (R = H, R^1^ = 3,4-methylenedioxy-C_6_H_3_, R^2^ = 2,2,5,5-tetramethyl-2,5-dihydro-1*H*-pyrrol-3-yl-*N*-oxide) (IC_50_ = 1.12-2.03 μM) showed superior potency to etoposide (IC_50_ = 2.03 to >20 μM), a clinically available anticancer drug. According to these results, compounds **224e** and **224h** were claimed for further developments as a new generation of epipodophyllotoxin-derived antitumor clinical trial candidates [225].

**Scheme 88:**
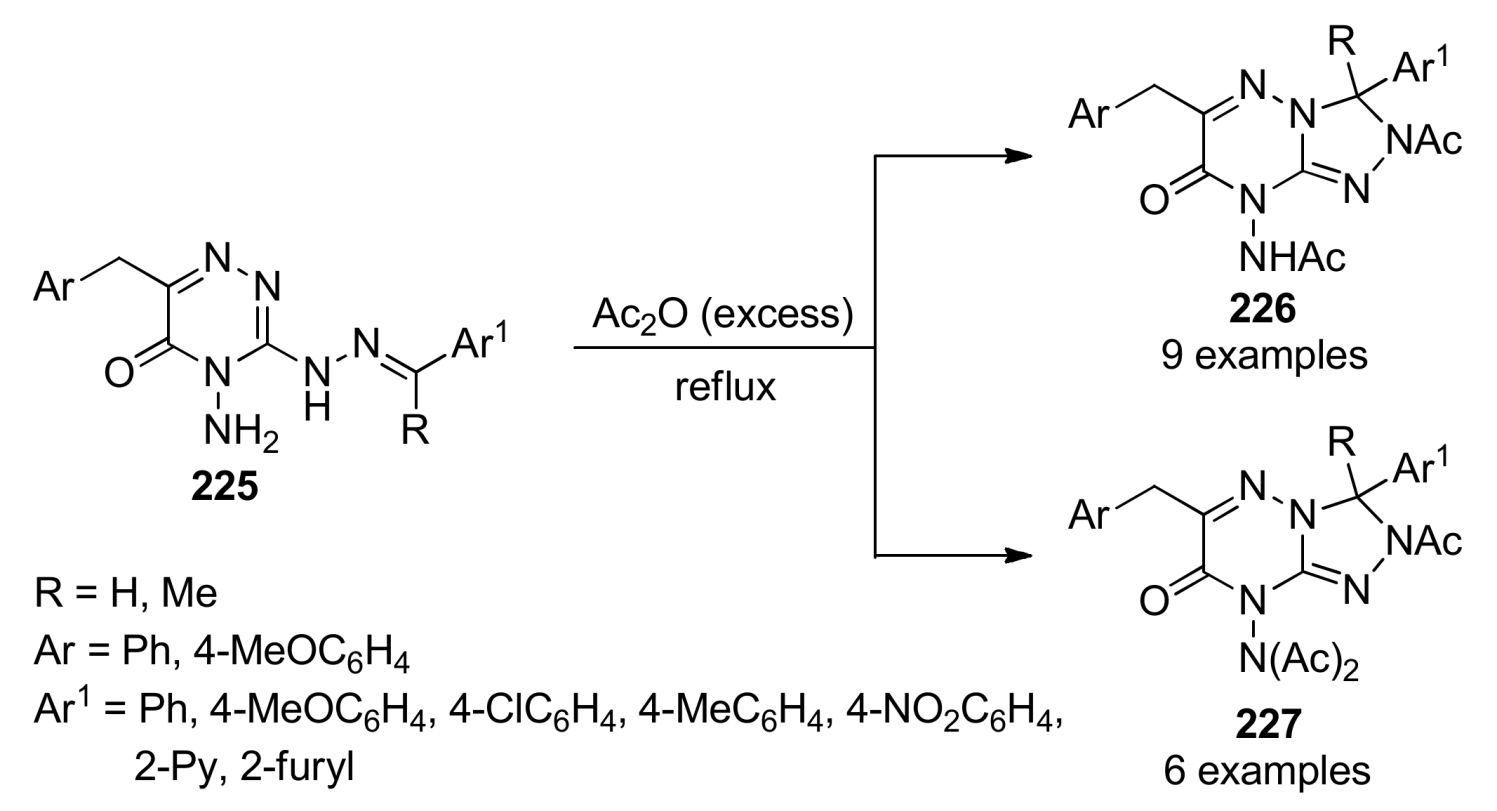
Multicomponent synthesis of polyacetylated fused 1,2,4-triazine derivatives **226**/**227** to be screened their potential toward CPY1A1 inhibition.

Synthesis and structure elucidation of two series of polyacetylated fused 1,2,4-triazine derivatives **226** (68–92% yield) and **227** (65–94% yield) was established by heating the starting Schiff’s bases **225** under an excess of acetic anhydride (Scheme 88). The inhibitory effect of compounds **226** and **227** toward the CPY1A1 activity was screened to determine their potential as promising anticancer drugs. Data showed that compound **226e** (R = Me, Ar = Ph, Ar^1^ = 4-ClC_6_H_4_) displayed the highest inhibitory effects among all tested 1,2,4-triazine derivatives. Furthermore, docking analysis showed that these compounds bind only at the interface of substrate recognition site 2 (SRS2) and (SRS6) at the outer surface of the protein, and that amino-acids ASN214, SER216 and ILE462 participate in the binding of these compounds through H-bonds [226].

**Scheme 89:**
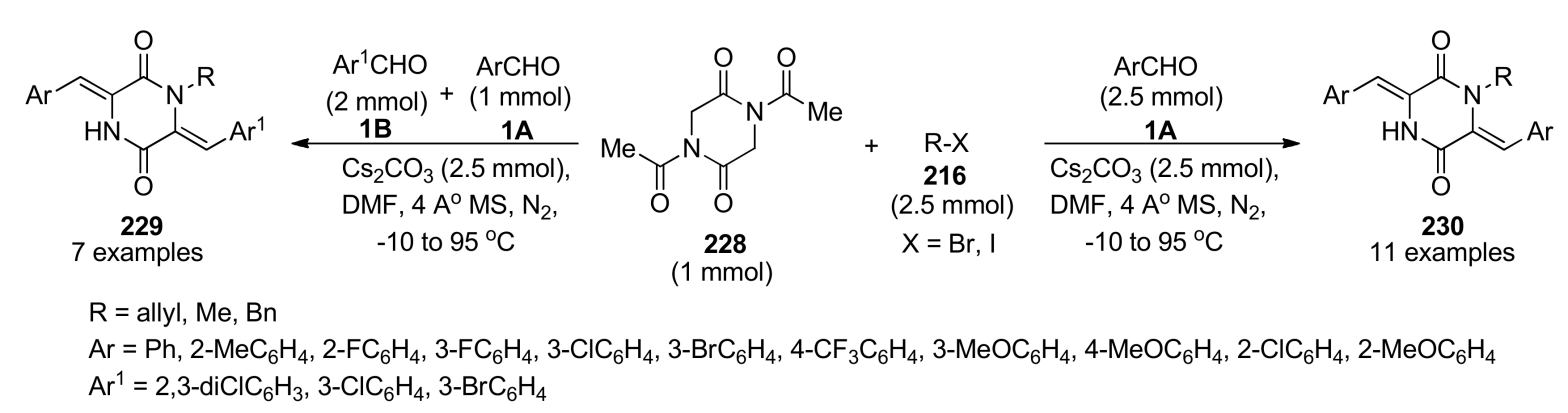
Cs_2_CO_3_-Catalyzed multicomponent synthesis of 1,3,6-trisubstituted 3,6-diunsaturated (*3Z*,*6Z*)-2,5-diketopiperazine derivatives **229**/**230** to be tested in vitro against five cancer cell lines.

A one-pot four-component approach was established for site selective synthesis of 1,3,6-trisubstituted 3,6-diunsaturated (*3Z*,*6Z*)-2,5-diketopiperazine derivatives **229** (38–60% yield) and **230** (31–64% yield), mediated by double-aldolic condensations between pyrazine **228** and two equivalents of aldehydes type **1A/1B**, involving also a mono-alkylation reaction in the same reaction pot, Scheme 89. The computational studies revealed that the steric hindrances between the 2-hydrogen atoms on the aromatic rings and the carbonyl, as well as, the steric repulsions between the hydrogen atoms of the CH group in the benzylidene and the CH_2_ group in the *N*-alkylative part might be responsible for the *Z*/*E* selectivity. The obtained compounds were tested in vitro against five cancer cell lines (i.e., U937, HL60, DU145, HT29 and K562). Results reveled that compound **230h** (R = Me, Ar = 3-MeOC_6_H_4_) (IC_50_ = 11 nM) had a close activity to the positive compound plinabulin (IC_50_ = 15 nM) against the cancer cell line HL60 [227].

**Scheme 90:**
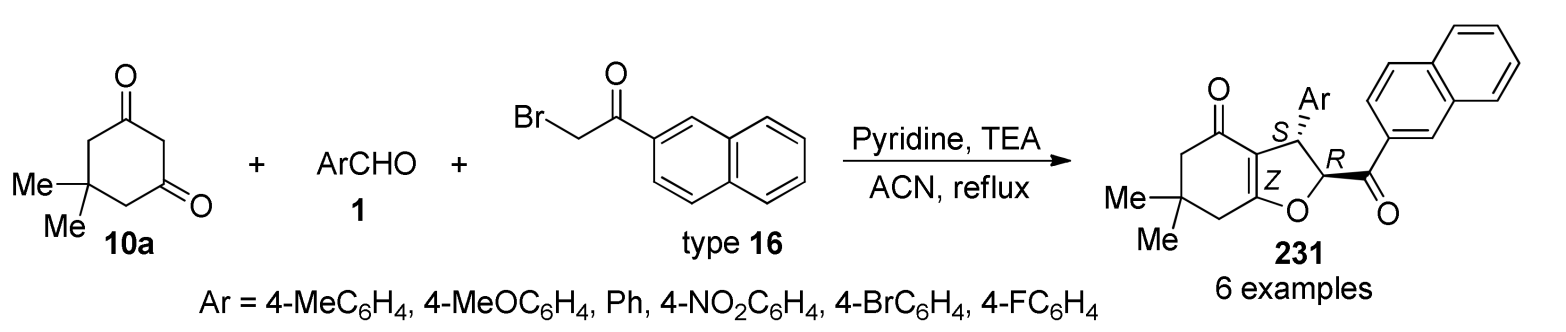
Three-component synthesis of 2-(2-naphthoyl)-6,6-dimethyl-3-aryl-2,3,6,7 tetrahydrobenzofuran-4(5*H*)-ones **231** for evaluation of their potential as inhibitors of cathepsins B, H and L.

A one-pot three-component synthesis of some 2-(2-naphthoyl)-6,6-dimethyl-3-aryl-2,3,6,7 tetrahydrobenzofuran-4(5*H*)-ones **231** in 84–92% yield is described in Scheme 90. 

The synthesized compounds **231** were found to be potential inhibitors to cathepsins B, H and L. The extent of inhibition varied with the substitution. Among the synthesized compounds, derivative **231d** (Ar = 4-NO_2_C_6_H_4_) was selected as most inhibitory to cathepsin H, however compound **231f** (Ar = 4-FC_6_H_4_) was the best inhibitor of cathepsin B and cathepsin L. In vitro inhibition studies correlated well when tested using MTT assay on HepG2 cells, a hepatocellular carcinoma cell line. The results validated by in silico studies performed with iGemDock predicted that among the synthesized compounds, **231d** experienced the highest affinity for cathepsin B and H sites, whereas **231f** had the highest affinity to cathepsin L [228].

**Scheme 91:**
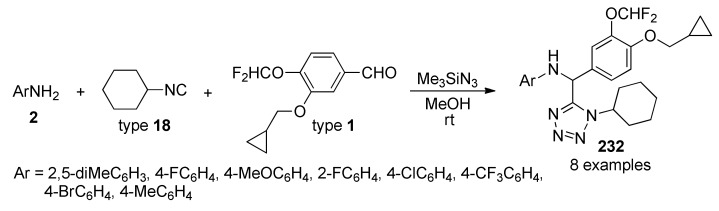
Ugi-Mediated multicomponent synthesis of tetrazole scaffolds **232** to be evaluated by the NCI for their in vitro cytotoxicity against sixty different cancer cell lines.

In the current research scenario, an efficient synthesis of tetrazole scaffolds **232** in 93–96% yield was developed by a single step four-component reaction. The synthesis of the target compounds **232** was undertaken by the Ugi multicomponent approach with the condensation of various aryl amines **2**, TMS-N_3_, cyclohexyl isocyanide **18** and aromatic aldehyde containing active pharmacophore **1**, under catalyst-free reaction condition at room temperature (Scheme 91). The potency of the obtained tetrazoles **232** was checked at the NIH using sixty different cell-lines with respect to nine cancer panels, among which compounds **232a** (Ar = 2,5-diMeC_6_H_3_) and **232b** (Ar = 4-FC_6_H_4_) displayed the higher activity against different cell lines [229].

**Scheme 92:**
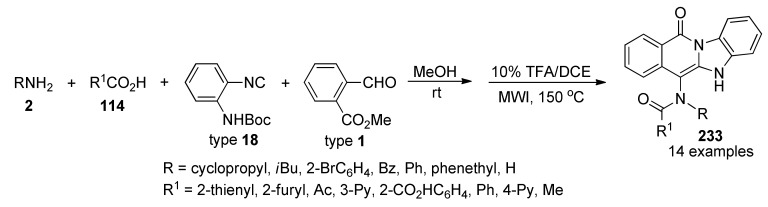
Ugi-Mediated multicomponent synthesis of benzimidazole-isoquinolinone derivatives (BIDs) **233** for screening to identify novel scaffolds for *Colorectal* cancer CRC.

A series of benzimidazole-isoquinolinone derivatives (BIDs) **233** was synthesized in 52–82% yield. The process was conducted by an Ugi four-component reaction (U-4CR) using an amine **2**, carboxylic acid **114**, isonitrile **18**, and the methyl 2-formylbenzoate type **1** in methanol at room temperature (Scheme 92). Subsequently, the solvent was removed under a stream of nitrogen, and the intermediate crude Ugi product obtained was deprotected and cyclized in situ under MWI using 10% TFA/DCE at 150 °C. Further, the target products **233** were screened to identify novel scaffolds for *Colorectal* cancer CRC. Among the compounds evaluated, **233g** (R = *i*Bu, R^1^ = 3-Py) exhibited the most promising anti-cancer properties. Employing two CRC cell lines, SW620 and HT29, **233g** was found to suppress growth and proliferation of the cell lines at a concentration of ∼20 μM. Treatment followed an increase in G2/M cell cycle arrest, which was attributed to cyclin B1 and cyclin-dependent kinase 1 (CDK1) signaling deficiencies with simultaneous enhancement in p21 and p53 activity. In addition, mitochondrial mediated apoptosis was induced in CRC cells. Interestingly, **233g** decreased phosphorylated AKT, mTOR and 4E-BP1 levels, while promoting the expression/stability of PTEN [230].

Series of diverse thiophene, pyran, azole and azine derivatives **234**–**242** in 63–89% yield were synthesized from cyclopentanone type **16** as common starting material, which reacted with malononitrile and ethyl cyanoacetate (**12**)**/**(**38**), among other reagents (Table 5 and Table 6). The biological evaluation of the obtained compounds **234**–**242** was tested on three different tumor cell lines such as breast adenocarcinoma, *CNS* cancer, and non-small lung cancer and was compared to the inhibitory effect of doxorubicin. The results revealed that, among the heterocyclic products, furan derivatives **234e** (Ar = 4-MeOC_6_H_4_, Y = CONH_2_, X = NH_2_) and **234f** (Ar = 2-furyl, Y = CN, X = OH) had the highest inhibitory effect [231].

**Scheme 93:**
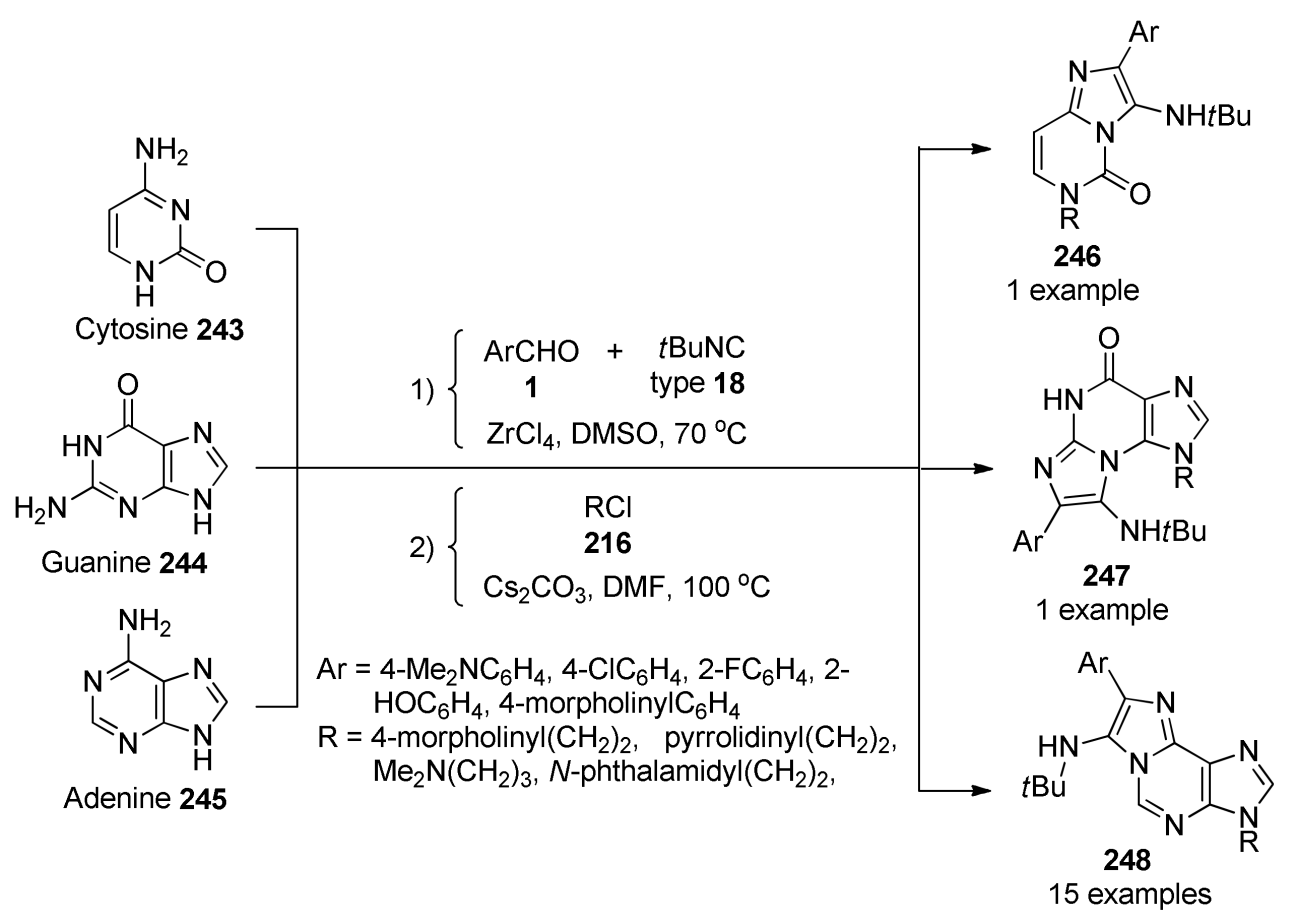
Groebke-Blackburn-Bienaymé-mediated three-component synthesis of imidazo[2,1-*i*]purine derivatives **246**–**248** for the identification of dual BCL2/c-MYC gene promoter G-quadruplex ligands.

Finally, in the search for new drug-like selective G-quadruplex binders, a bioinspired design focused on the use of nucleobases as synthons in a multicomponent reaction was provided by Pelliccia et al. [232]. Thus a series of multifunctionalized imidazo[2,1-*i*]purine derivatives **246** (57% yield), **247** (28% yield) and **248** (22–84% yield) were synthesized via a convergent Groebke-Blackburn-Bienaymé three-component reaction (GBB-3CR) of amino-aza-heterocycles **243**–**245**, benzaldehydes **1**, and isocyanide **18**, followed by a S_N_2 with aminoalkyl chlorides type **216** (Scheme 93).

Biophysical studies over products **246**–**248** allowed for the identification of the first dual BCL2/c-MYC gene promoter G-quadruplex ligand, which involved circular dichroism melting experiments, microscale thermophoresis measurements, NMR titrations, and computational docking calculations, as well as biological investigations including cytotoxicity and apoptotic assays, and quantitative polymerase chain reaction and Western blot analyses. Resulst permitted to assess the potency and to characterize the binding mode of the newly identified lead compound. The absence of toxicity toward normal cells, together with the small molecular weight (≅500 Da), the water solubility, the ease of functionalization, and the selectivity profile, showed to be promising and desirable features to develop G-quadruplex binders as safe and effective anticancer agents.

## 4. Conclusions

Through this review it has been underscored the tremendous potential of MCRs as a very important tool for the synthesis of a vast number of organic acyclic and heterocyclic molecules, coupled to the prospect that many of them are biologically active or at least have been submitted to any biological screen. After a trip through the most relevant literature dealt with libraries of organic molecules synthesized via MCRs and subjected to screening for biological activity, it was found that most of such screening were addressed to anticancer, antimicrobial, anti-leihsmanial, anti-inflammatory, ROCK inhibitor, antioxidant, antimycobacterial, bromodomain inhibitor, antifibrotic agents, human receptor 8-active, neuroprotective agents, acetylcholinesterase inhibitor and anti-HIV activities. Interestingly, more than 60% of the found literature was oriented to anticancer activity evaluation. Thus, after the searching for appropriated reports to support the subject of this review we could establish that MCRs still represent an excellent synthetic strategy for the generation of a vast number of organic scaffolds, suitable for structure–activity relationship (SAR) studies in Medicinal Chemistry and drug discovery programs.

## Figures and Tables

**Table 1:** Synthesis of dihydropyrimidone(thione)s **152**–**156** of antiproliferative interest mediated by Biginelli type three-component reactions.

Reagents			Conditions	Product	Reference
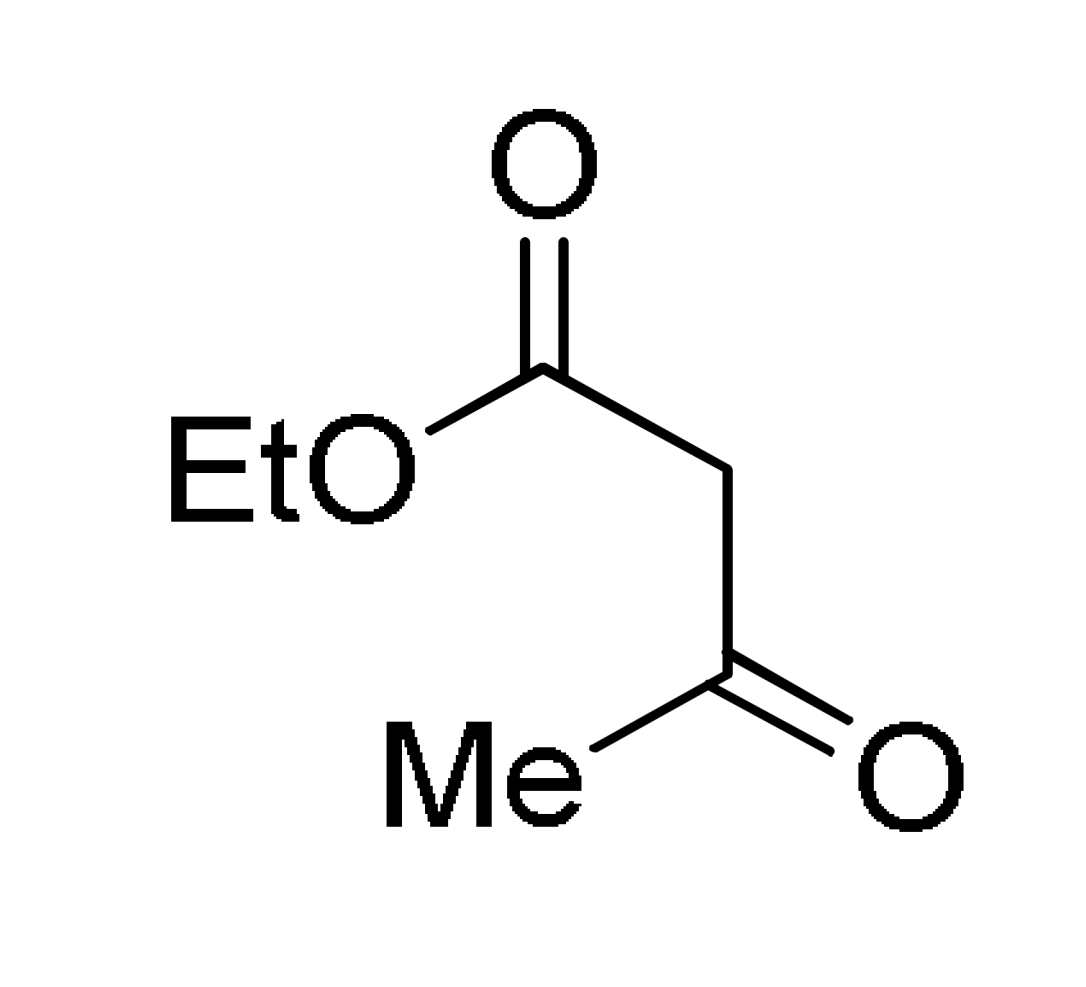 type **8**	RCHO type **1**	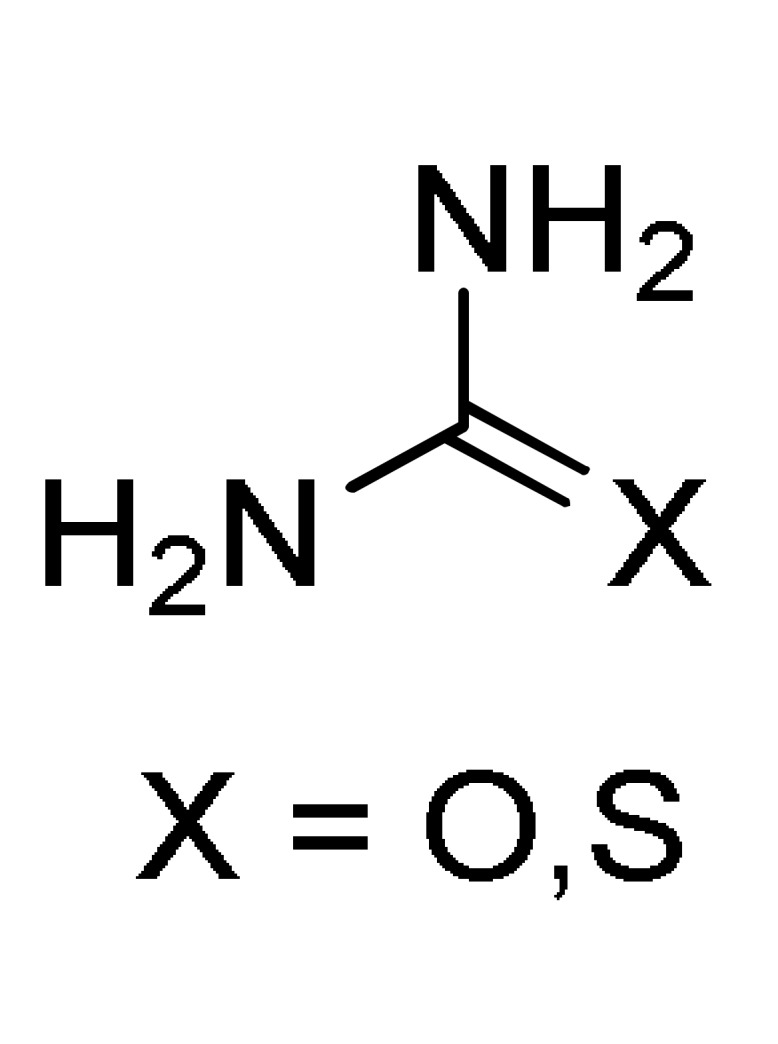 **6**	*p*-sulfonic acid calix[4]arene (0.5% mol), EtOH, reflux	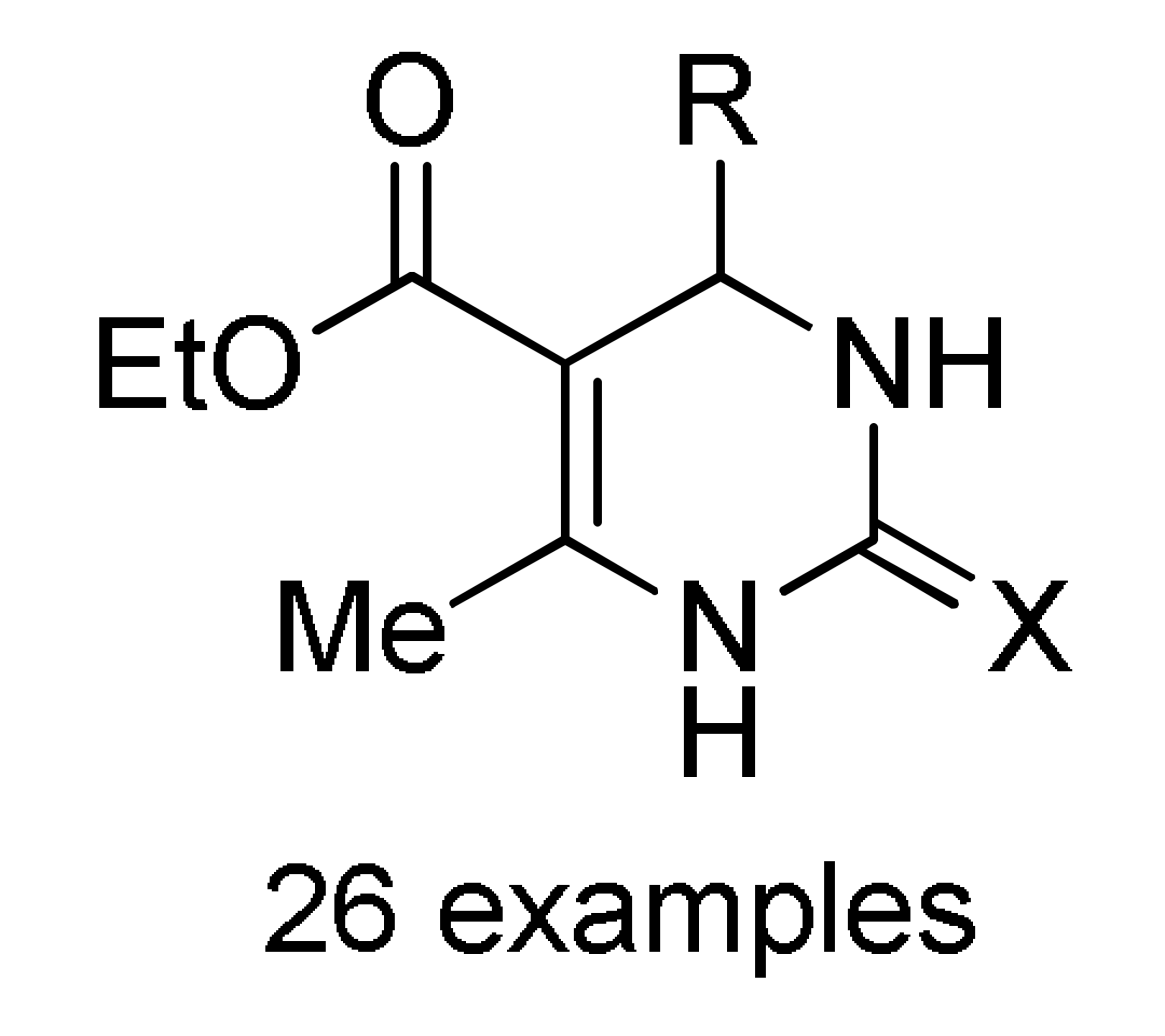 **152** ^a^	[169]
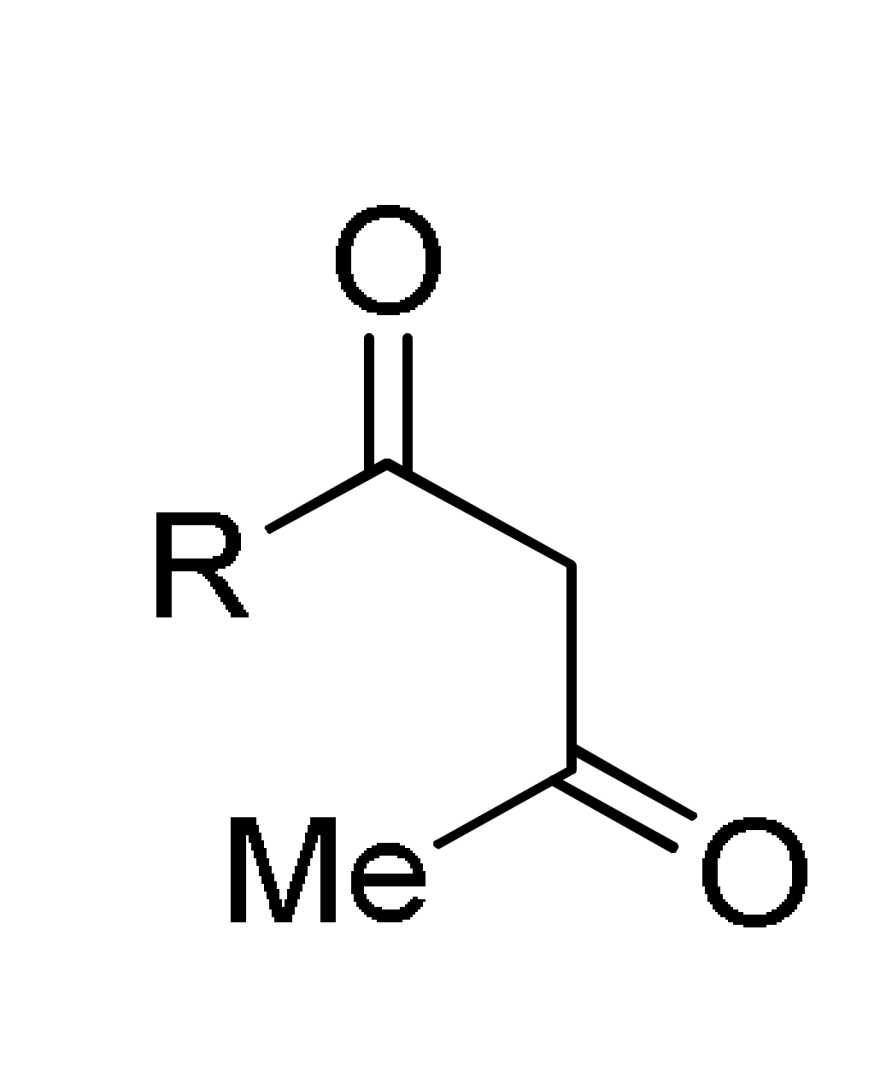 type **27**	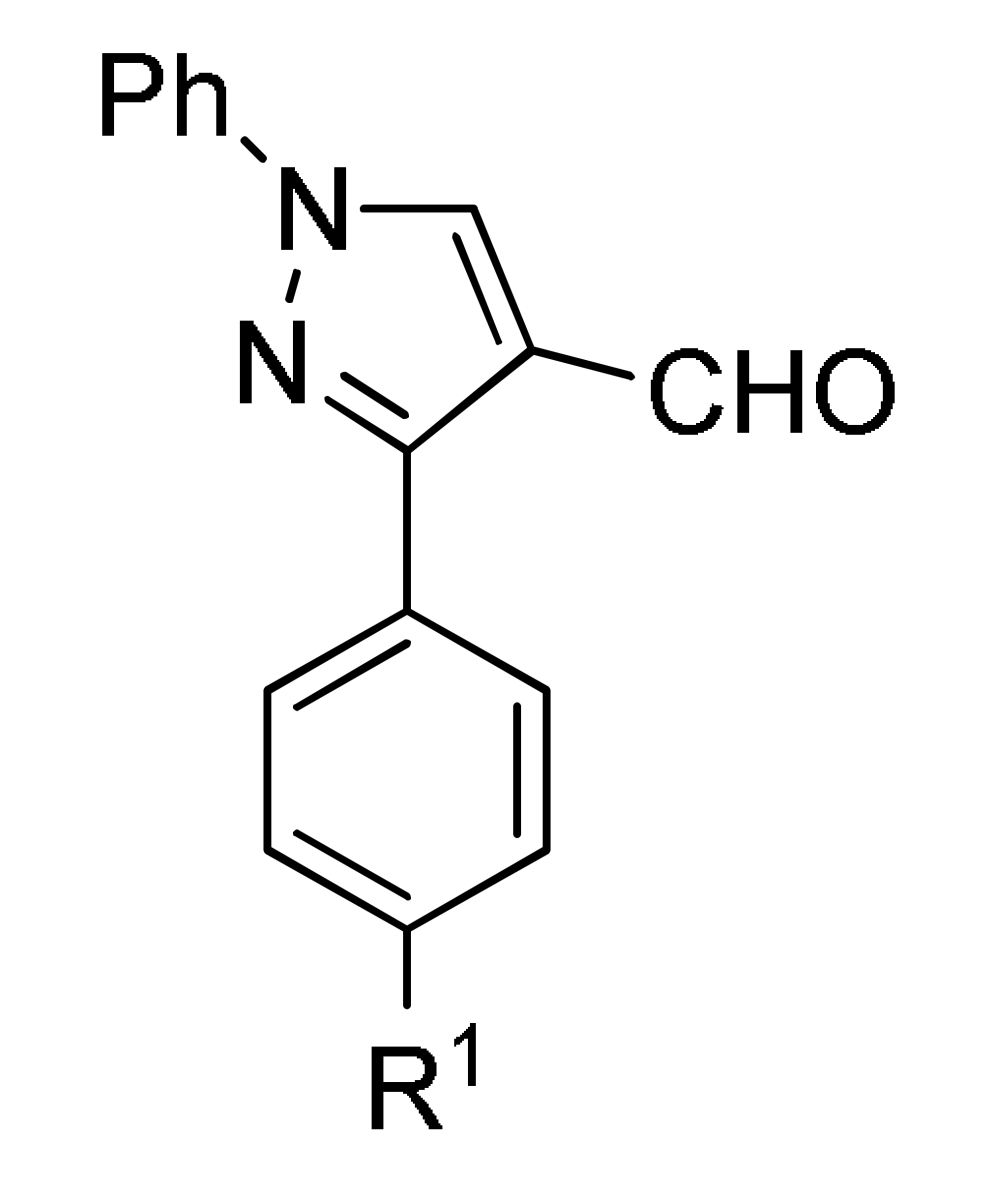 type **1**		*p*-sulfonic acid, EtOH, reflux	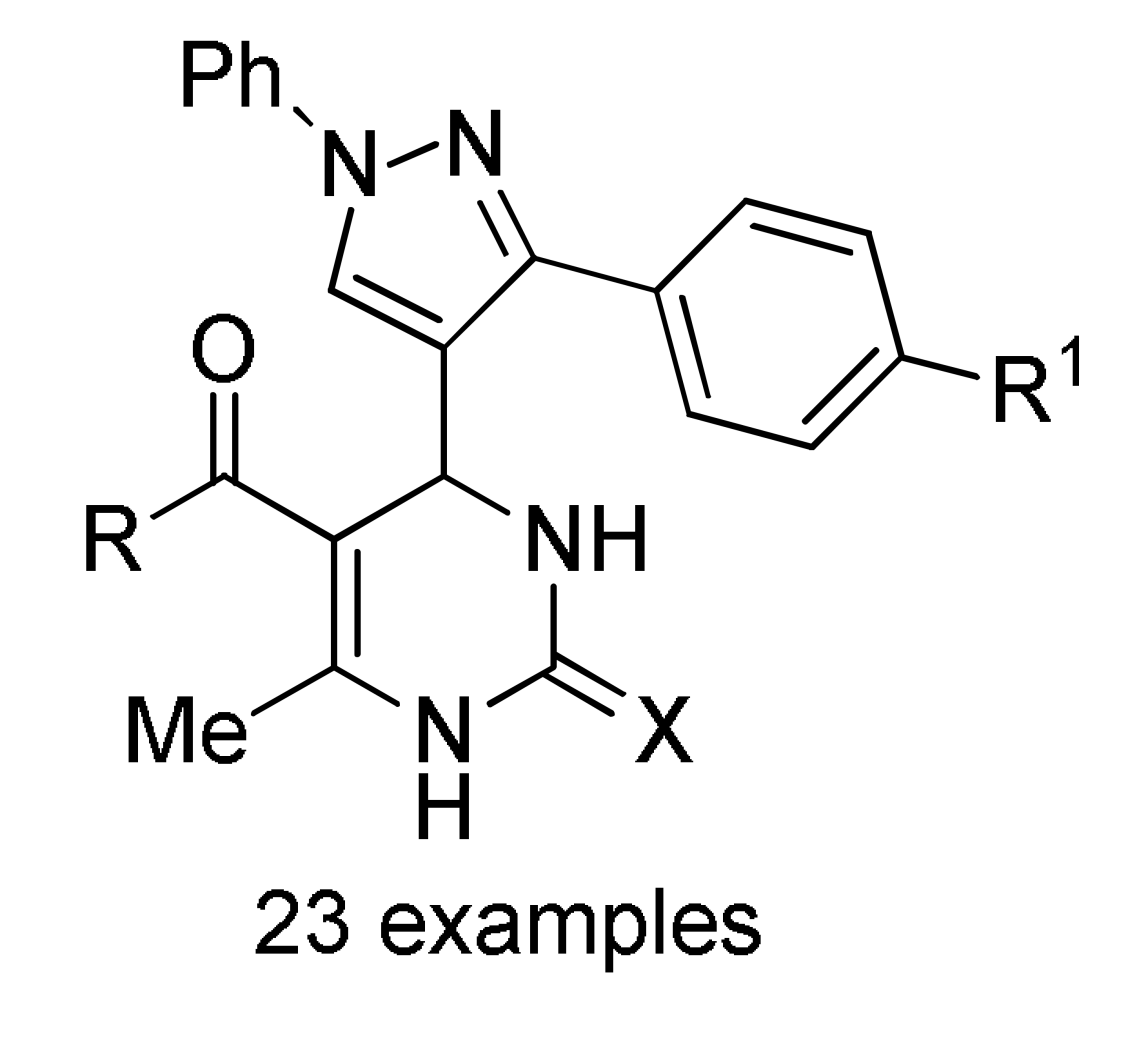 **153** ^b^	[169]
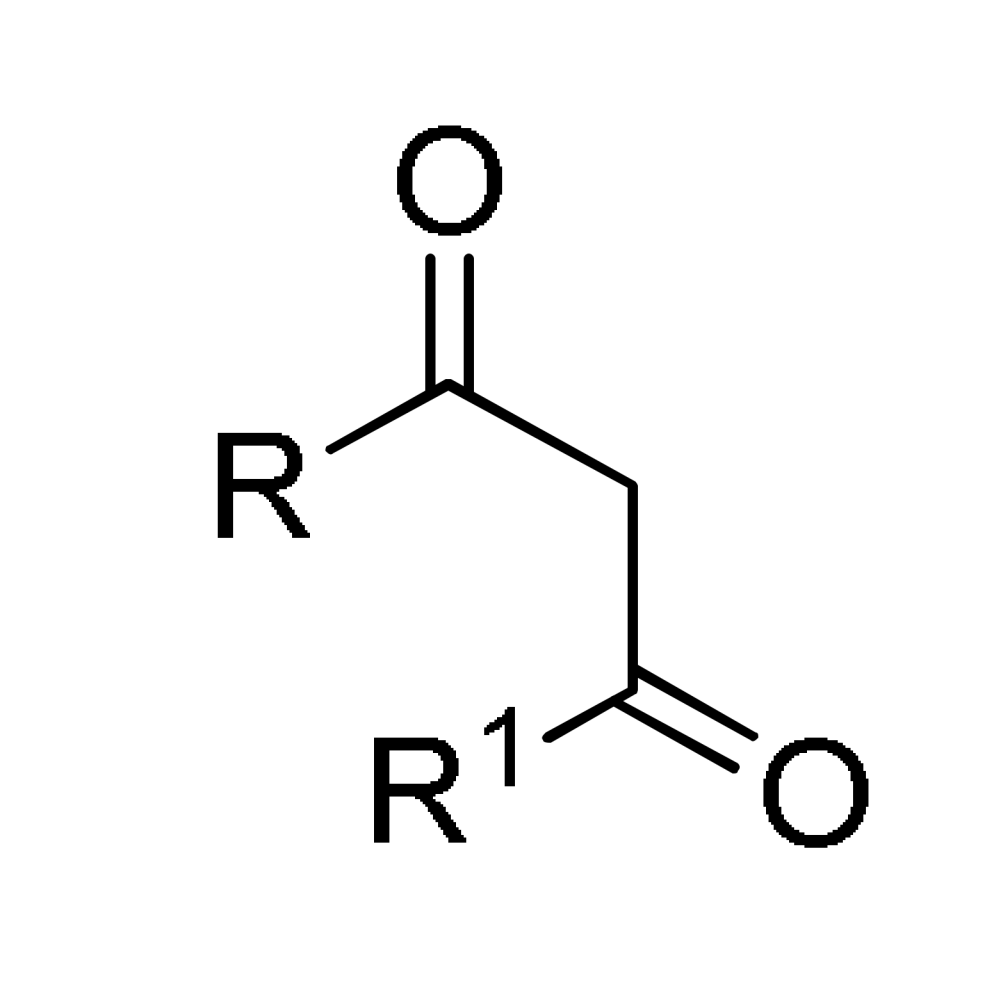 type **27**	ArCHO/R^2^CHO **1**		MAI·Fe_2_Cl_7_ (5 mol%), BMI·BF_4_ IL (1 mL), 80 °C	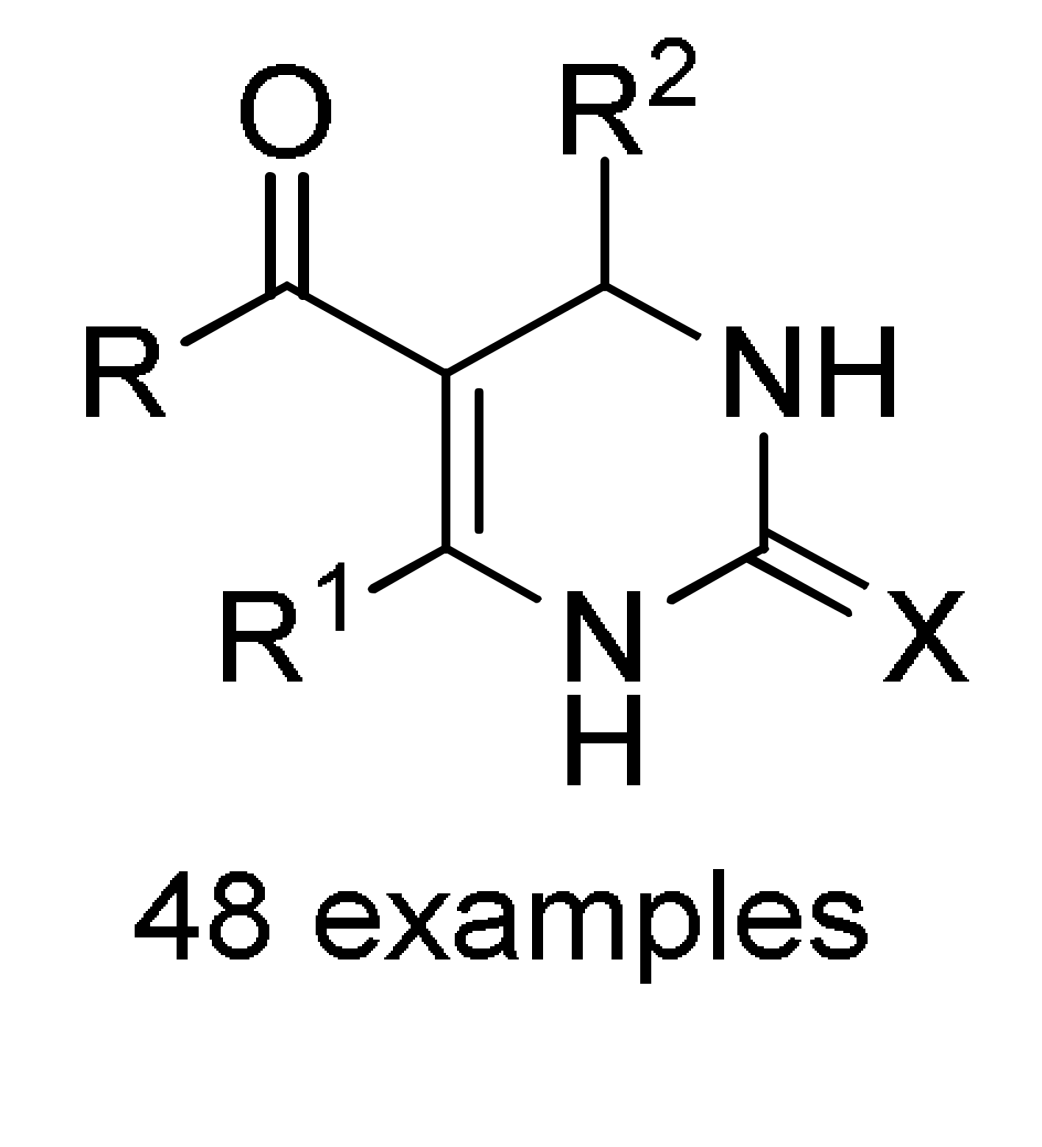 **154** ^c^	[170]
			Heterogeneous Zn- and Cd-based CPs catalysts (5 mol%), 70–100 °C, Continuous flow	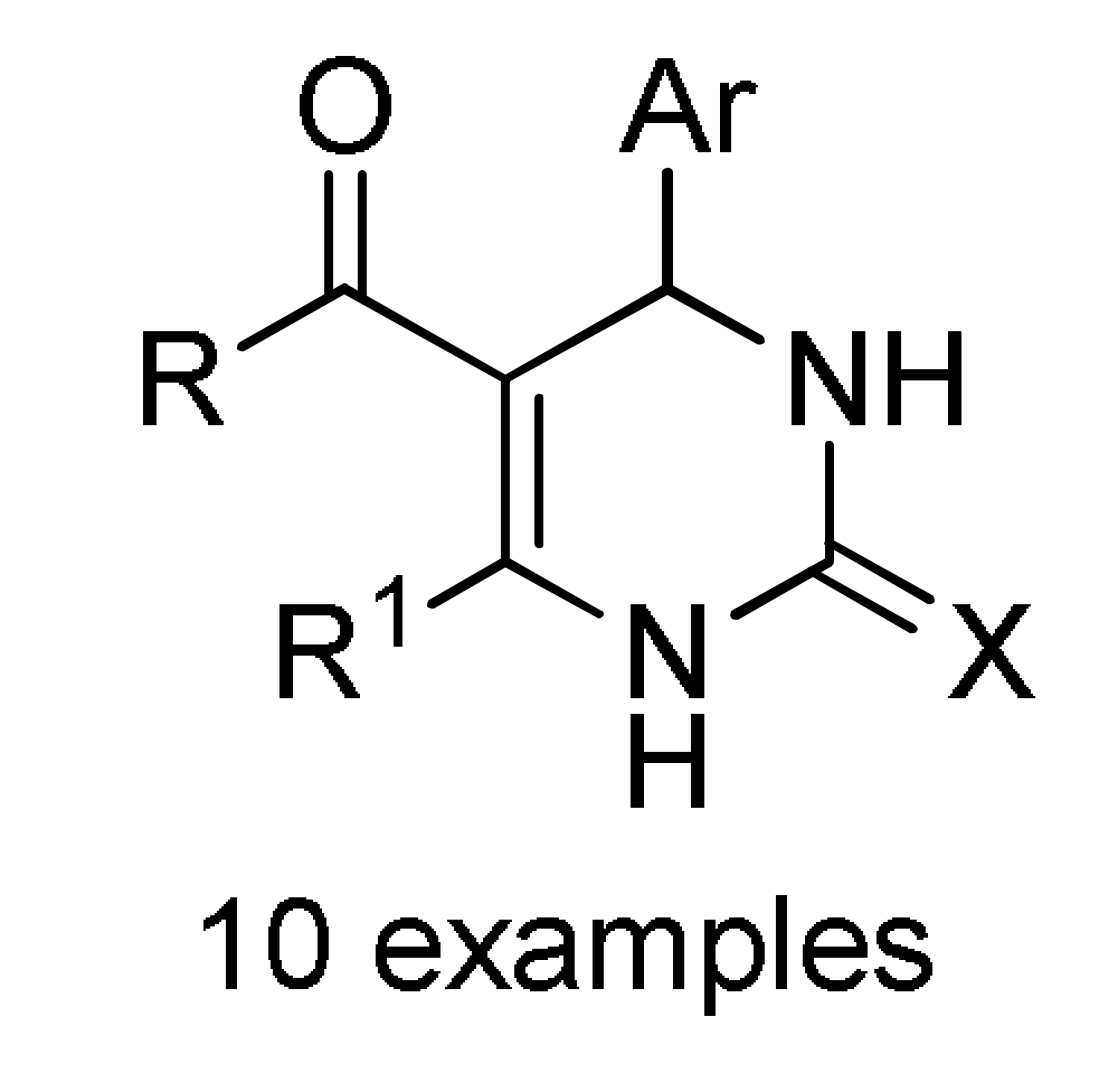 **155** ^d^	[171]
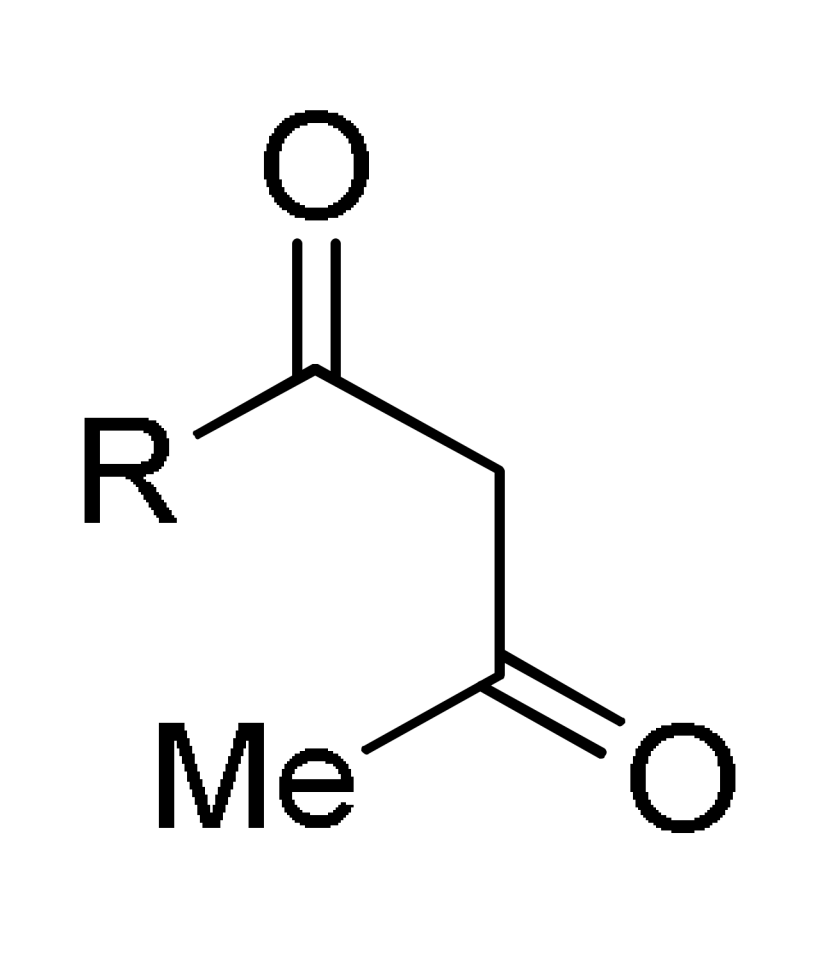 type **27**		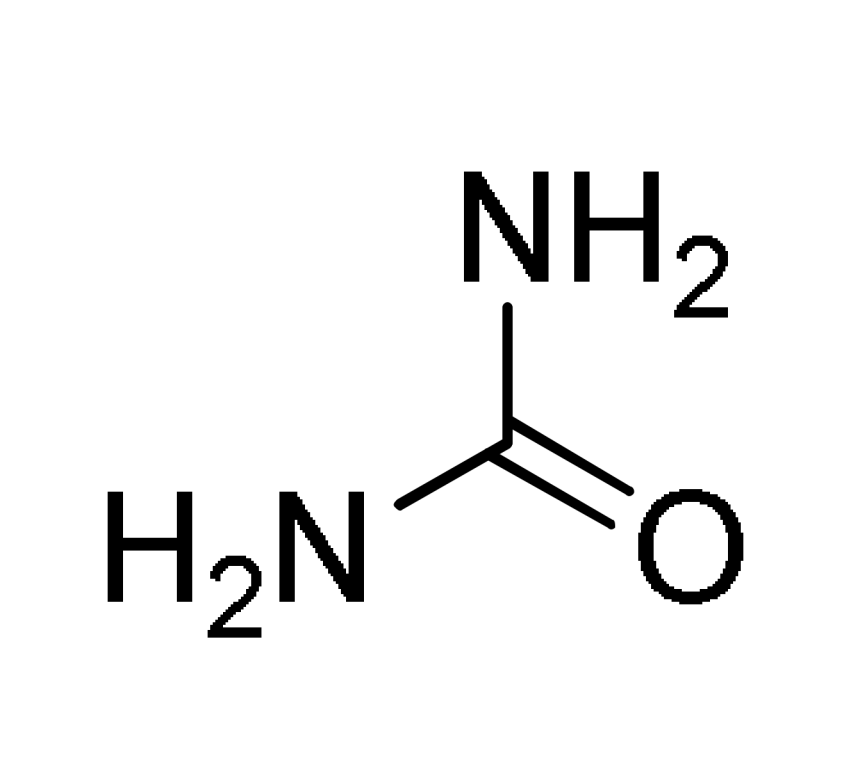 type **6**	Bi(NO_3_)_3_.5H_2_O, 70 °C, solvent free	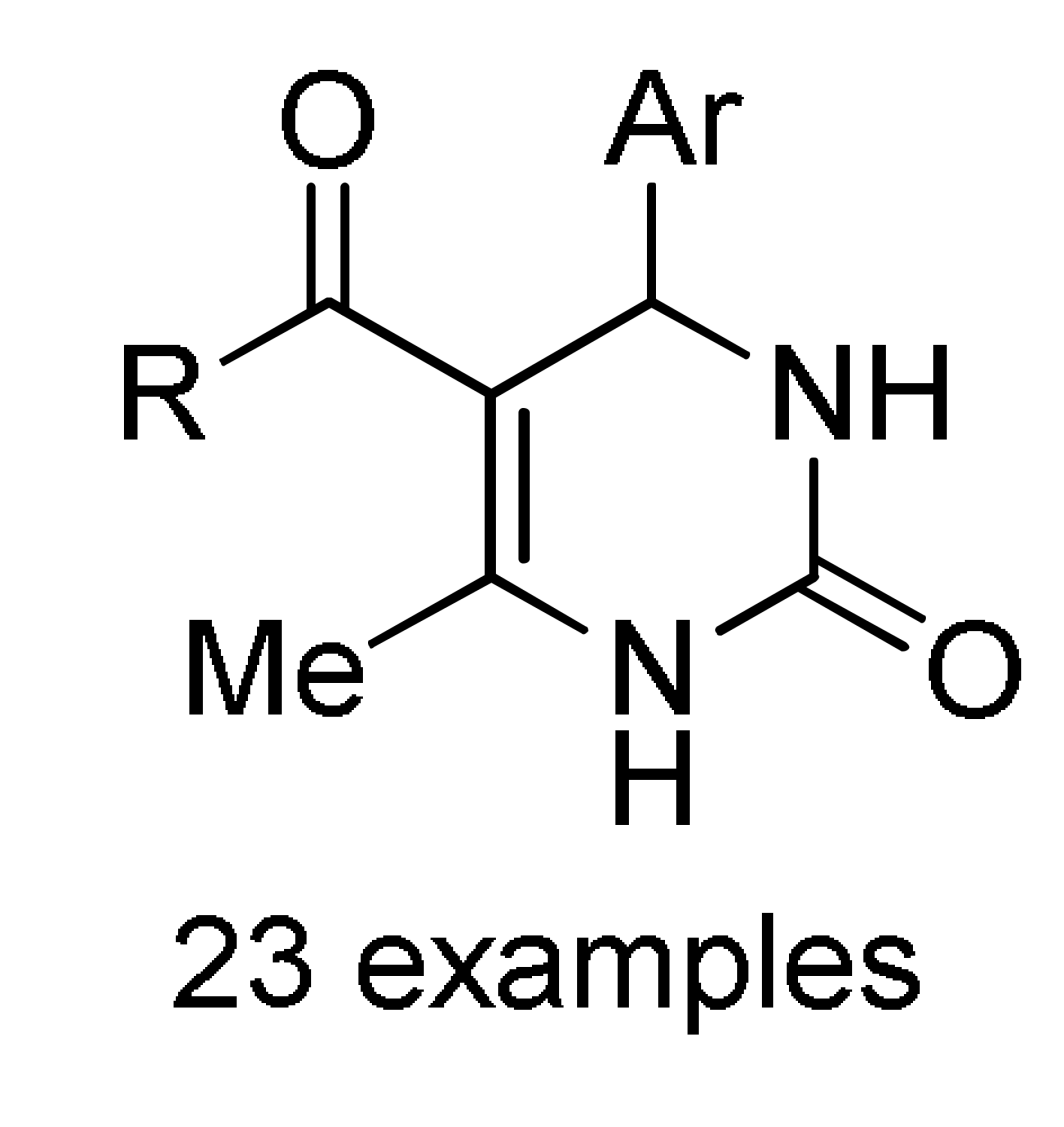 **156** ^e^	[172]

^a^ For compounds **152**: X = O, S; R = 3-HOC_6_H_4_, Ph, 4-HOC_6_H_4_, 3,4-(HO)_2_C_6_H_3_, 4-MeOC_6_H_4_, 3-MeOC_6_H_4_, 4-HO-3-MeOC_6_H_3_, 4-HO-3,5-(MeO)_2_C_6_H_2_, 4-MeSC_6_H_4_, 3,4-(OCH_2_O)C_6_H_3_, 4-FC_6_H_4_, Pr, cyclohexyl. ^b^ For compounds **153**: X = O, S; R = OEt, PhNH, 4-MeC_6_H_4_NH, 4-MeOC_6_H_4_NH, 4-ClC_6_H_4_NH, 2-ClC_6_H_4_NH, 4-NO_2_C_6_H_4_NH; R^1^ = H, Cl. ^c^ For compounds **154**: X = O, S; R = OEt, Me; R^1^ = Me; R^2^ = Ph, 4-ClC_6_H_4_, 3-HOC_6_H_4_, 2-HOC_6_H_4_, 3-NO_2_C_6_H_4_, 2-NO_2_C_6_H_4_, 4-HO-3-MeOC_6_H_3_, H, Me, 2-furyl, 3,4-(OCH_2_O)C_6_H_3_. ^d^ For compounds **155**: X = O, S; R = OEt; R^1^ = Me; Ar = Ph, 3-HOC_6_H_4_, 3-NO_2_C_6_H_4_, 4-HO-3-MeOC_6_H_3_, 3,4-(OCH_2_O)C_6_H_3_. ^e^ For compounds **156**: R = OMe, OEt, Me; Ar = Ph, 4-MeOC_6_H_4_, 4-MeC_6_H_4_, 4-NO_2_C_6_H_4_, 2,4-(Cl)_2_C_6_H_3_, 2,3-(Cl)_2_C_6_H_3_, 2,3-(F)_2_C_6_H_3_, 2-furyl.

**Table 2:** Multicomponent synthesis of dihydropyridine-based heterocyclic systems **157**–**161** of potential anticancer activity.

Reagents				Conditions	Product	Reference
Ar^1^CHO **1**	ArCOMe **16**	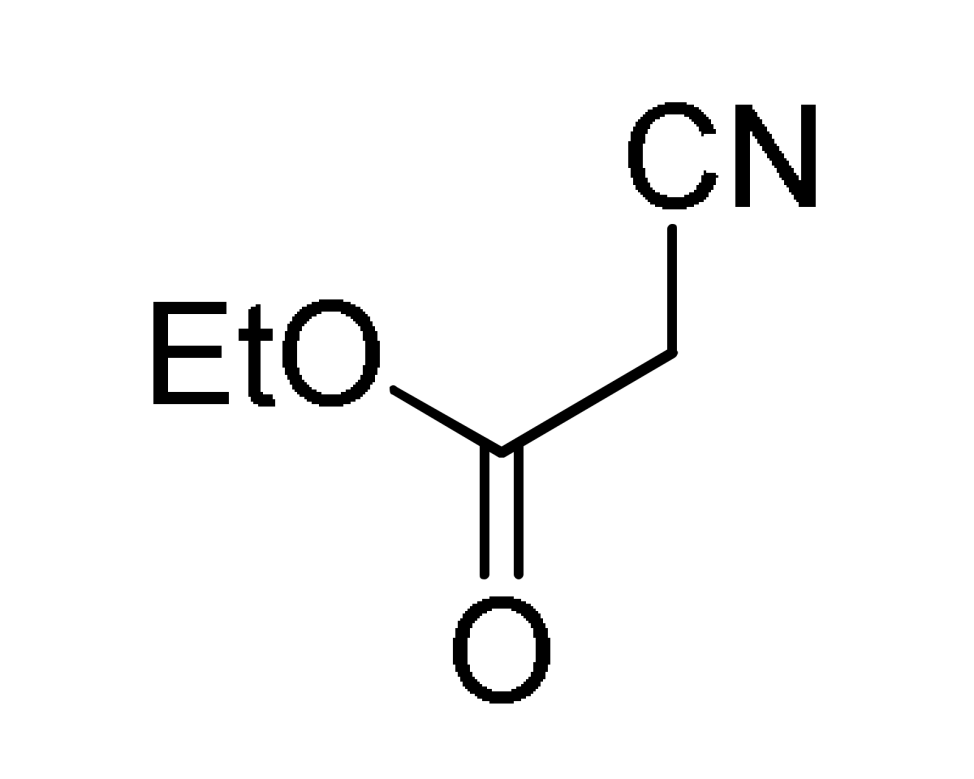 **38**	AcONH_4_	EtOH, reflux	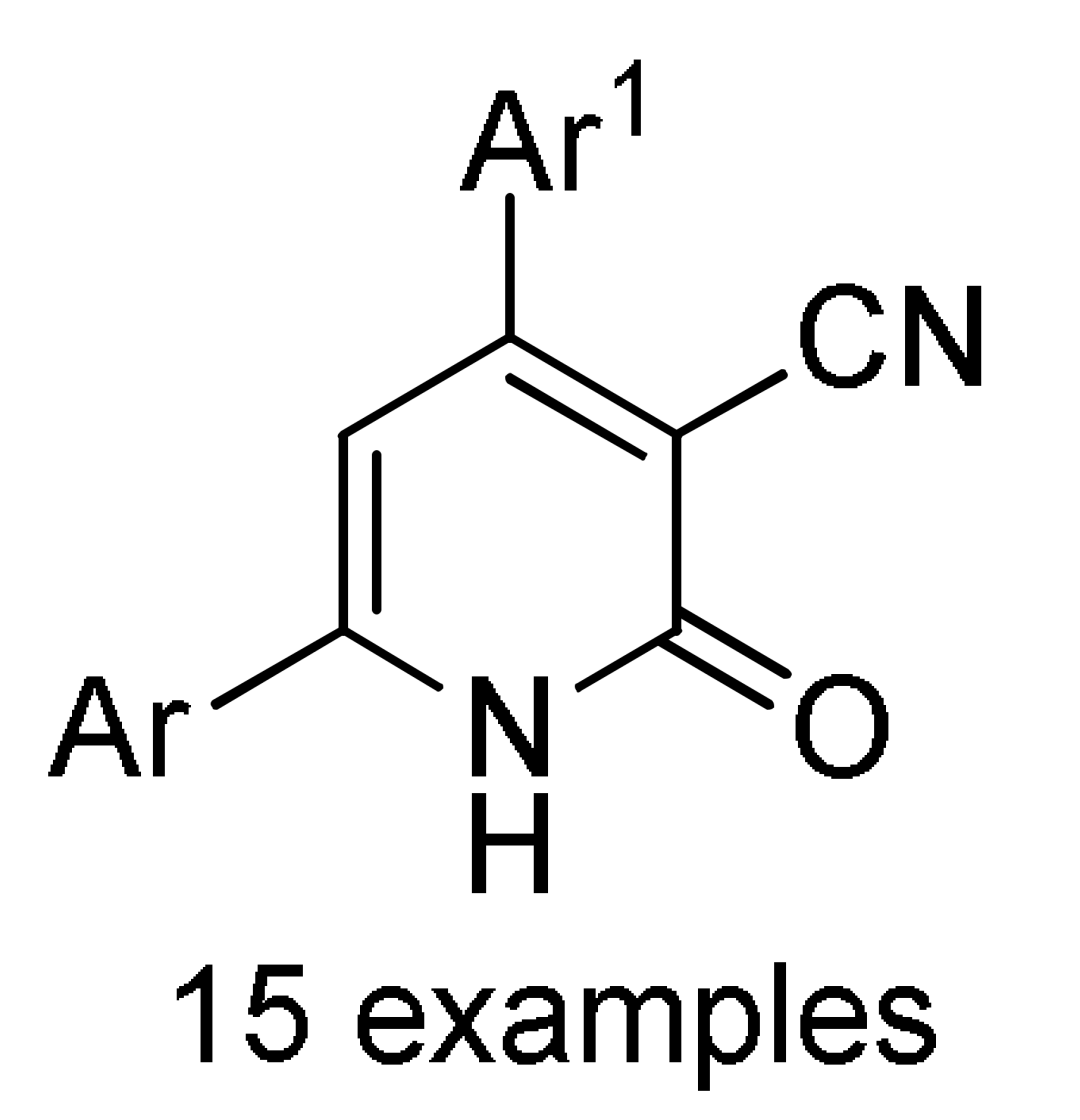 **157** ^a^	[176]
		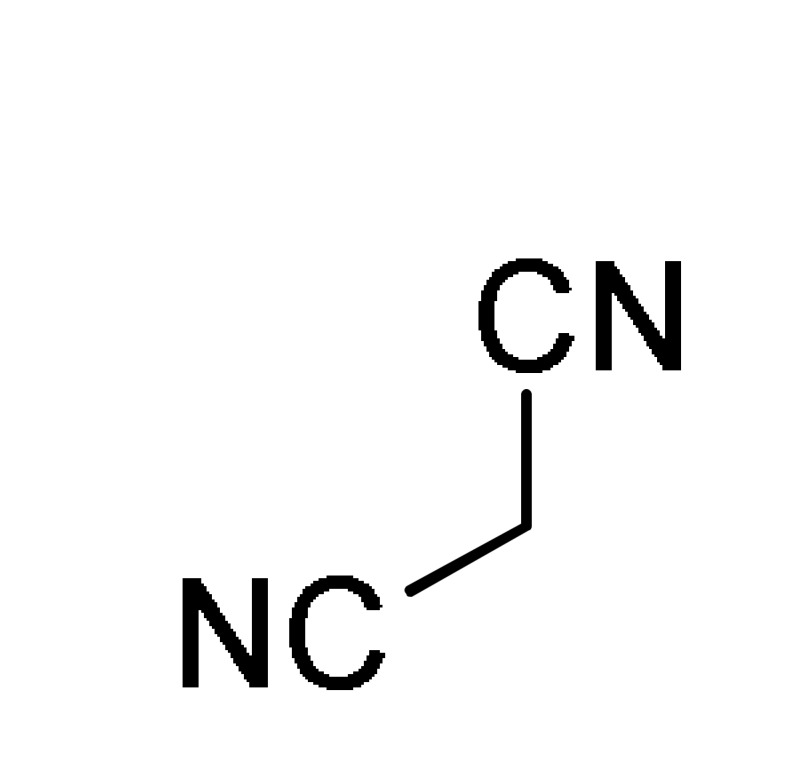 **12**			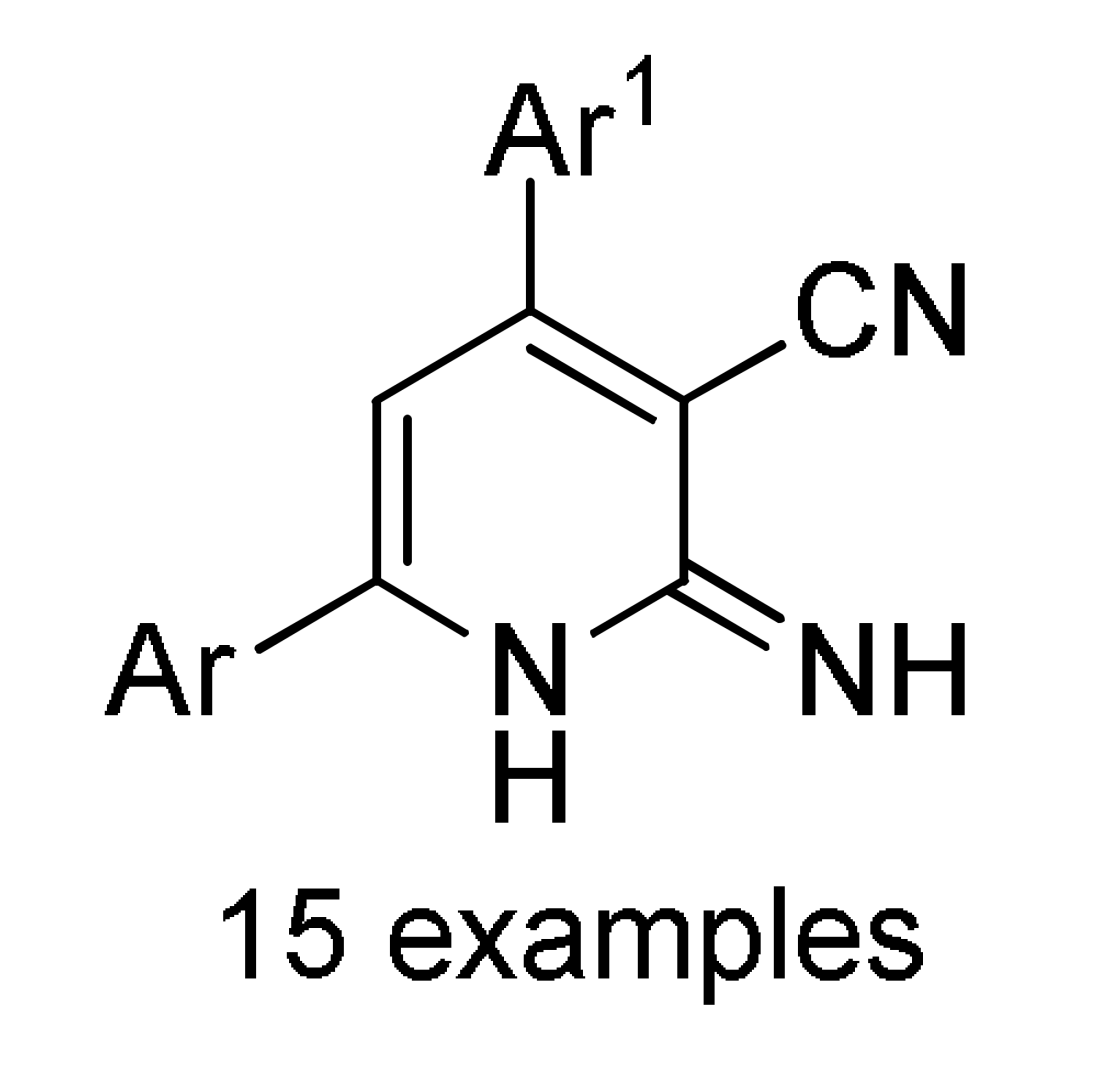 **158** ^a^	
	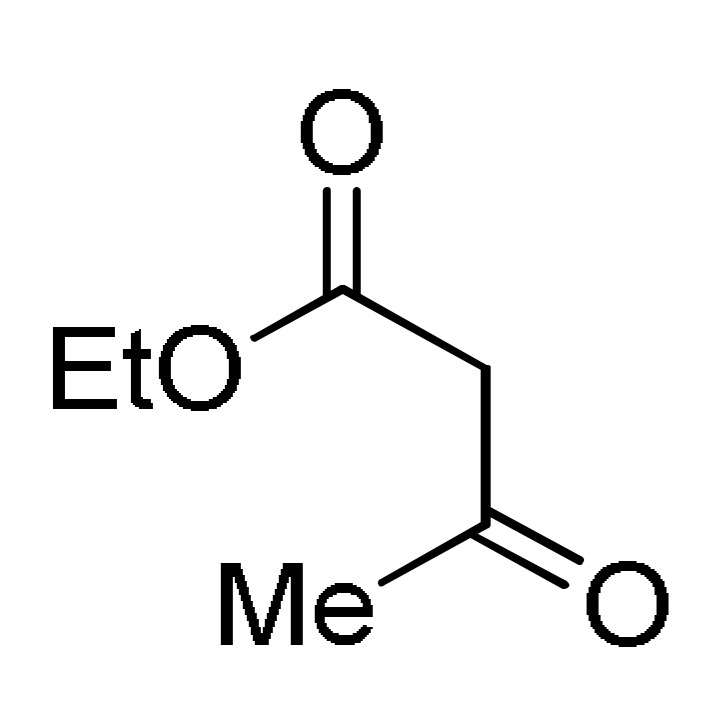 **8**	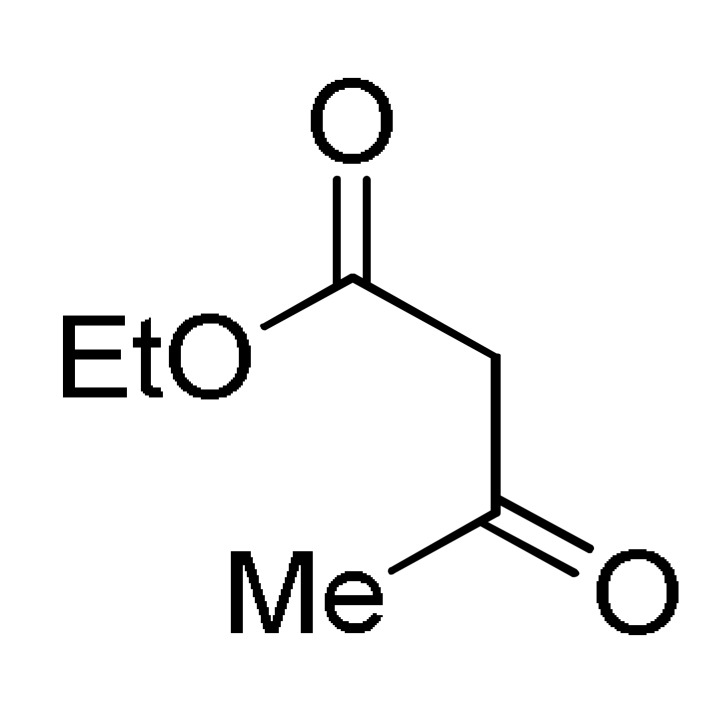 **8**	ArNH_2_ **2**	PEG-400, 200 W, 80 °C	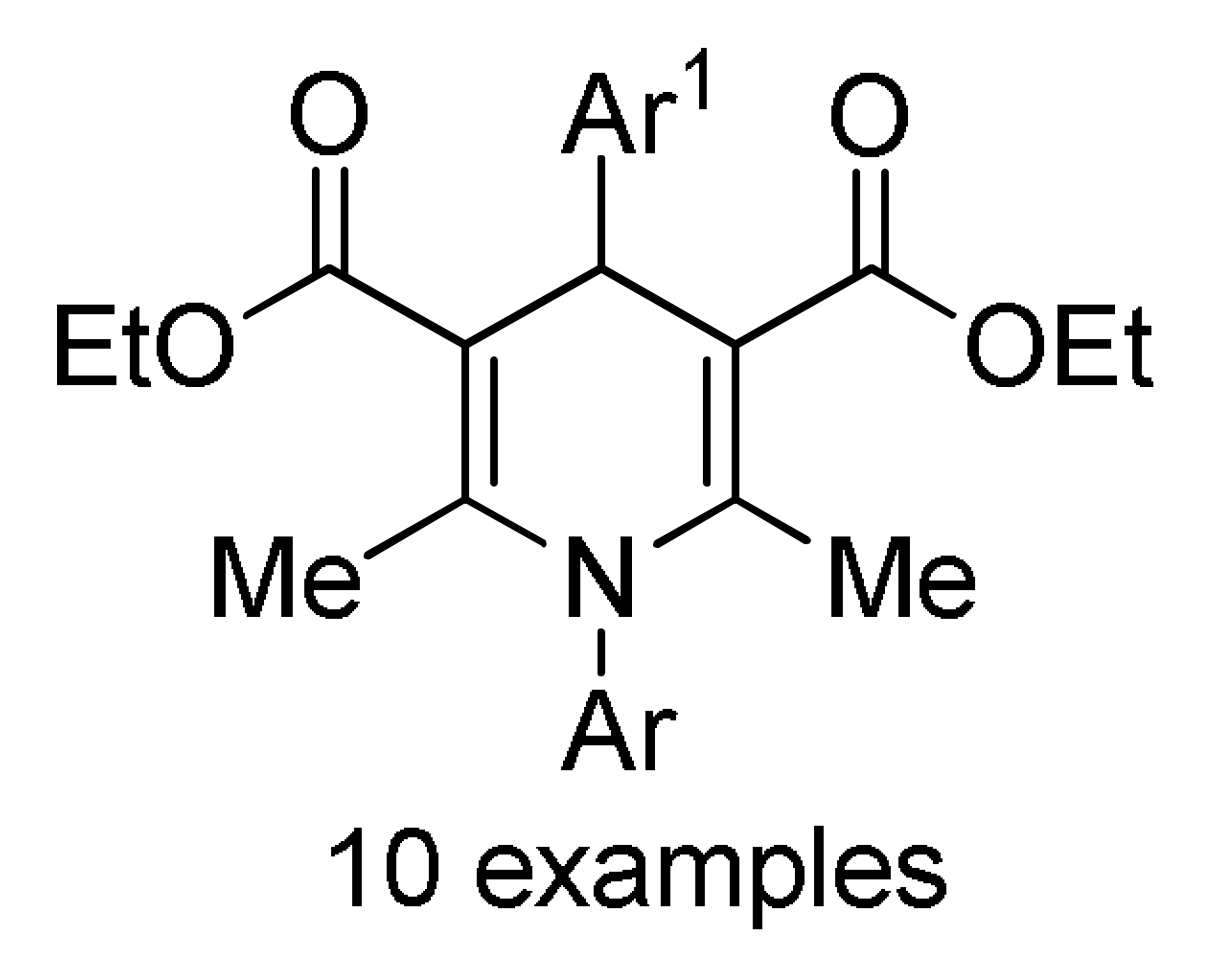 **159** ^b^	[177]
	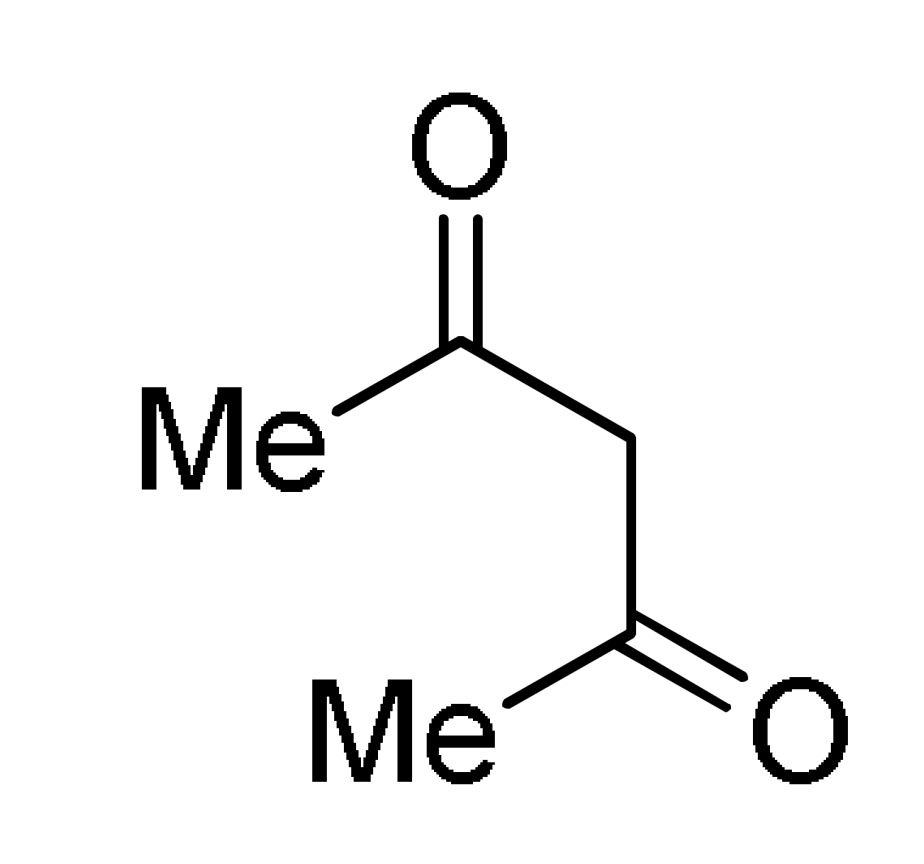 **27**	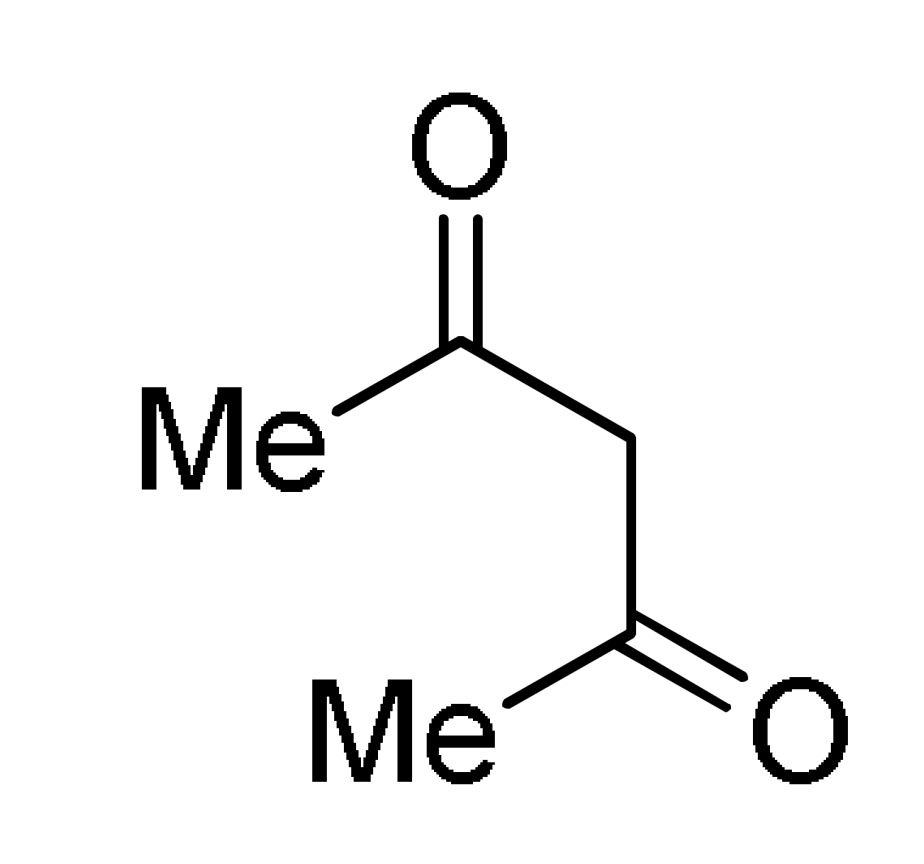 **27**	(NH_4_)_2_CO_3_		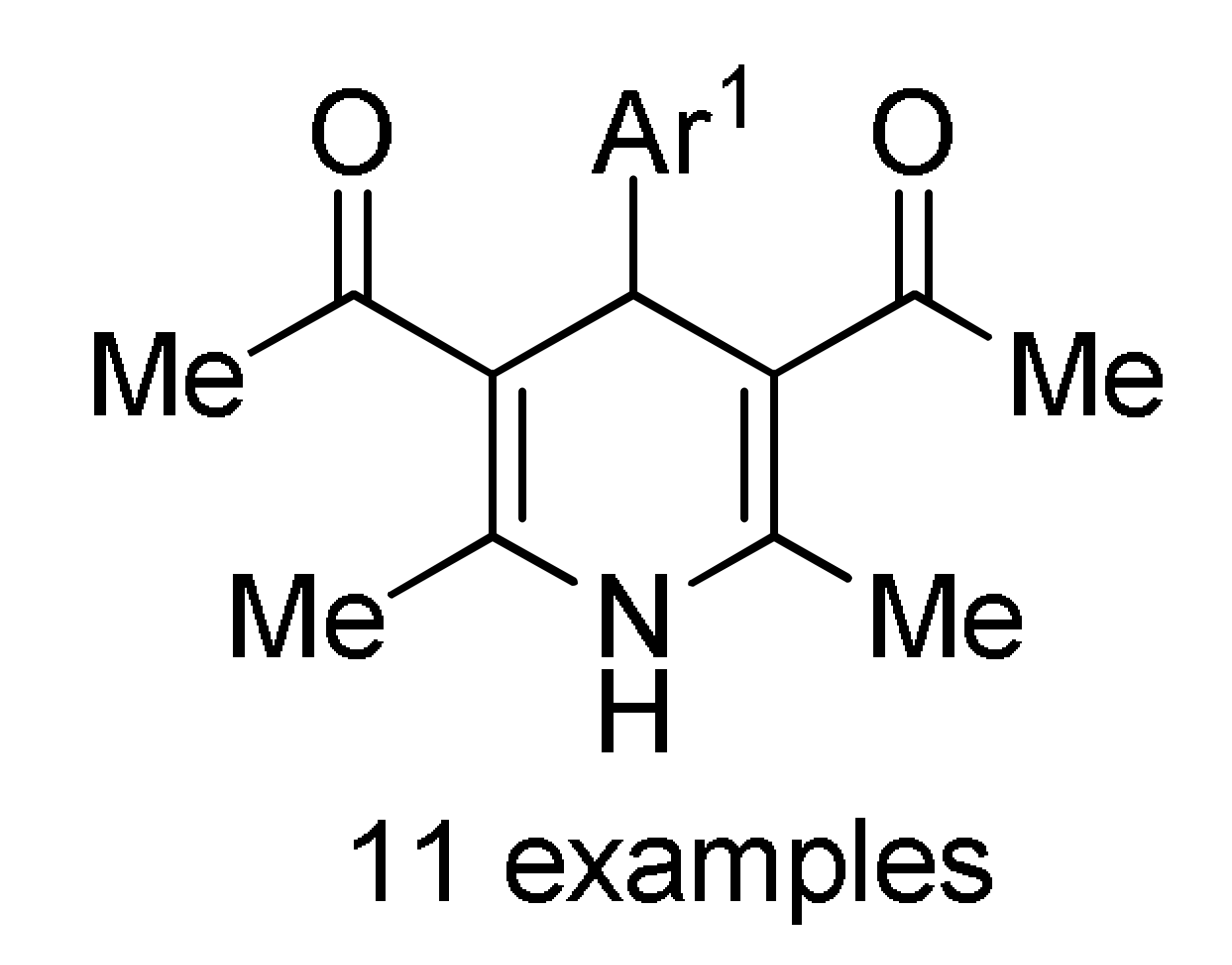 **160** ^b^	
	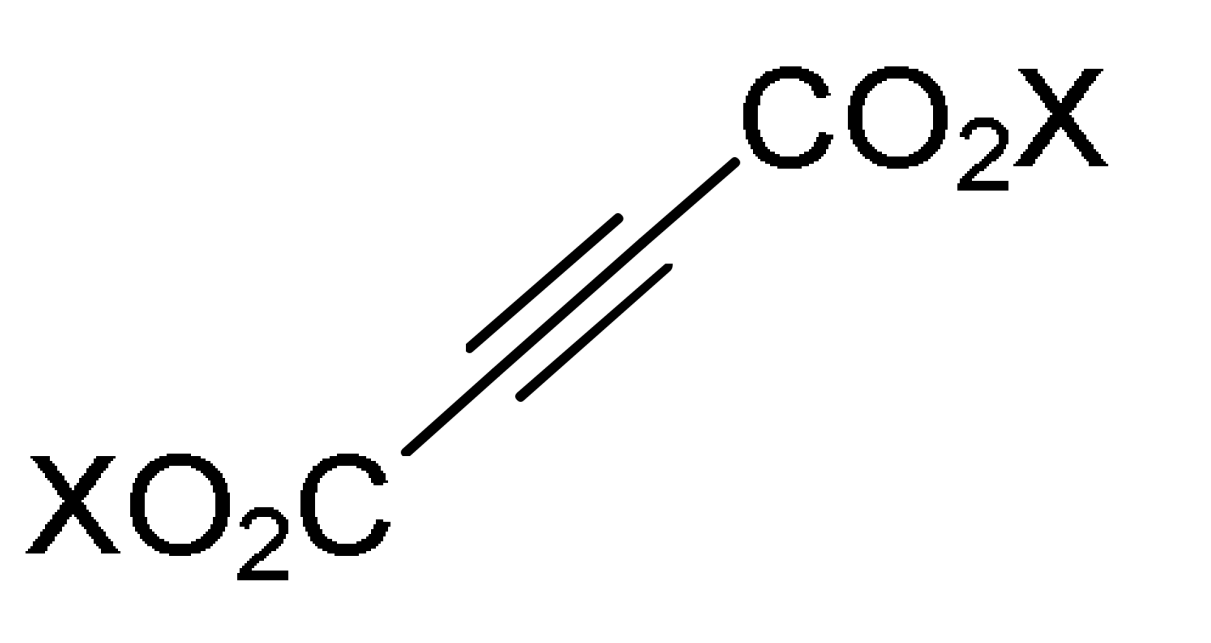 **47**	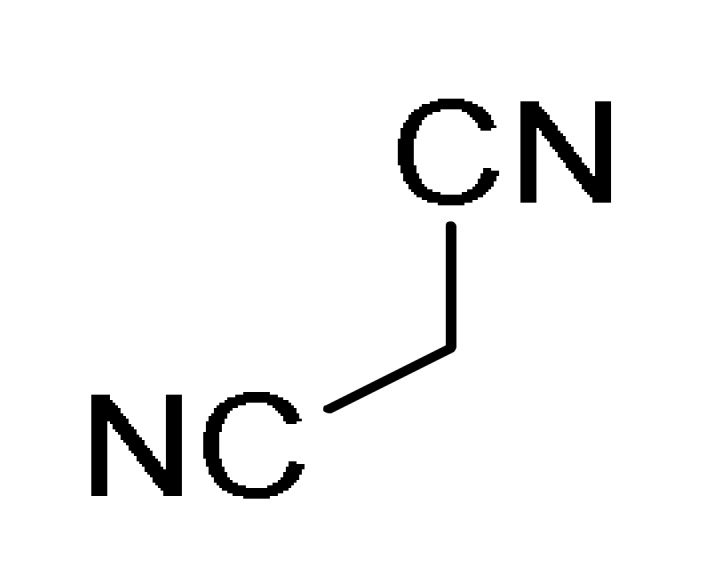 **12**	ArNH_2_ **2**	Montmorillonite-K10, H_2_O/EtOH (2:1), 60 °C	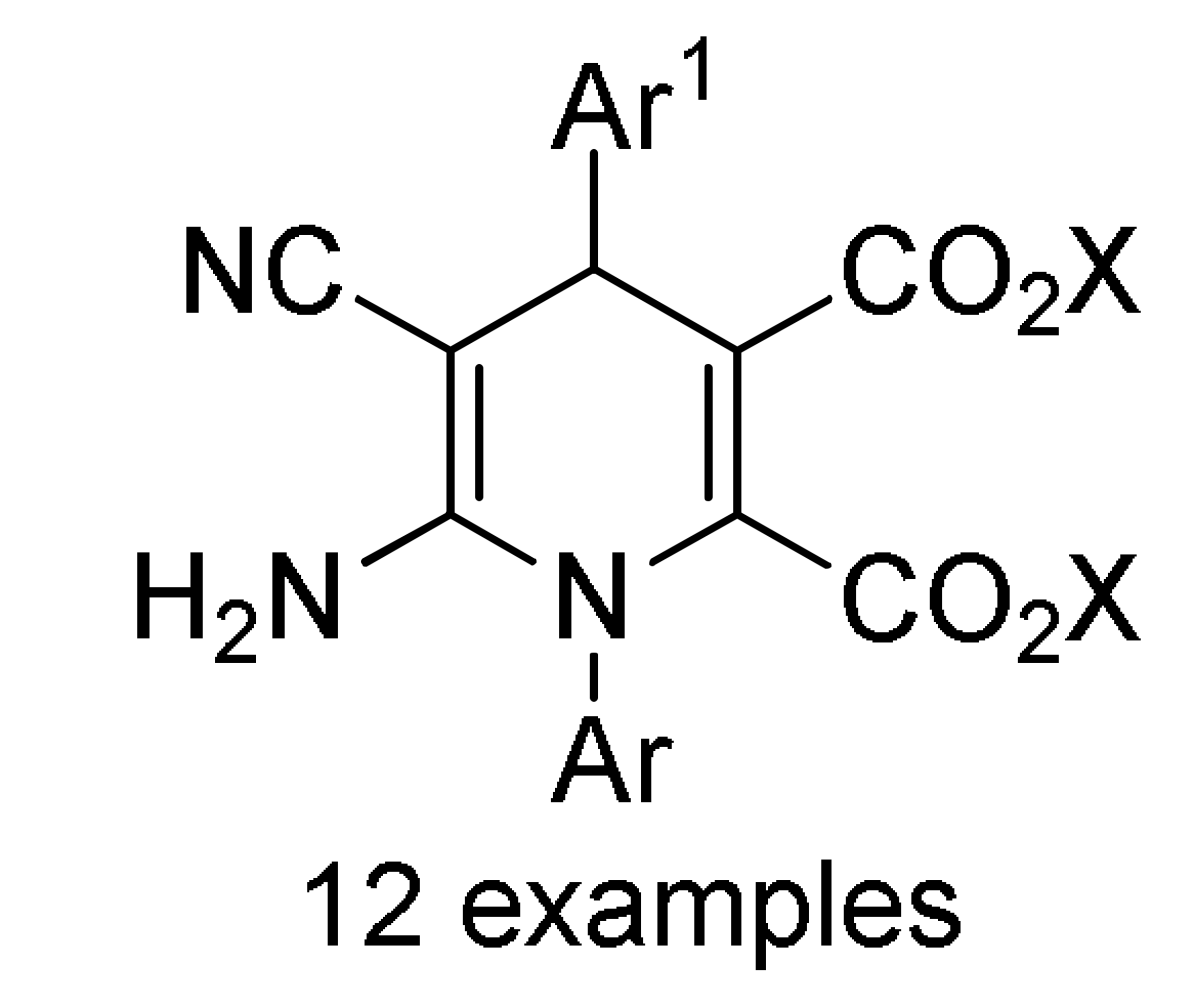 **161** ^c^	[178]

^a^ For compounds **157/158**: Ar^1^ = 2-HOC_6_H_4_, 2-ClC_6_H_4_, 3-ClC_6_H_4_, 2,5-(MeO)_2_C_6_H_3_, 2,4-(MeO)_2_C_6_H_3_, 3-thienyl, 4-EtOC_6_H_4_, 2-EtOC_6_H_4_, 4-MeOC_6_H_4_, 2-MeOC_6_H_4_, 4-ClC_6_H_4_; Ar = 4-BrC_6_H_4_, 3-BrC_6_H_4_, 2-BrC_6_H_4_, Ph, 3-thienyl. ^b^ For compounds **159/160**: Ar^1^ = 3-NO_2_C_6_H_4_, 3-PhOC_6_H_4_, 3-ClC_6_H_4_, 4-HO-3-MeOC_6_H_3_, 4-Me_2_NC_6_H_4_, 4-MeOC_6_H_4_, 4-ClC_6_H_4_, 4-MeSC_6_H_4_, 3-BrC_6_H_4_, 2-ClC_6_H_4_, Ph, 2-NO_2_C_6_H_4_, 3-MeC_6_H_4_, 2-MeC_6_H_4_; Ar = 4-MeSC_6_H_4_, 4-MeOC_6_H_4_, 3-NO_2_C_6_H_4_, 4-ClC_6_H_4_, 3-MeOC_6_H_4_, 4-HO-3-MeOC_6_H_3_. ^c^ For compounds **161**: X = Me, Et; Ar^1^ = Ph, 4-BrC_6_H_4_, 3-BrC_6_H_4_, 4-MeOC_6_H_4_, 4-ClC_6_H_4_, 3-MeC_6_H_4_, 2-HOC_6_H_4_; Ar = Ph, 4-MeOC_6_H_4_, 4-BrC_6_H_4_, 4-IC_6_H_4_, 4-FC_6_H_4_, 4-NO_2_C_6_H_4_.

**Table 3:** Multicomponent synthesis of fused dihydroquinolines **162**–**165** for evaluation of their potential anticancer properties.

Reagents			Conditions	Product	Reference
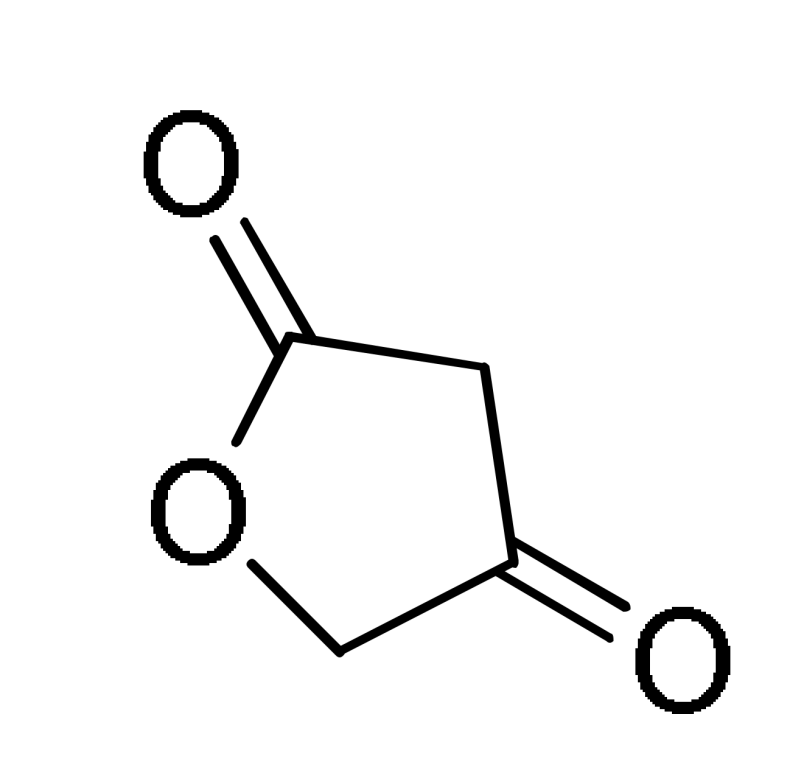 **27**	ArCHO **1**	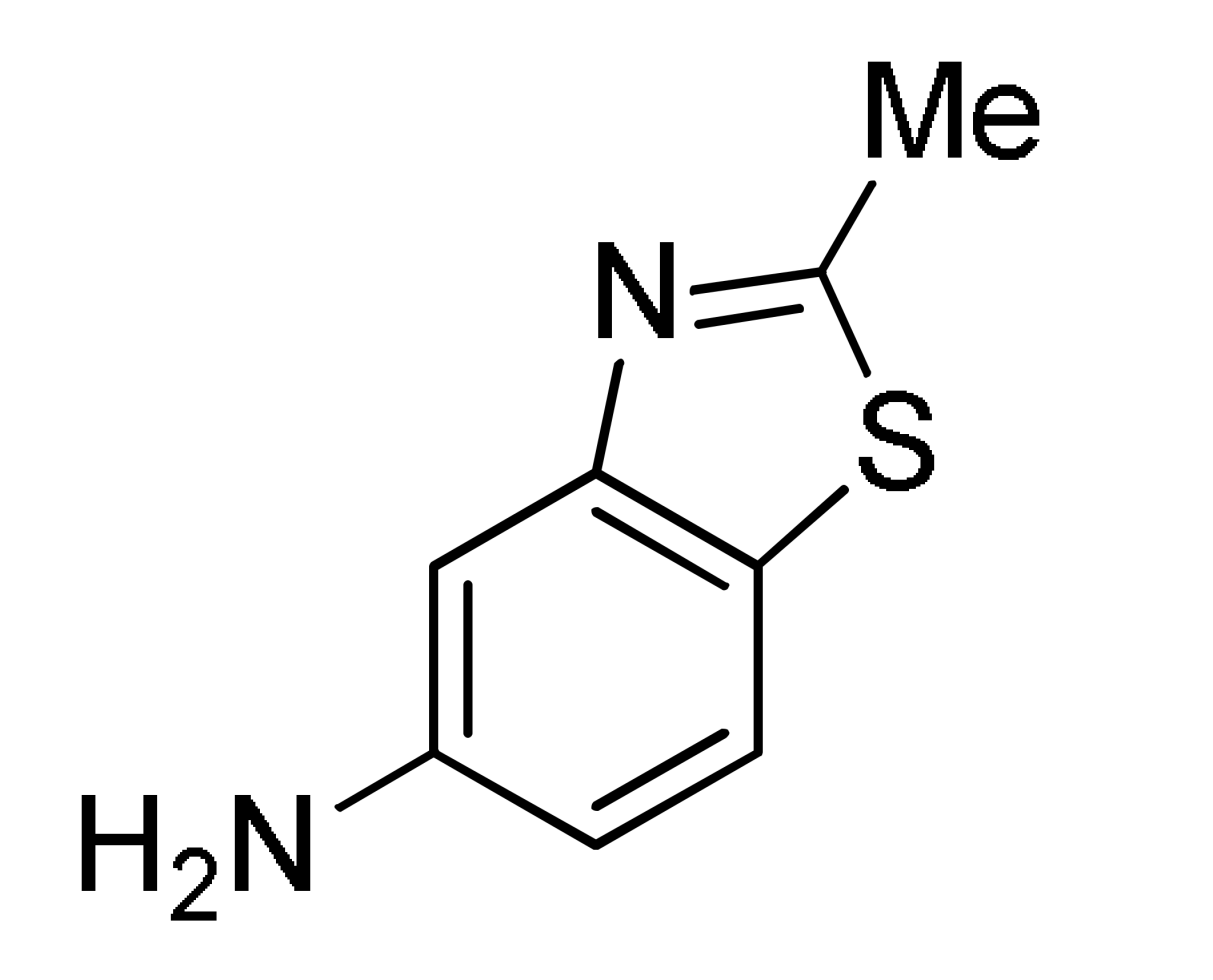 **2**	AcOH, MWI, 120 °C	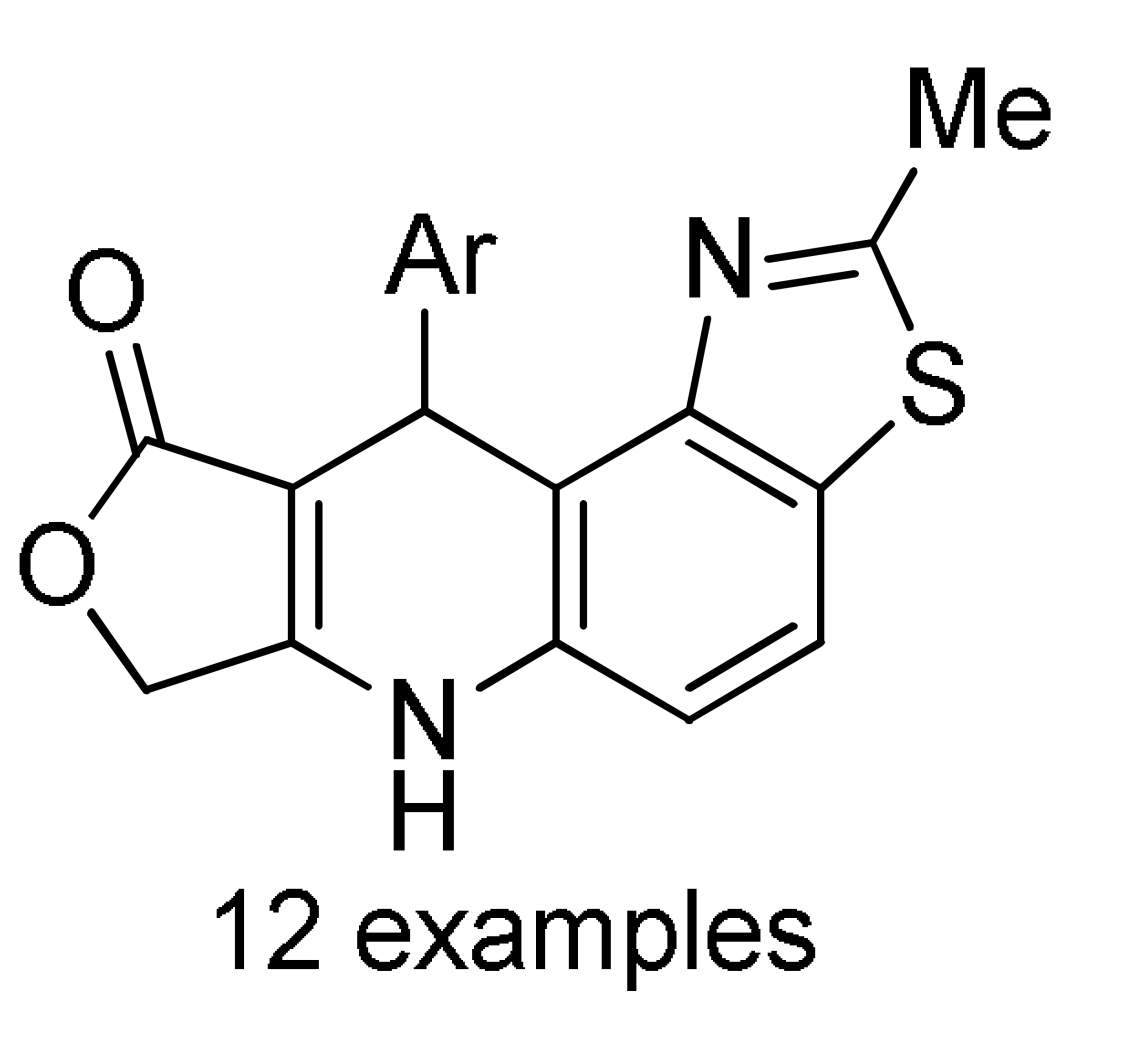 **162** ^a^	[145]
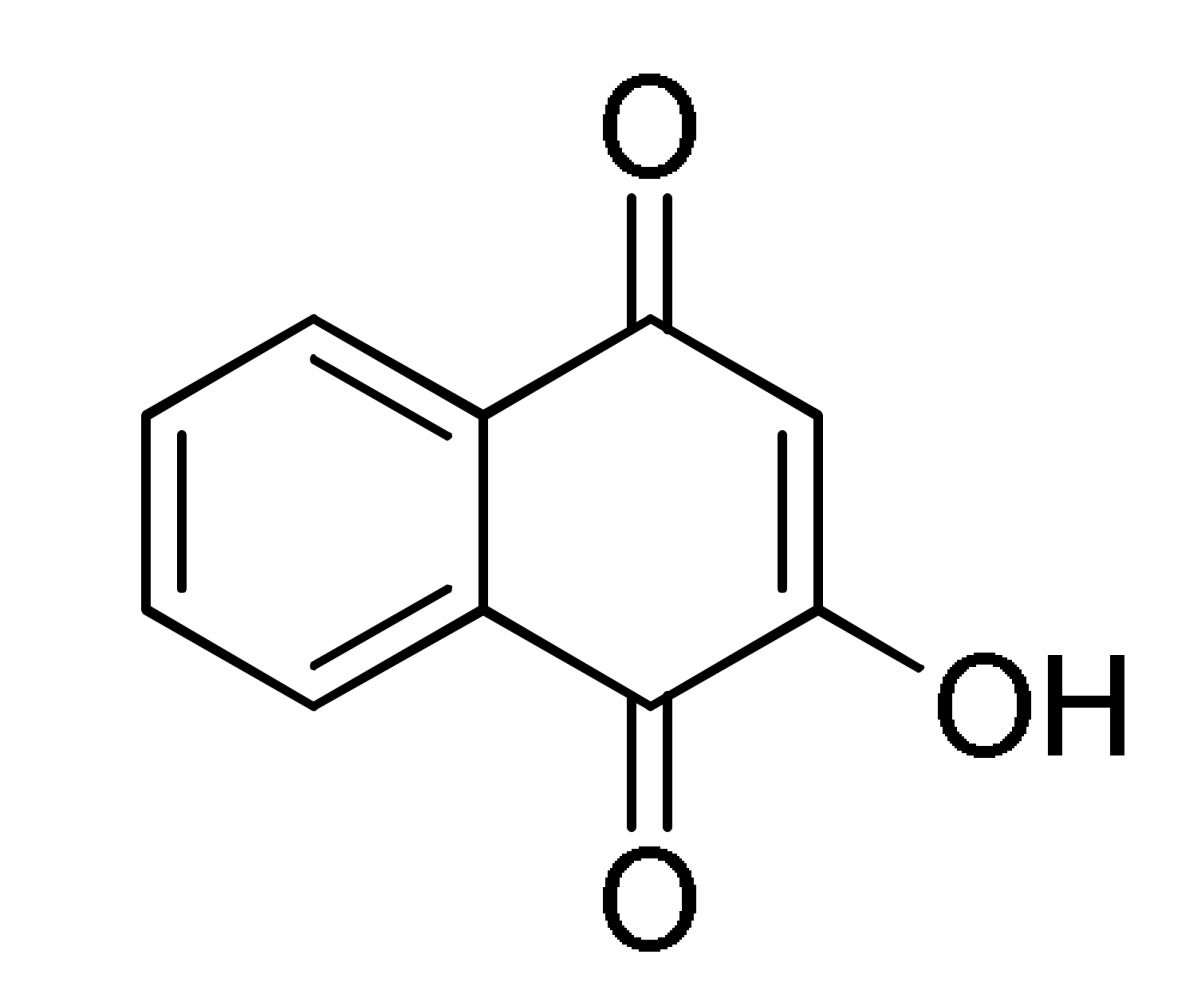 **94**	RCHO **1**	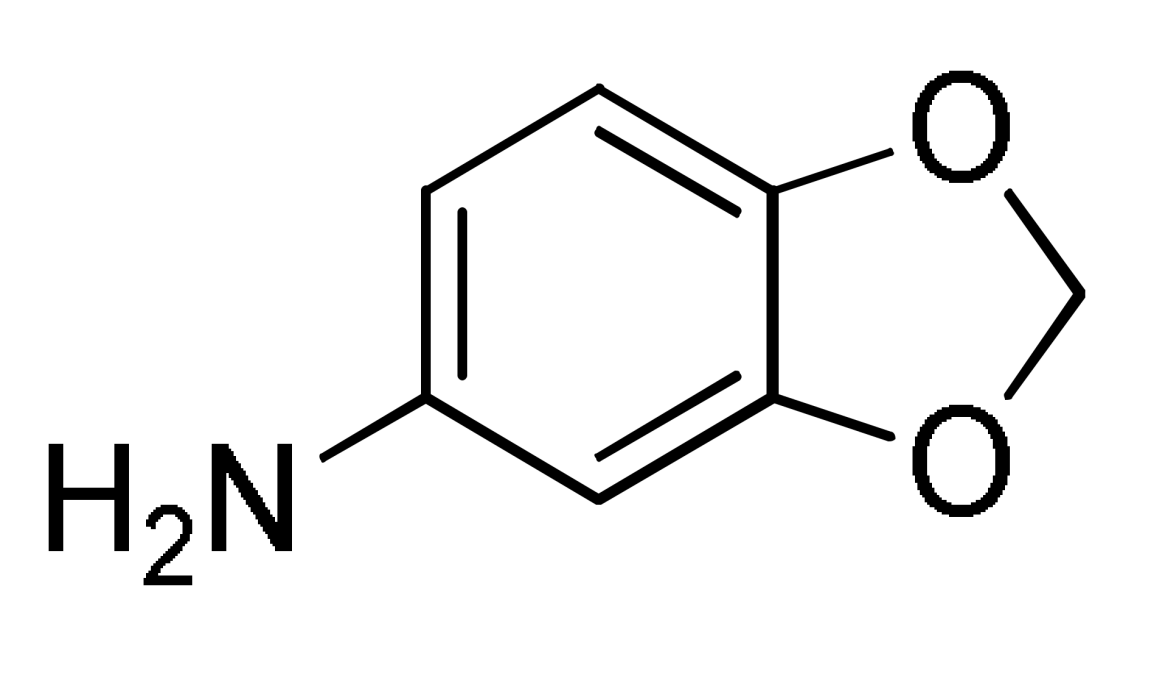 **2**	L-Proline, EtOH, reflux	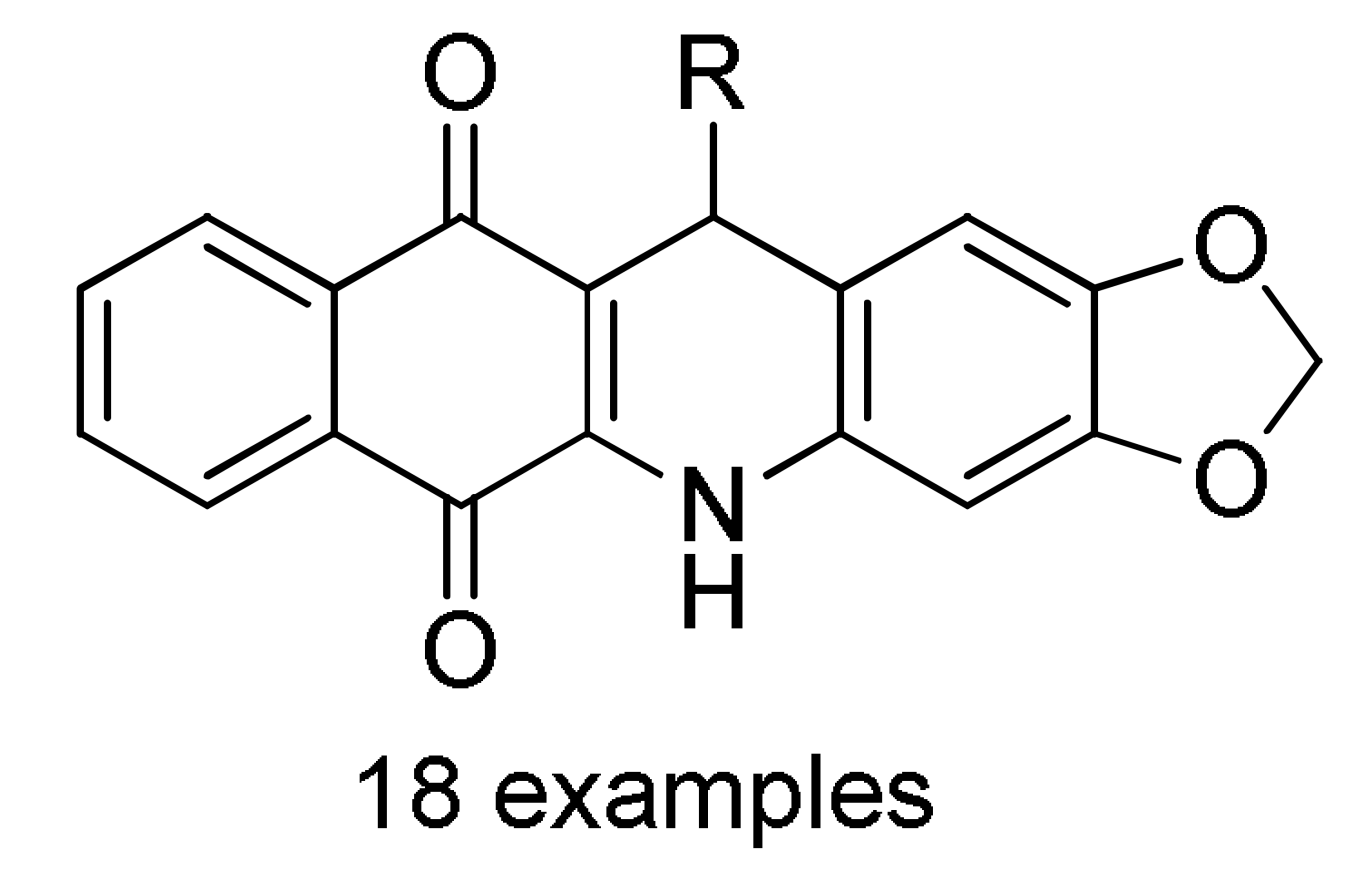 **163** ^b^	[181]
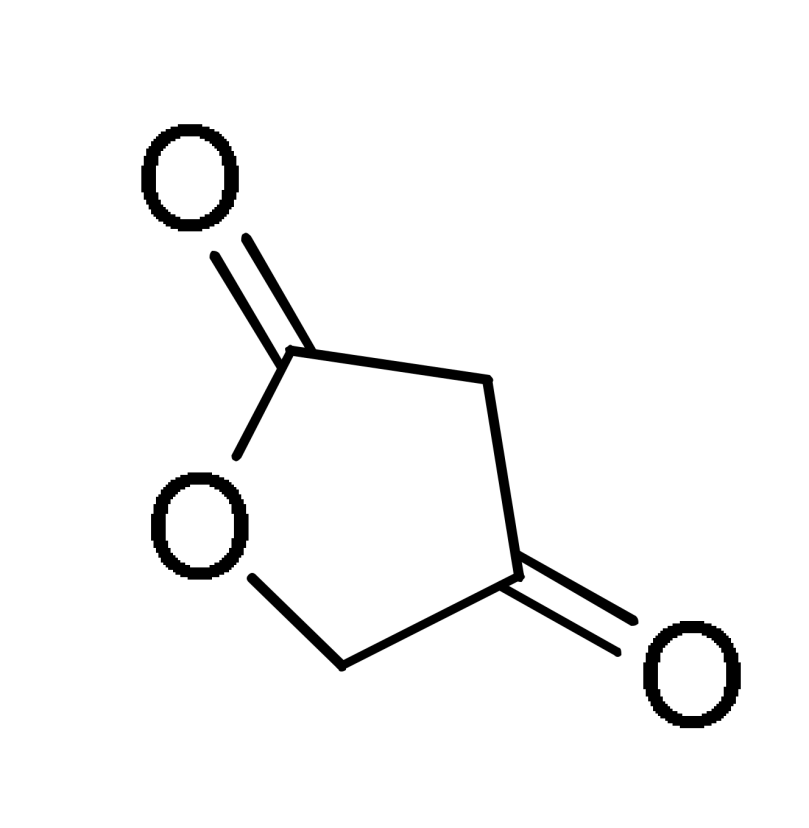 **27**	ArCHO **1**	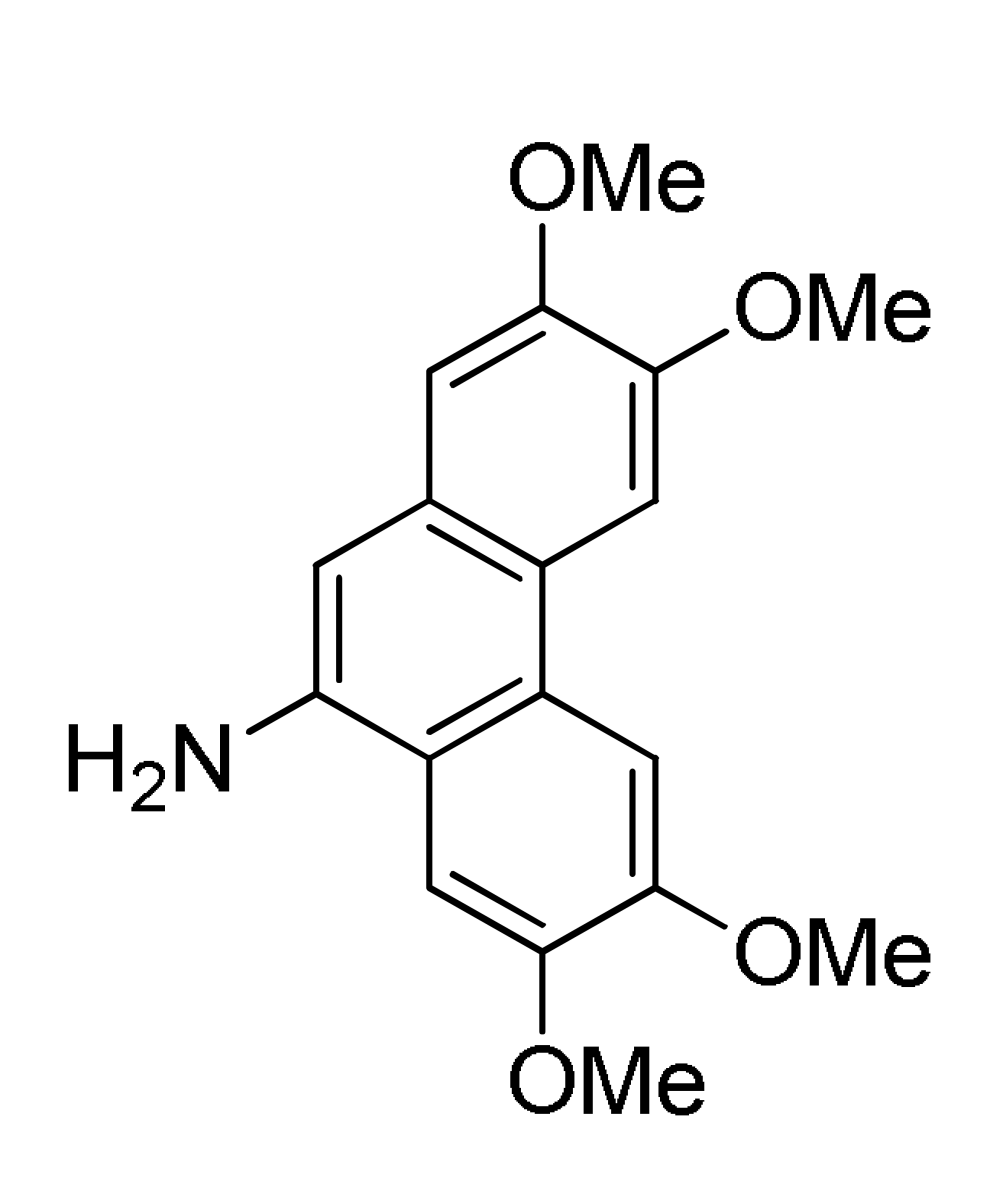 **2**	EtOH, reflux	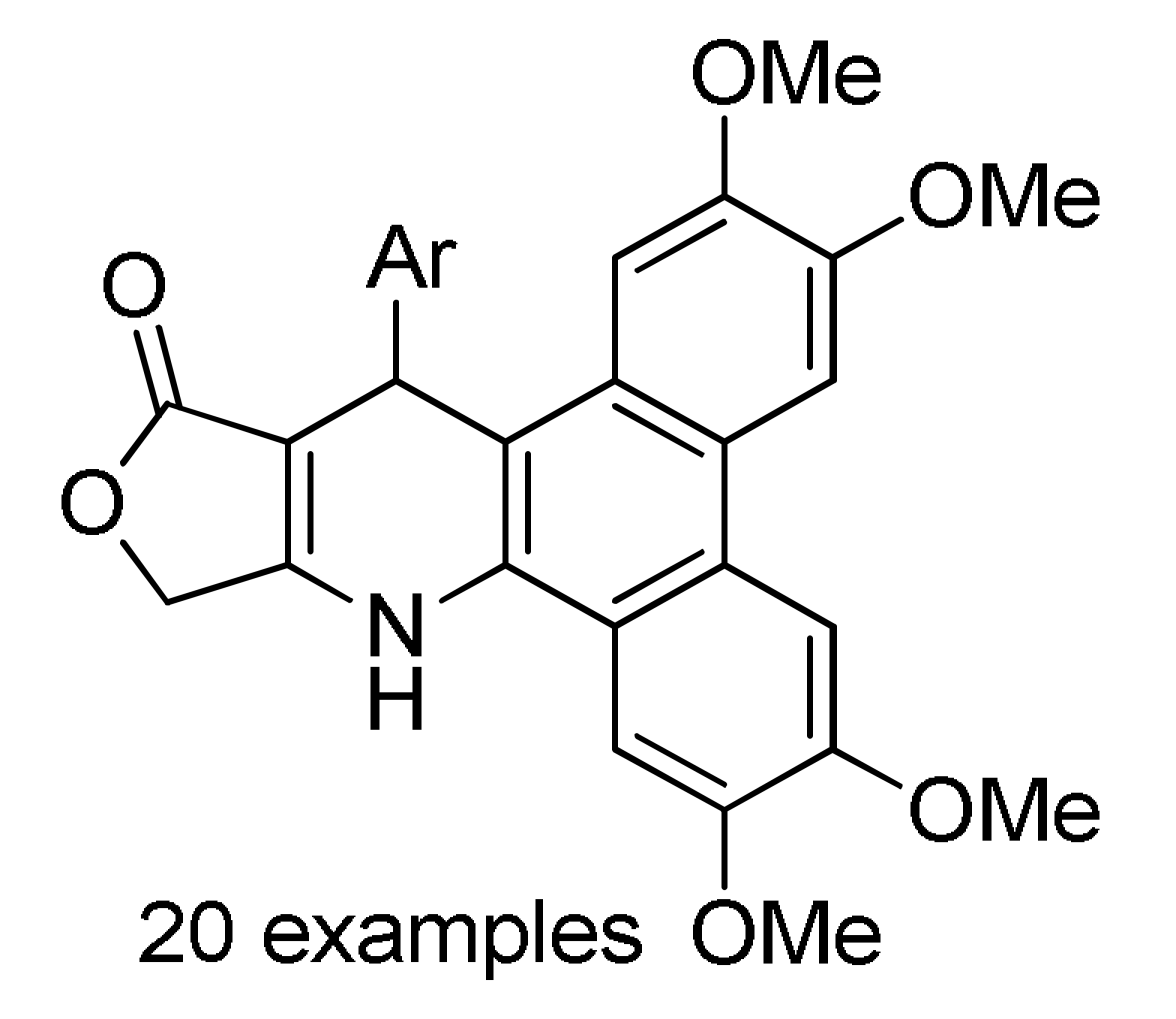 **164** ^c^	[182]
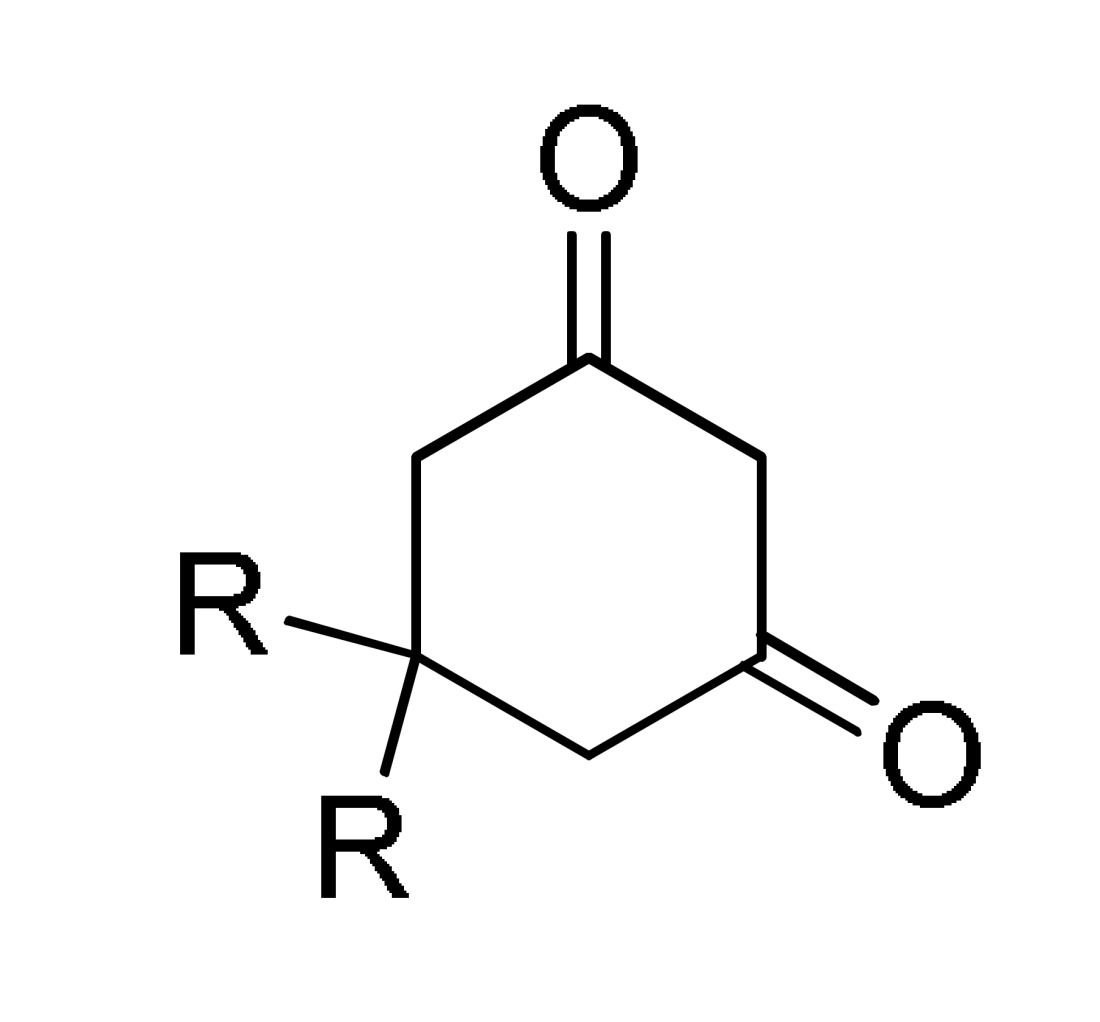 **10a**			EtOH, MWI, 150 °C	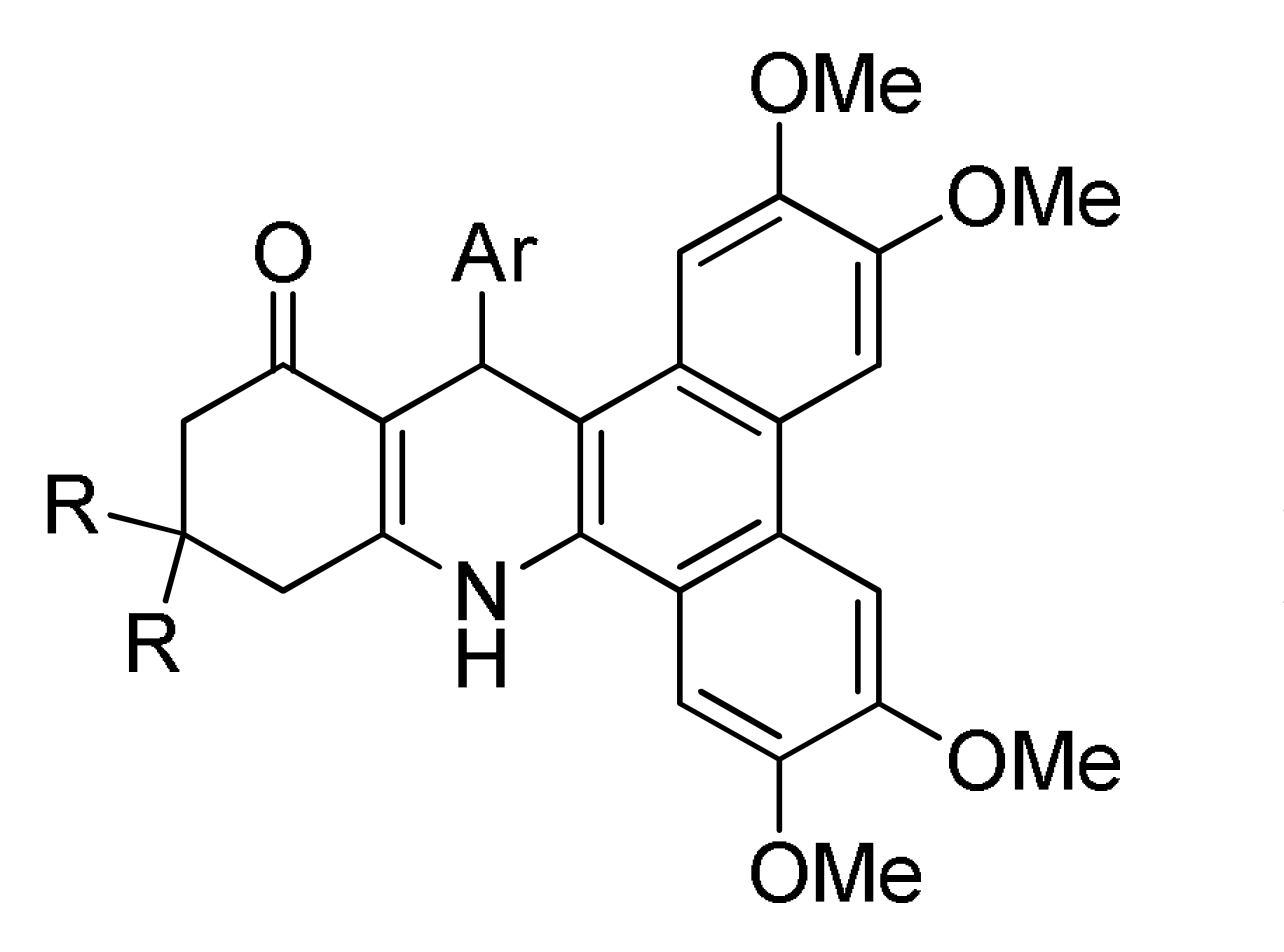 **165** ^d^	[183]

^a^ For compounds **162**: Ar = 4-FC_6_H_4_, 4-ClC_6_H_4_, 4-BrC_6_H_4_, 3-NO_2_C_6_H_4_, 4-NO_2_C_6_H_4_, Ph, 4-MeC_6_H_4_, 4-MeOC_6_H_4_, 2,4-(Cl)_2_C_6_H_3_, 4-HO-3-NO_2_C_6_H_3_, 3,4,5-(MeO)_3_C_6_H_2_, 2-thienyl. ^b^ For compounds **163**: R = Ph, 4-MeC_6_H_4_, 4-MeOC_6_H_4_, 3-MeOC_6_H_4_, 4-ClC_6_H_4_, 2-ClC_6_H_4_, 4-FC_6_H_4_, 2-FC_6_H_4_, 3-NO_2_C_6_H_4_, 4-NO_2_C_6_H_4_, 3,4-(Cl)_2_C_6_H_3_, 2,4-(Cl)_2_C_6_H_3_, 2,5-(MeO)_2_C_6_H_3_, 3,5-(MeO)_2_C_6_H_3_, 3,4,5-(MeO)_3_C_6_H_2_, 2-furyl, 2-thienyl, Me. ^c^ For compounds **164**: Ar = 3,4-(MeO)_2_C_6_H_3_, 3,4,5-(MeO)_3_C_6_H_2_, 2,4-(F)_2_C_6_H_3_, 3,4-(HO)_2_C_6_H_3_, 4-ClC_6_H_4_, 4-HO-3,5-(MeO)_2_C_6_H_2_, 4-CNC_6_H_4_, 3-BrC_6_H_4_, 4-FC_6_H_4_, 4-MeOC_6_H_4_, 3-CF_3_C_6_H_4_, 4-*i*PrC_6_H_4_, 4-MeC_6_H_4_, Ph, 2-naphthyl, 3-MeOC_6_H_4_, 2-thienyl, 3-HOC_6_H_4_, 2-FC_6_H_4_, 4-BrC_6_H_4_. ^d^ For compounds **165**: R = H, Me; Ar = 4-ClC_6_H_4_, 3,4-(MeO)_2_C_6_H_3_, 4-CF_3_C_6_H_4_, 4-*i*PrC_6_H_4_, 3,4,5-(MeO)_3_C_6_H_2_, 4-BrC_6_H_4_, 4-HO-3-MeOC_6_H_3_, 4-HO-3,5-(MeO)_2_C_6_H_2_, 3,4-(OCH_2_O)C_6_H_3_, 4-MeC_6_H_4_, 4-MeOC_6_H_4_, 3-CF_3_C_6_H_4_, 1-naphthyl, 2-thienyl, 5-Me-2-thienyl.

**Table 4:** Multicomponent approaches for the synthesis of 7-deazaadenine (**166**) 7-deazapurine (**167**) and 7-deazahypoxanthine skeletons **168** and **169** of potential antitumor activity.

Reagents				Conditions	Product	Reference
ArCHO **1**	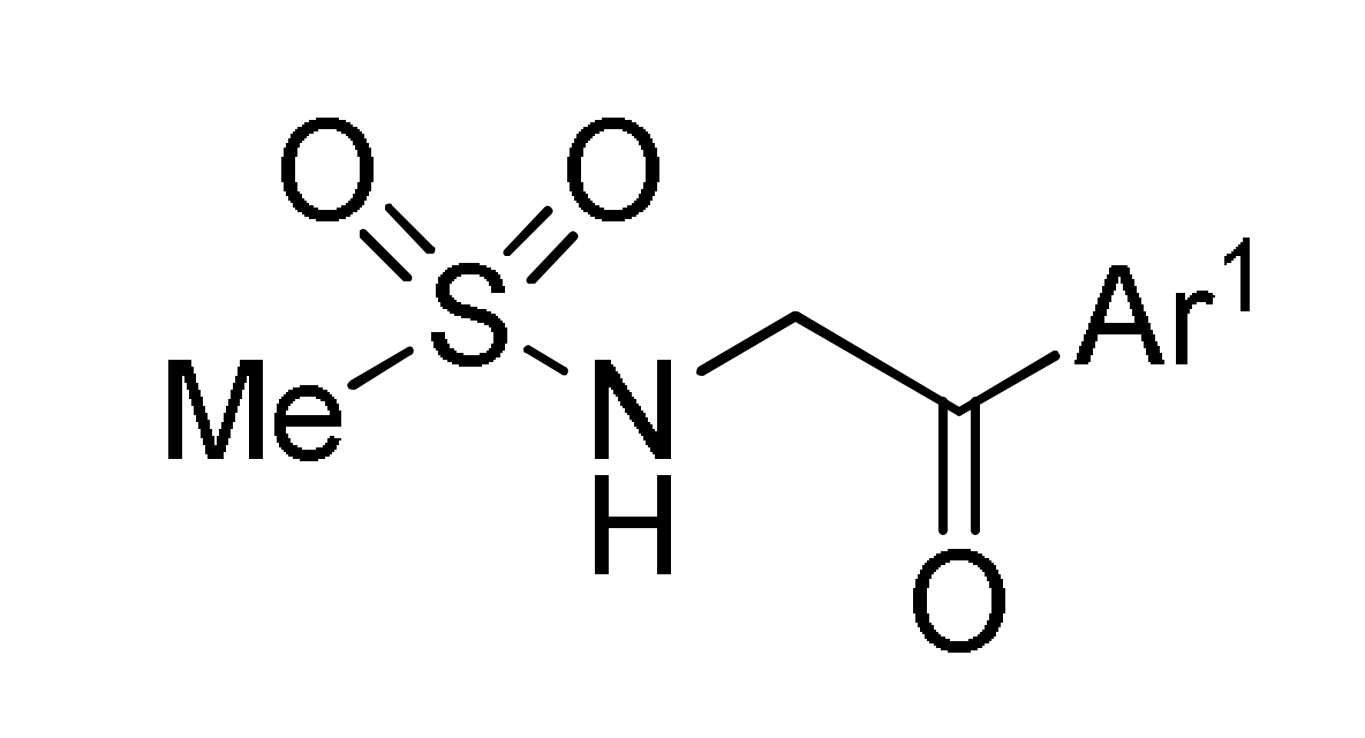 **119**	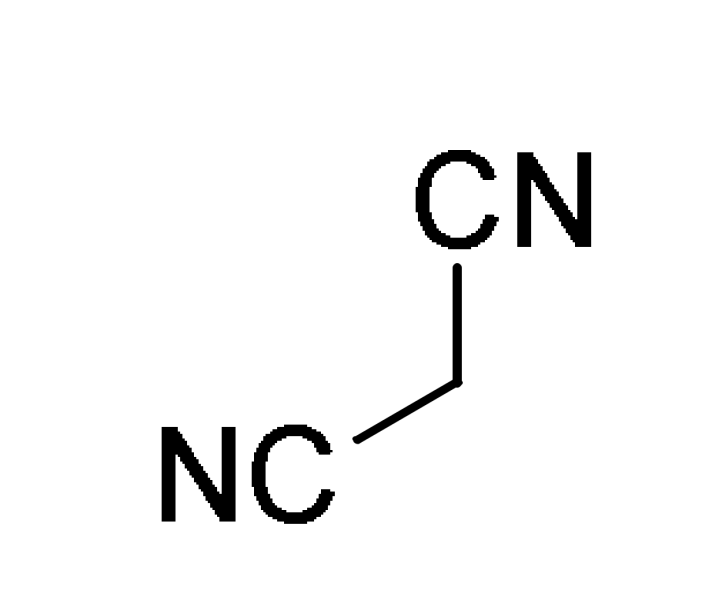 **12**	HCONH_2_	K_2_CO_3_, 90 °C → 150 °C	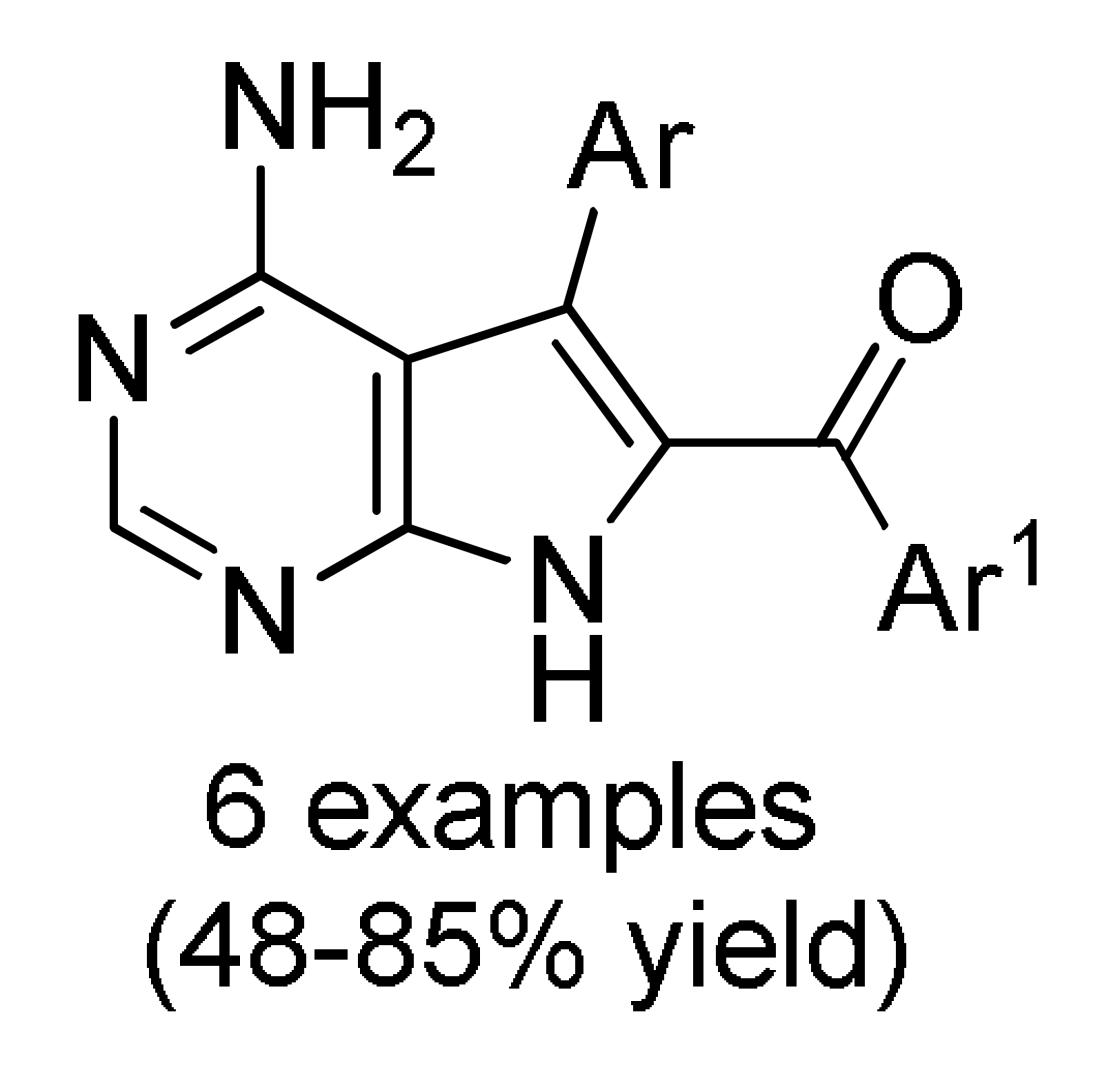 **166** ^a^	[187]
		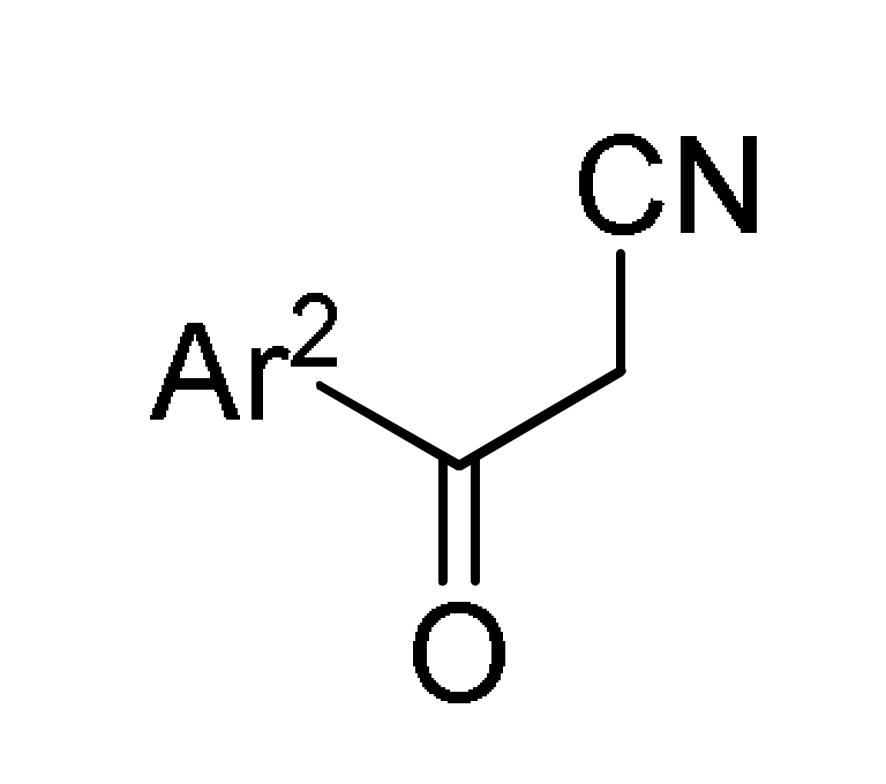 **38**			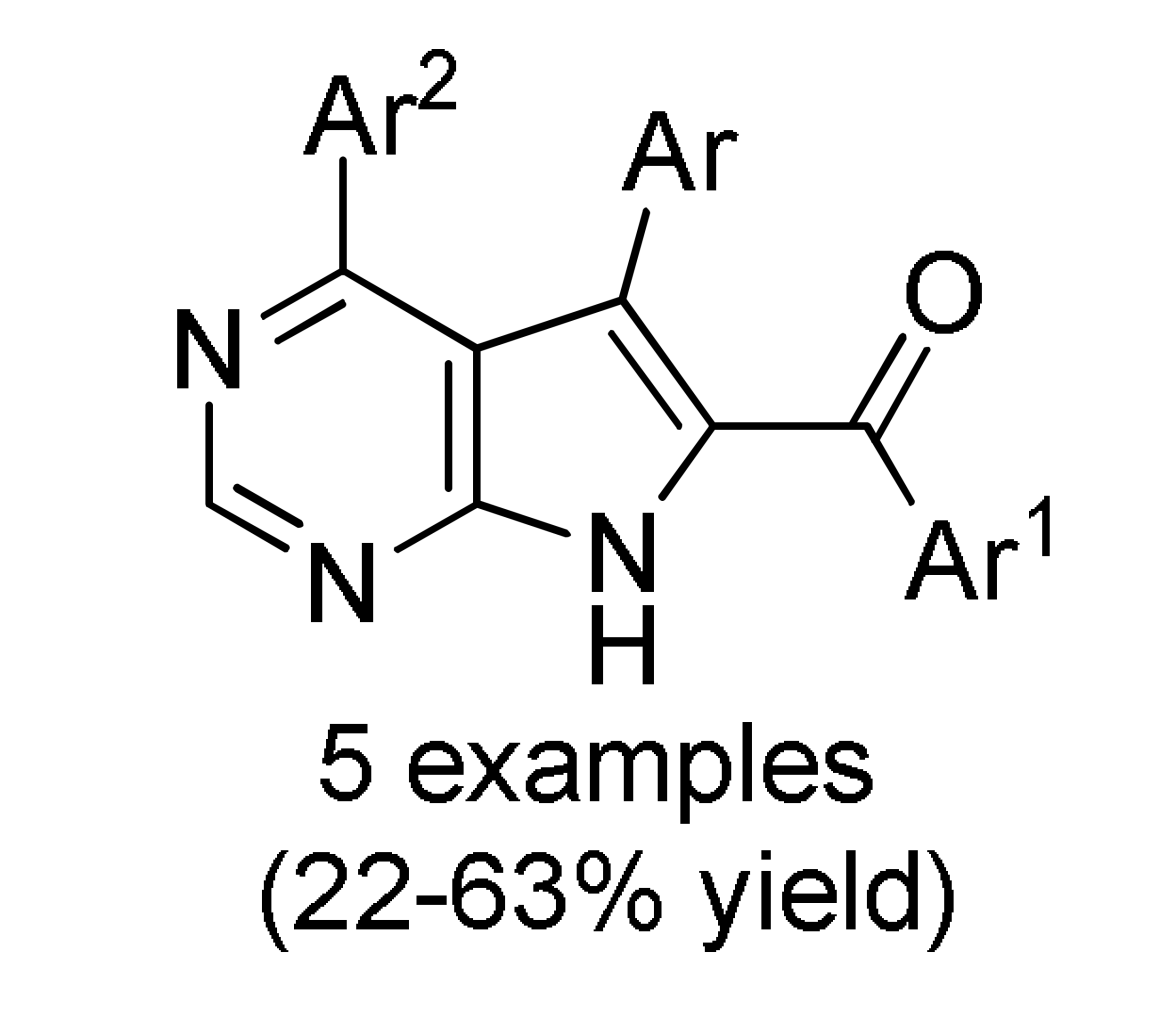 **167** ^b^	
		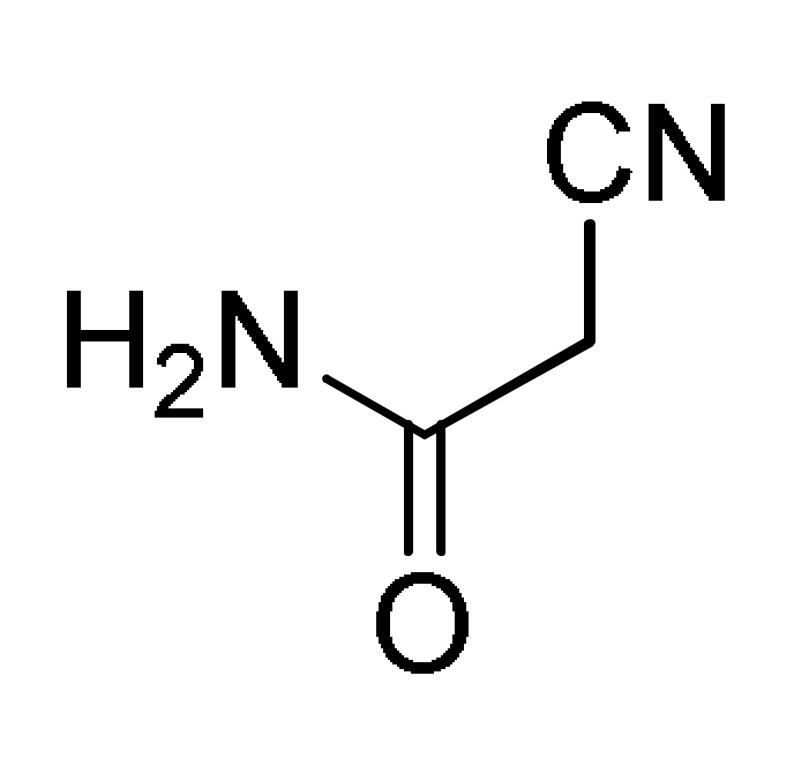 **38**	HC(OEt)_3_	EtOH, K_2_CO_3_, 90 °C → 150 °C	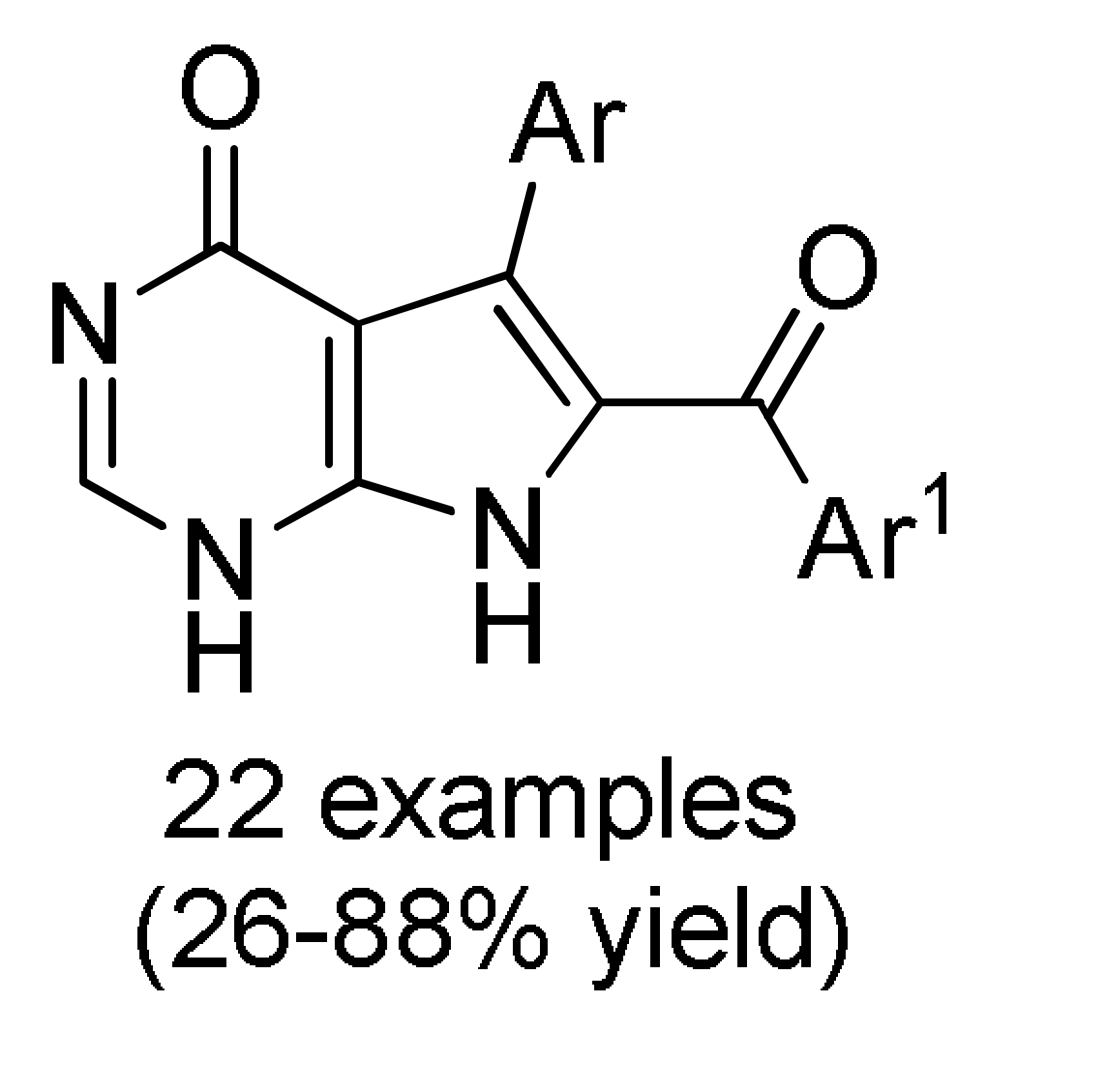 **168** ^c^	
	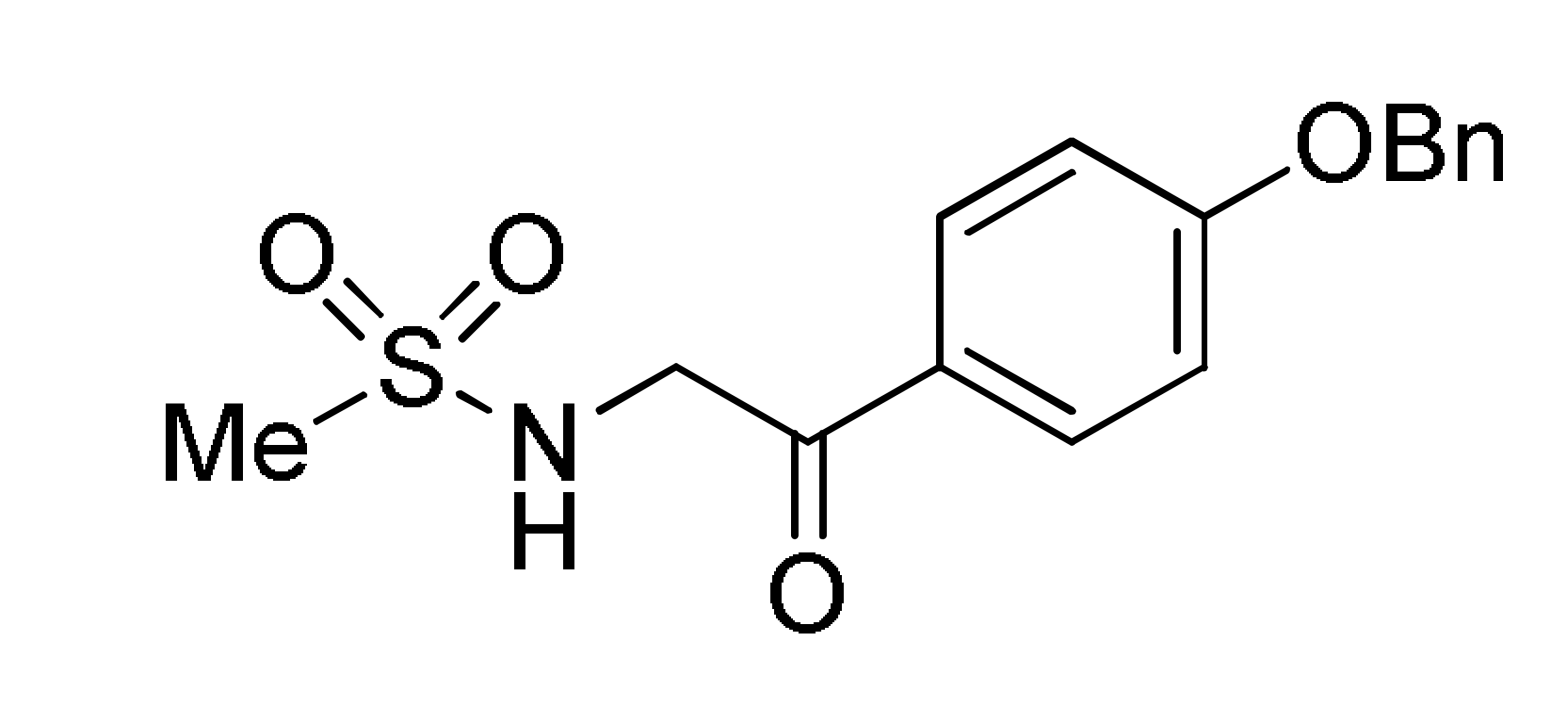 **119**		HCO_2_H	EtOH, K_2_CO_3_, 90 °C → 100 °C	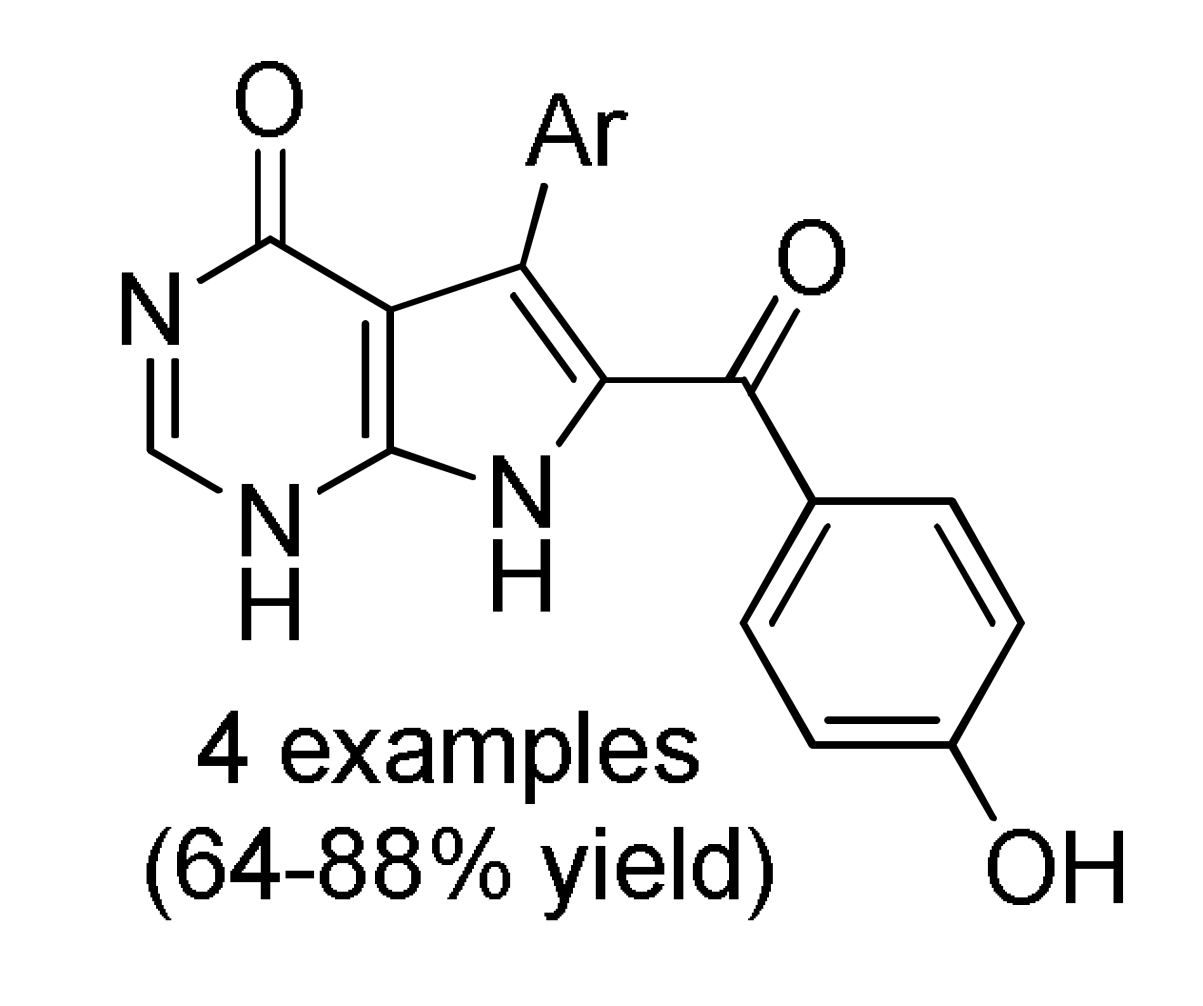 **169** ^d^	

^a^ For compounds **166**: Ar = 3,5-(Br)_2_C_6_H_3_, 5-bromo-3-Py, Ph, 2-thienyl, 3-CNC_6_H_4_, 2,6-(Cl)_2_C_6_H_3_; Ar^1^ = Ph, 4-BnOC_6_H_4_. ^b^ For compounds **167**: Ar = 4-MeOC_6_H_4_, 3,5-(Br)_2_C_6_H_3_, 3-Py; Ar^1^ = Ph, 4-MeOC_6_H_4_; Ar^2^ = Ph, 4-MeOC_6_H_4_, 4-MeC_6_H_4_. ^c^ For compounds **168**: Ar = Ph, 3-IC_6_H_4_, 3-BrC_6_H_4_, 3-ClC_6_H_4_, 2,6-(Cl)_2_C_6_H_3_, 3,5-(Br)_2_C_6_H_3_, 3-HOC_6_H_4_, 3-CNC_6_H_4_, 3-FC_6_H_4_, 6-Br-2-Py, 5-Br-3-Py, 3-Py, 3,4,5-(MeO)_3_C_6_H_2_, 3,4-(MeO)_2_-5-IC_6_H_2_, 5-Br-3,4-(MeO)_2_C_6_H_2_, 4-Br-2-thienyl; Ar^1^ = Ph, 4-MeOC_6_H_4_, 4-FC_6_H_4_, 4-BnOC_6_H_4_, 4-BrC_6_H_4_. ^d^ For compounds **169**: Ar = Ph, 3,5-(Br)_2_C_6_H_3_, 4-HOC_6_H_4_, 4-HO-3-MeOC_6_H_3_.

**Table 5:** Multicomponent synthesis of cyclopentanone-based dihydropyran/dihydropyridine heterocyclic systems **234**–**238** of potential anticancer activity.

Reagents				Conditions	Product	Reference
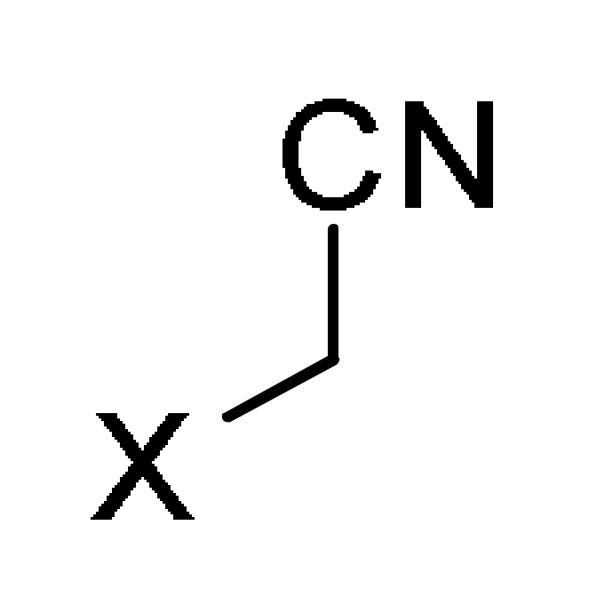 **12/38** X = CN, CO_2_Et, CONH_2_	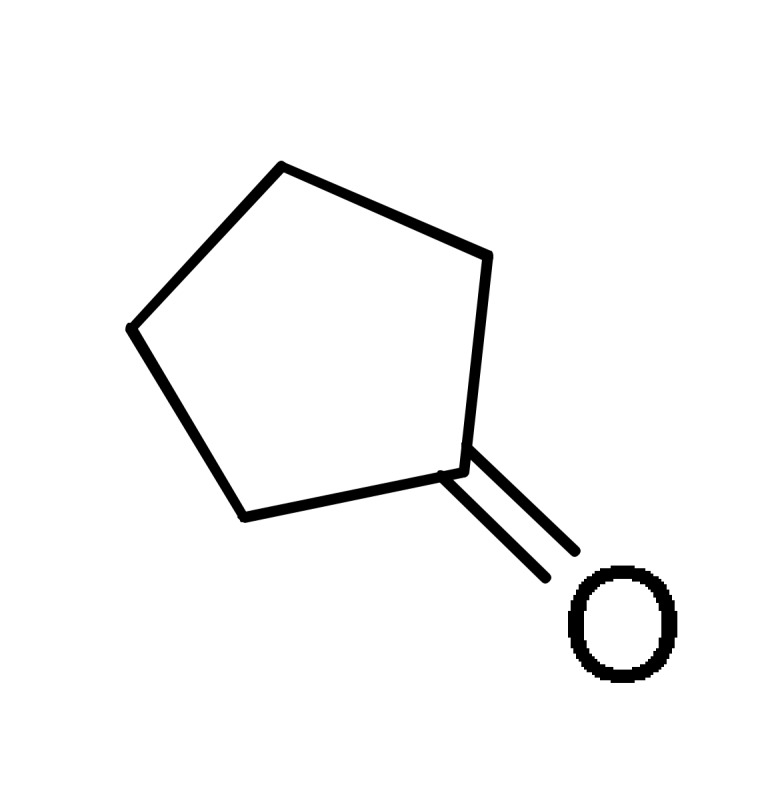 **16**	ArCHO **1**	TEA	EtOH, reflux	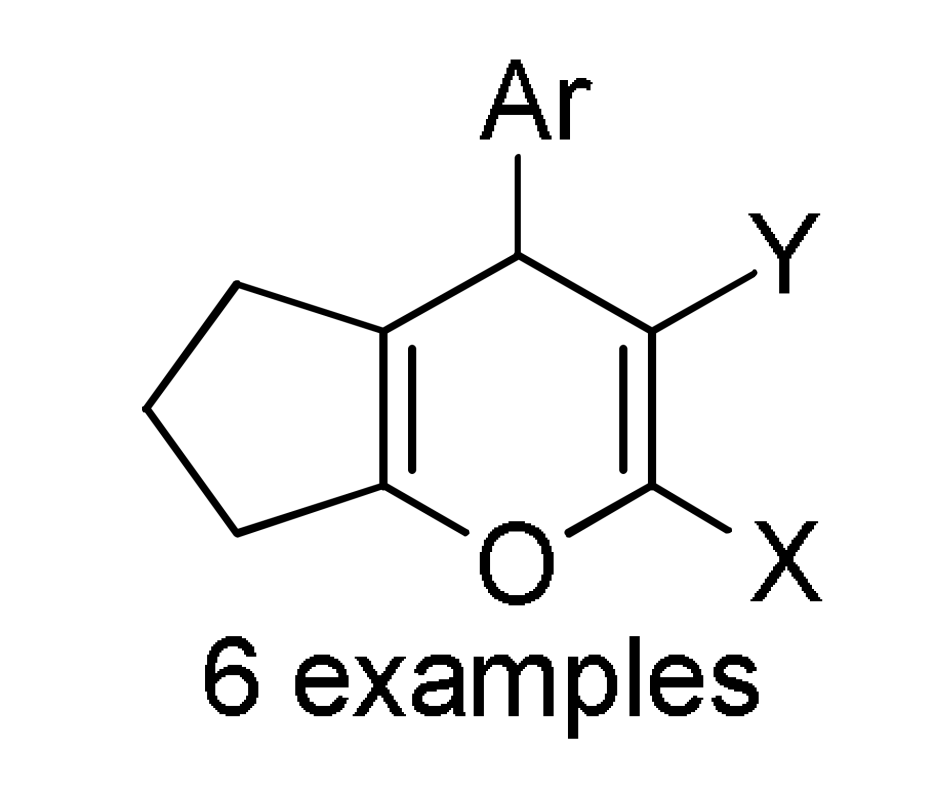 **234** ^a^	[231]
		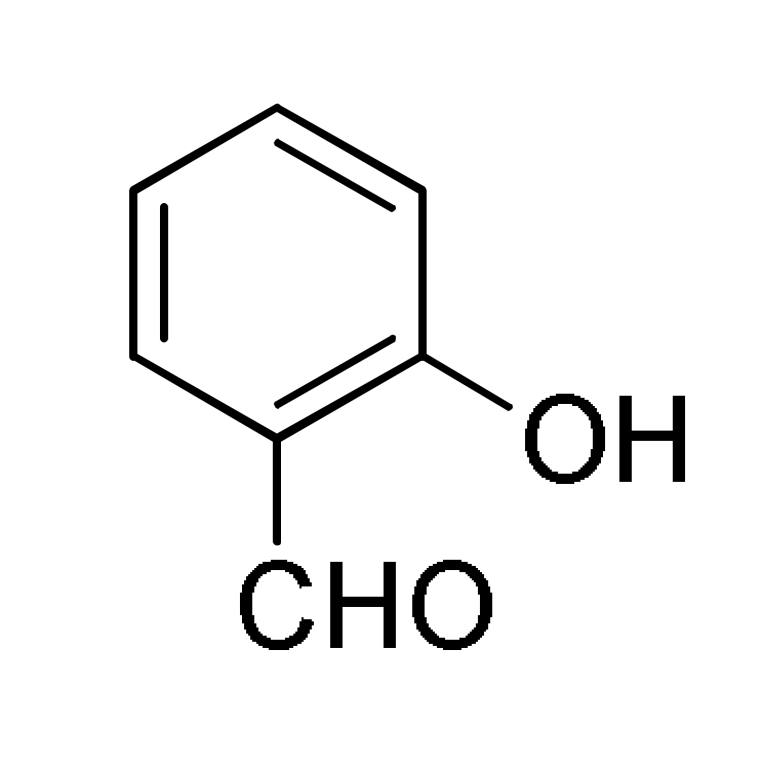 **1**			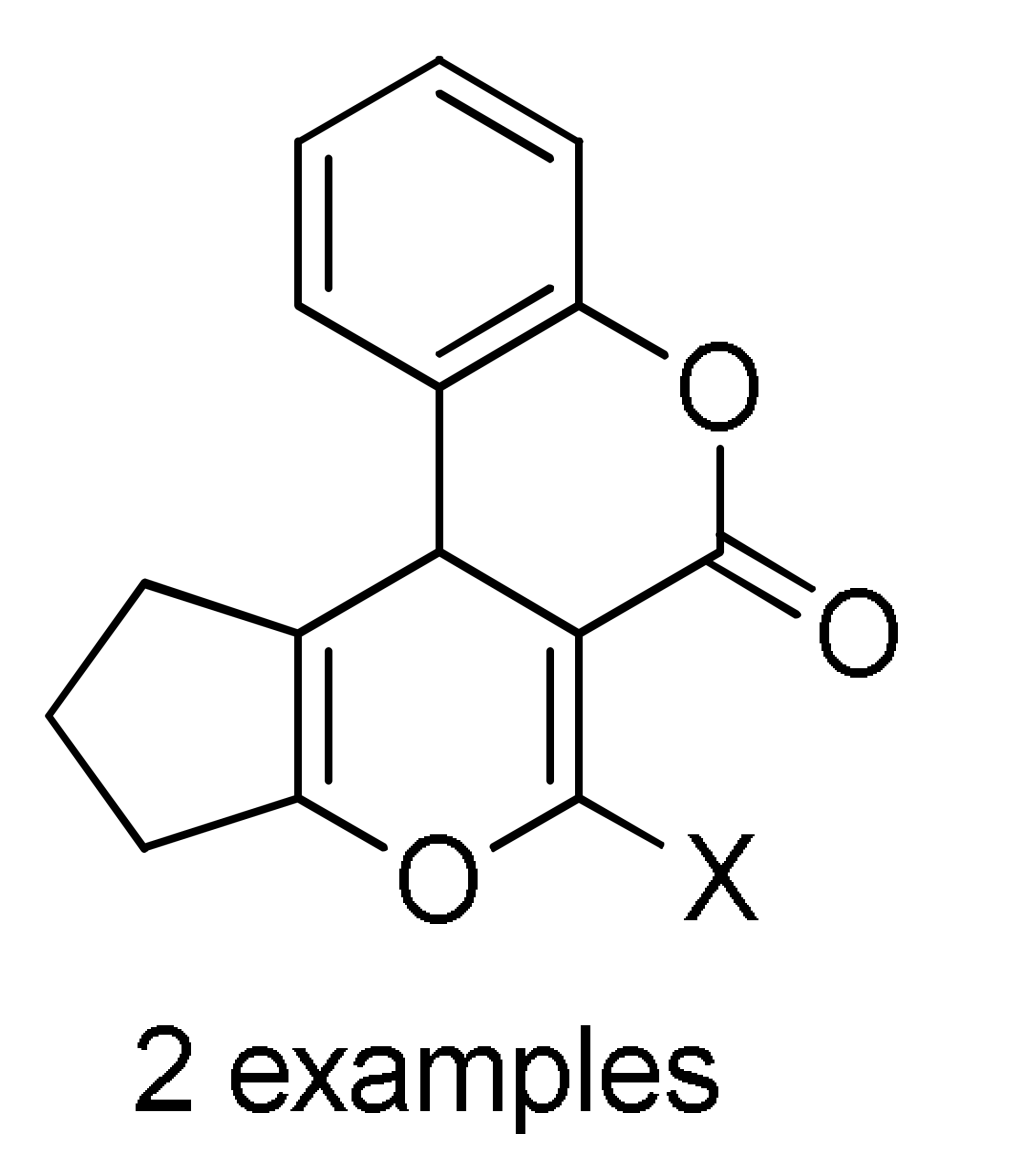 **235** ^b^	
		ArCHO **1**	AcONH_4_		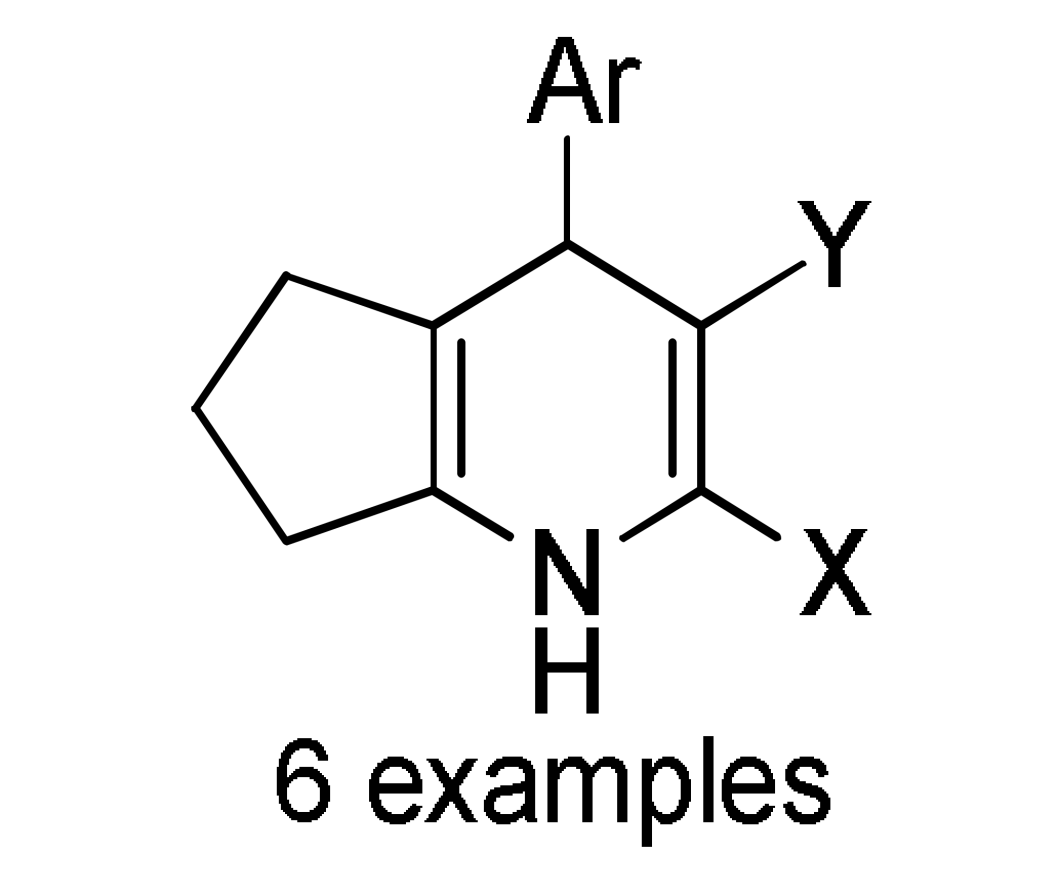 **236** ^a^	
		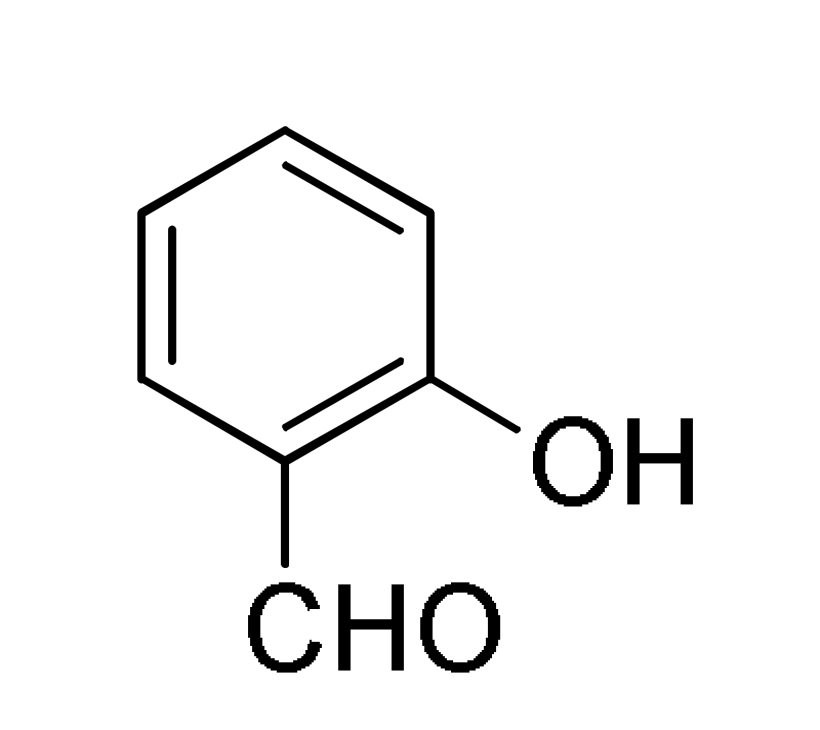 **1**			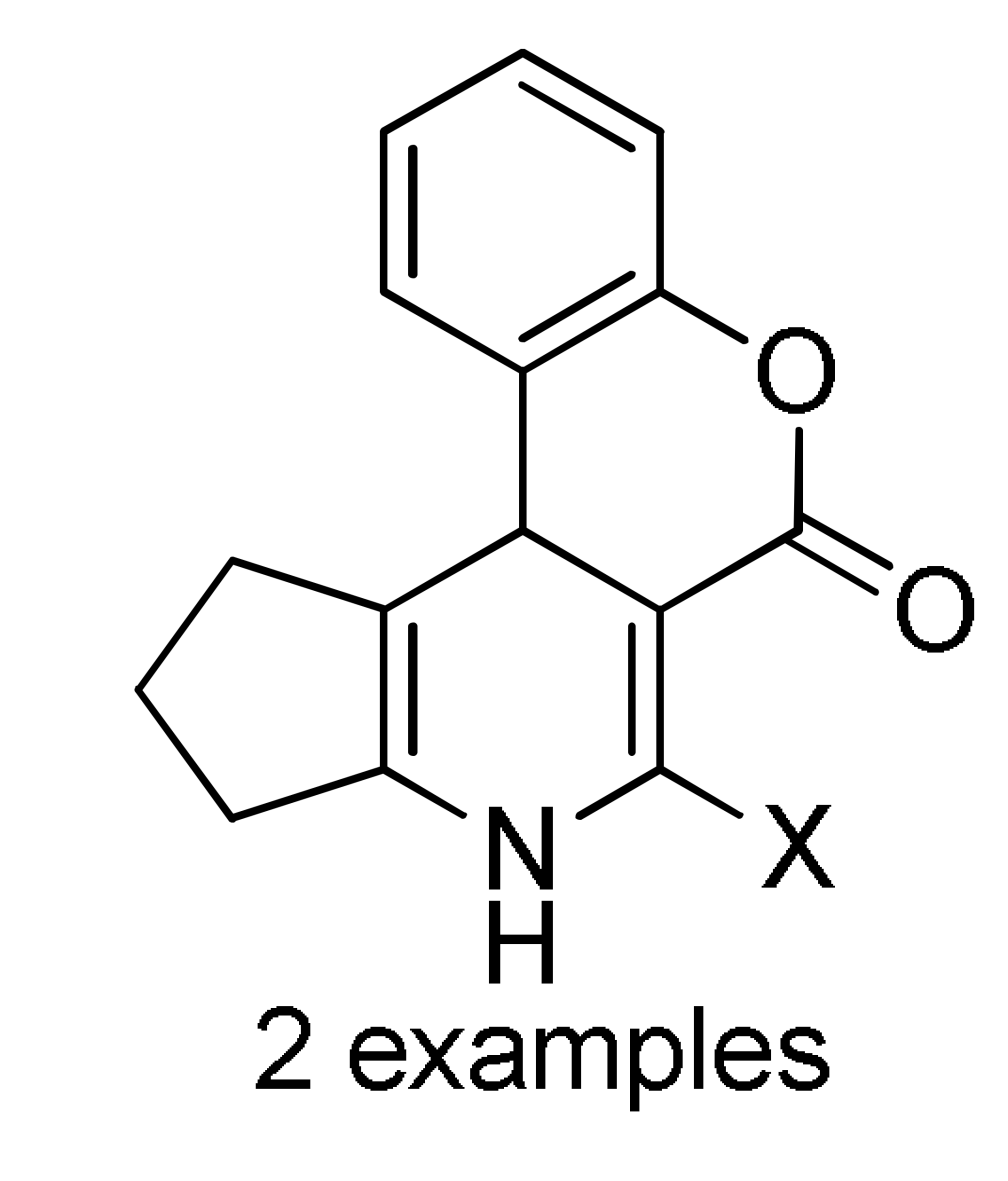 **237** ^b^	
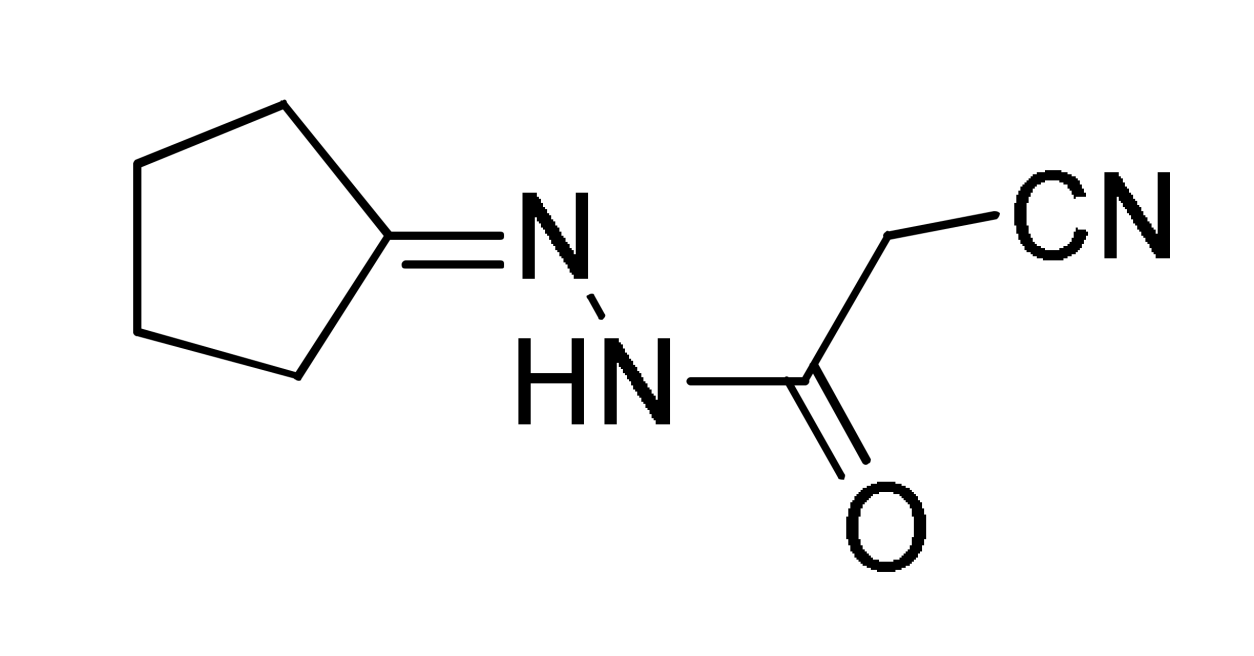 **119**	PhCHO **1**	CH_2_(CN)_2_ **12**	-----	1,4-dioxane, TEA, reflux	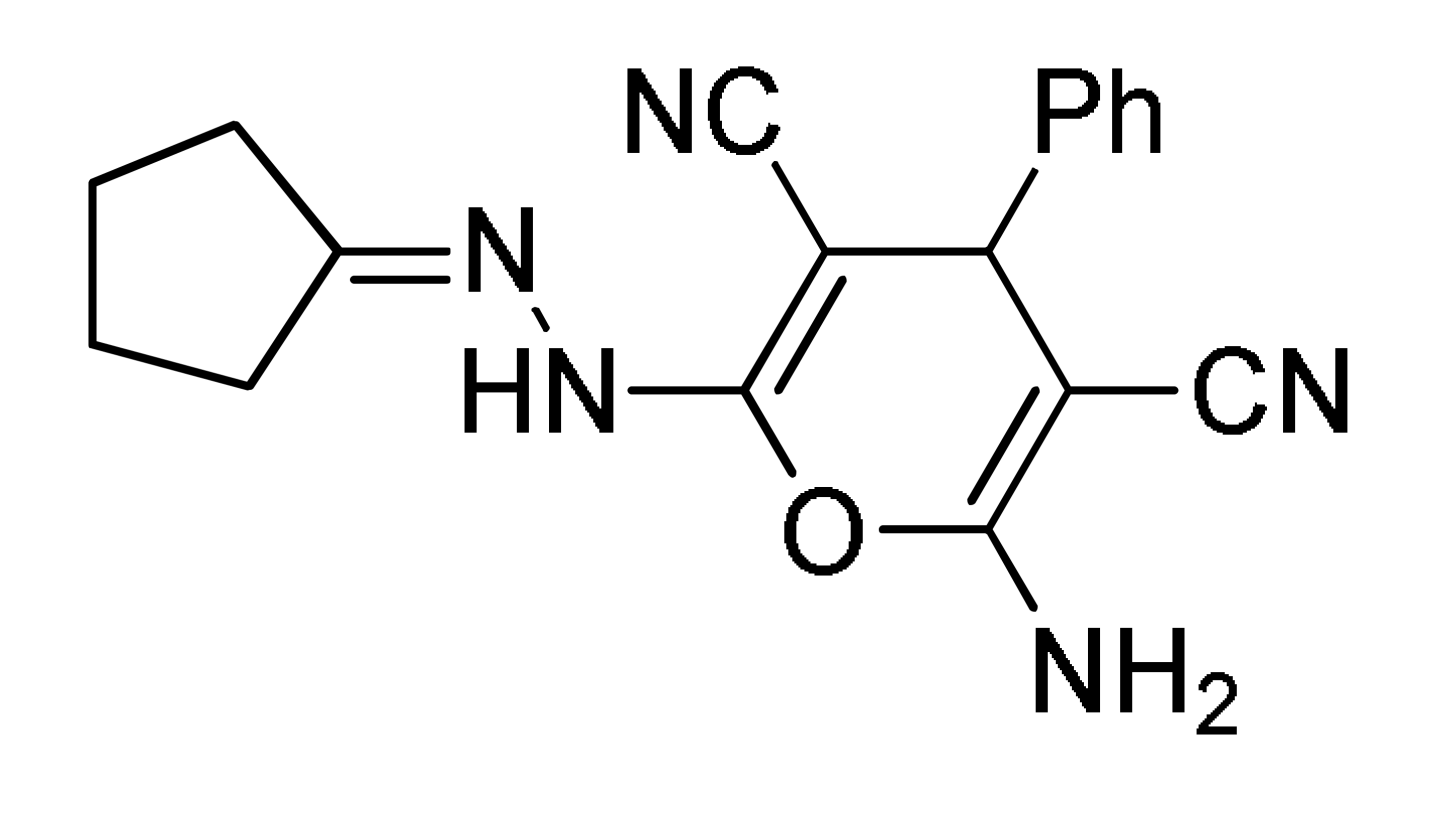 **238**	

^a^ For compounds **234/236**: X = NH_2_, OH; Y = CN, CONH_2_; Ar = Ph, 2-furyl, 4-MeOC_6_H_4_. ^b^ For compounds **235/237**: X = NH_2_, OH.

**Table 6:** Multicomponent synthesis of cyclopentanone-based thiophene/thiazol heterocyclic systems **239**–**242** of potential anticancer activity.

Reagents			Conditions	Product	Reference
S_8_	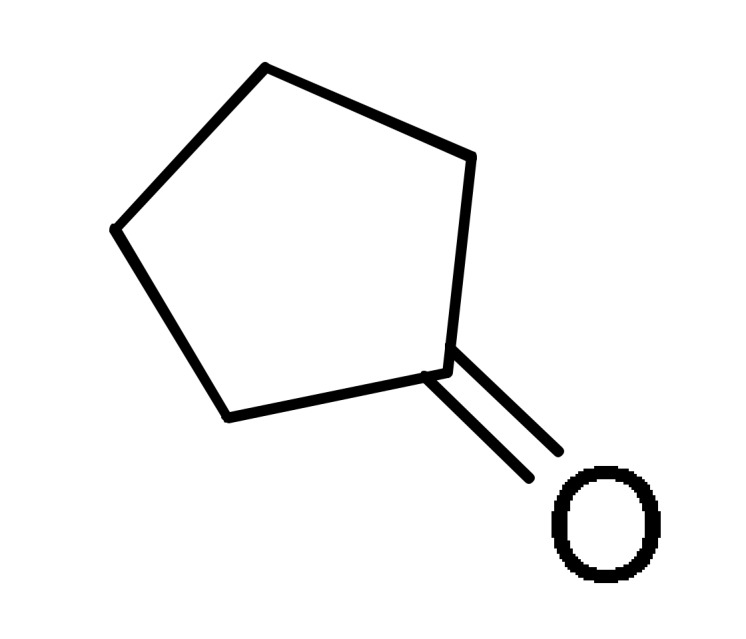 type **16**	PhNCS type **54**	EtOH, TEA, reflux	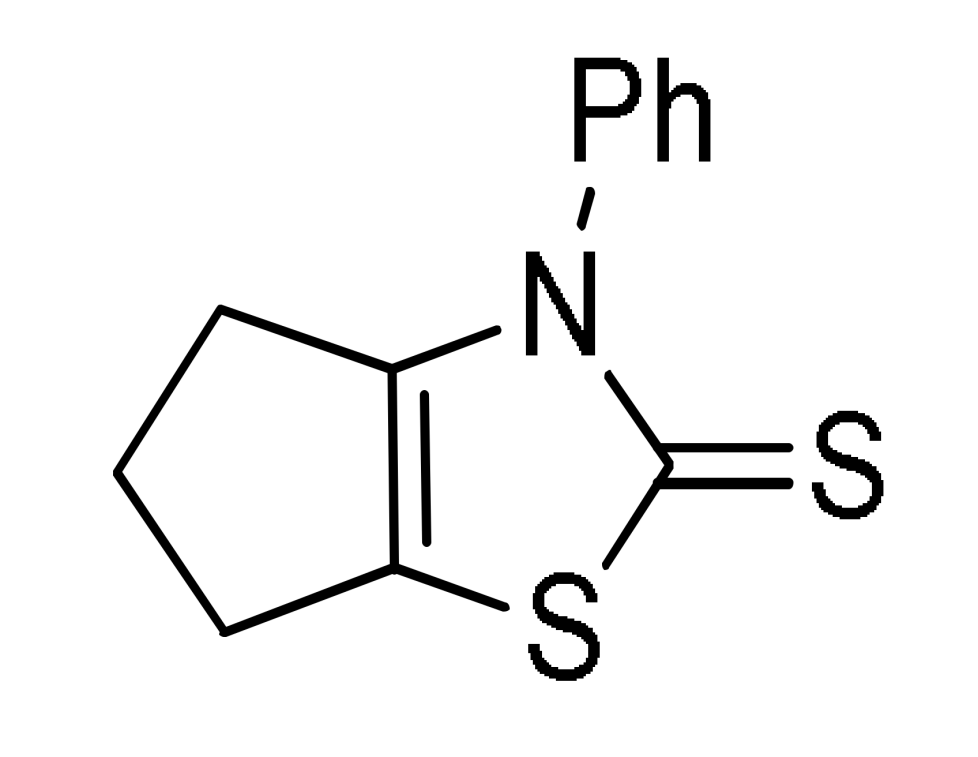 **239**	[231]
	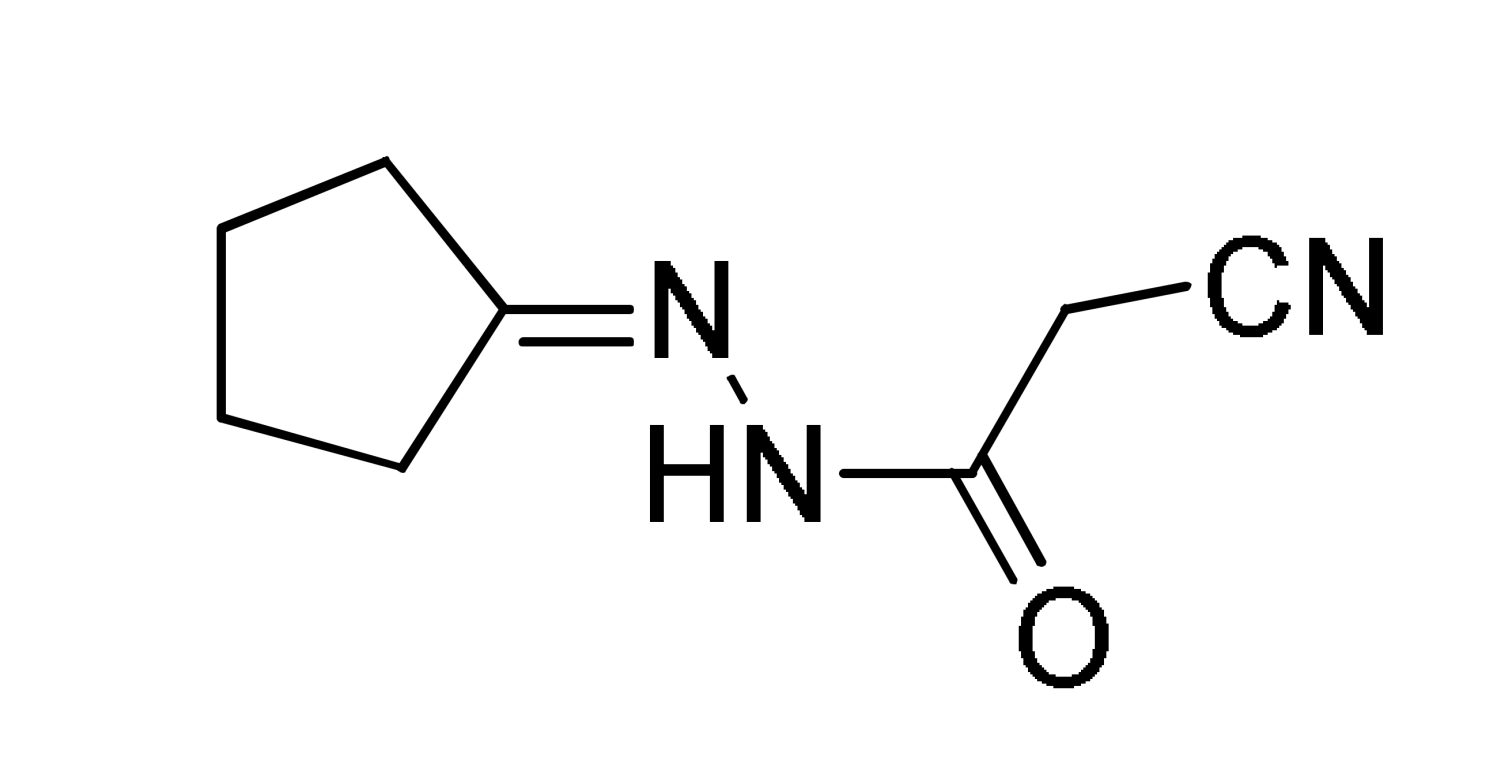 type **119**		1,4-dioxane, TEA, reflux	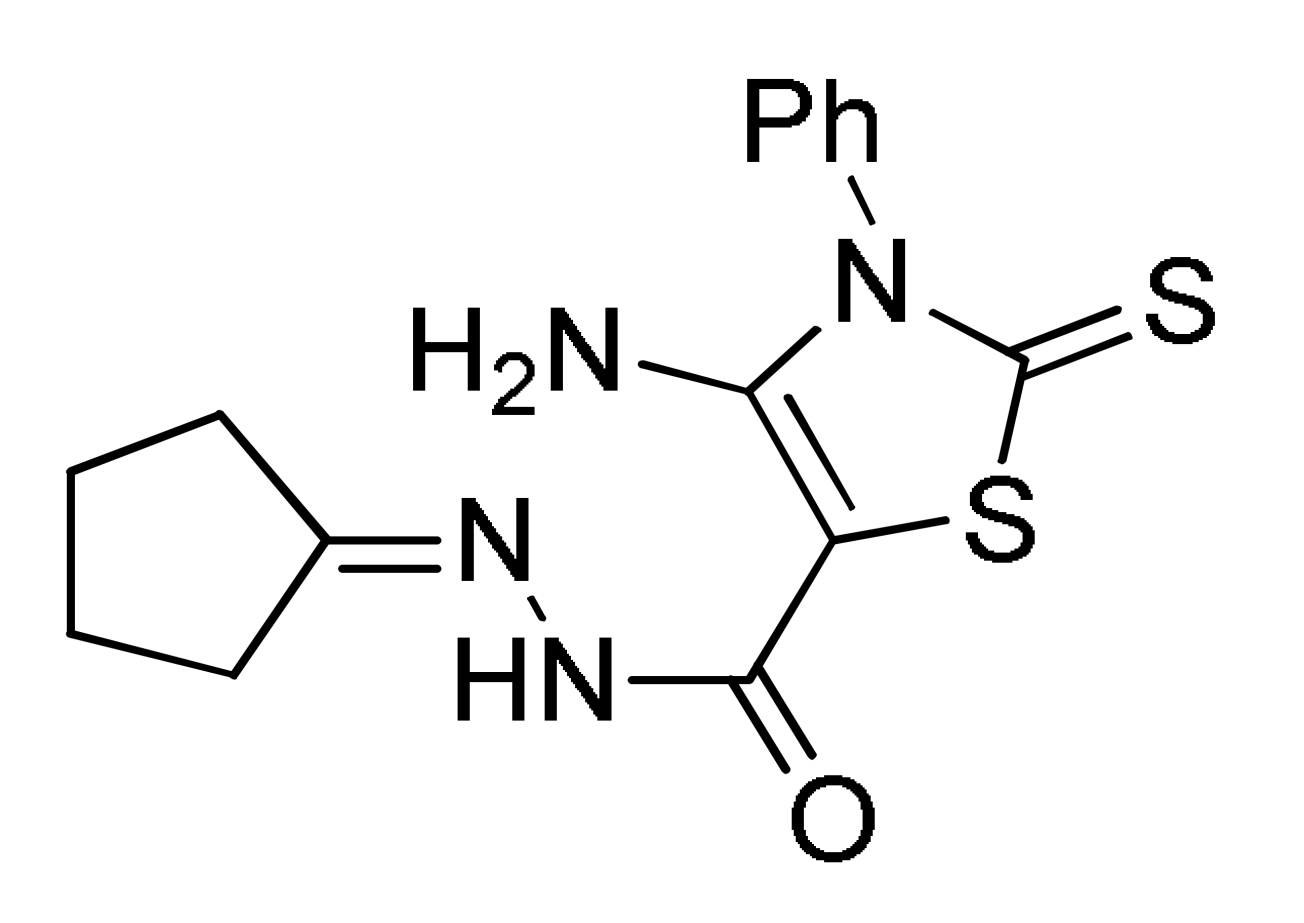 **240**	
		CH_2_(CN)_2_ **12**		**241**	
	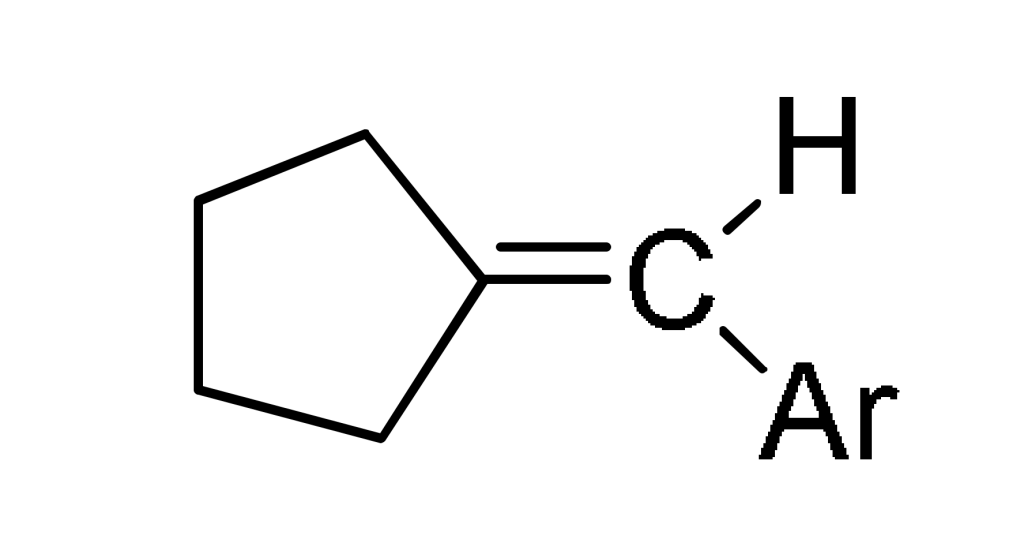 type **200**	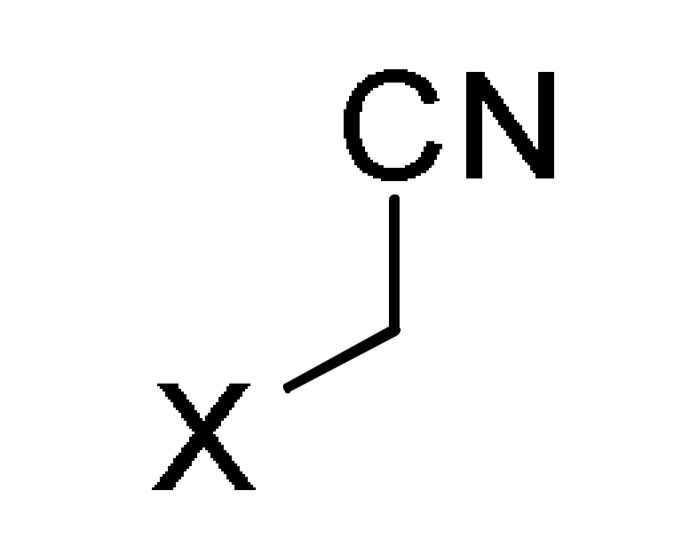 type **12/38** X = CN, CO_2_Et	EtOH, TEA, reflux	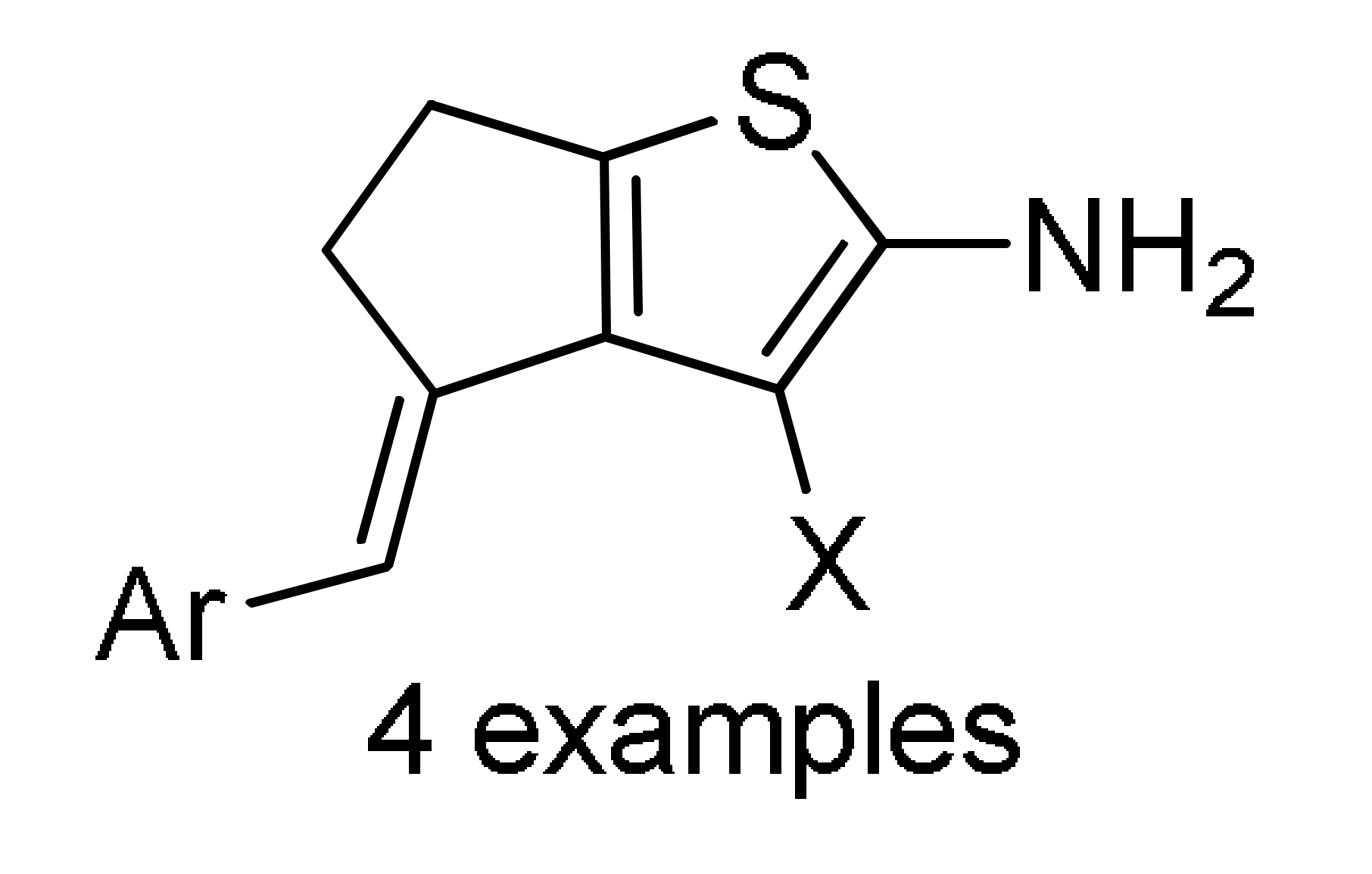 **242** ^a^	

^a^ For compounds **242**: X = CN, CO_2_Et; Ar = 4-MeOC_6_H_4_, 4-ClC_6_H_4_.
